# The Material Basis and Mechanism of Xuefu Zhuyu Decoction in Treating Stable Angina Pectoris and Unstable Angina Pectoris

**DOI:** 10.1155/2022/3741027

**Published:** 2022-01-31

**Authors:** Guanpeng Qi, Kaiwen Jiang, Jiaming Qu, Aijun Zhang, Ze Xu, Zhaohang Li, Xiaosong Zheng, Zuojing Li

**Affiliations:** ^1^School of Pharmacy, Shenyang Pharmaceutical University, Shenyang, China; ^2^School of Medical Devices, Shenyang Pharmaceutical University, Shenyang, China

## Abstract

**Methods:**

Firstly, we used a network proximity approach to calculate and compare the effectiveness of the formula with that of Western drugs for each type of angina, including all targets and intersecting targets, from a topological perspective. Secondly, we compared the mechanisms of action of the two angina pectoris at three levels and five aspects, including conventional and modular analysis approaches. Thirdly, based on the unique functions of each angina in the complex heterogeneous network, we designed a reverse process for finding the material basis using dynamic, static, and enriched items as well as a total item. Finally, the designed inverse process, material basis, and mechanism of action were validated.

**Results:**

The target network of Xuefu Zhuyu decoction is closer to the target network of each type of angina than that of Western drugs, and the intersection targets have a closer proximity. Comparison of the mechanisms of action showed that stable angina and unstable angina had 158 common targets, while the unique targets were 34 and 1, respectively. Modularity analysis showed that the GO similarity of target modules was highly correlated with KEGG similarity. We ended up with 67 compounds upregulated for stable angina and 47 compounds upregulated for unstable angina. Our results were validated by literature mining, high-volume molecular docking, and miRNA enrichment analysis.

**Conclusions:**

For both types of angina pectoris, Xuefu Zhuyu decoction is superior to Western drugs. A comparison of various aspects led to the unique mechanisms of action, from which the material basis of each type of angina was deduced.

## 1. Introduction

The principle of treating different diseases with the same treatment is a major feature of traditional Chinese medicine (TCM) treatment, reflecting the spirit of “treatment by syndrome differentiation” [1]. For example, the Qiang-Huo-Sheng-Shi decoction is effective in the treatment of both rheumatoid arthritis and osteoarthritis because they share a similar pathogenesis [2]. Another example is that although cardiovascular diseases and gastrointestinal diseases are diseases of different organs, they are molecularly related and, therefore, Sanhe decoction can modulate their common pathological processes [3]. Both of these examples illustrate that different diseases are treated in the same way due to the same pathogenesis in their development. This may be the rationale behind the law of treating different diseases together. The application of the rule of same treatment for different diseases is quite common, and its effectiveness has been tested in clinical practice [4]. However, there is still a lack of systematic methods for comparing the mechanisms of action of herbal formulas that conform to this rule in the treatment of different diseases. Looking for the differences in the mechanisms of action and exploring the material basis behind them could lead to a better understanding and development of the formula, as well as to a more precise and personalized treatment.

Coronary heart disease is one of the leading causes of death worldwide, and angina is the main type of coronary heart disease [5]. Coronary angina is an ischemic heart disease that is common in clinical cases. There are two types of angina. Stable angina (SA) is the more common form of angina and is associated with myocardial ischemia. Unstable angina (UA) is another, more severe form of angina, a heterogeneous clinical syndrome associated with a high risk of cardiac ischemic events and sudden death [6, 7]. Chinese medicine experts consider angina pectoris to be in the category of “xiong bi” in the TCM concept [8], and its main pathogenesis is blood stasis. Xuefu Zhuyu decoction is a classic formula in the category of invigorating blood circulation and resolving blood stasis and is also a representative formula for treating different diseases together [9, 10]. A series of clinical studies and meta-analyses have shown that both the Xuefu Zhuyu decoction and the derived pills or capsules are effective in both stable and unstable angina pectoris and are more effective than the commonly used Western drugs, i.e., nitrates, in certain indices [11–13]. Moreover, this formula was authoritatively recommended by the National Medical Products Administration (NMPA) as the mainstream treatment for angina pectoris [5]. However, comparative studies on the mechanism of action of Xuefu Zhuyu decoction in the treatment of the two types of angina pectoris are rare, and studies examining the material basis for the different mechanisms of action have not been retrieved. The study of the mechanisms of action and material basis of the TCM principle of treating different diseases with the same treatment using Xuefu Zhuyu decoction for stable angina pectoris and unstable angina pectoris as a real case study is of great relevance.

**Figure 1:**
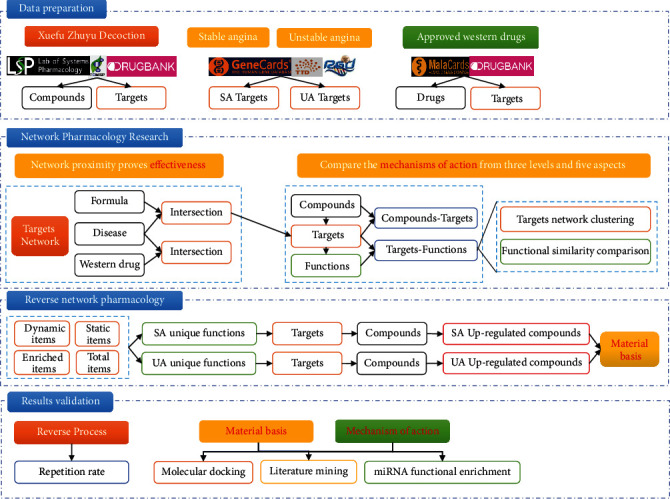
Flow chart for the study of the material basis and mechanism of action of Xuefu Zhuyu decoction.

Network pharmacology explains the function and basis of complex biological systems from a network perspective. Based on the research idea of network pharmacology, we can calculate and compare the mechanism of action of this formula for treating two types of angina pectoris from a systematic, holistic, and modular perspective, by constructing the complex networks [14, 15]. In contrast to forward network pharmacology, the reverse network pharmacology approach allows for the reverse access to the targets and components at play in the complex networks constructed from the perspective of disease characterization and function [16]. The strength of binding of a compound to different targets can be expressed in terms of the results of molecular docking [17, 18]. Furthermore, a range of compounds in a prescription can be docked to target proteins of different diseases to reveal the therapeutic characteristics of the prescription for different diseases. In the present study, we used forward network pharmacology to investigate the mechanism of action of Xuefu Zhuyu decoction in the treatment of two types of angina pectoris. The reverse network pharmacology approach was then used to study the material basis. Bulk molecular docking was used to validate the reliability of the upregulated compounds screened for the two angina pectoris species. The comparison of the similarities and differences between the mechanism of action and the material basis is the basis for understanding the treatment of different diseases with the same treatment. The flow chart of this study is shown in Figure 1.

## 2. Materials and Methods

### 2.1. Data Preparation

#### 2.1.1. Collection of Herbal Ingredients

Xuefu Zhuyu decoction is a formula made of eleven herbs, including Bupleuri Radix (CH), Paeoniae Radix Rubra (CS), Chuanxiong Rhizoma (CX), Glycyrrhizae Radix Et Rhizoma (GC), Angelicae Sinensis Radix (DG), Rehmanniae Radix (DH), Carthami Flos (HH), Platycodonis Radix (JG), Achyranthis Bidentatae Radix (NX), Persicae Semen (TR), and Aurantii Fructus (ZK). It has the effects of promoting blood circulation, removing blood stasis, promoting qi, and relieving pain. The prescription mainly treats blood stasis syndrome in the chest [19, 20].

**Figure 2:**
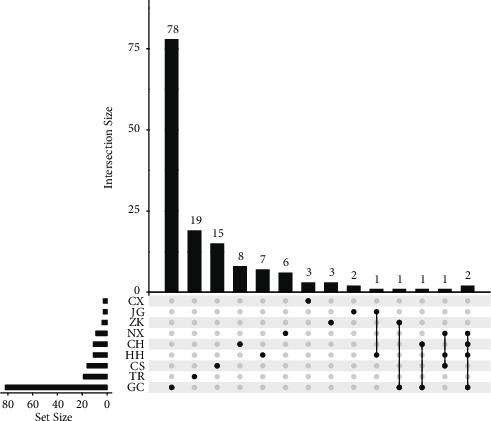
Venn diagram of the compounds contained in the nine herbs in this formula. The horizontal coordinate indicates the number of all compounds contained in the herb. The vertical coordinate indicates the size of the intersection formed by these compounds. A continuous line indicates that there is an intersection; otherwise, it indicates that there is no intersection.

The chemical components of eleven herbs were obtained through the Traditional Chinese Medicine Systems Pharmacology Database and Analysis Platform (TCMSP) [21]. The chemical components in the herbs were then screened according to Lipinski's rule [22] and the conditions of OB ≥ 30% and DL ≥ 0.18. Many studies have shown that molecules meeting OB ≥ 30% and DL ≥ 0.18 show better pharmacological properties [23]. In addition, components that meet Lipinski's five rules often have better pharmacokinetic properties and higher bioavailability during in vivo metabolism and are, therefore, more likely to be drug candidates [24]. As a result, 147 compounds were searched from eleven herbs, specifically from nine herbs, as detailed in Table S1 in the supplementary materials. The Venn diagram of the compounds contained in the nine herbs in this formula is shown in Figure 2.

#### 2.1.2. Collection of Compound-Targets

The possible targets of the ingredients were collected using TCMSP, Drugbank, and Therapeutic Target Database (TTD). A total of 125 compounds have targets. All proteins were derived from Homo sapiens and converted to standard gene names through the Uniprot database. There are 1900 interactions between compounds and proteins. See Table S2 in the supplementary materials for details.

#### 2.1.3. Collection of Disease Targets

The disease targets of stable angina and unstable angina were collected through GeneCards, TTD, and Rat Genome Database (RGD). See Table S3 in the supplementary materials for details.

#### 2.1.4. Collection of Approved Therapeutic Drugs and Their Targets

Small-molecule drugs for the treatment of these two types of angina pectoris were found in the MalaCards database [25]. The database collects drugs for the treatment of one disease and other concurrent diseases, for example, drugs for angina pectoris and hypertension. Information on therapeutic drugs is obtained from a range of databases such as DrugBank, Human Metabolome Database (HMDB), Drug Gene Interaction Database (Dgidb), and Pharmacogenomics Knowledgebase (PharmGKB) [26]. All the selected treatment drugs were approved or under investigation. We then searched their targets in the DrugBank database. The information on the targets has been supported by evidence from the literature to ensure that the information is reliable. Information on these drugs and targets is in Tables S4 and S5 in the supplementary materials.

#### 2.1.5. Protein-Protein Interaction (PPI) Network

The protein-protein interaction data were obtained from the String database [27]. The species option was set to *Homo sapiens*, and the minimum connection score will be mentioned in the next section. Disconnected nodes in the network have been hidden.

#### 2.1.6. GO and KEGG Enrichment Analysis

The data of GO and KEGG enrichment analysis were obtained through the *clusterProfiler* package (version 3.18.0) in the *R* software [28]. Both the *p* value and the *q* value calculated using the false discovery rate were less than 0.05.

#### 2.1.7. Ligands and Receptors Used for Molecular Docking

Ligands were obtained from the PubChem database, while receptors were obtained from the Uniprot database and the RCSB-PDB database. The ligands and receptors were converted to pdbqt file format in preparation for docking. The docking grid for each protein was set to the maximum to ensure coverage of the entire target protein.

### 2.2. Analysis of the Effectiveness of the Formula and Its Mechanism of Action

#### 2.2.1. Effectiveness of the Formula

Cheng et al. have demonstrated that the disease genes are likely to be in the same network neighborhood or clustered as disease modules in the human PPI network [29]. Drug target nodes usually locate in or near disease modules within the molecular network [30]. We used the network proximity index proposed by Menche et al. [31] to compare the network proximity of the formula targets and disease targets and the network proximity of approved drug targets and disease targets.

(1) $$\begin{array}{c} S_{AB}=d_{AB}-\frac{d_{A}+d_{B}}{2}, \end{array}$$

where *d*_*A*_ and *d*_*B*_ represent the average shortest distance between nodes within networks A and B, respectively, while *d*_*AB*_ represents the average shortest distance between nodes in network A and nodes in network B. The smaller the value of *d*_*AB*_, the closer the targets of the drugs are to the targets of the disease, which means that the drugs are more likely to have a therapeutic effect [32]. The average shortest distance was calculated using the *igraph* package (version 1.2.6) in *R* 4.0.2.

#### 2.2.2. Comparison of Network Proximity of Formula Targets, Disease Targets, Approved Drug Targets, and Intersection Targets

We used the network proximity index described above to compare the targets of this formula with the disease targets of the two classes of angina pectoris and the targets of approved drugs. At the same time, to demonstrate that the intersection of the targets of the formula with the disease targets can be used in subsequent studies, the targets of the Chinese medicine and the targets of the Western medicine were taken to intersect with the disease targets separately to facilitate the comparison of topological similarity using the network proximity index again.

In Section 3.1.2, we initially demonstrate that the intersection targets of the formula and the disease were closer to the disease targets than the full formula targets in terms of topical structure, so we then used the intersection targets for subsequent studies.

### 2.3. Similarities and Differences in the Mechanism of Action of the Formula

The treatment of stable angina pectoris and unstable angina pectoris with Xuefu Zhuyu decoction belongs to the content of simultaneous treatment of different diseases in traditional Chinese medicine. After the effectiveness of this formula has been proven, it will be valuable to investigate the similarities and differences in the mechanisms of action of the formula in the treatment of the two types of angina pectoris.

We explored the similarities and differences of the action mechanism of the formula in the two disease conditions from the three levels of ingredient, target, and function. Specifically, the five aspects of formula compound, target, compound-target, function, and target-function were compared for the two disease conditions. The results of the comparison of these five aspects are shown in Section 3.2.

The therapeutic action of the formula is reflected in the functions produced by its numerous targets [33]. We next analyzed the function of the targets using network clustering and functional similarity methods. Two PPI-weighted networks were constructed to divide functional modules, and GO similarity [32] and KEGG similarity [34] were used to compare the mechanism of action of the formulas when treating stable angina versus unstable angina. Furthermore, we calculated the Spearman correlation coefficient between the GO similarity and KEGG similarity of the modules to explore the feasibility of using GO or KEGG for subsequent studies.

### 2.4. Three Complex Network-Based Approaches to Explore the Therapeutic Ingredients of the Formula

#### 2.4.1. Random Walk with Restart on the Complex Heterogeneous Network (Dynamic Item)

**Figure 3:**
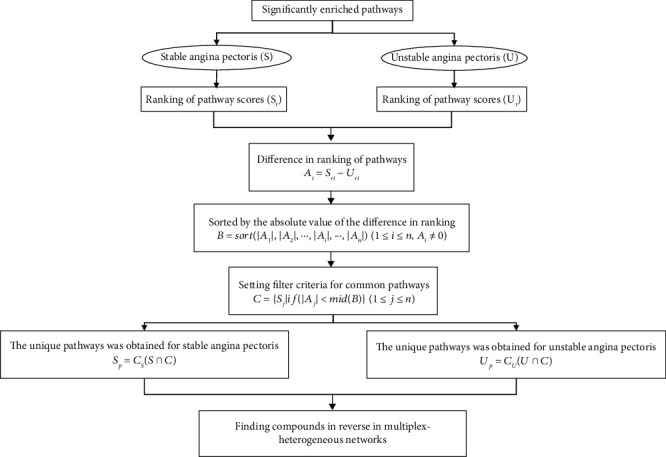
Calculation process for common and unique pathways in two disease conditions. All rankings were calculated in descending order.

**Figure 4:**
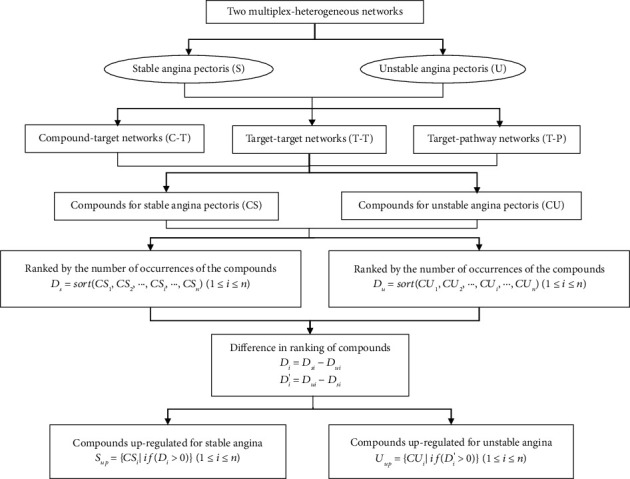
Calculation process for upregulated compounds vs. downregulated compounds. The meaning of upregulated and downregulated compounds is relative. An upregulated compound in one disease condition is a downregulated compound in another disease condition. Nodes in the T-T networks were considered only for their primary and secondary nodes. All rankings were calculated in descending order.

Complex heterogeneous networks [34] for stable angina and unstable angina were constructed based on the compound-target network (C-T), target-target network (T-T), and target-pathway network (T-P). To ensure that the target-target interactions were reliable, the interaction score was set to be greater than or equal to 0.9. The construction of the network and the reverse finding of ingredients in the network are carried out according to the following steps:

1. Constructing complex networks: two complex heterogeneous networks were constructed based on the compound-target network, target-target network, and target-pathway network.
2. Random walk with restart (RWR): random walk on two complex heterogeneous networks with all active compounds as seeds, with a restart probability of 0.7 as default [35]. The RWR algorithm was performed by package *RandomWalkRestartMH* (version 1.10.0) in *R* 4.0.2.
3. Score and ranking of results: we obtained the ranking of the importance of the pathways and obtained the common and unique pathways based on the process shown in Figure 3.
4. Finding compounds in the network in reverse: based on the unique pathways obtained in the two disease conditions, the compounds are then found in the network in reverse. An algorithm was constructed to obtain the upregulated versus downregulated ingredients of the formula based on the ranking of the importance of the obtained compounds. The process is shown in Figure 4.

#### 2.4.2. Calculation of the Degree Value of Pathways Containing Key Genes (Static Item)

The degree values of the pathways were calculated independently in two large complex networks. The degree value of a pathway represents the number of genes contained in that pathway, and the degree value alone does not fully reflect the importance of the pathway. It has been shown that key genes in a pathway can play an important role in that pathway [36], so we added experimentally identified human key genes [37] to the calculation of the degree values and used the following formula to calculate and rank the scores of the pathways. Subsequently, the process shown in Figures 3 and 4 was followed to find the compounds in the network in reverse.

(2) $$\begin{array}{c} S_{O}=\frac{S_{i}-S_{\min}}{S_{\max}-S_{\min}}, \end{array}$$

(3) $$\begin{array}{c} S=S_{o}\cdot S_{k}, \end{array}$$

where *S*_*i*_ is the degree value of a pathway and *S*_max_ and *S*_min_ represent the maximum and minimum values of the degree values of all pathways, respectively. *S*_*k*_ represents the percentage of the number of key genes in the pathway.

#### 2.4.3. Calculation of the Enrichment Effect of the Pathway (Enrichment Item)

The value of fold enrichment is the ratio of GeneRatio to BgRatio and represents the extent to which the target under study is enriched in a particular pathway and the importance of that pathway. Fold enrichment was used as a score to rank the importance of the pathways, and then, the compounds were found according to the process shown in Figures 3 and 4.

#### 2.4.4. A Formula including Static, Dynamic, and Enriched Items Was Devised

By integrating the three previous approaches based on complex networks, a formula including static, dynamic, and enriched items was devised to find the ingredients in the network in reverse. The form of the formula named total item is as follows:

(4) $$\begin{array}{c} X_{\text{nor}}=\frac{X-X_{\min}}{X_{\max}-X_{\min}}\;\left(X\;\in \;S,\;D,\;F\right), \end{array}$$

(5) $$\begin{array}{c} T=S_{\text{nor}}+D_{\text{nor}}+F_{\text{nor}}. \end{array}$$

*S*, *D*, and *F* are referred to as static, dynamic, and enriched items, respectively, while *T* represents total item. Firstly, the values of *S*, *D*, and *F* are normalized according to equation (4). These three items were then given the same weight by default. Finally, the three items are summed to form the total item. We defined *S*, *D*, and *F* to represent the aforementioned *S* value, RWR score, and fold enrichment value, respectively. The subsequent process of finding compounds in the network in reverse is the same as in the first three parts.

#### 2.4.5. Assessment of the Designed Reverse Process

The unique pathways for the two disease conditions obtained from the scores of the three items, static, dynamic, and enrichment, and the total item were the source and key point of the designed reverse process. We compared the unique pathways obtained from the three items and the total item and next compared the compounds obtained from the reverse process. The comparison method is the repetition rate of the results for each item. The calculation of the repetition rate of a pathway is different from the calculation of the repetition rate of a compound because we have assumed that the repetition rate of a pathway may be large and the repetition rate of a compound may be low. In practice, however, for our reverse search process in complex networks, the lower the repetition rate of pathways, the better and the higher the repetition rate of compounds the better. So, with the aim of overturning the original hypothesis, we have devised the following six formulas. Firstly, the repetition rate of the pathways was calculated.

(6) $$\begin{array}{c} Z_{1}=\frac{S_{p}\cap^{\;}D_{p}\cap^{\;}F_{p}}{S_{p}\cup^{\;}D_{p}\cup^{\;}F_{p}}, \end{array}$$

(7) $$\begin{array}{c} Z_{2}=\frac{S_{P}\cap^{\;}D_{P}\cap^{\;}F_{P}\cap^{\;}T_{P}}{S_{P}\cup^{\;}D_{P}\cup^{\;}F_{P}\cup^{\;}T_{P}}, \end{array}$$

(8) $$\begin{array}{c} Z_{3}=\frac{\max\left(S_{p}\cap^{\;}D_{p},S_{p}\cap^{\;}F_{p},D_{p}\cap^{\;}F_{p}\right)}{\min\left(S_{p}\cup^{\;}D_{p},S_{p}\cup^{\;}F_{p},D_{p}\cup^{\;}F_{p}\right)}, \end{array}$$

(9) $$\begin{array}{c} Z_{4}=\frac{\max\left(S_{p}\cap^{\;}D_{p},S_{p}\cap^{\;}F_{p},S_{p}\cap^{\;}T_{p},D_{p}\cap^{\;}F_{p},D_{p}\cap^{\;}T_{p},F_{p}\cap^{\;}T_{p}\right)}{\min\left(S_{p}\cup^{\;}D_{p},S_{p}\cup^{\;}F_{p},S_{p}\cup^{\;}T_{p},D_{p}\cup^{\;}F_{p},D_{p}\cup^{\;}T_{p},F_{p}\cup^{\;}T_{p}\right)}. \end{array}$$

*S*_*p*_, *D*_*p*_, *F*_*p*_, and *T*_*p*_ represent the set of unique pathways obtained from static, dynamic, enriched, and total items, respectively. Also, when calculating the duplication rates of compounds, they represent the set of upregulated compounds obtained from the abovementioned four items, respectively. When calculating the repetition rate of the compounds, *Z*_3_ and *Z*_4_ take of the following form:

(10) $$\begin{array}{c} Z_{3}^{\prime }=\frac{\min\left(S_{p}\cap^{\;}D_{p},S_{p}\cap^{\;}F_{p},D_{p}\cap^{\;}F_{p}\right)}{\max\left(S_{p}\cup^{\;}D_{p},S_{p}\cup^{\;}F_{p},D_{p}\cup^{\;}F_{p}\right)}, \end{array}$$

(11) $$\begin{array}{c} Z_{4}^{\prime }=\frac{\min\left(S_{p}\cap^{\;}D_{p},S_{p}\cap^{\;}F_{p},S_{p}\cap^{\;}T_{p},D_{p}\cap^{\;}F_{p},D_{p}\cap^{\;}T_{p},F_{p}\cap^{\;}T_{p}\right)}{\max\left(S_{p}\cup^{\;}D_{p},S_{p}\cup^{\;}F_{p},S_{p}\cup^{\;}T_{p},D_{p}\cup^{\;}F_{p},D_{p}\cup^{\;}T_{p},F_{p}\cup^{\;}T_{p}\right)}. \end{array}$$

### 2.5. Three Methods for Validation of Results

#### 2.5.1. Literature Mining

We searched the PubMed database and the Web of Science database for literature on the therapeutic effects and mechanisms of action of the compounds in this formula. Specifically, a systematic search was conducted for literature in which compounds were linked to cardiovascular diseases and for literature in which compounds acted on possible therapeutic targets. In particular, we deliberately focused on literature related to the therapeutic effects and mechanisms of action of up- and downregulated ingredients. Literature identification numbers are recorded in Tables S6 and S7 in the supplementary materials.

#### 2.5.2. Molecular Docking

The compounds up- and downregulated in each of the two disease conditions were molecularly docked to the unique targets contained in each of the two diseases. The molecular docking software used was PyRx 0.8 and AutoDock Vina 1.1.2 [38]. If the overall binding of the upregulated compounds was better than that of the downregulated compounds, then it can be shown that our process of finding compounds in reverse in the network was essentially correct.

In addition, to further confirm the plausibility of the designed inverse process, 50 compounds were randomly selected as random ingredients from the TCM@Taiwan database of over 60,000 compounds for molecular docking with the abovementioned targets without put-back sampling. The docking results of the random compounds were compared with the results of the upregulated compounds and the results of the downregulated compounds. In the end, we performed a total of 3990 molecular docking sessions. The specific comparison methods and binding are shown in the results section.

#### 2.5.3. Indirect Evidence from the Gene Expression Omnibus Database

Most of the targets described above are protein products produced by the encoded genes. Considering the combined effects of target gene expression and regulation of target gene expression on disease function, miRNAs were explored as important regulators of the regulation of target gene expression [39]. Specifically, we obtained experimentally measured miRNA expression data for stable angina versus unstable angina from the Gene Expression Omnibus (GEO) database (GSE66752 and GSE94605).

Specifically, firstly, we obtained data on experimentally measured miRNA expression in the GEO database for stable angina and unstable angina, and secondly, we performed differential expression analysis using the GEO2R analysis tool. The screening condition for miRNA was set to |logFC| >2 and *p* < 0.05 [40]. Afterwards, the miRabel database [41] was searched for miRNA targets that were experimentally confirmed and had a score of >0.5. Finally, KEGG enrichment analysis of target genes was performed using the *clusterProfiler* package (version 3.18.0) in *R* 4.0.2. The results of the miRNA functional enrichment analysis were compared with the results of the enrichment analysis of the crossover genes described above and with the results of the designed reverse process. If the duplication rate of the pathway is high, then it can indirectly prove that our forward process and reverse process are reliable.

## 3. Results

### 3.1. The Effectiveness of the Formula and the Feasibility of Studying the Intersectional Targets

#### 3.1.1. The Formula Is Effective and Superior to Approved Western Drugs

There were 3321 and 1626 targets for stable angina and unstable angina obtained by searching the databases, respectively. The total number of targets for this formula after deduplication was 230. In addition, there are 398 and 221 targets for approved Western drugs for the two types of angina, respectively. Information on the formula, diseases, and approved Western drugs is recorded in detail in Table 1.

The network proximity approach compares the topological similarity of two dense networks, where the similarity of the topology of the two networks represents the possibility that the two networks perform some similar functions [42]. Specifically, we use the separateness method of the network proximity approach to measure the proximity of two networks [30]. In terms of equation (1), a smaller value of *d*_*AB*_ represents a closer proximity of the two networks.

The results in Table 2 present a comparison of the network proximity of this formula and approved Western drugs to each of the two angina pectoris. The results show that the targets of the formula are always closer to the targets of the disease than the targets of the Western drugs in terms of topology, for both stable and unstable angina. Therefore, these results suggest that the treatment of both stable and unstable angina pectoris with Xuefu Zhuyu decoction is effective and superior to approved Western drugs.

#### 3.1.2. Proximity of Intersecting Targets to Disease Targets

The results in Table 2 also illustrate that the intersection targets of the formula and diseases are closer to the disease targets than the intersection targets of Western drugs and diseases, and such results are consistent with the results in Section 3.1.1.

In addition, a more important point illustrated in Table 2 is that when the targets of the drugs are taken to intersect with the targets of the two angina pectoris, the intersecting targets is closer to the targets of the disease, both for the formula and the Western drugs. Such results lead us to realize that their intersecting targets with the disease are feasible for the next studies, regardless of whether they are prescription or Western drugs. Subsequent studies with intersecting targets may lead to a more relevant material basis or mechanism of action for the disease.

### 3.2. The Mechanism of Action Was Compared from Three Levels and Five Aspects

#### 3.2.1. Comparison of Similarities and Differences in the Usual way

We calculated and compared the number of compounds that actually acted on the target in the two complex heterogeneous networks. We then also calculated and compared the number of intersecting targets on which the compounds acted and the number of enriched GOs and KEGGs derived from this extension. BP is the most numerous and dominant type of enrichment in GO enrichment analysis; therefore, we used GO-BP for the follow-up study [33]. In the screening conditions, *p* value and *q* value were set to less than 0.05. The results of the comparison of compounds, targets, and functional enrichment are shown in Table 3.

**Figure 5:**
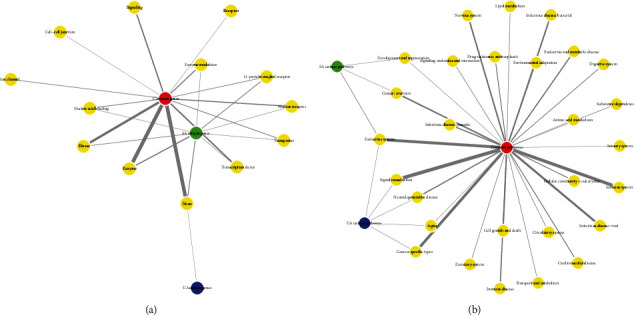
Classification and comparison of targets and pathways in the two types of angina. (a) Classification of targets. (b) Classification of pathways. The thickness of the connecting line represents the number of targets or pathways classified under that item. SA represents stable angina, and UA represents unstable angina.

In terms of compound-target, that is, which types of targets the compounds acted on, we have analyzed the actual targets on which compounds act in the complex network. A comparison of the classification of target proteins in the two angina conditions is shown in Figure 5(a). We can find that the targets common to both types of angina are mostly focused on enzymes, kinases, transcription factors, and signaling molecules. In comparison, there are no signaling molecules among the targets unique to stable angina, while the importance of G-protein-coupled receptors and enzyme modulators, for example, increases. The one target unique to unstable angina is currently not clearly classified.

We then analyzed the classification of the pathways, and the results are shown in Figure 5(b). As can be seen from the figure, the pathways common to both types of angina are classified in the main areas of signal transduction, the immune system, the endocrine system, specific types of cancer, and infectious diseases. The number of pathways included in each of these five broad pathway categories is greater than 10. Pathways unique to stable angina are classified in the areas of endocrine system, cancer, development, and regeneration, while pathways unique to unstable angina are classified in the areas of neurodegenerative disease, signal transduction, endocrine system, ageing, and specific types of cancer.

We can conclude that both types of angina have a significant proportion of common components in terms of target classification and pathway classification, which may underlie their common pharmacological effects. That is, the common action on common targets and pathways produces a common mechanism of action. However, both classes of angina have a proportion of unique targets and unique pathways. Also, even within the common targets and pathways, the ranking of their importance is different.

#### 3.2.2. Comparison of Similarities and Differences Using Modular Analysis

Modular analysis was used to compare the target-functional aspects of the two classes of angina [43]. We obtained the protein-protein interaction network (PPI) for stable angina and unstable angina on the basis of defaulting the intersection targets of the formula action as the therapeutic targets, and the interaction score was set to be greater than or equal to 0.4 to ensure that as much interaction information as possible was included. Next, the two PPI networks were divided into modules using the fast greedy algorithm, the infomap algorithm, and the walktrap algorithm, respectively [44], and the interaction scores were used for weighting. In other words, this process actually clustered the two large weighted networks using each of the three network clustering algorithms and then analyzed the modules obtained from the clustering.

**Figure 6:**
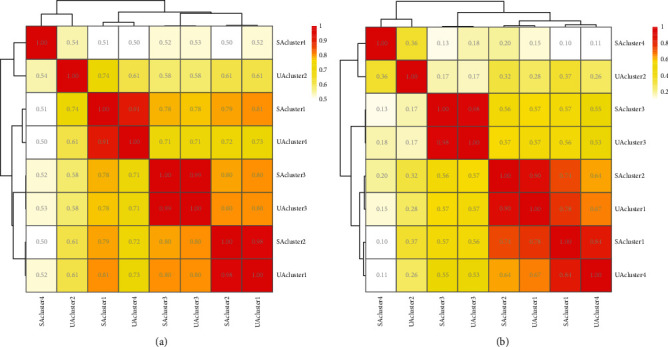
The comparison of the functional similarity of the modules classified by the network clustering. (a) Heat map and hierarchical clustering for the comparison of GO similarity. (b) Heat map and hierarchical clustering for the comparison of KEGG similarity. The colors and values of the small squares represent the functional similarity between modules. Darker colors and larger values indicate higher similarity.

Our results have shown that both networks clustered with the fast greedy algorithm outperformed the other two algorithms on the metric of modularity index [44], so we used the fast greedy algorithm for network clustering. The results of the comparison of the modularity index are presented in Table S8 in the supplementary materials. The results of the network clustering showed that both weighted PPI networks were divided into four modules, and we then compared these modules for GO similarity and KEGG similarity. For the specific calculations, the GO similarity was calculated using the GOSemSim package (version 2.16.1), while the KEGG similarity was calculated using the BioCor package (version 1.14.0). The results of the comparison of the functional similarity of the modules are shown in Figures 6(a) and 6(b).

The results of the comparison of the functional similarity of the modules illustrate several conclusions. Firstly, the results of the comparison of GO similarity and KEGG similarity for each module are highly consistent. At this level, it can be shown that the use of either GO or KEGG to indicate the functions of the targets is reliable and that the two can be substituted for each other. Secondly, the modules for stable angina and unstable angina always cluster together, for example, SAcluster1 and UAcluster4, SAcluster2 and UAcluster1, SAcluster3 and UAcluster3, and SAcluster4 and UAcluster2. This suggests that stable angina and unstable angina are very close in terms of the function of the divided modules, which may be the basis for the formula to work in treating different diseases together. Finally, there are still differences between the modules for stable angina and those for unstable angina. For example, in the clustering of GO similarity and KEGG similarity, SAcluster4 and UAcluster2 are clustered together, but the two functional similarities between the two modules are only 0.54 and 0.36, respectively.

We then calculated the Spearman correlation coefficient between the two functional similarities, and the results show an extremely strong correlation. The results of the correlation coefficients are shown in Table 4.

### 3.3. Results of Three Complex Network-Based Methods for Reverse Lookups in Networks

#### 3.3.1. Scores for the Three Complex Network-Based Approaches

**Figure 7:**
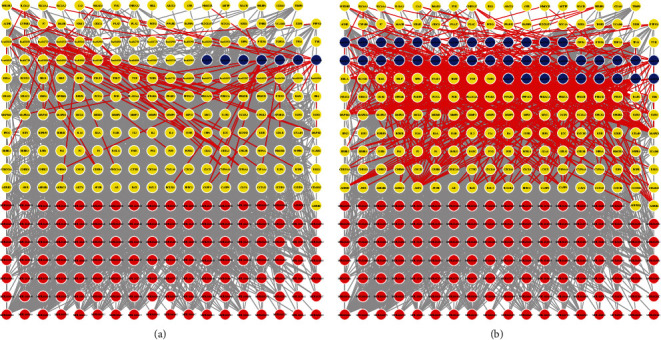
Schematic representation of the results of random wandering with restart in two complex heterogeneous networks. (a) Random wandering with restart in stable angina. (b) Random wandering with restart in unstable angina. Red nodes represent compounds, yellow nodes represent targets, and blue nodes represent pathways. Only the seed nodes and the top two hundred scoring nodes in the complex network are shown.

Running the restart random wandering algorithm in a constructed complex network gives the score of a node or some nodes (seed nodes) with any other nodes, and this score can indicate the strength of the relationship between the nodes [45, 46]. The meaning of restart can be equated to the fact that the seed node may or may not perform a wander; i.e. it is given a restart probability before performing a wander. We performed restart random wandering in two complex heterogeneous networks using all the compounds that acted as seed nodes and then obtained the scores of these seed nodes on the pathways. A schematic of these two random walks is shown in Figures 7(a) and 7(b).

**Figure 8:**
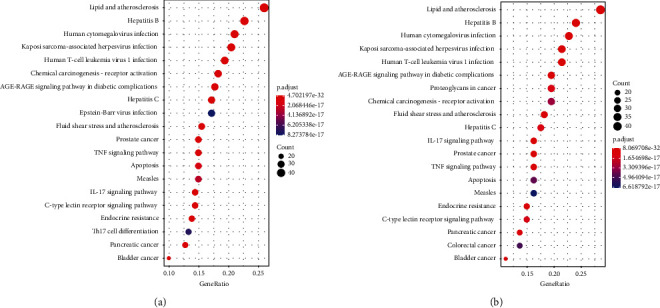
Bubble plot of the results of the KEGG significant enrichment analysis. (a) KEGG enrichment analysis in stable angina. (b) KEGG enrichment analysis in unstable angina. Only the top 20 significantly enriched pathways are shown. GeneRadio indicates the ratio of the number of genes enriched in the pathway to the number of genes studied.

We have shown in the previous section that GO similarity correlates extremely well with KEGG similarity, so in enrichment analysis, either GO or KEGG enrichment analysis can be used to represent the therapeutic effect produced by the targets. Therefore, we chose the results of the KEGG enrichment analysis as the source for the reverse process of the enrichment term. The results of the KEGG significant enrichment analysis are shown in Figures 8(a) and 8(b). In practice, we used the ratio of GeneRadio to BgRadio to represent the scores of enriched terms.

We found that these three complex network-based approaches had different emphases in their scores across all pathways. The dynamic, static, and enrichment terms all had their own tendency to score high on the pathways. In other words, each pathway has a different tendency to score differently on the dynamic, static, and enrichment terms, and we have drawn a graph of the tendency to score differently on the pathways in Figures S1 and S2 in the supplementary materials. However, all three items are important. Therefore, we designed a total item combining dynamic, static, and enrichment items to indicate the importance of the pathways.

#### 3.3.2. Evaluation of the Results of Dynamic, Static, Enrichment, and Total Items

**Figure 9:**
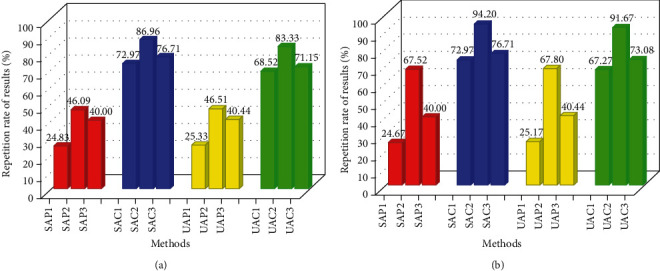
Percentage stacked plots of repeat rate of compounds and repeat rate of pathways. (a) Repetition rate of results based on dynamic, static, and enriched items. (b) Repetition rate of results based on dynamic, static, enriched, and total items. SA refers to stable angina, while UA refers to unstable angina. P represents pathway, while C represents compound. In (a), numbers 1, 2, and 3 represent equations (6), (8), and (10), respectively, while in (b), numbers 1, 2, and 3 represent equations (7), (9), and (11), respectively.

The basis and focus of the scores for dynamic, static, and enrichment items are different, as can be seen from the ranking of the importance of the pathways. As can be seen in Figure 9(a), the unique pathways for stable angina and unstable angina that we obtained based on the scores for the three items mentioned above have a repeat rate of less than 47%. The majority of the results are only 40% and below. There are three formulas used to calculate the repeat rate of unique pathways, numbers 1, 2, and 3, corresponding to equations (6), (8), and (10), respectively. Equations (8) and (10) were calculated using different numerators and denominators, and the results represent the two extreme values of the maximum and minimum values for this calculation condition. In Figure 9(b), the results of the pathways with the total term are added to the calculation, so numbers 1, 2, and 3 correspond to equations (7), (9), and (11), respectively. Equations (9) and (11) also represent the maximum and minimum values for that calculation condition, respectively. As can be seen in Figure 9(b), when the results of the pathway for the total term were added to the calculation, the repetition rate calculated by equation (9) is suddenly much higher. Such results suggest that the total term is the result of a combination of dynamic, static, and enrichment terms and that the scores for the importance of the pathways bring together the characteristics of each of these three terms.

However, despite the different focus of the pathway scores and the relatively low repetition rates of the pathways, the compounds obtained from the reverse process all have repetition rates close to or above 70%, with some results close to or even above 90%. These results indicate that the upregulated compounds obtained for that disease condition are always relatively consistent, whether they are dynamic, static, enriched, or total terms. In other words, the functions played by some important and unique compounds encapsulate the functions characterized by the dynamic, static, enrichment, and total terms. All four processes for finding compounds in reverse in the network based on the four items mentioned above are feasible because of the high duplication rate of the upregulated compounds found.

#### 3.3.3. Unique Pathways and Upregulated Compounds in Stable Angina versus Unstable Angina

**Figure 10:**
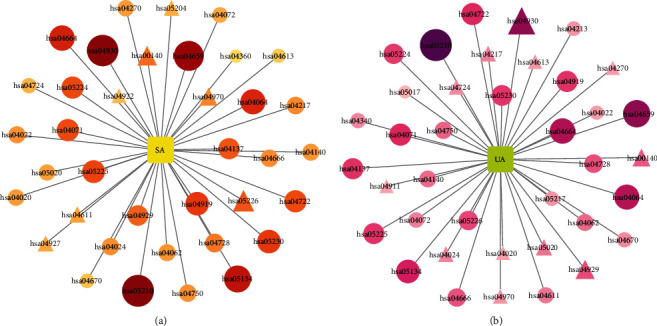
Pathways unique to stable angina versus unstable angina. (a) Pathways unique to stable angina. (b) Pathways unique to unstable angina. The size of the nodes and the shade of color represent the value of the fold enrichment. The darker the color and the larger the node, the greater the value of fold enrichment. Circular nodes indicate unique pathways where dynamic, static, enriched, total terms, and miRNA functions coexist. Triangular nodes indicate unique pathways shared by dynamic, static, enriched, and total terms.

The unique pathways for the two types of angina obtained from the dynamic or static or enriched terms were different, and we took duplicate values for the results obtained based on the three mentioned above, i.e., the unique pathways calculated for all three mentioned above. As for the total term, we found that the results of all three terms after taking duplicate values were in the results of the total term, for both types of angina. This is another indication that the results of the total term are a fusion of the results of the three previous terms. The unique pathways for both angina categories are shown in Figures 10(a) and 10(b). We can see that some pathways are unique for both angina categories; however, their importance ranking is different, see Tables S9 and S10 in the supplementary materials. The node size in the figure refers to fold enrichment, i.e., the relative enrichment of the target in that pathway, not the importance ranking.

After reverse search and calculation, there were 67 and 47 upregulated compounds for stable angina and unstable angina, respectively. In fact, the upregulated compounds for stable angina are the downregulated compounds for unstable angina and vice versa. To further demonstrate the reliability of the designed inverse process, we increased the screening condition among the upregulated compounds to find selected compounds, as described in Tables S12 and S13 in the supplementary materials. The screening condition was that the importance rank of the upregulated compounds was greater than the median of the importance rank, resulting in 30 selected upregulated compounds for stable angina and 19 selected upregulated compounds for unstable angina. Both the upregulated compounds and the selected upregulated compounds are in Tables S12 and S13 in the supplementary materials. We next validated both the upregulated compounds and the selected upregulated compounds.

### 3.4. Validation of Results and Evaluation of the Process

#### 3.4.1. Literature Mining Provides Evidence from the Literature

We used PubMed and Web of Science literature databases to systematically collect literature on the effects and mechanisms of action of all compounds and the calculated upregulated compounds. The results of the literature search for the therapeutic effects and mechanisms of action of the selected upregulated compounds were also listed.

**Figure 11:**
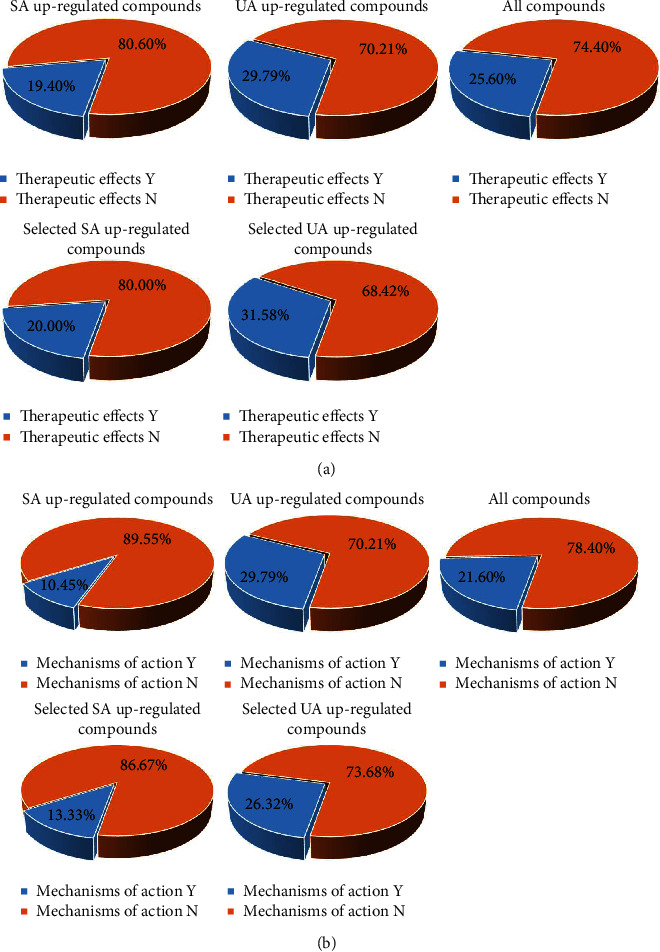
3D fan chart of the literature validation rates for therapeutic effects and mechanisms of action. (a) Literature validation rates for the therapeutic effects of compounds. (b) Literature validation rate of compounds' mechanisms of action. Y indicates supported by literature evidence, and N indicates not supported by literature evidence.

In terms of literature mining for therapeutic efficacy, i.e., whether it works, the literature validation rate ranged from 20% to 30%, with some results above 30%. From the results in Figure 11(a), it appears that the literature validation rate for selected upregulated compounds is higher than for unselected upregulated compounds. This suggests that the proportion of upregulated compounds with literature evidence of association with cardiovascular disease has increased after more stringent screening. In terms of the mechanism of action, i.e., how it worked, the literature validation rate for which targets the compounds acted on was slightly lower, between 10% and 30%, as shown in Figure 11(b). However, the literature validation rate for selected upregulated compounds in stable angina was still higher than for unselected compounds, still supporting the abovementioned pattern. It is important to note that the absence of literature evidence supporting an association with cardiovascular disease or with those targets collected does not mean that the compound does not have a therapeutic function in angina, as the compound may act on other aspects of the body to produce efficacy in angina [47]. For example, most compounds have been searched in the literature in relation to inflammatory, hormonal, and psychiatric disorders [48–50]. There are also approved Western drugs that treat angina through hormonal and psychiatric aspects [51, 52].

Overall, the upregulated compounds obtained by reverse search in the network and subsequent comparative analysis were supported by a certain amount of literature in terms of therapeutic effects and mechanisms of action. Nearly a quarter of all compounds in the formula were also validated in the literature. Moreover, selected upregulated compounds had a higher percentage of literature validation than unselected upregulated compounds.

#### 3.4.2. Molecular Docking Reveals the Relationship of Several Classes of Compounds

**Figure 12:**
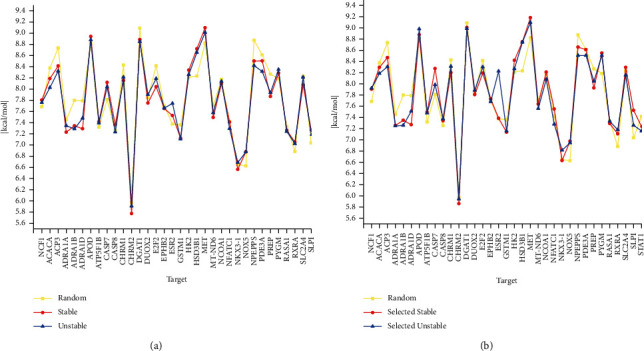
Results of molecular docking. (a) Mean results of molecular docking for random compounds, stable angina upregulated compounds, and unstable angina upregulated compounds. (b) Mean results for molecular docking of random compounds, selected upregulated compounds for stable angina, and selected upregulated compounds for unstable angina. The value of the vertical coordinate is the absolute value of the binding energy. The horizontal coordinates indicate unique targets.

We performed molecular docking of upregulated compounds, downregulated compounds, selected upregulated compounds, selected downregulated compounds, and random compounds with unique targets for the two classes of angina. Detailed values of the docking results are shown in Table S14 in the supplementary materials, and folded line graphs are shown in Figures 12(a) and 12(b).

The IDs and names of the upregulated compounds and selected upregulated compounds are listed in Tables S12 and S13 in the supplementary materials. Alternatively, the randomly selected compounds can be found in Table S15 in the supplementary materials. The target section presented in Table 3 shows 34 unique targets for stable angina and 1 unique target for unstable angina. These unique targets are the respective difference sets of these two angina targets after removing the intersection. Detailed information on the Gene Symbols of the unique targets and the molecular docking results is also included in Table S14 in the supplementary materials. For example, the unique targets for stable angina include CASP7 and SLC2A4, while the target for unstable angina is NCF1.

Specifically, a total of 114 up- or downregulated compounds, 49 selected up- or downregulated compounds, and 50 randomly selected compounds were molecularly docked to 35 unique targets in stable angina or unstable angina. In terms of numbers, we performed a total of 3990 molecular docking exercises. Each unique target was studied, and the mean value of the binding capacity of all compounds docked to that target was used as a criterion for determination. For unique targets in stable angina, a target was labelled if the ranking of binding capacity was random compounds <SA downregulated compounds <SA upregulated compounds. On the other hand, for unique targets in unstable angina, if the ranking of the binding capacity was random compounds < UA downregulated compounds < UA upregulated compounds, then the target was also labelled. To further illustrate the feasibility of the reverse process and the importance of the screening process, two additional determination criteria were added to that mentioned above. For stable unique targets, if the ranking of the binding capacity was random compounds <selected SA downregulated compounds <selected SA upregulated compounds or unselected upregulated compounds <selected upregulated compounds, then the target was labelled. In addition, for unstable unique targets, if the ranking of the binding capacity was random compounds < selected UA downregulated compounds < selected UA upregulated compounds or unselected upregulated compounds < selected upregulated compounds, then the target was labelled.

In total, 34% of unique targets proved to bind better to upregulated compounds than to downregulated compounds and random compounds, and the downregulated compounds bound better than random compounds, as shown in the results in Figure 12(a). The screening conditions were increased for both the upregulated and downregulated compounds, and then, the results of molecular docking were further analyzed, as shown in Figure 12(b). We found that 60% of the targets had a binding profile in which the selected upregulated compounds outperformed the selected downregulated compounds and the random compounds or the selected upregulated compounds outperformed the unselected upregulated compounds. Excluding random compounds and comparing the results of upregulated compounds only with those of downregulated compounds, we found that 58.82% of unique targets in stable angina resulted in better binding of upregulated compounds than downregulated compounds for docking. Increasing the screening conditions resulted in 61.76% of unique targets being better bound for the selected upregulated compounds than for the selected downregulated compounds. Also, after screening conditions were raised for compounds upregulated in unstable angina, the selected upregulated compounds also bind more strongly to unique targets in unstable angina than the unselected upregulated compounds.

Such results could indicate at the molecular docking level that the upregulated compounds bind more strongly to unique targets than the downregulated compounds, and in addition, increasing the screening conditions may help find more strongly bound upregulated compounds. Our reverse process of searching for the substance bases of the two classes of angina and, in particular, the substances ranked significantly upregulated in importance was validated by the molecular docking results.

**Figure 13:**
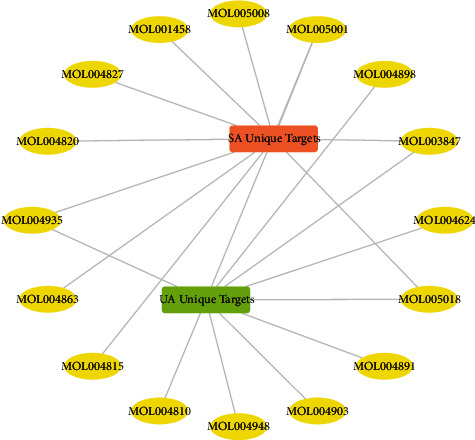
Compounds with high binding capacity to all the unique targets of both types of angina. Only the top 10 compounds for each of the two types of angina are shown. The thickness of the connecting line is proportional to the absolute value of the binding energy. The thicker the line, the stronger the binding capacity.

Of the two classes of upregulated compounds for angina, those with high binding capacity for all unique targets are shown in Figure 13, calculated by summing the binding capacity values for all unique targets and taking the mean value. The mean values of the binding energies of these compounds are all less than −8.3 kcal/mol.

#### 3.4.3. Indirect Evidence Was Obtained Using miRNA Enrichment Analysis

**Figure 14:**
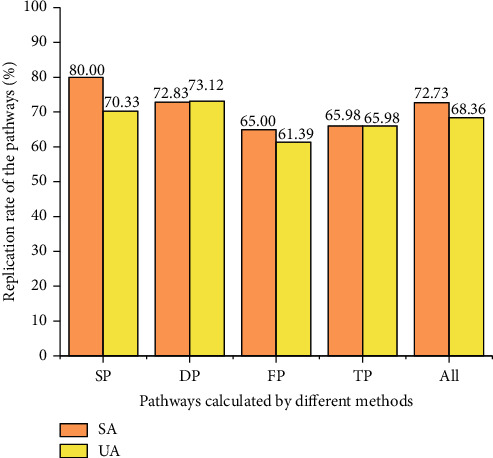
S, D, F, and T represent static, dynamic, enriched, and total terms, respectively. P represents pathways. All represents all pathways obtained by the forward process. SA and UA represent stable angina and unstable angina, respectively.

We first analyzed the dataset of miRNAs for stable angina and unstable angina in the GEO database to obtain data on the differential expression of miRNAs for the two types of angina. The data for the differential expression analysis were obtained by comparing miRNA expression data from angina patients with miRNA expression data from healthy controls using the GEO2R analysis tool. We next collected reliable targets of significantly up- or downregulated miRNAs, i.e., which genes are regulated by the miRNAs. Finally, the obtained target genes were subjected to KEGG enrichment analysis, and the results of significant enrichment were compared with those obtained by the various methods described previously. The replication of the enrichment pathways is shown in Figure 14. The specific miRNAs and the enriched pathways are shown in Tables S16 and S17 in the supplementary materials.

As can be seen in Figure 14, the calculated duplication of the unique pathways for both types of angina with the pathways enriched for miRNA function was above 60%, with some results as high as 80%, whether for dynamic, static, enriched, or total terms. The duplication rates of all pathways obtained from the C-T-P forward process with miRNA-enriched pathways were also higher than 70%. Since miRNAs are the main representatives of gene expression regulation [53], the abovementioned results indicate that both gene expression and gene expression regulation are concentrated in certain important pathways. The forward and reverse processes of our complex network were indirectly validated by the functional enrichment of miRNAs.

## 4. Discussion

Numerous clinical studies have demonstrated the effectiveness of Xuefu Zhuyu decoction in the treatment of coronary heart disease or in the treatment of stable and unstable angina pectoris in coronary heart disease [54, 55]. However, from the perspective of complex networks, few studies have been conducted to investigate the efficacy of Xuefu Zhuyu decoction and its comparison with classical Western drugs. In this study, we first applied a network proximity approach to demonstrate that the targets of the formula are closer to the disease targets than those of approved Western drugs, for both stable and unstable angina pectoris. We then take the intersection of the targets of the drugs and the targets of the disease and then use the method shown in equation (1) to perform the calculations again. The results show that, after taking the intersection, the targets of action of the formulation is obtained closer to the targets of the disease. The results of the network proximity after taking the intersection also demonstrate that the formulation is closer to both diseases than the Western drugs in terms of target network. Therefore, in terms of network proximity of target networks, it can be demonstrated that Xuefu Zhuyu decoction is superior to the approved classical Western drugs and, therefore, may have better therapeutic effects. On the other hand, the results of the intersecting targets are even better, so for this study and a large number of previous studies, it is feasible to take the intersection for follow-up studies.

Network pharmacology can be used to study the biological basis of prescriptions and diseases by constructing complex networks [14]. In the present study, we used the network pharmacology approach to construct C-T, T-T, and T-P networks, which together form a complex heterogeneous network. Within these two complex heterogeneous networks for both angina pectoris, we compared them at three levels and in five dimensions, i.e., mechanisms of action. In particular, on the target-function side, we compared the target networks in terms of network clustering and functional similarity, and a heat map with hierarchical clustering was drawn. The results of the comparisons show a large proportion of common compounds, targets, and functions and a smaller proportion of unique components. However, even within the common fraction, the importance of each target and the importance of each pathway still varies.

In fact, in terms of the common fraction, targets and pathways can correspond to each other. For example, the first ranked classification in the common pathways is signal transduction, which corresponds directly to the fifth ranked classification in the common targets, signaling. Of course, signal transduction is also closely related to enzymes, kinases, and transcription factors [56, 57]. It is also true that the onset and development of angina is closely linked to signal transduction, enzymes, and kinases [58, 59], and the literature that this formula acts on these types of targets has been documented in the literature validation section. The use of network clustering to divide target networks into modules for subsequent studies is a current hot topic in network pharmacology and drug design research [60]. However, the decision to use GO or KEGG for functional studies of modules is not conclusive. We calculated and clustered both GO similarity and KEGG similarity between the two angina modules and showed that the modules for stable angina and unstable angina always clustered together at the bottom of the clustering tree and that the correlation coefficients showed that the two functional similarities were highly correlated. The functions generated by the targets were well represented by either GO or KEGG. The results of the modular analysis also illustrate the mechanism of action of the two angina pectoris, i.e., functional enrichment, with most of the similarities and a small number of differences.

In terms of mining the material basis of the formula, we applied all three scoring methods, random wandering with restart, calculation of degree values with focus genes, and KEGG enrichment, to the reverse process of design. Together with a total term combining the three scoring features described above, four sets of pathways specific to each angina were obtained as well as up- and downregulated compounds. The results showed that the duplication rate for the four sets of specific pathways was low while the duplication rate for the upregulated compounds was high. This suggests that certain important compounds can be found regardless of which scoring method is applied for reverse lookup; i.e., certain upregulated compounds play many important roles in the complex network. The results after taking duplicate values for the four unique pathway groups suggest that, for both angina pectoris, many signaling pathways play an important role [61, 62]. We have performed additional screening among the upregulated compounds, and very valuable ones that bind well to all the unique targets are shown in the molecular docking section.

In the results validation section, three methods were applied to verify the reliability of the material basis and mechanism of action obtained. The literature mining focused on the mechanism of action part, specifically whether all compounds and upregulated compounds had a therapeutic effect (relationship to the disease) and how they acted (relationship to the target). The literature validation rate is broadly comparable to previous studies [32], with some of our literature validation results exceeding 25% and even exceeding 30%, which is relatively high. Molecular docking focused on the material basis part, and two classes of angina up- and downregulated compounds obtained by reverse lookup as well as randomly selected random compounds were used for molecular docking to unique targets. The results of nearly four thousand molecular dockings showed that, after further screening, the selected upregulated compounds bind better than the selected downregulated compounds and that the selected upregulated compounds also bind better than the random compounds. This suggests that our process of reverse compound finding and the reselection of upregulated compounds based on importance ranking is relatively reliable. The compounds with very good binding capacity are shown in Figure 13. Among the compounds that bind particularly well to the targets of stable angina, literature mining showed that coptisine (MOL001458) lowered blood pressure and improved endothelial function [63], while Sigmoidin-B (MOL004935) had an anti-inflammatory activity and antioxidant properties [64]. Also, Kanzonol B (MOL004815) could regulate the expression of inducible nitric oxide synthase (iNOS) and cyclooxygenase-2 (COX-2) in microglia [65]. Among the particularly excellent compounds for unstable angina, liquiritin (MOL004903) reduces mRNA expression of TNF*α*, IL-6, and IL-1*β*, inhibits inflammatory cell migration, suppresses oxidative stress and apoptosis in the heart, and improves metabolism [66]. Sigmoidin-B (MOL004935), which has anti-inflammatory effects, is also present here in unstable angina. This suggests that these excellent compounds have been reported to have potential therapeutic effects in angina pectoris.

The miRNA functional enrichment analysis focused on the functional aspects, i.e., the source of the reverse process. The results show that miRNAs also always regulate those functions for which enrichment has been obtained. Certain functions of stable angina include the calcium signaling pathway (hsa04020), leukocyte transendothelial migration (hsa04670), and NF-kappa B signaling pathway (hsa04064) in miRNA functions as well as in dynamic, static, enriched, and total terms. One of the antiangina therapeutic agents is calcium channel blockers [67]. Inhibition of NF-*κ*B signaling and reduction of NF-*κ*B levels by certain herbal ingredients can enhance the antioxidant and anti-inflammatory capacity of patients with coronary artery disease and improve their symptoms of stable angina [68, 69]. In addition, leukocytes play an active role in the progression of coronary artery disease, and leukocyte immobilization is significantly different in patients with coronary artery disease than in healthy subjects [70]. In unstable angina, many of the unique pathways are the same as in stable angina, but the importance of the pathways is different. In addition, five unique pathways exclusive to unstable angina (mentioned in Table 3) are included in this result, see Figures 10(a) and 10(b). For example, for insulin secretion (hsa04911), high plasma phospholipid transfer protein (PLTP) is associated with type II diabetes and obesity, while PLTP mediates the net transfer and exchange of phospholipids between different lipoproteins and is strongly associated with the development of atherosclerosis and, thus, angina [71]. In conclusion, the functions of miRNAs validate the results of the pathways obtained from the four scores in terms of gene expression regulation, and certain important functions are always present in their results.

## 5. Conclusions

In summary, our study led to the following conclusions: firstly, for both angina pectoris types, Xuefu Zhuyu decoction produced better therapeutic effects than approved Western drugs in terms of network topology. Moreover, we demonstrated that the study of intersecting targets is more meaningful from the perspective of the topology of the target network. Secondly, there is significant overlap between the mechanisms of action of the two types of angina, but the unique functions of each type of angina can be found using either conventional methods or modular analysis. In addition, the GO similarity of the target modules of both angina pectoris is highly correlated with the KEGG similarity, making both GO and KEGG meaningful studies. Finally, the upregulated compounds, i.e., the material basis, of each angina could be found using the designed inverse process, and four validation methods including duplication rate, literature mining, molecular docking, and miRNA functional enrichment all supported our results.

## Figures and Tables

**Table 1:** Target profiles of the formula, Western drugs, and diseases and their interactions.

	Xuefu Zhuyu decoction				Approved Western drugs				Disease targets
	Compounds	Targets	Compounds-targets	Compounds-disease targets	Drugs	Targets	Drugs-targets	Drugs-disease targets	
Stable angina	125	230	1900	1677	68	398	898	593	3321
Unstable angina	125	230	1900	1461	22	221	338	161	1626

**Table 2:** Comparison of network proximity of formula targets, disease targets, approved drug targets, and intersection targets.

		Average shortest distance	Formula-disease	Drug-disease	Intersecting targets-disease
Stable angina	Formula	1.9967	0.0423		0.0374
	Disease	2.2119			
	Drug	2.1465		0.1541	0.0886

Unstable angina	Formula	2.0166	0.0492		0.0179
	Disease	2.2010			
	Drug	2.1580		0.2213	0.0919

**Table 3:** Results of the comparison of compounds, targets, and functional enrichment.

Level	Stable angina	Unstable angina	Common parts	Different parts	
				Stable angina	Unstable angina
Compound	125	124	124	1	0
Target	192	159	158	34	1
GO (BP)	2382	2353	2236	146	117
KEGG	176	177	172	4	5

**Table 4:** Correlation between the two functional similarities.

	Comparison of two kinds of functional similarity			
	clust1	clust2	clust3	clust4
Spearman's rank correlation rho	1	0.8	1	1
*p* value	<2.2*e* − 16	0.2	<2.2*e* − 16	<2.2*e* − 16

## Data Availability

All data used to support the results of this study can be found in the article or in the supplementary materials.

## Supplementary Materials

Supplementary Materials The supplementary materials consist of 8 parts, with a total of 17 tables and 2 figures. Table S1: herbs and compounds in the formula. Table S2: targets of compound action. Table S3: targets for stable versus unstable angina pectoris. Table S4 and Table S5: approved Western drugs and their targets. Table S6 and Table S7: the literature obtained by literature mining. Table S8: the modularity index of network clustering. Table S9, Table S10, and Table S11: scores for each pathway and replicated pathways. Table S12 and Table S13: upregulated compounds and selected upregulated compounds. Table S14 and Table S15: results of molecular docking and random compounds. Table S16: results of differentially expressed miRNAs. Table S17: results of miRNA functional enrichment. Figure S1 and Figure S2: the score propensity of the pathways.
