# Supplementary material for: The Material Basis and Mechanism of Xuefu Zhuyu Decoction in Treating Stable Angina Pectoris and Unstable Angina Pectoris
Source: Evid Based Complement Alternat Med. 2022 Jan 31;2022:3741027. doi: 10.1155/2022/3741027 (PMC8820872; doi:10.1155/2022/3741027)
Supplement: Supplementary Materials — The supplementary materials consist of 8 parts, with a total of 17 tables and 2 figures. Table S1: herbs and compounds in the formula. Table S2: targets of compound action. Table S3: targets for stable versus unstable angina pectoris. Table S4 and Table S5: approved Western drugs and their targets. Table S6 and Table S7: the literature obtained by literature mining. Table S8: the modularity index of network clustering. Table S9, Table S10, and Table S11: scores for each pathway and replicated pathways. Table S12 and Table S13: upregulated compounds and selected upregulated compounds. Table S14 and Table S15: results of molecular docking and random compounds. Table S16: results of differentially expressed miRNAs. Table S17: results of miRNA functional enrichment. Figure S1 and Figure S2: the score propensity of the pathways. [file 3741027.f1.docx]

**The supplementary document consists of 8 parts, with a total of 17 tables and 2 figures.**

Part 1: The herbs and compounds contained in the formula and the targets of action (Pages 1 to 55).

Part 2: Targets for stable versus unstable angina pectoris (Pages 55 to 92).

Part 3: Approved Western drugs and their targets (Pages 92 to 134).

Part 4: The literature obtained by literature mining (Pages 134 to 143).

Part 5: The modularity index of network clustering, the score propensity of the pathways, and the importance ranking of the pathways (Pages 143 to 153).

Part 6: Up-regulated compounds and selected up-regulated compounds (Pages 153 to 157).

Part 7: Results of molecular docking (Pages 157 to 159).

Part 8: Results of differentially expressed miRNAs and their functional enrichment (Pages 159 to 169).

**Part 1 contains 2 tables, including Table S1 and Table S2.**

**Table S1 Herbs and ingredients**

| Herb | English Name | Mol_ID | MoleculeName |
| --- | --- | --- | --- |
| 柴胡 | Bupleuri Radix | MOL000354 | isorhamnetin |
| 柴胡 | Bupleuri Radix | MOL000422 | kaempferol |
| 柴胡 | Bupleuri Radix | MOL004598 | 3,5,6,7-tetramethoxy-2-(3,4,5-trimethoxyphenyl)chromone |
| 柴胡 | Bupleuri Radix | MOL004609 | Areapillin |
| 柴胡 | Bupleuri Radix | MOL013187 | Cubebin |
| 柴胡 | Bupleuri Radix | MOL004624 | Longikaurin A |
| 柴胡 | Bupleuri Radix | MOL004628 | Octalupine |
| 柴胡 | Bupleuri Radix | MOL004644 | Sainfuran |
| 柴胡 | Bupleuri Radix | MOL004702 | saikosaponin c_qt |
| 柴胡 | Bupleuri Radix | MOL000490 | petunidin |
| 柴胡 | Bupleuri Radix | MOL000098 | quercetin |
| 赤芍 | Paeoniae Radix Rubra | MOL001002 | ellagic acid |
| 赤芍 | Paeoniae Radix Rubra | MOL001918 | paeoniflorgenone |
| 赤芍 | Paeoniae Radix Rubra | MOL001921 | Lactiflorin |
| 赤芍 | Paeoniae Radix Rubra | MOL001925 | paeoniflorin_qt |
| 赤芍 | Paeoniae Radix Rubra | MOL002714 | baicalein |
| 赤芍 | Paeoniae Radix Rubra | MOL000492 | (+)-catechin |
| 赤芍 | Paeoniae Radix Rubra | MOL006990 | (1S,2S,4R)-trans-2-hydroxy-1,8-cineole-B-D-glucopyranoside |
| 赤芍 | Paeoniae Radix Rubra | MOL006992 | (2R,3R)-4-methoxyl-distylin |
| 赤芍 | Paeoniae Radix Rubra | MOL006994 | 1-o-beta-d-glucopyranosyl-8-o-benzoylpaeonisuffrone_qt |
| 赤芍 | Paeoniae Radix Rubra | MOL006996 | 1-o-beta-d-glucopyranosylpaeonisuffrone_qt |
| 赤芍 | Paeoniae Radix Rubra | MOL007005 | Albiflorin_qt |
| 赤芍 | Paeoniae Radix Rubra | MOL007008 | 4-ethyl-paeoniflorin_qt |
| 赤芍 | Paeoniae Radix Rubra | MOL007012 | 4-o-methyl-paeoniflorin_qt |
| 赤芍 | Paeoniae Radix Rubra | MOL007016 | Paeoniflorigenone |
| 赤芍 | Paeoniae Radix Rubra | MOL007018 | 9-ethyl-neo-paeoniaflorin A_qt |
| 赤芍 | Paeoniae Radix Rubra | MOL007022 | evofolinB |
| 川芎 | Chuanxiong Rhizoma | MOL002135 | Myricanone |
| 川芎 | Chuanxiong Rhizoma | MOL002140 | Perlolyrine |
| 川芎 | Chuanxiong Rhizoma | MOL002157 | wallichilide |
| 甘草 | Glycyrrhizae Radix Et Rhizoma | MOL000354 | isorhamnetin |
| 甘草 | Glycyrrhizae Radix Et Rhizoma | MOL000422 | kaempferol |
| 甘草 | Glycyrrhizae Radix Et Rhizoma | MOL000098 | quercetin |
| 甘草 | Glycyrrhizae Radix Et Rhizoma | MOL001484 | Inermine |
| 甘草 | Glycyrrhizae Radix Et Rhizoma | MOL001792 | DFV |
| 甘草 | Glycyrrhizae Radix Et Rhizoma | MOL002311 | Glycyrol |
| 甘草 | Glycyrrhizae Radix Et Rhizoma | MOL000239 | Jaranol |
| 甘草 | Glycyrrhizae Radix Et Rhizoma | MOL002565 | Medicarpin |
| 甘草 | Glycyrrhizae Radix Et Rhizoma | MOL003656 | Lupiwighteone |
| 甘草 | Glycyrrhizae Radix Et Rhizoma | MOL003896 | 7-Methoxy-2-methyl isoflavone |
| 甘草 | Glycyrrhizae Radix Et Rhizoma | MOL000392 | formononetin |
| 甘草 | Glycyrrhizae Radix Et Rhizoma | MOL000417 | Calycosin |
| 甘草 | Glycyrrhizae Radix Et Rhizoma | MOL004328 | naringenin |
| 甘草 | Glycyrrhizae Radix Et Rhizoma | MOL004808 | glyasperin B |
| 甘草 | Glycyrrhizae Radix Et Rhizoma | MOL004810 | glyasperin F |
| 甘草 | Glycyrrhizae Radix Et Rhizoma | MOL004811 | Glyasperin C |
| 甘草 | Glycyrrhizae Radix Et Rhizoma | MOL004814 | Isotrifoliol |
| 甘草 | Glycyrrhizae Radix Et Rhizoma | MOL004815 | (E)-1-(2,4-dihydroxyphenyl)-3-(2,2-dimethylchromen-6-yl)prop-2-en-1-one |
| 甘草 | Glycyrrhizae Radix Et Rhizoma | MOL004820 | kanzonols W |
| 甘草 | Glycyrrhizae Radix Et Rhizoma | MOL004824 | (2S)-6-(2,4-dihydroxyphenyl)-2-(2-hydroxypropan-2-yl)-4-methoxy-2,3-dihydrofuro[3,2-g]chromen-7-one |
| 甘草 | Glycyrrhizae Radix Et Rhizoma | MOL004827 | Semilicoisoflavone B |
| 甘草 | Glycyrrhizae Radix Et Rhizoma | MOL004828 | Glepidotin A |
| 甘草 | Glycyrrhizae Radix Et Rhizoma | MOL004829 | Glepidotin B |
| 甘草 | Glycyrrhizae Radix Et Rhizoma | MOL004833 | Phaseolinisoflavan |
| 甘草 | Glycyrrhizae Radix Et Rhizoma | MOL004835 | Glypallichalcone |
| 甘草 | Glycyrrhizae Radix Et Rhizoma | MOL004838 | 8-(6-hydroxy-2-benzofuranyl)-2,2-dimethyl-5-chromenol |
| 甘草 | Glycyrrhizae Radix Et Rhizoma | MOL004841 | Licochalcone B |
| 甘草 | Glycyrrhizae Radix Et Rhizoma | MOL004848 | licochalcone G |
| 甘草 | Glycyrrhizae Radix Et Rhizoma | MOL004849 | 3-(2,4-dihydroxyphenyl)-8-(1,1-dimethylprop-2-enyl)-7-hydroxy-5-methoxy-coumarin |
| 甘草 | Glycyrrhizae Radix Et Rhizoma | MOL004855 | Licoricone |
| 甘草 | Glycyrrhizae Radix Et Rhizoma | MOL004856 | Gancaonin A |
| 甘草 | Glycyrrhizae Radix Et Rhizoma | MOL004857 | Gancaonin B |
| 甘草 | Glycyrrhizae Radix Et Rhizoma | MOL004863 | 3-(3,4-dihydroxyphenyl)-5,7-dihydroxy-8-(3-methylbut-2-enyl)chromone |
| 甘草 | Glycyrrhizae Radix Et Rhizoma | MOL004864 | 5,7-dihydroxy-3-(4-methoxyphenyl)-8-(3-methylbut-2-enyl)chromone |
| 甘草 | Glycyrrhizae Radix Et Rhizoma | MOL004866 | 2-(3,4-dihydroxyphenyl)-5,7-dihydroxy-6-(3-methylbut-2-enyl)chromone |
| 甘草 | Glycyrrhizae Radix Et Rhizoma | MOL004879 | Glycyrin |
| 甘草 | Glycyrrhizae Radix Et Rhizoma | MOL004882 | Licocoumarone |
| 甘草 | Glycyrrhizae Radix Et Rhizoma | MOL004883 | Licoisoflavone |
| 甘草 | Glycyrrhizae Radix Et Rhizoma | MOL004884 | Licoisoflavone B |
| 甘草 | Glycyrrhizae Radix Et Rhizoma | MOL004885 | licoisoflavanone |
| 甘草 | Glycyrrhizae Radix Et Rhizoma | MOL004891 | shinpterocarpin |
| 甘草 | Glycyrrhizae Radix Et Rhizoma | MOL004898 | (E)-3-[3,4-dihydroxy-5-(3-methylbut-2-enyl)phenyl]-1-(2,4-dihydroxyphenyl)prop-2-en-1-one |
| 甘草 | Glycyrrhizae Radix Et Rhizoma | MOL004903 | liquiritin |
| 甘草 | Glycyrrhizae Radix Et Rhizoma | MOL004904 | licopyranocoumarin |
| 甘草 | Glycyrrhizae Radix Et Rhizoma | MOL004907 | Glyzaglabrin |
| 甘草 | Glycyrrhizae Radix Et Rhizoma | MOL004908 | Glabridin |
| 甘草 | Glycyrrhizae Radix Et Rhizoma | MOL004910 | Glabranin |
| 甘草 | Glycyrrhizae Radix Et Rhizoma | MOL004911 | Glabrene |
| 甘草 | Glycyrrhizae Radix Et Rhizoma | MOL004912 | Glabrone |
| 甘草 | Glycyrrhizae Radix Et Rhizoma | MOL004913 | 1,3-dihydroxy-9-methoxy-6-benzofurano[3,2-c]chromenone |
| 甘草 | Glycyrrhizae Radix Et Rhizoma | MOL004914 | 1,3-dihydroxy-8,9-dimethoxy-6-benzofurano[3,2-c]chromenone |
| 甘草 | Glycyrrhizae Radix Et Rhizoma | MOL004915 | Eurycarpin A |
| 甘草 | Glycyrrhizae Radix Et Rhizoma | MOL004924 | (-)-Medicocarpin |
| 甘草 | Glycyrrhizae Radix Et Rhizoma | MOL004935 | Sigmoidin-B |
| 甘草 | Glycyrrhizae Radix Et Rhizoma | MOL004941 | (2R)-7-hydroxy-2-(4-hydroxyphenyl)chroman-4-one |
| 甘草 | Glycyrrhizae Radix Et Rhizoma | MOL004945 | (2S)-7-hydroxy-2-(4-hydroxyphenyl)-8-(3-methylbut-2-enyl)chroman-4-one |
| 甘草 | Glycyrrhizae Radix Et Rhizoma | MOL004948 | Isoglycyrol |
| 甘草 | Glycyrrhizae Radix Et Rhizoma | MOL004949 | Isolicoflavonol |
| 甘草 | Glycyrrhizae Radix Et Rhizoma | MOL004957 | HMO |
| 甘草 | Glycyrrhizae Radix Et Rhizoma | MOL004959 | 1-Methoxyphaseollidin |
| 甘草 | Glycyrrhizae Radix Et Rhizoma | MOL004961 | Quercetin der. |
| 甘草 | Glycyrrhizae Radix Et Rhizoma | MOL004966 | 3'-Hydroxy-4'-O-Methylglabridin |
| 甘草 | Glycyrrhizae Radix Et Rhizoma | MOL000497 | licochalcone a |
| 甘草 | Glycyrrhizae Radix Et Rhizoma | MOL004974 | 3'-Methoxyglabridin |
| 甘草 | Glycyrrhizae Radix Et Rhizoma | MOL004978 | 2-[(3R)-8,8-dimethyl-3,4-dihydro-2H-pyrano[6,5-f]chromen-3-yl]-5-methoxyphenol |
| 甘草 | Glycyrrhizae Radix Et Rhizoma | MOL004980 | Inflacoumarin A |
| 甘草 | Glycyrrhizae Radix Et Rhizoma | MOL004989 | 6-prenylated eriodictyol |
| 甘草 | Glycyrrhizae Radix Et Rhizoma | MOL004990 | 7,2',4'-trihydroxy－5-methoxy-3－arylcoumarin |
| 甘草 | Glycyrrhizae Radix Et Rhizoma | MOL004991 | 7-Acetoxy-2-methylisoflavone |
| 甘草 | Glycyrrhizae Radix Et Rhizoma | MOL004993 | 8-prenylated eriodictyol |
| 甘草 | Glycyrrhizae Radix Et Rhizoma | MOL000500 | Vestitol |
| 甘草 | Glycyrrhizae Radix Et Rhizoma | MOL005000 | Gancaonin G |
| 甘草 | Glycyrrhizae Radix Et Rhizoma | MOL005001 | Gancaonin H |
| 甘草 | Glycyrrhizae Radix Et Rhizoma | MOL005003 | Licoagrocarpin |
| 甘草 | Glycyrrhizae Radix Et Rhizoma | MOL005007 | Glyasperins M |
| 甘草 | Glycyrrhizae Radix Et Rhizoma | MOL005008 | Glycyrrhiza flavonol A |
| 甘草 | Glycyrrhizae Radix Et Rhizoma | MOL005012 | Licoagroisoflavone |
| 甘草 | Glycyrrhizae Radix Et Rhizoma | MOL005013 | 18α-hydroxyglycyrrhetic acid |
| 甘草 | Glycyrrhizae Radix Et Rhizoma | MOL005016 | Odoratin |
| 甘草 | Glycyrrhizae Radix Et Rhizoma | MOL005017 | Phaseol |
| 甘草 | Glycyrrhizae Radix Et Rhizoma | MOL005018 | Xambioona |
| 甘草 | Glycyrrhizae Radix Et Rhizoma | MOL005020 | dehydroglyasperins C |
| 红花 | Carthami Flos | MOL000422 | kaempferol |
| 红花 | Carthami Flos | MOL000098 | quercetin |
| 红花 | Carthami Flos | MOL002714 | baicalein |
| 红花 | Carthami Flos | MOL000006 | luteolin |
| 红花 | Carthami Flos | MOL002694 | 4-[(E)-4-(3,5-dimethoxy-4-oxo-1-cyclohexa-2,5-dienylidene)but-2-enylidene]-2,6-dimethoxycyclohexa-2,5-dien-1-one |
| 红花 | Carthami Flos | MOL002695 | lignan |
| 红花 | Carthami Flos | MOL002710 | Pyrethrin II |
| 红花 | Carthami Flos | MOL002712 | 6-Hydroxykaempferol |
| 红花 | Carthami Flos | MOL002717 | qt_carthamone |
| 红花 | Carthami Flos | MOL002719 | 6-Hydroxynaringenin |
| 红花 | Carthami Flos | MOL002757 | 7,8-dimethyl-1H-pyrimido[5,6-g]quinoxaline-2,4-dione |
| 桔梗 | Platycodonis Radix | MOL001689 | acacetin |
| 桔梗 | Platycodonis Radix | MOL004580 | cis-Dihydroquercetin |
| 桔梗 | Platycodonis Radix | MOL000006 | luteolin |
| 牛膝 | Achyranthis Bidentatae Radix | MOL000422 | kaempferol |
| 牛膝 | Achyranthis Bidentatae Radix | MOL000098 | quercetin |
| 牛膝 | Achyranthis Bidentatae Radix | MOL002714 | baicalein |
| 牛膝 | Achyranthis Bidentatae Radix | MOL001454 | berberine |
| 牛膝 | Achyranthis Bidentatae Radix | MOL001458 | coptisine |
| 牛膝 | Achyranthis Bidentatae Radix | MOL000173 | wogonin |
| 牛膝 | Achyranthis Bidentatae Radix | MOL002897 | epiberberine |
| 牛膝 | Achyranthis Bidentatae Radix | MOL003847 | Inophyllum E |
| 牛膝 | Achyranthis Bidentatae Radix | MOL000785 | palmatine |
| 桃仁 | Persicae Semen | MOL001328 | 2,3-didehydro GA70 |
| 桃仁 | Persicae Semen | MOL001329 | 2,3-didehydro GA77 |
| 桃仁 | Persicae Semen | MOL001339 | GA119 |
| 桃仁 | Persicae Semen | MOL001340 | GA120 |
| 桃仁 | Persicae Semen | MOL001342 | GA121-isolactone |
| 桃仁 | Persicae Semen | MOL001343 | GA122 |
| 桃仁 | Persicae Semen | MOL001344 | GA122-isolactone |
| 桃仁 | Persicae Semen | MOL001348 | gibberellin 17 |
| 桃仁 | Persicae Semen | MOL001349 | 4a-formyl-7alpha-hydroxy-1-methyl-8-methylidene-4aalpha,4bbeta-gibbane-1alpha,10beta-dicarboxylic acid |
| 桃仁 | Persicae Semen | MOL001350 | GA30 |
| 桃仁 | Persicae Semen | MOL001351 | Gibberellin A44 |
| 桃仁 | Persicae Semen | MOL001352 | GA54 |
| 桃仁 | Persicae Semen | MOL001353 | GA60 |
| 桃仁 | Persicae Semen | MOL001355 | GA63 |
| 桃仁 | Persicae Semen | MOL001358 | gibberellin 7 |
| 桃仁 | Persicae Semen | MOL001360 | GA77 |
| 桃仁 | Persicae Semen | MOL001361 | GA87 |
| 桃仁 | Persicae Semen | MOL001368 | 3-O-p-coumaroylquinic acid |
| 桃仁 | Persicae Semen | MOL001371 | Populoside_qt |
| 枳壳 | Aurantii Fructus | MOL004328 | naringenin |
| 枳壳 | Aurantii Fructus | MOL013381 | Marmin |
| 枳壳 | Aurantii Fructus | MOL002341 | Hesperetin |
| 枳壳 | Aurantii Fructus | MOL005828 | nobiletin |

**Table S2 Ingredients and Targets**

| Mol_ID | Molecule_Name | gene |
| --- | --- | --- |
| MOL000006 | luteolin | ADCY2 |
| MOL000006 | luteolin | AKT1 |
| MOL000006 | luteolin | AR |
| MOL000006 | luteolin | BCL2L1 |
| MOL000006 | luteolin | BIRC5 |
| MOL000006 | luteolin | CASP3 |
| MOL000006 | luteolin | CASP7 |
| MOL000006 | luteolin | CASP9 |
| MOL000006 | luteolin | CCNB1 |
| MOL000006 | luteolin | CCND1 |
| MOL000006 | luteolin | CD40LG |
| MOL000006 | luteolin | CDKN1A |
| MOL000006 | luteolin | DPP4 |
| MOL000006 | luteolin | EGFR |
| MOL000006 | luteolin | ERBB2 |
| MOL000006 | luteolin | HMOX1 |
| MOL000006 | luteolin | HSP90AA |
| MOL000006 | luteolin | ICAM1 |
| MOL000006 | luteolin | IFNG |
| MOL000006 | luteolin | IL10 |
| MOL000006 | luteolin | IL2 |
| MOL000006 | luteolin | IL4 |
| MOL000006 | luteolin | IL6 |
| MOL000006 | luteolin | INSR |
| MOL000006 | luteolin | JUN |
| MOL000006 | luteolin | MAPK1 |
| MOL000006 | luteolin | MCL1 |
| MOL000006 | luteolin | MDM2 |
| MOL000006 | luteolin | MET |
| MOL000006 | luteolin | MMP1 |
| MOL000006 | luteolin | MMP2 |
| MOL000006 | luteolin | MMP9 |
| MOL000006 | luteolin | NCOA2 |
| MOL000006 | luteolin | NFKBIA |
| MOL000006 | luteolin | NUF2 |
| MOL000006 | luteolin | PCNA |
| MOL000006 | luteolin | PPARG |
| MOL000006 | luteolin | PRKACA |
| MOL000006 | luteolin | PRSS1 |
| MOL000006 | luteolin | PTGES |
| MOL000006 | luteolin | PTGS1 |
| MOL000006 | luteolin | PTGS2 |
| MOL000006 | luteolin | RB1 |
| MOL000006 | luteolin | RELA |
| MOL000006 | luteolin | SLC2A4 |
| MOL000006 | luteolin | TNF |
| MOL000006 | luteolin | TOP1 |
| MOL000006 | luteolin | TOP2A |
| MOL000006 | luteolin | TP53 |
| MOL000006 | luteolin | TYR |
| MOL000006 | luteolin | UBE2O |
| MOL000098 | quercetin | ACACA |
| MOL000098 | quercetin | ACHE |
| MOL000098 | quercetin | ACP3 |
| MOL000098 | quercetin | ADRB2 |
| MOL000098 | quercetin | AHR |
| MOL000098 | quercetin | AHSA1 |
| MOL000098 | quercetin | AKT1 |
| MOL000098 | quercetin | AR |
| MOL000098 | quercetin | BAX |
| MOL000098 | quercetin | BCL2 |
| MOL000098 | quercetin | BCL2L1 |
| MOL000098 | quercetin | BIRC5 |
| MOL000098 | quercetin | CASP3 |
| MOL000098 | quercetin | CASP8 |
| MOL000098 | quercetin | CASP9 |
| MOL000098 | quercetin | CAV1 |
| MOL000098 | quercetin | CCNB1 |
| MOL000098 | quercetin | CCND1 |
| MOL000098 | quercetin | CD40LG |
| MOL000098 | quercetin | CDKN1A |
| MOL000098 | quercetin | CHEK2 |
| MOL000098 | quercetin | CHUK |
| MOL000098 | quercetin | CLDN4 |
| MOL000098 | quercetin | CTSD |
| MOL000098 | quercetin | CXCL10 |
| MOL000098 | quercetin | CXCL11 |
| MOL000098 | quercetin | CXCL2 |
| MOL000098 | quercetin | CXCL8 |
| MOL000098 | quercetin | CYP1A1 |
| MOL000098 | quercetin | CYP1B1 |
| MOL000098 | quercetin | CYP3A4 |
| MOL000098 | quercetin | DCAF5 |
| MOL000098 | quercetin | DIO1 |
| MOL000098 | quercetin | DPP4 |
| MOL000098 | quercetin | DUOX2 |
| MOL000098 | quercetin | E2F1 |
| MOL000098 | quercetin | E2F2 |
| MOL000098 | quercetin | EGFR |
| MOL000098 | quercetin | EIF6 |
| MOL000098 | quercetin | ELK1 |
| MOL000098 | quercetin | ERBB2 |
| MOL000098 | quercetin | ERBB3 |
| MOL000098 | quercetin | F10 |
| MOL000098 | quercetin | F2 |
| MOL000098 | quercetin | F3 |
| MOL000098 | quercetin | F7 |
| MOL000098 | quercetin | FOS |
| MOL000098 | quercetin | GJA1 |
| MOL000098 | quercetin | GSTM1 |
| MOL000098 | quercetin | GSTM2 |
| MOL000098 | quercetin | HAS2 |
| MOL000098 | quercetin | HIF1A |
| MOL000098 | quercetin | HK2 |
| MOL000098 | quercetin | HMOX1 |
| MOL000098 | quercetin | HSF1 |
| MOL000098 | quercetin | HSP90AA |
| MOL000098 | quercetin | HSPB1 |
| MOL000098 | quercetin | ICAM1 |
| MOL000098 | quercetin | IFNG |
| MOL000098 | quercetin | IGF2 |
| MOL000098 | quercetin | IGFBP3 |
| MOL000098 | quercetin | IL10 |
| MOL000098 | quercetin | IL1A |
| MOL000098 | quercetin | IL1B |
| MOL000098 | quercetin | IL2 |
| MOL000098 | quercetin | IL6 |
| MOL000098 | quercetin | INSR |
| MOL000098 | quercetin | IRF1 |
| MOL000098 | quercetin | JUN |
| MOL000098 | quercetin | KCNH2 |
| MOL000098 | quercetin | MAOB |
| MOL000098 | quercetin | MAPK1 |
| MOL000098 | quercetin | MMP1 |
| MOL000098 | quercetin | MMP2 |
| MOL000098 | quercetin | MMP3 |
| MOL000098 | quercetin | MMP9 |
| MOL000098 | quercetin | MPO |
| MOL000098 | quercetin | MYC |
| MOL000098 | quercetin | NCF1 |
| MOL000098 | quercetin | NCOA2 |
| MOL000098 | quercetin | NFE2L2 |
| MOL000098 | quercetin | NFKBIA |
| MOL000098 | quercetin | NKX3-1 |
| MOL000098 | quercetin | NOS3 |
| MOL000098 | quercetin | NPEPPS |
| MOL000098 | quercetin | NR1I2 |
| MOL000098 | quercetin | NR1I3 |
| MOL000098 | quercetin | ODC1 |
| MOL000098 | quercetin | PARP1 |
| MOL000098 | quercetin | PCOLCE |
| MOL000098 | quercetin | PLAT |
| MOL000098 | quercetin | PLAU |
| MOL000098 | quercetin | PON1 |
| MOL000098 | quercetin | PPARA |
| MOL000098 | quercetin | PPARD |
| MOL000098 | quercetin | PPARG |
| MOL000098 | quercetin | PRKACA |
| MOL000098 | quercetin | PRKCA |
| MOL000098 | quercetin | PRKCB |
| MOL000098 | quercetin | PRSS1 |
| MOL000098 | quercetin | PSMD3 |
| MOL000098 | quercetin | PTGS1 |
| MOL000098 | quercetin | PTGS2 |
| MOL000098 | quercetin | RAF1 |
| MOL000098 | quercetin | RASA1 |
| MOL000098 | quercetin | RASSF1 |
| MOL000098 | quercetin | RB1 |
| MOL000098 | quercetin | RELA |
| MOL000098 | quercetin | RUNX1T1 |
| MOL000098 | quercetin | RUNX2 |
| MOL000098 | quercetin | RXRA |
| MOL000098 | quercetin | SCN5A |
| MOL000098 | quercetin | SELE |
| MOL000098 | quercetin | SERPINE |
| MOL000098 | quercetin | SLC2A4 |
| MOL000098 | quercetin | SOD1 |
| MOL000098 | quercetin | SPP1 |
| MOL000098 | quercetin | STAT1 |
| MOL000098 | quercetin | THBD |
| MOL000098 | quercetin | TNF |
| MOL000098 | quercetin | TOP1 |
| MOL000098 | quercetin | TOP2A |
| MOL000098 | quercetin | TP53 |
| MOL000098 | quercetin | UBE2O |
| MOL000098 | quercetin | VCAM1 |
| MOL000173 | wogonin | ADRB2 |
| MOL000173 | wogonin | AHSA1 |
| MOL000173 | wogonin | AKT1 |
| MOL000173 | wogonin | AR |
| MOL000173 | wogonin | BAX |
| MOL000173 | wogonin | BCL2 |
| MOL000173 | wogonin | CASP3 |
| MOL000173 | wogonin | CASP9 |
| MOL000173 | wogonin | CCND1 |
| MOL000173 | wogonin | CDKN1A |
| MOL000173 | wogonin | CHEK1 |
| MOL000173 | wogonin | CXCL8 |
| MOL000173 | wogonin | DPP4 |
| MOL000173 | wogonin | EIF6 |
| MOL000173 | wogonin | ESR1 |
| MOL000173 | wogonin | FN1 |
| MOL000173 | wogonin | GSK3B |
| MOL000173 | wogonin | HSP90AA |
| MOL000173 | wogonin | IL6 |
| MOL000173 | wogonin | JUN |
| MOL000173 | wogonin | KDR |
| MOL000173 | wogonin | MAPK14 |
| MOL000173 | wogonin | MCL1 |
| MOL000173 | wogonin | MMP1 |
| MOL000173 | wogonin | NOS2 |
| MOL000173 | wogonin | PDE3A |
| MOL000173 | wogonin | PPARG |
| MOL000173 | wogonin | PRKACA |
| MOL000173 | wogonin | PRKCD |
| MOL000173 | wogonin | PRSS1 |
| MOL000173 | wogonin | PTGS1 |
| MOL000173 | wogonin | PTGS2 |
| MOL000173 | wogonin | RELA |
| MOL000173 | wogonin | RXRA |
| MOL000173 | wogonin | SCN5A |
| MOL000173 | wogonin | TEP1 |
| MOL000173 | wogonin | TNF |
| MOL000173 | wogonin | TP53 |
| MOL000173 | wogonin | XDH |
| MOL000239 | Jaranol | AR |
| MOL000239 | Jaranol | CHEK1 |
| MOL000239 | Jaranol | DPP4 |
| MOL000239 | Jaranol | ESR2 |
| MOL000239 | Jaranol | HSP90AA |
| MOL000239 | Jaranol | NCOA2 |
| MOL000239 | Jaranol | NOS2 |
| MOL000239 | Jaranol | PRSS1 |
| MOL000239 | Jaranol | PTGS1 |
| MOL000239 | Jaranol | PTGS2 |
| MOL000239 | Jaranol | SCN5A |
| MOL000354 | isorhamnetin | ACHE |
| MOL000354 | isorhamnetin | AR |
| MOL000354 | isorhamnetin | CCNA2 |
| MOL000354 | isorhamnetin | CHEK1 |
| MOL000354 | isorhamnetin | CYP1B1 |
| MOL000354 | isorhamnetin | DPP4 |
| MOL000354 | isorhamnetin | ESR1 |
| MOL000354 | isorhamnetin | ESR2 |
| MOL000354 | isorhamnetin | F2 |
| MOL000354 | isorhamnetin | F7 |
| MOL000354 | isorhamnetin | GRIA2 |
| MOL000354 | isorhamnetin | GSK3B |
| MOL000354 | isorhamnetin | HSP90AA |
| MOL000354 | isorhamnetin | MAOB |
| MOL000354 | isorhamnetin | MAPK14 |
| MOL000354 | isorhamnetin | NCF1 |
| MOL000354 | isorhamnetin | NCOA1 |
| MOL000354 | isorhamnetin | NCOA2 |
| MOL000354 | isorhamnetin | NOS2 |
| MOL000354 | isorhamnetin | OLR1 |
| MOL000354 | isorhamnetin | PPARD |
| MOL000354 | isorhamnetin | PPARG |
| MOL000354 | isorhamnetin | PRKACA |
| MOL000354 | isorhamnetin | PRSS1 |
| MOL000354 | isorhamnetin | PTGS1 |
| MOL000354 | isorhamnetin | PTGS2 |
| MOL000354 | isorhamnetin | PTPN1 |
| MOL000354 | isorhamnetin | PYGM |
| MOL000354 | isorhamnetin | RELA |
| MOL000392 | formononetin | ACHE |
| MOL000392 | formononetin | ADRA1A |
| MOL000392 | formononetin | ADRB2 |
| MOL000392 | formononetin | AR |
| MOL000392 | formononetin | ATP5F1B |
| MOL000392 | formononetin | CCNA2 |
| MOL000392 | formononetin | CHEK1 |
| MOL000392 | formononetin | CHRM1 |
| MOL000392 | formononetin | DPP4 |
| MOL000392 | formononetin | ESR1 |
| MOL000392 | formononetin | ESR2 |
| MOL000392 | formononetin | F2 |
| MOL000392 | formononetin | GSK3B |
| MOL000392 | formononetin | HSD3B1 |
| MOL000392 | formononetin | HSD3B2 |
| MOL000392 | formononetin | HSP90AA |
| MOL000392 | formononetin | IL4 |
| MOL000392 | formononetin | JUN |
| MOL000392 | formononetin | MAOB |
| MOL000392 | formononetin | MAPK14 |
| MOL000392 | formononetin | MT-ND6 |
| MOL000392 | formononetin | NOS2 |
| MOL000392 | formononetin | PDE3A |
| MOL000392 | formononetin | PKIA |
| MOL000392 | formononetin | PPARG |
| MOL000392 | formononetin | PRKACA |
| MOL000392 | formononetin | PRSS1 |
| MOL000392 | formononetin | PTGS1 |
| MOL000392 | formononetin | PTGS2 |
| MOL000392 | formononetin | RXRA |
| MOL000392 | formononetin | SLC6A3 |
| MOL000392 | formononetin | SLC6A4 |
| MOL000417 | Calycosin | ADRB2 |
| MOL000417 | Calycosin | AR |
| MOL000417 | Calycosin | CCNA2 |
| MOL000417 | Calycosin | CHEK1 |
| MOL000417 | Calycosin | DPP4 |
| MOL000417 | Calycosin | ESR1 |
| MOL000417 | Calycosin | ESR2 |
| MOL000417 | Calycosin | GSK3B |
| MOL000417 | Calycosin | HSP90AA |
| MOL000417 | Calycosin | MAPK14 |
| MOL000417 | Calycosin | NCOA2 |
| MOL000417 | Calycosin | NOS2 |
| MOL000417 | Calycosin | PDE3A |
| MOL000417 | Calycosin | PPARG |
| MOL000417 | Calycosin | PRKACA |
| MOL000417 | Calycosin | PRSS1 |
| MOL000417 | Calycosin | PTGS1 |
| MOL000417 | Calycosin | PTGS2 |
| MOL000417 | Calycosin | RXRA |
| MOL000422 | kaempferol | ACHE |
| MOL000422 | kaempferol | ADRA1B |
| MOL000422 | kaempferol | AHR |
| MOL000422 | kaempferol | AHSA1 |
| MOL000422 | kaempferol | AKR1B1 |
| MOL000422 | kaempferol | AKR1C3 |
| MOL000422 | kaempferol | AKT1 |
| MOL000422 | kaempferol | AR |
| MOL000422 | kaempferol | BAX |
| MOL000422 | kaempferol | BCL2 |
| MOL000422 | kaempferol | CASP3 |
| MOL000422 | kaempferol | CHRM1 |
| MOL000422 | kaempferol | CHRM2 |
| MOL000422 | kaempferol | CYP1A1 |
| MOL000422 | kaempferol | CYP1B1 |
| MOL000422 | kaempferol | CYP3A4 |
| MOL000422 | kaempferol | DIO1 |
| MOL000422 | kaempferol | DPP4 |
| MOL000422 | kaempferol | F2 |
| MOL000422 | kaempferol | F7 |
| MOL000422 | kaempferol | GLO1 |
| MOL000422 | kaempferol | GSTM1 |
| MOL000422 | kaempferol | GSTM2 |
| MOL000422 | kaempferol | HAS2 |
| MOL000422 | kaempferol | HMOX1 |
| MOL000422 | kaempferol | HSP90AA |
| MOL000422 | kaempferol | ICAM1 |
| MOL000422 | kaempferol | IKBKB |
| MOL000422 | kaempferol | INSR |
| MOL000422 | kaempferol | JUN |
| MOL000422 | kaempferol | MAPK8 |
| MOL000422 | kaempferol | MMP1 |
| MOL000422 | kaempferol | NCOA2 |
| MOL000422 | kaempferol | NOS2 |
| MOL000422 | kaempferol | NR1I2 |
| MOL000422 | kaempferol | NR1I3 |
| MOL000422 | kaempferol | PGR |
| MOL000422 | kaempferol | PPARG |
| MOL000422 | kaempferol | PPP3CA |
| MOL000422 | kaempferol | PRKACA |
| MOL000422 | kaempferol | PRSS1 |
| MOL000422 | kaempferol | PSMD3 |
| MOL000422 | kaempferol | PTGS1 |
| MOL000422 | kaempferol | PTGS2 |
| MOL000422 | kaempferol | RELA |
| MOL000422 | kaempferol | SELE |
| MOL000422 | kaempferol | SLC2A4 |
| MOL000422 | kaempferol | SLC6A2 |
| MOL000422 | kaempferol | SLPI |
| MOL000422 | kaempferol | STAT1 |
| MOL000422 | kaempferol | TNF |
| MOL000422 | kaempferol | TOP2A |
| MOL000422 | kaempferol | UBE2O |
| MOL000422 | kaempferol | VCAM1 |
| MOL000490 | petunidin | ESR2 |
| MOL000490 | petunidin | GSK3B |
| MOL000490 | petunidin | HSP90AA |
| MOL000490 | petunidin | MAPK14 |
| MOL000490 | petunidin | NCOA2 |
| MOL000490 | petunidin | NOS2 |
| MOL000490 | petunidin | PTGS1 |
| MOL000490 | petunidin | PTGS2 |
| MOL000492 | (+)-catechin | ESR1 |
| MOL000492 | (+)-catechin | HAS2 |
| MOL000492 | (+)-catechin | HSP90AA |
| MOL000492 | (+)-catechin | NCOA2 |
| MOL000492 | (+)-catechin | PRKACA |
| MOL000492 | (+)-catechin | PTGS1 |
| MOL000492 | (+)-catechin | PTGS2 |
| MOL000492 | (+)-catechin | RXRA |
| MOL000492 | (+)-catechin | UBE2O |
| MOL000497 | licochalcone a | ADRA1B |
| MOL000497 | licochalcone a | ADRB2 |
| MOL000497 | licochalcone a | AR |
| MOL000497 | licochalcone a | BCL2 |
| MOL000497 | licochalcone a | CA2 |
| MOL000497 | licochalcone a | CCNA2 |
| MOL000497 | licochalcone a | CCND1 |
| MOL000497 | licochalcone a | CHEK1 |
| MOL000497 | licochalcone a | CHRM1 |
| MOL000497 | licochalcone a | EIF6 |
| MOL000497 | licochalcone a | ESR1 |
| MOL000497 | licochalcone a | ESR2 |
| MOL000497 | licochalcone a | F10 |
| MOL000497 | licochalcone a | FOSL2 |
| MOL000497 | licochalcone a | GSK3B |
| MOL000497 | licochalcone a | HSP90AA |
| MOL000497 | licochalcone a | MAPK1 |
| MOL000497 | licochalcone a | MAPK14 |
| MOL000497 | licochalcone a | NCOA2 |
| MOL000497 | licochalcone a | NOS2 |
| MOL000497 | licochalcone a | PPARG |
| MOL000497 | licochalcone a | PTGS1 |
| MOL000497 | licochalcone a | PTGS2 |
| MOL000497 | licochalcone a | RB1 |
| MOL000497 | licochalcone a | RELA |
| MOL000497 | licochalcone a | SCN5A |
| MOL000497 | licochalcone a | SLC6A3 |
| MOL000497 | licochalcone a | STAT3 |
| MOL000500 | Vestitol | ADRA1A |
| MOL000500 | Vestitol | ADRA1B |
| MOL000500 | Vestitol | ADRB2 |
| MOL000500 | Vestitol | AR |
| MOL000500 | Vestitol | CCNA2 |
| MOL000500 | Vestitol | CHEK1 |
| MOL000500 | Vestitol | CHRM1 |
| MOL000500 | Vestitol | CHRM4 |
| MOL000500 | Vestitol | DPP4 |
| MOL000500 | Vestitol | ESR1 |
| MOL000500 | Vestitol | ESR2 |
| MOL000500 | Vestitol | GSK3B |
| MOL000500 | Vestitol | HSP90AA |
| MOL000500 | Vestitol | MAPK14 |
| MOL000500 | Vestitol | NOS2 |
| MOL000500 | Vestitol | PDE3A |
| MOL000500 | Vestitol | PKIA |
| MOL000500 | Vestitol | PPARG |
| MOL000500 | Vestitol | PRKACA |
| MOL000500 | Vestitol | PRSS1 |
| MOL000500 | Vestitol | PTGS1 |
| MOL000500 | Vestitol | PTGS2 |
| MOL000500 | Vestitol | RXRA |
| MOL000500 | Vestitol | SCN5A |
| MOL000500 | Vestitol | SLC6A3 |
| MOL000500 | Vestitol | SLC6A4 |
| MOL000785 | palmatine | ADRB2 |
| MOL000785 | palmatine | AR |
| MOL000785 | palmatine | CYP3A4 |
| MOL000785 | palmatine | ESR1 |
| MOL000785 | palmatine | ESR2 |
| MOL000785 | palmatine | F7 |
| MOL000785 | palmatine | HSP90AA |
| MOL000785 | palmatine | KCNH2 |
| MOL000785 | palmatine | NCOA2 |
| MOL000785 | palmatine | NOS2 |
| MOL000785 | palmatine | PRKACA |
| MOL000785 | palmatine | PRSS1 |
| MOL000785 | palmatine | PTGS1 |
| MOL000785 | palmatine | PTGS2 |
| MOL000785 | palmatine | RXRA |
| MOL000785 | palmatine | SCN5A |
| MOL001002 | ellagic acid | AR |
| MOL001002 | ellagic acid | CDKN1A |
| MOL001002 | ellagic acid | CSNK2A1 |
| MOL001002 | ellagic acid | CXCL8 |
| MOL001002 | ellagic acid | ESR1 |
| MOL001002 | ellagic acid | GSTA2 |
| MOL001002 | ellagic acid | GSTM1 |
| MOL001002 | ellagic acid | GSTM2 |
| MOL001002 | ellagic acid | HSP90AA |
| MOL001002 | ellagic acid | IGF2 |
| MOL001002 | ellagic acid | MMP2 |
| MOL001002 | ellagic acid | MMP9 |
| MOL001002 | ellagic acid | NFKBIA |
| MOL001002 | ellagic acid | PGR |
| MOL001002 | ellagic acid | PRKCB |
| MOL001002 | ellagic acid | RELA |
| MOL001002 | ellagic acid | SMAD3 |
| MOL001002 | ellagic acid | SQLE |
| MOL001002 | ellagic acid | SYK |
| MOL001002 | ellagic acid | UBE2O |
| MOL001328 | 2,3-didehydro GA70 | CHRM1 |
| MOL001328 | 2,3-didehydro GA70 | GRIA2 |
| MOL001328 | 2,3-didehydro GA70 | PRSS1 |
| MOL001328 | 2,3-didehydro GA70 | PTGS1 |
| MOL001328 | 2,3-didehydro GA70 | PTGS2 |
| MOL001328 | 2,3-didehydro GA70 | SLC6A2 |
| MOL001329 | 2,3-didehydro GA77 | CA2 |
| MOL001329 | 2,3-didehydro GA77 | NCOA2 |
| MOL001329 | 2,3-didehydro GA77 | PTGS2 |
| MOL001340 | GA120 | CHRM1 |
| MOL001340 | GA120 | CHRM2 |
| MOL001340 | GA120 | CHRM3 |
| MOL001340 | GA120 | CHRNA7 |
| MOL001340 | GA120 | PTGS2 |
| MOL001342 | GA121-isolactone | PGR |
| MOL001344 | GA122-isolactone | PGR |
| MOL001349 | 4a-formyl-7alpha-hydroxy-1-methyl-8-methylidene-4aalpha,4bbeta-gibbane-1alpha,10beta-dicarboxylic acid | NR3C2 |
| MOL001349 | 4a-formyl-7alpha-hydroxy-1-methyl-8-methylidene-4aalpha,4bbeta-gibbane-1alpha,10beta-dicarboxylic acid | PGR |
| MOL001351 | Gibberellin A44 | NR3C2 |
| MOL001352 | GA54 | F10 |
| MOL001352 | GA54 | HSP90AA |
| MOL001352 | GA54 | NCOA2 |
| MOL001352 | GA54 | PTGS2 |
| MOL001352 | GA54 | PTPN1 |
| MOL001353 | GA60 | CHRM2 |
| MOL001353 | GA60 | GRIA2 |
| MOL001355 | GA63 | CHRNA7 |
| MOL001355 | GA63 | GRIA2 |
| MOL001355 | GA63 | PTGS2 |
| MOL001358 | gibberellin 7 | ADRB2 |
| MOL001358 | gibberellin 7 | CHRM1 |
| MOL001358 | gibberellin 7 | CHRM3 |
| MOL001358 | gibberellin 7 | PDE3A |
| MOL001358 | gibberellin 7 | PTGS2 |
| MOL001358 | gibberellin 7 | SLC6A3 |
| MOL001358 | gibberellin 7 | SLC6A4 |
| MOL001360 | GA77 | GRIA2 |
| MOL001361 | GA87 | CA2 |
| MOL001361 | GA87 | PTGS2 |
| MOL001368 | 3-O-p-coumaroylquinic acid | HSP90AA |
| MOL001368 | 3-O-p-coumaroylquinic acid | NCOA2 |
| MOL001368 | 3-O-p-coumaroylquinic acid | PRKACA |
| MOL001368 | 3-O-p-coumaroylquinic acid | PTGS1 |
| MOL001368 | 3-O-p-coumaroylquinic acid | PTGS2 |
| MOL001368 | 3-O-p-coumaroylquinic acid | PTPN1 |
| MOL001454 | berberine | ADRB2 |
| MOL001454 | berberine | AR |
| MOL001454 | berberine | ESR1 |
| MOL001454 | berberine | F10 |
| MOL001454 | berberine | HSP90AA |
| MOL001454 | berberine | KCNH2 |
| MOL001454 | berberine | NCOA2 |
| MOL001454 | berberine | NOS2 |
| MOL001454 | berberine | PDE10A |
| MOL001454 | berberine | PRKACA |
| MOL001454 | berberine | PRSS1 |
| MOL001454 | berberine | PTGS1 |
| MOL001454 | berberine | PTGS2 |
| MOL001454 | berberine | RXRA |
| MOL001454 | berberine | SCN5A |
| MOL001454 | berberine | TERT |
| MOL001458 | coptisine | AR |
| MOL001458 | coptisine | ESR1 |
| MOL001458 | coptisine | KCNH2 |
| MOL001458 | coptisine | NOS2 |
| MOL001458 | coptisine | PRSS1 |
| MOL001458 | coptisine | PTGS1 |
| MOL001458 | coptisine | PTGS2 |
| MOL001458 | coptisine | SCN5A |
| MOL001484 | Inermine | ADRA1B |
| MOL001484 | Inermine | ADRA1D |
| MOL001484 | Inermine | ADRB2 |
| MOL001484 | Inermine | CHRM1 |
| MOL001484 | Inermine | CHRM3 |
| MOL001484 | Inermine | HSP90AA |
| MOL001484 | Inermine | OPRM1 |
| MOL001484 | Inermine | PRKACA |
| MOL001484 | Inermine | PRSS1 |
| MOL001484 | Inermine | PTGS1 |
| MOL001484 | Inermine | PTGS2 |
| MOL001484 | Inermine | RXRA |
| MOL001484 | Inermine | SCN5A |
| MOL001689 | acacetin | ADRB2 |
| MOL001689 | acacetin | AR |
| MOL001689 | acacetin | BAX |
| MOL001689 | acacetin | BCL2 |
| MOL001689 | acacetin | CASP3 |
| MOL001689 | acacetin | CASP8 |
| MOL001689 | acacetin | CDKN1A |
| MOL001689 | acacetin | CHEK1 |
| MOL001689 | acacetin | CYP1B1 |
| MOL001689 | acacetin | DPP4 |
| MOL001689 | acacetin | FASLG |
| MOL001689 | acacetin | FASN |
| MOL001689 | acacetin | HSP90AA |
| MOL001689 | acacetin | NCOA1 |
| MOL001689 | acacetin | NCOA2 |
| MOL001689 | acacetin | NOS2 |
| MOL001689 | acacetin | PDE3A |
| MOL001689 | acacetin | PRKACA |
| MOL001689 | acacetin | PRSS1 |
| MOL001689 | acacetin | PTGS1 |
| MOL001689 | acacetin | PTGS2 |
| MOL001689 | acacetin | RELA |
| MOL001689 | acacetin | TP53 |
| MOL001689 | acacetin | XDH |
| MOL001792 | DFV | ADRB2 |
| MOL001792 | DFV | ESR1 |
| MOL001792 | DFV | HSP90AA |
| MOL001792 | DFV | MAOB |
| MOL001792 | DFV | PKIA |
| MOL001792 | DFV | PRKACA |
| MOL001792 | DFV | PTGS1 |
| MOL001792 | DFV | PTGS2 |
| MOL001792 | DFV | RXRA |
| MOL001792 | DFV | SLC6A4 |
| MOL002135 | Myricanone | ADRB2 |
| MOL002135 | Myricanone | AR |
| MOL002135 | Myricanone | CCNA2 |
| MOL002135 | Myricanone | CHEK1 |
| MOL002135 | Myricanone | DPP4 |
| MOL002135 | Myricanone | ESR1 |
| MOL002135 | Myricanone | ESR2 |
| MOL002135 | Myricanone | F2 |
| MOL002135 | Myricanone | F7 |
| MOL002135 | Myricanone | GSK3B |
| MOL002135 | Myricanone | HSP90AA |
| MOL002135 | Myricanone | KCNH2 |
| MOL002135 | Myricanone | KDR |
| MOL002135 | Myricanone | MAPK14 |
| MOL002135 | Myricanone | NCOA1 |
| MOL002135 | Myricanone | NOS2 |
| MOL002135 | Myricanone | PDE3A |
| MOL002135 | Myricanone | PPARG |
| MOL002135 | Myricanone | PTGS1 |
| MOL002135 | Myricanone | PTGS2 |
| MOL002135 | Myricanone | RXRA |
| MOL002135 | Myricanone | SCN5A |
| MOL002140 | Perlolyrine | F2 |
| MOL002140 | Perlolyrine | PRKACA |
| MOL002140 | Perlolyrine | PTGS2 |
| MOL002140 | Perlolyrine | RXRA |
| MOL002157 | wallichilide | NCOA2 |
| MOL002157 | wallichilide | NR3C1 |
| MOL002157 | wallichilide | NR3C2 |
| MOL002157 | wallichilide | PTGS2 |
| MOL002311 | Glycyrol | CCNA2 |
| MOL002311 | Glycyrol | CHEK1 |
| MOL002311 | Glycyrol | ESR1 |
| MOL002311 | Glycyrol | F2 |
| MOL002311 | Glycyrol | GSK3B |
| MOL002311 | Glycyrol | KDR |
| MOL002311 | Glycyrol | MAPK14 |
| MOL002311 | Glycyrol | NOS2 |
| MOL002311 | Glycyrol | PPARG |
| MOL002311 | Glycyrol | PTGS2 |
| MOL002341 | Hesperetin | DGAT1 |
| MOL002341 | Hesperetin | HSP90AA |
| MOL002341 | Hesperetin | NCOA1 |
| MOL002341 | Hesperetin | NCOA2 |
| MOL002341 | Hesperetin | PRKACA |
| MOL002341 | Hesperetin | PTGS1 |
| MOL002341 | Hesperetin | PTGS2 |
| MOL002341 | Hesperetin | SCN5A |
| MOL002565 | Medicarpin | ADRA1A |
| MOL002565 | Medicarpin | ADRA1B |
| MOL002565 | Medicarpin | ADRA1D |
| MOL002565 | Medicarpin | ADRB2 |
| MOL002565 | Medicarpin | CCNA2 |
| MOL002565 | Medicarpin | CHRM1 |
| MOL002565 | Medicarpin | CHRM2 |
| MOL002565 | Medicarpin | CHRM3 |
| MOL002565 | Medicarpin | CHRM4 |
| MOL002565 | Medicarpin | CHRM5 |
| MOL002565 | Medicarpin | CHRNA7 |
| MOL002565 | Medicarpin | DPP4 |
| MOL002565 | Medicarpin | ESR1 |
| MOL002565 | Medicarpin | ESR2 |
| MOL002565 | Medicarpin | HSP90AA |
| MOL002565 | Medicarpin | MAPK10 |
| MOL002565 | Medicarpin | NOS2 |
| MOL002565 | Medicarpin | OPRD1 |
| MOL002565 | Medicarpin | OPRM1 |
| MOL002565 | Medicarpin | PDE3A |
| MOL002565 | Medicarpin | PRKACA |
| MOL002565 | Medicarpin | PRSS1 |
| MOL002565 | Medicarpin | PTGS1 |
| MOL002565 | Medicarpin | PTGS2 |
| MOL002565 | Medicarpin | RXRA |
| MOL002565 | Medicarpin | SCN5A |
| MOL002565 | Medicarpin | SLC6A3 |
| MOL002565 | Medicarpin | SLC6A4 |
| MOL002694 | 4-[(E)-4-(3,5-dimethoxy-4-oxo-1-cyclohexa-2,5-dienylidene)but-2-enylidene]-2,6-dimethoxycyclohexa-2,5-dien-1-one | F2 |
| MOL002694 | 4-[(E)-4-(3,5-dimethoxy-4-oxo-1-cyclohexa-2,5-dienylidene)but-2-enylidene]-2,6-dimethoxycyclohexa-2,5-dien-1-one | NCOA2 |
| MOL002694 | 4-[(E)-4-(3,5-dimethoxy-4-oxo-1-cyclohexa-2,5-dienylidene)but-2-enylidene]-2,6-dimethoxycyclohexa-2,5-dien-1-one | PTGS2 |
| MOL002695 | lignan | CA2 |
| MOL002695 | lignan | ESR1 |
| MOL002695 | lignan | F10 |
| MOL002695 | lignan | NCOA2 |
| MOL002695 | lignan | PTGS2 |
| MOL002695 | lignan | TOP2A |
| MOL002710 | Pyrethrin II | NCOA2 |
| MOL002710 | Pyrethrin II | PTGS2 |
| MOL002712 | 6-Hydroxykaempferol | AR |
| MOL002712 | 6-Hydroxykaempferol | DPP4 |
| MOL002712 | 6-Hydroxykaempferol | HSP90AA |
| MOL002712 | 6-Hydroxykaempferol | NCOA2 |
| MOL002712 | 6-Hydroxykaempferol | NOS2 |
| MOL002712 | 6-Hydroxykaempferol | PPARG |
| MOL002712 | 6-Hydroxykaempferol | PRSS1 |
| MOL002712 | 6-Hydroxykaempferol | PTGS1 |
| MOL002712 | 6-Hydroxykaempferol | PTGS2 |
| MOL002714 | baicalein | AHR |
| MOL002714 | baicalein | AKT1 |
| MOL002714 | baicalein | APOD |
| MOL002714 | baicalein | AR |
| MOL002714 | baicalein | BAX |
| MOL002714 | baicalein | BCL2 |
| MOL002714 | baicalein | CASP3 |
| MOL002714 | baicalein | CCNB1 |
| MOL002714 | baicalein | CYCS |
| MOL002714 | baicalein | DPP4 |
| MOL002714 | baicalein | EGLN1 |
| MOL002714 | baicalein | FOS |
| MOL002714 | baicalein | FOSL1 |
| MOL002714 | baicalein | FOSL2 |
| MOL002714 | baicalein | GLO1 |
| MOL002714 | baicalein | HIF1A |
| MOL002714 | baicalein | HSP90AA |
| MOL002714 | baicalein | IGF2 |
| MOL002714 | baicalein | MMP9 |
| MOL002714 | baicalein | MPO |
| MOL002714 | baicalein | NCOA1 |
| MOL002714 | baicalein | NCOA2 |
| MOL002714 | baicalein | NFATC1 |
| MOL002714 | baicalein | NOX5 |
| MOL002714 | baicalein | PDE3A |
| MOL002714 | baicalein | PREP |
| MOL002714 | baicalein | PRKACA |
| MOL002714 | baicalein | PRSS1 |
| MOL002714 | baicalein | PTGS1 |
| MOL002714 | baicalein | PTGS2 |
| MOL002714 | baicalein | RELA |
| MOL002714 | baicalein | SELL |
| MOL002714 | baicalein | SELP |
| MOL002714 | baicalein | TDRD7 |
| MOL002714 | baicalein | TNF |
| MOL002714 | baicalein | TP53 |
| MOL002714 | baicalein | UBE2O |
| MOL002714 | baicalein | XDH |
| MOL002717 | qt_carthamone | HSP90AA |
| MOL002717 | qt_carthamone | PTGS1 |
| MOL002717 | qt_carthamone | PTGS2 |
| MOL002757 | 7,8-dimethyl-1H-pyrimido[5,6-g]quinoxaline-2,4-dione | HSP90AA |
| MOL002757 | 7,8-dimethyl-1H-pyrimido[5,6-g]quinoxaline-2,4-dione | PRKACA |
| MOL002757 | 7,8-dimethyl-1H-pyrimido[5,6-g]quinoxaline-2,4-dione | PTGS1 |
| MOL002757 | 7,8-dimethyl-1H-pyrimido[5,6-g]quinoxaline-2,4-dione | PTGS2 |
| MOL002897 | epiberberine | AR |
| MOL002897 | epiberberine | ESR1 |
| MOL002897 | epiberberine | KCNH2 |
| MOL002897 | epiberberine | NCOA2 |
| MOL002897 | epiberberine | NOS2 |
| MOL002897 | epiberberine | PDE10A |
| MOL002897 | epiberberine | PRKACA |
| MOL002897 | epiberberine | PRSS1 |
| MOL002897 | epiberberine | PTGS2 |
| MOL002897 | epiberberine | RXRA |
| MOL003656 | Lupiwighteone | AR |
| MOL003656 | Lupiwighteone | CCNA2 |
| MOL003656 | Lupiwighteone | CHEK1 |
| MOL003656 | Lupiwighteone | DPP4 |
| MOL003656 | Lupiwighteone | ESR1 |
| MOL003656 | Lupiwighteone | ESR2 |
| MOL003656 | Lupiwighteone | F10 |
| MOL003656 | Lupiwighteone | F2 |
| MOL003656 | Lupiwighteone | GSK3B |
| MOL003656 | Lupiwighteone | HSP90AA |
| MOL003656 | Lupiwighteone | MAPK14 |
| MOL003656 | Lupiwighteone | NCOA2 |
| MOL003656 | Lupiwighteone | NOS2 |
| MOL003656 | Lupiwighteone | PPARG |
| MOL003656 | Lupiwighteone | PRSS1 |
| MOL003656 | Lupiwighteone | PTGS2 |
| MOL003656 | Lupiwighteone | SCN5A |
| MOL003656 | Lupiwighteone | TOP2A |
| MOL003847 | Inophyllum E | AR |
| MOL003847 | Inophyllum E | ESR1 |
| MOL003847 | Inophyllum E | ESR2 |
| MOL003847 | Inophyllum E | F10 |
| MOL003847 | Inophyllum E | GSK3B |
| MOL003847 | Inophyllum E | PTGS1 |
| MOL003847 | Inophyllum E | PTGS2 |
| MOL003847 | Inophyllum E | TOP2A |
| MOL003896 | 7-Methoxy-2-methyl isoflavone | ACHE |
| MOL003896 | 7-Methoxy-2-methyl isoflavone | ADRA1B |
| MOL003896 | 7-Methoxy-2-methyl isoflavone | ADRA1D |
| MOL003896 | 7-Methoxy-2-methyl isoflavone | ADRB1 |
| MOL003896 | 7-Methoxy-2-methyl isoflavone | ADRB2 |
| MOL003896 | 7-Methoxy-2-methyl isoflavone | AR |
| MOL003896 | 7-Methoxy-2-methyl isoflavone | CCNA2 |
| MOL003896 | 7-Methoxy-2-methyl isoflavone | CHEK1 |
| MOL003896 | 7-Methoxy-2-methyl isoflavone | CHRM1 |
| MOL003896 | 7-Methoxy-2-methyl isoflavone | CHRM3 |
| MOL003896 | 7-Methoxy-2-methyl isoflavone | CHRM5 |
| MOL003896 | 7-Methoxy-2-methyl isoflavone | CHRNA7 |
| MOL003896 | 7-Methoxy-2-methyl isoflavone | DPP4 |
| MOL003896 | 7-Methoxy-2-methyl isoflavone | ESR1 |
| MOL003896 | 7-Methoxy-2-methyl isoflavone | ESR2 |
| MOL003896 | 7-Methoxy-2-methyl isoflavone | F2 |
| MOL003896 | 7-Methoxy-2-methyl isoflavone | GSK3B |
| MOL003896 | 7-Methoxy-2-methyl isoflavone | HSP90AA |
| MOL003896 | 7-Methoxy-2-methyl isoflavone | LTA4H |
| MOL003896 | 7-Methoxy-2-methyl isoflavone | MAOB |
| MOL003896 | 7-Methoxy-2-methyl isoflavone | MAPK14 |
| MOL003896 | 7-Methoxy-2-methyl isoflavone | NCOA1 |
| MOL003896 | 7-Methoxy-2-methyl isoflavone | NCOA2 |
| MOL003896 | 7-Methoxy-2-methyl isoflavone | NOS2 |
| MOL003896 | 7-Methoxy-2-methyl isoflavone | OPRM1 |
| MOL003896 | 7-Methoxy-2-methyl isoflavone | PDE3A |
| MOL003896 | 7-Methoxy-2-methyl isoflavone | PKIA |
| MOL003896 | 7-Methoxy-2-methyl isoflavone | PPARG |
| MOL003896 | 7-Methoxy-2-methyl isoflavone | PRKACA |
| MOL003896 | 7-Methoxy-2-methyl isoflavone | PRSS1 |
| MOL003896 | 7-Methoxy-2-methyl isoflavone | PTGS1 |
| MOL003896 | 7-Methoxy-2-methyl isoflavone | PTGS2 |
| MOL003896 | 7-Methoxy-2-methyl isoflavone | RXRA |
| MOL003896 | 7-Methoxy-2-methyl isoflavone | SCN5A |
| MOL003896 | 7-Methoxy-2-methyl isoflavone | SLC6A3 |
| MOL003896 | 7-Methoxy-2-methyl isoflavone | SLC6A4 |
| MOL004328 | naringenin | ABAT |
| MOL004328 | naringenin | ABCC1 |
| MOL004328 | naringenin | ADIPOQ |
| MOL004328 | naringenin | AKR1C1 |
| MOL004328 | naringenin | AKT1 |
| MOL004328 | naringenin | APOB |
| MOL004328 | naringenin | BCL2 |
| MOL004328 | naringenin | CASP3 |
| MOL004328 | naringenin | CES1 |
| MOL004328 | naringenin | CYP19A1 |
| MOL004328 | naringenin | CYP1B1 |
| MOL004328 | naringenin | ESR1 |
| MOL004328 | naringenin | ESR2 |
| MOL004328 | naringenin | FASN |
| MOL004328 | naringenin | GLO1 |
| MOL004328 | naringenin | GOT1 |
| MOL004328 | naringenin | GSR |
| MOL004328 | naringenin | HMGCR |
| MOL004328 | naringenin | HSP90AA |
| MOL004328 | naringenin | LDLR |
| MOL004328 | naringenin | MAPK1 |
| MOL004328 | naringenin | MAPK3 |
| MOL004328 | naringenin | MTTP |
| MOL004328 | naringenin | PLB1 |
| MOL004328 | naringenin | PPARA |
| MOL004328 | naringenin | PPARG |
| MOL004328 | naringenin | PRKACA |
| MOL004328 | naringenin | PTGS1 |
| MOL004328 | naringenin | PTGS2 |
| MOL004328 | naringenin | RELA |
| MOL004328 | naringenin | SOAT1 |
| MOL004328 | naringenin | SOAT2 |
| MOL004328 | naringenin | SOD1 |
| MOL004328 | naringenin | SREBF1 |
| MOL004328 | naringenin | UBE2O |
| MOL004580 | cis-Dihydroquercetin | HSP90AA |
| MOL004580 | cis-Dihydroquercetin | PTGS1 |
| MOL004580 | cis-Dihydroquercetin | PTGS2 |
| MOL004580 | cis-Dihydroquercetin | RXRA |
| MOL004598 | 3,5,6,7-tetramethoxy-2-(3,4,5-trimethoxyphenyl)chromone | ACHE |
| MOL004598 | 3,5,6,7-tetramethoxy-2-(3,4,5-trimethoxyphenyl)chromone | AR |
| MOL004598 | 3,5,6,7-tetramethoxy-2-(3,4,5-trimethoxyphenyl)chromone | ESR1 |
| MOL004598 | 3,5,6,7-tetramethoxy-2-(3,4,5-trimethoxyphenyl)chromone | ESR2 |
| MOL004598 | 3,5,6,7-tetramethoxy-2-(3,4,5-trimethoxyphenyl)chromone | F10 |
| MOL004598 | 3,5,6,7-tetramethoxy-2-(3,4,5-trimethoxyphenyl)chromone | F2 |
| MOL004598 | 3,5,6,7-tetramethoxy-2-(3,4,5-trimethoxyphenyl)chromone | F7 |
| MOL004598 | 3,5,6,7-tetramethoxy-2-(3,4,5-trimethoxyphenyl)chromone | NCOA2 |
| MOL004598 | 3,5,6,7-tetramethoxy-2-(3,4,5-trimethoxyphenyl)chromone | PRSS1 |
| MOL004598 | 3,5,6,7-tetramethoxy-2-(3,4,5-trimethoxyphenyl)chromone | PTGS2 |
| MOL004598 | 3,5,6,7-tetramethoxy-2-(3,4,5-trimethoxyphenyl)chromone | TOP2A |
| MOL004609 | Areapillin | AR |
| MOL004609 | Areapillin | DPP4 |
| MOL004609 | Areapillin | ESR2 |
| MOL004609 | Areapillin | F10 |
| MOL004609 | Areapillin | F2 |
| MOL004609 | Areapillin | F7 |
| MOL004609 | Areapillin | HSP90AA |
| MOL004609 | Areapillin | NCOA1 |
| MOL004609 | Areapillin | NCOA2 |
| MOL004609 | Areapillin | NOS2 |
| MOL004609 | Areapillin | PRSS1 |
| MOL004609 | Areapillin | PTGS2 |
| MOL004609 | Areapillin | PTPN1 |
| MOL004609 | Areapillin | SCN5A |
| MOL004609 | Areapillin | TOP2A |
| MOL004624 | Longikaurin A | CHRM1 |
| MOL004624 | Longikaurin A | CHRM2 |
| MOL004624 | Longikaurin A | PRSS1 |
| MOL004808 | glyasperin B | ACHE |
| MOL004808 | glyasperin B | AR |
| MOL004808 | glyasperin B | CCNA2 |
| MOL004808 | glyasperin B | DPP4 |
| MOL004808 | glyasperin B | ESR1 |
| MOL004808 | glyasperin B | ESR2 |
| MOL004808 | glyasperin B | F10 |
| MOL004808 | glyasperin B | F2 |
| MOL004808 | glyasperin B | F7 |
| MOL004808 | glyasperin B | GSK3B |
| MOL004808 | glyasperin B | HSP90AA |
| MOL004808 | glyasperin B | KDR |
| MOL004808 | glyasperin B | NCOA2 |
| MOL004808 | glyasperin B | NOS2 |
| MOL004808 | glyasperin B | PPARG |
| MOL004808 | glyasperin B | PRSS1 |
| MOL004808 | glyasperin B | PTGS2 |
| MOL004808 | glyasperin B | TOP2A |
| MOL004810 | glyasperin F | AR |
| MOL004810 | glyasperin F | CCNA2 |
| MOL004810 | glyasperin F | ESR1 |
| MOL004810 | glyasperin F | ESR2 |
| MOL004810 | glyasperin F | F10 |
| MOL004810 | glyasperin F | GSK3B |
| MOL004810 | glyasperin F | HSP90AA |
| MOL004810 | glyasperin F | MAPK14 |
| MOL004810 | glyasperin F | NOS2 |
| MOL004810 | glyasperin F | PPARG |
| MOL004810 | glyasperin F | PRSS1 |
| MOL004810 | glyasperin F | PTGS1 |
| MOL004810 | glyasperin F | PTGS2 |
| MOL004810 | glyasperin F | SCN5A |
| MOL004810 | glyasperin F | TOP2A |
| MOL004811 | Glyasperin C | ACHE |
| MOL004811 | Glyasperin C | AR |
| MOL004811 | Glyasperin C | CCNA2 |
| MOL004811 | Glyasperin C | CHEK1 |
| MOL004811 | Glyasperin C | DPP4 |
| MOL004811 | Glyasperin C | ESR1 |
| MOL004811 | Glyasperin C | ESR2 |
| MOL004811 | Glyasperin C | F10 |
| MOL004811 | Glyasperin C | F2 |
| MOL004811 | Glyasperin C | GSK3B |
| MOL004811 | Glyasperin C | HSP90AA |
| MOL004811 | Glyasperin C | KCNH2 |
| MOL004811 | Glyasperin C | MAPK14 |
| MOL004811 | Glyasperin C | NCOA2 |
| MOL004811 | Glyasperin C | NOS2 |
| MOL004811 | Glyasperin C | PPARG |
| MOL004811 | Glyasperin C | PRSS1 |
| MOL004811 | Glyasperin C | PTGS2 |
| MOL004811 | Glyasperin C | RXRA |
| MOL004811 | Glyasperin C | SCN5A |
| MOL004811 | Glyasperin C | TOP2A |
| MOL004814 | Isotrifoliol | AR |
| MOL004814 | Isotrifoliol | CCNA2 |
| MOL004814 | Isotrifoliol | CHEK1 |
| MOL004814 | Isotrifoliol | ESR1 |
| MOL004814 | Isotrifoliol | ESR2 |
| MOL004814 | Isotrifoliol | GSK3B |
| MOL004814 | Isotrifoliol | HSP90AA |
| MOL004814 | Isotrifoliol | MAPK14 |
| MOL004814 | Isotrifoliol | NOS2 |
| MOL004814 | Isotrifoliol | PRKACA |
| MOL004814 | Isotrifoliol | PTGS2 |
| MOL004815 | (E)-1-(2,4-dihydroxyphenyl)-3-(2,2-dimethylchromen-6-yl)prop-2-en-1-one | ADRA1B |
| MOL004815 | (E)-1-(2,4-dihydroxyphenyl)-3-(2,2-dimethylchromen-6-yl)prop-2-en-1-one | AR |
| MOL004815 | (E)-1-(2,4-dihydroxyphenyl)-3-(2,2-dimethylchromen-6-yl)prop-2-en-1-one | CA2 |
| MOL004815 | (E)-1-(2,4-dihydroxyphenyl)-3-(2,2-dimethylchromen-6-yl)prop-2-en-1-one | CCNA2 |
| MOL004815 | (E)-1-(2,4-dihydroxyphenyl)-3-(2,2-dimethylchromen-6-yl)prop-2-en-1-one | CHEK1 |
| MOL004815 | (E)-1-(2,4-dihydroxyphenyl)-3-(2,2-dimethylchromen-6-yl)prop-2-en-1-one | ESR1 |
| MOL004815 | (E)-1-(2,4-dihydroxyphenyl)-3-(2,2-dimethylchromen-6-yl)prop-2-en-1-one | ESR2 |
| MOL004815 | (E)-1-(2,4-dihydroxyphenyl)-3-(2,2-dimethylchromen-6-yl)prop-2-en-1-one | F10 |
| MOL004815 | (E)-1-(2,4-dihydroxyphenyl)-3-(2,2-dimethylchromen-6-yl)prop-2-en-1-one | GSK3B |
| MOL004815 | (E)-1-(2,4-dihydroxyphenyl)-3-(2,2-dimethylchromen-6-yl)prop-2-en-1-one | MAPK14 |
| MOL004815 | (E)-1-(2,4-dihydroxyphenyl)-3-(2,2-dimethylchromen-6-yl)prop-2-en-1-one | NCOA2 |
| MOL004815 | (E)-1-(2,4-dihydroxyphenyl)-3-(2,2-dimethylchromen-6-yl)prop-2-en-1-one | NOS2 |
| MOL004815 | (E)-1-(2,4-dihydroxyphenyl)-3-(2,2-dimethylchromen-6-yl)prop-2-en-1-one | PPARG |
| MOL004815 | (E)-1-(2,4-dihydroxyphenyl)-3-(2,2-dimethylchromen-6-yl)prop-2-en-1-one | PTGS1 |
| MOL004815 | (E)-1-(2,4-dihydroxyphenyl)-3-(2,2-dimethylchromen-6-yl)prop-2-en-1-one | PTGS2 |
| MOL004815 | (E)-1-(2,4-dihydroxyphenyl)-3-(2,2-dimethylchromen-6-yl)prop-2-en-1-one | RXRA |
| MOL004815 | (E)-1-(2,4-dihydroxyphenyl)-3-(2,2-dimethylchromen-6-yl)prop-2-en-1-one | SCN5A |
| MOL004820 | kanzonols W | AR |
| MOL004820 | kanzonols W | CCNA2 |
| MOL004820 | kanzonols W | CHEK1 |
| MOL004820 | kanzonols W | ESR1 |
| MOL004820 | kanzonols W | ESR2 |
| MOL004820 | kanzonols W | F10 |
| MOL004820 | kanzonols W | GSK3B |
| MOL004820 | kanzonols W | MAPK14 |
| MOL004820 | kanzonols W | NCOA1 |
| MOL004820 | kanzonols W | NCOA2 |
| MOL004820 | kanzonols W | NOS2 |
| MOL004820 | kanzonols W | PPARG |
| MOL004820 | kanzonols W | PRSS1 |
| MOL004820 | kanzonols W | PTGS1 |
| MOL004820 | kanzonols W | PTGS2 |
| MOL004820 | kanzonols W | RXRA |
| MOL004820 | kanzonols W | SCN5A |
| MOL004820 | kanzonols W | TOP2A |
| MOL004824 | (2S)-6-(2,4-dihydroxyphenyl)-2-(2-hydroxypropan-2-yl)-4-methoxy-2,3-dihydrofuro[3,2-g]chromen-7-one | ACHE |
| MOL004824 | (2S)-6-(2,4-dihydroxyphenyl)-2-(2-hydroxypropan-2-yl)-4-methoxy-2,3-dihydrofuro[3,2-g]chromen-7-one | AR |
| MOL004824 | (2S)-6-(2,4-dihydroxyphenyl)-2-(2-hydroxypropan-2-yl)-4-methoxy-2,3-dihydrofuro[3,2-g]chromen-7-one | CCNA2 |
| MOL004824 | (2S)-6-(2,4-dihydroxyphenyl)-2-(2-hydroxypropan-2-yl)-4-methoxy-2,3-dihydrofuro[3,2-g]chromen-7-one | CHEK1 |
| MOL004824 | (2S)-6-(2,4-dihydroxyphenyl)-2-(2-hydroxypropan-2-yl)-4-methoxy-2,3-dihydrofuro[3,2-g]chromen-7-one | DPP4 |
| MOL004824 | (2S)-6-(2,4-dihydroxyphenyl)-2-(2-hydroxypropan-2-yl)-4-methoxy-2,3-dihydrofuro[3,2-g]chromen-7-one | ESR1 |
| MOL004824 | (2S)-6-(2,4-dihydroxyphenyl)-2-(2-hydroxypropan-2-yl)-4-methoxy-2,3-dihydrofuro[3,2-g]chromen-7-one | ESR2 |
| MOL004824 | (2S)-6-(2,4-dihydroxyphenyl)-2-(2-hydroxypropan-2-yl)-4-methoxy-2,3-dihydrofuro[3,2-g]chromen-7-one | F10 |
| MOL004824 | (2S)-6-(2,4-dihydroxyphenyl)-2-(2-hydroxypropan-2-yl)-4-methoxy-2,3-dihydrofuro[3,2-g]chromen-7-one | F2 |
| MOL004824 | (2S)-6-(2,4-dihydroxyphenyl)-2-(2-hydroxypropan-2-yl)-4-methoxy-2,3-dihydrofuro[3,2-g]chromen-7-one | F7 |
| MOL004824 | (2S)-6-(2,4-dihydroxyphenyl)-2-(2-hydroxypropan-2-yl)-4-methoxy-2,3-dihydrofuro[3,2-g]chromen-7-one | GSK3B |
| MOL004824 | (2S)-6-(2,4-dihydroxyphenyl)-2-(2-hydroxypropan-2-yl)-4-methoxy-2,3-dihydrofuro[3,2-g]chromen-7-one | KDR |
| MOL004824 | (2S)-6-(2,4-dihydroxyphenyl)-2-(2-hydroxypropan-2-yl)-4-methoxy-2,3-dihydrofuro[3,2-g]chromen-7-one | MAPK14 |
| MOL004824 | (2S)-6-(2,4-dihydroxyphenyl)-2-(2-hydroxypropan-2-yl)-4-methoxy-2,3-dihydrofuro[3,2-g]chromen-7-one | NOS2 |
| MOL004824 | (2S)-6-(2,4-dihydroxyphenyl)-2-(2-hydroxypropan-2-yl)-4-methoxy-2,3-dihydrofuro[3,2-g]chromen-7-one | PPARG |
| MOL004824 | (2S)-6-(2,4-dihydroxyphenyl)-2-(2-hydroxypropan-2-yl)-4-methoxy-2,3-dihydrofuro[3,2-g]chromen-7-one | PRSS1 |
| MOL004824 | (2S)-6-(2,4-dihydroxyphenyl)-2-(2-hydroxypropan-2-yl)-4-methoxy-2,3-dihydrofuro[3,2-g]chromen-7-one | PTGS2 |
| MOL004824 | (2S)-6-(2,4-dihydroxyphenyl)-2-(2-hydroxypropan-2-yl)-4-methoxy-2,3-dihydrofuro[3,2-g]chromen-7-one | TOP2A |
| MOL004827 | Semilicoisoflavone B | ACHE |
| MOL004827 | Semilicoisoflavone B | AR |
| MOL004827 | Semilicoisoflavone B | CHEK1 |
| MOL004827 | Semilicoisoflavone B | ESR1 |
| MOL004827 | Semilicoisoflavone B | F10 |
| MOL004827 | Semilicoisoflavone B | F2 |
| MOL004827 | Semilicoisoflavone B | F7 |
| MOL004827 | Semilicoisoflavone B | GSK3B |
| MOL004827 | Semilicoisoflavone B | HSP90AA |
| MOL004827 | Semilicoisoflavone B | NOS2 |
| MOL004827 | Semilicoisoflavone B | PPARG |
| MOL004827 | Semilicoisoflavone B | PRSS1 |
| MOL004827 | Semilicoisoflavone B | PTGS2 |
| MOL004827 | Semilicoisoflavone B | SCN5A |
| MOL004827 | Semilicoisoflavone B | TOP2A |
| MOL004828 | Glepidotin A | AR |
| MOL004828 | Glepidotin A | CCNA2 |
| MOL004828 | Glepidotin A | CHEK1 |
| MOL004828 | Glepidotin A | DPP4 |
| MOL004828 | Glepidotin A | ESR1 |
| MOL004828 | Glepidotin A | F10 |
| MOL004828 | Glepidotin A | F2 |
| MOL004828 | Glepidotin A | F7 |
| MOL004828 | Glepidotin A | GSK3B |
| MOL004828 | Glepidotin A | HSP90AA |
| MOL004828 | Glepidotin A | KDR |
| MOL004828 | Glepidotin A | MAPK14 |
| MOL004828 | Glepidotin A | NOS2 |
| MOL004828 | Glepidotin A | PDE3A |
| MOL004828 | Glepidotin A | PPARG |
| MOL004828 | Glepidotin A | PRSS1 |
| MOL004828 | Glepidotin A | PTGS1 |
| MOL004828 | Glepidotin A | PTGS2 |
| MOL004828 | Glepidotin A | RXRA |
| MOL004828 | Glepidotin A | SCN5A |
| MOL004828 | Glepidotin A | TOP2A |
| MOL004829 | Glepidotin B | ADRA1B |
| MOL004829 | Glepidotin B | ESR1 |
| MOL004829 | Glepidotin B | F10 |
| MOL004829 | Glepidotin B | F7 |
| MOL004829 | Glepidotin B | HSP90AA |
| MOL004829 | Glepidotin B | NCOA1 |
| MOL004829 | Glepidotin B | PDE3A |
| MOL004829 | Glepidotin B | PTGS1 |
| MOL004829 | Glepidotin B | PTGS2 |
| MOL004829 | Glepidotin B | RXRA |
| MOL004829 | Glepidotin B | SCN5A |
| MOL004829 | Glepidotin B | TOP2A |
| MOL004833 | Phaseolinisoflavan | ACHE |
| MOL004833 | Phaseolinisoflavan | ADRA1B |
| MOL004833 | Phaseolinisoflavan | ADRB2 |
| MOL004833 | Phaseolinisoflavan | AR |
| MOL004833 | Phaseolinisoflavan | CCNA2 |
| MOL004833 | Phaseolinisoflavan | CHEK1 |
| MOL004833 | Phaseolinisoflavan | CHRM1 |
| MOL004833 | Phaseolinisoflavan | ESR1 |
| MOL004833 | Phaseolinisoflavan | ESR2 |
| MOL004833 | Phaseolinisoflavan | F10 |
| MOL004833 | Phaseolinisoflavan | GSK3B |
| MOL004833 | Phaseolinisoflavan | MAPK14 |
| MOL004833 | Phaseolinisoflavan | NCOA1 |
| MOL004833 | Phaseolinisoflavan | NOS2 |
| MOL004833 | Phaseolinisoflavan | PPARG |
| MOL004833 | Phaseolinisoflavan | PRSS1 |
| MOL004833 | Phaseolinisoflavan | PTGS2 |
| MOL004833 | Phaseolinisoflavan | RXRA |
| MOL004833 | Phaseolinisoflavan | SCN5A |
| MOL004835 | Glypallichalcone | ADRA1B |
| MOL004835 | Glypallichalcone | ADRB2 |
| MOL004835 | Glypallichalcone | AR |
| MOL004835 | Glypallichalcone | CA2 |
| MOL004835 | Glypallichalcone | CCNA2 |
| MOL004835 | Glypallichalcone | CHEK1 |
| MOL004835 | Glypallichalcone | CHRM1 |
| MOL004835 | Glypallichalcone | ESR1 |
| MOL004835 | Glypallichalcone | ESR2 |
| MOL004835 | Glypallichalcone | GSK3B |
| MOL004835 | Glypallichalcone | HSP90AA |
| MOL004835 | Glypallichalcone | LTA4H |
| MOL004835 | Glypallichalcone | MAOB |
| MOL004835 | Glypallichalcone | MAPK14 |
| MOL004835 | Glypallichalcone | NCOA1 |
| MOL004835 | Glypallichalcone | NOS2 |
| MOL004835 | Glypallichalcone | PDE3A |
| MOL004835 | Glypallichalcone | PKIA |
| MOL004835 | Glypallichalcone | PPARG |
| MOL004835 | Glypallichalcone | PRKACA |
| MOL004835 | Glypallichalcone | PTGS1 |
| MOL004835 | Glypallichalcone | PTGS2 |
| MOL004835 | Glypallichalcone | SCN5A |
| MOL004835 | Glypallichalcone | SLC6A3 |
| MOL004835 | Glypallichalcone | SLC6A4 |
| MOL004838 | 8-(6-hydroxy-2-benzofuranyl)-2,2-dimethyl-5-chromenol | ESR1 |
| MOL004838 | 8-(6-hydroxy-2-benzofuranyl)-2,2-dimethyl-5-chromenol | HSP90AA |
| MOL004838 | 8-(6-hydroxy-2-benzofuranyl)-2,2-dimethyl-5-chromenol | NOS2 |
| MOL004838 | 8-(6-hydroxy-2-benzofuranyl)-2,2-dimethyl-5-chromenol | PTGS2 |
| MOL004838 | 8-(6-hydroxy-2-benzofuranyl)-2,2-dimethyl-5-chromenol | RXRA |
| MOL004841 | Licochalcone B | ADRB2 |
| MOL004841 | Licochalcone B | AR |
| MOL004841 | Licochalcone B | CA2 |
| MOL004841 | Licochalcone B | CCNA2 |
| MOL004841 | Licochalcone B | CHEK1 |
| MOL004841 | Licochalcone B | ESR1 |
| MOL004841 | Licochalcone B | ESR2 |
| MOL004841 | Licochalcone B | GSK3B |
| MOL004841 | Licochalcone B | HSP90AA |
| MOL004841 | Licochalcone B | MAPK14 |
| MOL004841 | Licochalcone B | NOS2 |
| MOL004841 | Licochalcone B | PDE3A |
| MOL004841 | Licochalcone B | PPARG |
| MOL004841 | Licochalcone B | PRKACA |
| MOL004841 | Licochalcone B | PTGS1 |
| MOL004841 | Licochalcone B | PTGS2 |
| MOL004848 | licochalcone G | AR |
| MOL004848 | licochalcone G | CCNA2 |
| MOL004848 | licochalcone G | ESR1 |
| MOL004848 | licochalcone G | ESR2 |
| MOL004848 | licochalcone G | F10 |
| MOL004848 | licochalcone G | GSK3B |
| MOL004848 | licochalcone G | HSP90AA |
| MOL004848 | licochalcone G | KDR |
| MOL004848 | licochalcone G | MAPK14 |
| MOL004848 | licochalcone G | NCOA2 |
| MOL004848 | licochalcone G | NOS2 |
| MOL004848 | licochalcone G | PPARG |
| MOL004848 | licochalcone G | PTGS2 |
| MOL004849 | 3-(2,4-dihydroxyphenyl)-8-(1,1-dimethylprop-2-enyl)-7-hydroxy-5-methoxy-coumarin | AR |
| MOL004849 | 3-(2,4-dihydroxyphenyl)-8-(1,1-dimethylprop-2-enyl)-7-hydroxy-5-methoxy-coumarin | CHEK1 |
| MOL004849 | 3-(2,4-dihydroxyphenyl)-8-(1,1-dimethylprop-2-enyl)-7-hydroxy-5-methoxy-coumarin | DPP4 |
| MOL004849 | 3-(2,4-dihydroxyphenyl)-8-(1,1-dimethylprop-2-enyl)-7-hydroxy-5-methoxy-coumarin | ESR1 |
| MOL004849 | 3-(2,4-dihydroxyphenyl)-8-(1,1-dimethylprop-2-enyl)-7-hydroxy-5-methoxy-coumarin | ESR2 |
| MOL004849 | 3-(2,4-dihydroxyphenyl)-8-(1,1-dimethylprop-2-enyl)-7-hydroxy-5-methoxy-coumarin | F10 |
| MOL004849 | 3-(2,4-dihydroxyphenyl)-8-(1,1-dimethylprop-2-enyl)-7-hydroxy-5-methoxy-coumarin | F2 |
| MOL004849 | 3-(2,4-dihydroxyphenyl)-8-(1,1-dimethylprop-2-enyl)-7-hydroxy-5-methoxy-coumarin | F7 |
| MOL004849 | 3-(2,4-dihydroxyphenyl)-8-(1,1-dimethylprop-2-enyl)-7-hydroxy-5-methoxy-coumarin | GSK3B |
| MOL004849 | 3-(2,4-dihydroxyphenyl)-8-(1,1-dimethylprop-2-enyl)-7-hydroxy-5-methoxy-coumarin | HSP90AA |
| MOL004849 | 3-(2,4-dihydroxyphenyl)-8-(1,1-dimethylprop-2-enyl)-7-hydroxy-5-methoxy-coumarin | KCNH2 |
| MOL004849 | 3-(2,4-dihydroxyphenyl)-8-(1,1-dimethylprop-2-enyl)-7-hydroxy-5-methoxy-coumarin | KDR |
| MOL004849 | 3-(2,4-dihydroxyphenyl)-8-(1,1-dimethylprop-2-enyl)-7-hydroxy-5-methoxy-coumarin | MAPK14 |
| MOL004849 | 3-(2,4-dihydroxyphenyl)-8-(1,1-dimethylprop-2-enyl)-7-hydroxy-5-methoxy-coumarin | NCOA1 |
| MOL004849 | 3-(2,4-dihydroxyphenyl)-8-(1,1-dimethylprop-2-enyl)-7-hydroxy-5-methoxy-coumarin | NCOA2 |
| MOL004849 | 3-(2,4-dihydroxyphenyl)-8-(1,1-dimethylprop-2-enyl)-7-hydroxy-5-methoxy-coumarin | NOS2 |
| MOL004849 | 3-(2,4-dihydroxyphenyl)-8-(1,1-dimethylprop-2-enyl)-7-hydroxy-5-methoxy-coumarin | PPARG |
| MOL004849 | 3-(2,4-dihydroxyphenyl)-8-(1,1-dimethylprop-2-enyl)-7-hydroxy-5-methoxy-coumarin | PRSS1 |
| MOL004849 | 3-(2,4-dihydroxyphenyl)-8-(1,1-dimethylprop-2-enyl)-7-hydroxy-5-methoxy-coumarin | PTGS2 |
| MOL004849 | 3-(2,4-dihydroxyphenyl)-8-(1,1-dimethylprop-2-enyl)-7-hydroxy-5-methoxy-coumarin | TOP2A |
| MOL004855 | Licoricone | AR |
| MOL004855 | Licoricone | CHEK1 |
| MOL004855 | Licoricone | ESR1 |
| MOL004855 | Licoricone | F10 |
| MOL004855 | Licoricone | F2 |
| MOL004855 | Licoricone | KCNH2 |
| MOL004855 | Licoricone | KDR |
| MOL004855 | Licoricone | NCOA2 |
| MOL004855 | Licoricone | NOS2 |
| MOL004855 | Licoricone | PPARG |
| MOL004855 | Licoricone | PRSS1 |
| MOL004855 | Licoricone | PTGS2 |
| MOL004855 | Licoricone | TOP2A |
| MOL004856 | Gancaonin A | ACHE |
| MOL004856 | Gancaonin A | AR |
| MOL004856 | Gancaonin A | CCNA2 |
| MOL004856 | Gancaonin A | CHEK1 |
| MOL004856 | Gancaonin A | DPP4 |
| MOL004856 | Gancaonin A | ESR1 |
| MOL004856 | Gancaonin A | ESR2 |
| MOL004856 | Gancaonin A | F10 |
| MOL004856 | Gancaonin A | F2 |
| MOL004856 | Gancaonin A | GSK3B |
| MOL004856 | Gancaonin A | HSP90AA |
| MOL004856 | Gancaonin A | NCOA2 |
| MOL004856 | Gancaonin A | NOS2 |
| MOL004856 | Gancaonin A | PPARG |
| MOL004856 | Gancaonin A | PRSS1 |
| MOL004856 | Gancaonin A | PTGS2 |
| MOL004856 | Gancaonin A | SCN5A |
| MOL004856 | Gancaonin A | TOP2A |
| MOL004857 | Gancaonin B | ADRA1B |
| MOL004857 | Gancaonin B | ADRB2 |
| MOL004857 | Gancaonin B | AR |
| MOL004857 | Gancaonin B | CCNA2 |
| MOL004857 | Gancaonin B | CHEK1 |
| MOL004857 | Gancaonin B | DPP4 |
| MOL004857 | Gancaonin B | ESR1 |
| MOL004857 | Gancaonin B | ESR2 |
| MOL004857 | Gancaonin B | F10 |
| MOL004857 | Gancaonin B | F2 |
| MOL004857 | Gancaonin B | F7 |
| MOL004857 | Gancaonin B | GSK3B |
| MOL004857 | Gancaonin B | HSP90AA |
| MOL004857 | Gancaonin B | KDR |
| MOL004857 | Gancaonin B | NCOA2 |
| MOL004857 | Gancaonin B | NOS2 |
| MOL004857 | Gancaonin B | PPARG |
| MOL004857 | Gancaonin B | PRSS1 |
| MOL004857 | Gancaonin B | PTGS2 |
| MOL004857 | Gancaonin B | TOP2A |
| MOL004863 | 3-(3,4-dihydroxyphenyl)-5,7-dihydroxy-8-(3-methylbut-2-enyl)chromone | AR |
| MOL004863 | 3-(3,4-dihydroxyphenyl)-5,7-dihydroxy-8-(3-methylbut-2-enyl)chromone | CCNA2 |
| MOL004863 | 3-(3,4-dihydroxyphenyl)-5,7-dihydroxy-8-(3-methylbut-2-enyl)chromone | CHEK1 |
| MOL004863 | 3-(3,4-dihydroxyphenyl)-5,7-dihydroxy-8-(3-methylbut-2-enyl)chromone | ESR1 |
| MOL004863 | 3-(3,4-dihydroxyphenyl)-5,7-dihydroxy-8-(3-methylbut-2-enyl)chromone | F10 |
| MOL004863 | 3-(3,4-dihydroxyphenyl)-5,7-dihydroxy-8-(3-methylbut-2-enyl)chromone | F2 |
| MOL004863 | 3-(3,4-dihydroxyphenyl)-5,7-dihydroxy-8-(3-methylbut-2-enyl)chromone | GSK3B |
| MOL004863 | 3-(3,4-dihydroxyphenyl)-5,7-dihydroxy-8-(3-methylbut-2-enyl)chromone | HSP90AA |
| MOL004863 | 3-(3,4-dihydroxyphenyl)-5,7-dihydroxy-8-(3-methylbut-2-enyl)chromone | MAPK14 |
| MOL004863 | 3-(3,4-dihydroxyphenyl)-5,7-dihydroxy-8-(3-methylbut-2-enyl)chromone | NCOA2 |
| MOL004863 | 3-(3,4-dihydroxyphenyl)-5,7-dihydroxy-8-(3-methylbut-2-enyl)chromone | NOS2 |
| MOL004863 | 3-(3,4-dihydroxyphenyl)-5,7-dihydroxy-8-(3-methylbut-2-enyl)chromone | PPARG |
| MOL004863 | 3-(3,4-dihydroxyphenyl)-5,7-dihydroxy-8-(3-methylbut-2-enyl)chromone | PRSS1 |
| MOL004863 | 3-(3,4-dihydroxyphenyl)-5,7-dihydroxy-8-(3-methylbut-2-enyl)chromone | PTGS2 |
| MOL004863 | 3-(3,4-dihydroxyphenyl)-5,7-dihydroxy-8-(3-methylbut-2-enyl)chromone | PTPN1 |
| MOL004864 | 5,7-dihydroxy-3-(4-methoxyphenyl)-8-(3-methylbut-2-enyl)chromone | AR |
| MOL004864 | 5,7-dihydroxy-3-(4-methoxyphenyl)-8-(3-methylbut-2-enyl)chromone | CCNA2 |
| MOL004864 | 5,7-dihydroxy-3-(4-methoxyphenyl)-8-(3-methylbut-2-enyl)chromone | CHEK1 |
| MOL004864 | 5,7-dihydroxy-3-(4-methoxyphenyl)-8-(3-methylbut-2-enyl)chromone | DPP4 |
| MOL004864 | 5,7-dihydroxy-3-(4-methoxyphenyl)-8-(3-methylbut-2-enyl)chromone | ESR1 |
| MOL004864 | 5,7-dihydroxy-3-(4-methoxyphenyl)-8-(3-methylbut-2-enyl)chromone | ESR2 |
| MOL004864 | 5,7-dihydroxy-3-(4-methoxyphenyl)-8-(3-methylbut-2-enyl)chromone | F10 |
| MOL004864 | 5,7-dihydroxy-3-(4-methoxyphenyl)-8-(3-methylbut-2-enyl)chromone | GSK3B |
| MOL004864 | 5,7-dihydroxy-3-(4-methoxyphenyl)-8-(3-methylbut-2-enyl)chromone | HSP90AA |
| MOL004864 | 5,7-dihydroxy-3-(4-methoxyphenyl)-8-(3-methylbut-2-enyl)chromone | KCNH2 |
| MOL004864 | 5,7-dihydroxy-3-(4-methoxyphenyl)-8-(3-methylbut-2-enyl)chromone | MAPK14 |
| MOL004864 | 5,7-dihydroxy-3-(4-methoxyphenyl)-8-(3-methylbut-2-enyl)chromone | NCOA2 |
| MOL004864 | 5,7-dihydroxy-3-(4-methoxyphenyl)-8-(3-methylbut-2-enyl)chromone | NOS2 |
| MOL004864 | 5,7-dihydroxy-3-(4-methoxyphenyl)-8-(3-methylbut-2-enyl)chromone | PPARG |
| MOL004864 | 5,7-dihydroxy-3-(4-methoxyphenyl)-8-(3-methylbut-2-enyl)chromone | PRSS1 |
| MOL004864 | 5,7-dihydroxy-3-(4-methoxyphenyl)-8-(3-methylbut-2-enyl)chromone | PTGS2 |
| MOL004864 | 5,7-dihydroxy-3-(4-methoxyphenyl)-8-(3-methylbut-2-enyl)chromone | TOP2A |
| MOL004866 | 2-(3,4-dihydroxyphenyl)-5,7-dihydroxy-6-(3-methylbut-2-enyl)chromone | ADRB2 |
| MOL004866 | 2-(3,4-dihydroxyphenyl)-5,7-dihydroxy-6-(3-methylbut-2-enyl)chromone | AR |
| MOL004866 | 2-(3,4-dihydroxyphenyl)-5,7-dihydroxy-6-(3-methylbut-2-enyl)chromone | CCNA2 |
| MOL004866 | 2-(3,4-dihydroxyphenyl)-5,7-dihydroxy-6-(3-methylbut-2-enyl)chromone | CHEK1 |
| MOL004866 | 2-(3,4-dihydroxyphenyl)-5,7-dihydroxy-6-(3-methylbut-2-enyl)chromone | DPP4 |
| MOL004866 | 2-(3,4-dihydroxyphenyl)-5,7-dihydroxy-6-(3-methylbut-2-enyl)chromone | F10 |
| MOL004866 | 2-(3,4-dihydroxyphenyl)-5,7-dihydroxy-6-(3-methylbut-2-enyl)chromone | F2 |
| MOL004866 | 2-(3,4-dihydroxyphenyl)-5,7-dihydroxy-6-(3-methylbut-2-enyl)chromone | F7 |
| MOL004866 | 2-(3,4-dihydroxyphenyl)-5,7-dihydroxy-6-(3-methylbut-2-enyl)chromone | HSP90AA |
| MOL004866 | 2-(3,4-dihydroxyphenyl)-5,7-dihydroxy-6-(3-methylbut-2-enyl)chromone | PPARG |
| MOL004866 | 2-(3,4-dihydroxyphenyl)-5,7-dihydroxy-6-(3-methylbut-2-enyl)chromone | PRSS1 |
| MOL004866 | 2-(3,4-dihydroxyphenyl)-5,7-dihydroxy-6-(3-methylbut-2-enyl)chromone | PTGS2 |
| MOL004866 | 2-(3,4-dihydroxyphenyl)-5,7-dihydroxy-6-(3-methylbut-2-enyl)chromone | SCN5A |
| MOL004879 | Glycyrin | AR |
| MOL004879 | Glycyrin | CHEK1 |
| MOL004879 | Glycyrin | DPP4 |
| MOL004879 | Glycyrin | ESR1 |
| MOL004879 | Glycyrin | ESR2 |
| MOL004879 | Glycyrin | F10 |
| MOL004879 | Glycyrin | F2 |
| MOL004879 | Glycyrin | KCNH2 |
| MOL004879 | Glycyrin | KDR |
| MOL004879 | Glycyrin | NCOA2 |
| MOL004879 | Glycyrin | NOS2 |
| MOL004879 | Glycyrin | PPARG |
| MOL004879 | Glycyrin | PRSS1 |
| MOL004879 | Glycyrin | PTGS2 |
| MOL004879 | Glycyrin | TOP2A |
| MOL004882 | Licocoumarone | AR |
| MOL004882 | Licocoumarone | CCNA2 |
| MOL004882 | Licocoumarone | ESR1 |
| MOL004882 | Licocoumarone | ESR2 |
| MOL004882 | Licocoumarone | GSK3B |
| MOL004882 | Licocoumarone | HSP90AA |
| MOL004883 | Licoisoflavone | AR |
| MOL004883 | Licoisoflavone | CCNA2 |
| MOL004883 | Licoisoflavone | CHEK1 |
| MOL004883 | Licoisoflavone | DPP4 |
| MOL004883 | Licoisoflavone | ESR1 |
| MOL004883 | Licoisoflavone | F10 |
| MOL004883 | Licoisoflavone | F2 |
| MOL004883 | Licoisoflavone | HSP90AA |
| MOL004883 | Licoisoflavone | KDR |
| MOL004883 | Licoisoflavone | MAPK14 |
| MOL004883 | Licoisoflavone | NCOA2 |
| MOL004883 | Licoisoflavone | NOS2 |
| MOL004883 | Licoisoflavone | PPARG |
| MOL004883 | Licoisoflavone | PRSS1 |
| MOL004883 | Licoisoflavone | PTGS2 |
| MOL004883 | Licoisoflavone | TOP2A |
| MOL004884 | Licoisoflavone B | ACHE |
| MOL004884 | Licoisoflavone B | AR |
| MOL004884 | Licoisoflavone B | CCNA2 |
| MOL004884 | Licoisoflavone B | CHEK1 |
| MOL004884 | Licoisoflavone B | ESR1 |
| MOL004884 | Licoisoflavone B | ESR2 |
| MOL004884 | Licoisoflavone B | F10 |
| MOL004884 | Licoisoflavone B | F2 |
| MOL004884 | Licoisoflavone B | GSK3B |
| MOL004884 | Licoisoflavone B | NOS2 |
| MOL004884 | Licoisoflavone B | PPARG |
| MOL004884 | Licoisoflavone B | PRSS1 |
| MOL004884 | Licoisoflavone B | PTGS2 |
| MOL004884 | Licoisoflavone B | TOP2A |
| MOL004885 | licoisoflavanone | ACHE |
| MOL004885 | licoisoflavanone | AR |
| MOL004885 | licoisoflavanone | CCNA2 |
| MOL004885 | licoisoflavanone | ESR1 |
| MOL004885 | licoisoflavanone | ESR2 |
| MOL004885 | licoisoflavanone | F10 |
| MOL004885 | licoisoflavanone | F7 |
| MOL004885 | licoisoflavanone | GSK3B |
| MOL004885 | licoisoflavanone | HSP90AA |
| MOL004885 | licoisoflavanone | NCOA1 |
| MOL004885 | licoisoflavanone | NOS2 |
| MOL004885 | licoisoflavanone | PPARG |
| MOL004885 | licoisoflavanone | PRSS1 |
| MOL004885 | licoisoflavanone | PTGS1 |
| MOL004885 | licoisoflavanone | PTGS2 |
| MOL004885 | licoisoflavanone | SCN5A |
| MOL004885 | licoisoflavanone | TOP2A |
| MOL004891 | shinpterocarpin | ADRA1B |
| MOL004891 | shinpterocarpin | ADRA1D |
| MOL004891 | shinpterocarpin | ADRB2 |
| MOL004891 | shinpterocarpin | AR |
| MOL004891 | shinpterocarpin | CCNA2 |
| MOL004891 | shinpterocarpin | CHRM1 |
| MOL004891 | shinpterocarpin | CHRM3 |
| MOL004891 | shinpterocarpin | CHRNA7 |
| MOL004891 | shinpterocarpin | ESR1 |
| MOL004891 | shinpterocarpin | ESR2 |
| MOL004891 | shinpterocarpin | GSK3B |
| MOL004891 | shinpterocarpin | KCNH2 |
| MOL004891 | shinpterocarpin | MAPK14 |
| MOL004891 | shinpterocarpin | NCOA1 |
| MOL004891 | shinpterocarpin | NOS2 |
| MOL004891 | shinpterocarpin | OPRD1 |
| MOL004891 | shinpterocarpin | OPRM1 |
| MOL004891 | shinpterocarpin | PPARG |
| MOL004891 | shinpterocarpin | PRKACA |
| MOL004891 | shinpterocarpin | PRSS1 |
| MOL004891 | shinpterocarpin | PTGS1 |
| MOL004891 | shinpterocarpin | PTGS2 |
| MOL004891 | shinpterocarpin | RXRA |
| MOL004891 | shinpterocarpin | SCN5A |
| MOL004891 | shinpterocarpin | UBE2O |
| MOL004898 | (E)-3-[3,4-dihydroxy-5-(3-methylbut-2-enyl)phenyl]-1-(2,4-dihydroxyphenyl)prop-2-en-1-one | AR |
| MOL004898 | (E)-3-[3,4-dihydroxy-5-(3-methylbut-2-enyl)phenyl]-1-(2,4-dihydroxyphenyl)prop-2-en-1-one | CCNA2 |
| MOL004898 | (E)-3-[3,4-dihydroxy-5-(3-methylbut-2-enyl)phenyl]-1-(2,4-dihydroxyphenyl)prop-2-en-1-one | ESR1 |
| MOL004898 | (E)-3-[3,4-dihydroxy-5-(3-methylbut-2-enyl)phenyl]-1-(2,4-dihydroxyphenyl)prop-2-en-1-one | GSK3B |
| MOL004898 | (E)-3-[3,4-dihydroxy-5-(3-methylbut-2-enyl)phenyl]-1-(2,4-dihydroxyphenyl)prop-2-en-1-one | HSP90AA |
| MOL004898 | (E)-3-[3,4-dihydroxy-5-(3-methylbut-2-enyl)phenyl]-1-(2,4-dihydroxyphenyl)prop-2-en-1-one | MAPK14 |
| MOL004898 | (E)-3-[3,4-dihydroxy-5-(3-methylbut-2-enyl)phenyl]-1-(2,4-dihydroxyphenyl)prop-2-en-1-one | NCOA2 |
| MOL004898 | (E)-3-[3,4-dihydroxy-5-(3-methylbut-2-enyl)phenyl]-1-(2,4-dihydroxyphenyl)prop-2-en-1-one | PPARG |
| MOL004898 | (E)-3-[3,4-dihydroxy-5-(3-methylbut-2-enyl)phenyl]-1-(2,4-dihydroxyphenyl)prop-2-en-1-one | PTGS2 |
| MOL004903 | liquiritin | F10 |
| MOL004903 | liquiritin | F7 |
| MOL004903 | liquiritin | KDR |
| MOL004903 | liquiritin | PTGS2 |
| MOL004903 | liquiritin | SOD1 |
| MOL004904 | licopyranocoumarin | ACHE |
| MOL004904 | licopyranocoumarin | AR |
| MOL004904 | licopyranocoumarin | CCNA2 |
| MOL004904 | licopyranocoumarin | ESR1 |
| MOL004904 | licopyranocoumarin | F10 |
| MOL004904 | licopyranocoumarin | F2 |
| MOL004904 | licopyranocoumarin | F7 |
| MOL004904 | licopyranocoumarin | KDR |
| MOL004904 | licopyranocoumarin | NOS2 |
| MOL004904 | licopyranocoumarin | PPARG |
| MOL004904 | licopyranocoumarin | PRSS1 |
| MOL004904 | licopyranocoumarin | PTGS2 |
| MOL004904 | licopyranocoumarin | TOP2A |
| MOL004907 | Glyzaglabrin | AR |
| MOL004907 | Glyzaglabrin | CCNA2 |
| MOL004907 | Glyzaglabrin | CHEK1 |
| MOL004907 | Glyzaglabrin | DPP4 |
| MOL004907 | Glyzaglabrin | ESR1 |
| MOL004907 | Glyzaglabrin | ESR2 |
| MOL004907 | Glyzaglabrin | GSK3B |
| MOL004907 | Glyzaglabrin | HSP90AA |
| MOL004907 | Glyzaglabrin | MAPK14 |
| MOL004907 | Glyzaglabrin | NOS2 |
| MOL004907 | Glyzaglabrin | PPARG |
| MOL004907 | Glyzaglabrin | PRKACA |
| MOL004907 | Glyzaglabrin | PRSS1 |
| MOL004907 | Glyzaglabrin | PTGS1 |
| MOL004907 | Glyzaglabrin | PTGS2 |
| MOL004908 | Glabridin | ACHE |
| MOL004908 | Glabridin | ADRA1B |
| MOL004908 | Glabridin | ADRB2 |
| MOL004908 | Glabridin | AR |
| MOL004908 | Glabridin | CCNA2 |
| MOL004908 | Glabridin | CHEK1 |
| MOL004908 | Glabridin | CHRM1 |
| MOL004908 | Glabridin | ESR1 |
| MOL004908 | Glabridin | ESR2 |
| MOL004908 | Glabridin | GSK3B |
| MOL004908 | Glabridin | MAPK14 |
| MOL004908 | Glabridin | NCOA1 |
| MOL004908 | Glabridin | NCOA2 |
| MOL004908 | Glabridin | NOS2 |
| MOL004908 | Glabridin | PPARG |
| MOL004908 | Glabridin | PRKACA |
| MOL004908 | Glabridin | PRSS1 |
| MOL004908 | Glabridin | PTGS2 |
| MOL004908 | Glabridin | RXRA |
| MOL004908 | Glabridin | SCN5A |
| MOL004908 | Glabridin | UBE2O |
| MOL004910 | Glabranin | ESR1 |
| MOL004910 | Glabranin | F10 |
| MOL004910 | Glabranin | HSP90AA |
| MOL004910 | Glabranin | NOS2 |
| MOL004910 | Glabranin | PDE3A |
| MOL004910 | Glabranin | PRKACA |
| MOL004910 | Glabranin | PTGS1 |
| MOL004910 | Glabranin | PTGS2 |
| MOL004910 | Glabranin | SCN5A |
| MOL004911 | Glabrene | ADRB2 |
| MOL004911 | Glabrene | AR |
| MOL004911 | Glabrene | ESR1 |
| MOL004911 | Glabrene | ESR2 |
| MOL004911 | Glabrene | F10 |
| MOL004911 | Glabrene | GSK3B |
| MOL004911 | Glabrene | HSP90AA |
| MOL004911 | Glabrene | MAPK14 |
| MOL004911 | Glabrene | NCOA2 |
| MOL004911 | Glabrene | NOS2 |
| MOL004911 | Glabrene | PPARG |
| MOL004911 | Glabrene | PRSS1 |
| MOL004911 | Glabrene | PTGS1 |
| MOL004911 | Glabrene | PTGS2 |
| MOL004911 | Glabrene | RXRA |
| MOL004911 | Glabrene | SCN5A |
| MOL004912 | Glabrone | ACHE |
| MOL004912 | Glabrone | AR |
| MOL004912 | Glabrone | CCNA2 |
| MOL004912 | Glabrone | CHEK1 |
| MOL004912 | Glabrone | DPP4 |
| MOL004912 | Glabrone | ESR1 |
| MOL004912 | Glabrone | ESR2 |
| MOL004912 | Glabrone | F10 |
| MOL004912 | Glabrone | F2 |
| MOL004912 | Glabrone | GSK3B |
| MOL004912 | Glabrone | MAPK14 |
| MOL004912 | Glabrone | NOS2 |
| MOL004912 | Glabrone | PPARG |
| MOL004912 | Glabrone | PRSS1 |
| MOL004912 | Glabrone | PTGS1 |
| MOL004912 | Glabrone | PTGS2 |
| MOL004912 | Glabrone | RXRA |
| MOL004912 | Glabrone | SCN5A |
| MOL004913 | 1,3-dihydroxy-9-methoxy-6-benzofurano[3,2-c]chromenone | CCNA2 |
| MOL004913 | 1,3-dihydroxy-9-methoxy-6-benzofurano[3,2-c]chromenone | CHEK1 |
| MOL004913 | 1,3-dihydroxy-9-methoxy-6-benzofurano[3,2-c]chromenone | ESR1 |
| MOL004913 | 1,3-dihydroxy-9-methoxy-6-benzofurano[3,2-c]chromenone | ESR2 |
| MOL004913 | 1,3-dihydroxy-9-methoxy-6-benzofurano[3,2-c]chromenone | GSK3B |
| MOL004913 | 1,3-dihydroxy-9-methoxy-6-benzofurano[3,2-c]chromenone | HSP90AA |
| MOL004913 | 1,3-dihydroxy-9-methoxy-6-benzofurano[3,2-c]chromenone | MAPK14 |
| MOL004913 | 1,3-dihydroxy-9-methoxy-6-benzofurano[3,2-c]chromenone | PPARG |
| MOL004913 | 1,3-dihydroxy-9-methoxy-6-benzofurano[3,2-c]chromenone | PRKACA |
| MOL004914 | 1,3-dihydroxy-8,9-dimethoxy-6-benzofurano[3,2-c]chromenone | AR |
| MOL004914 | 1,3-dihydroxy-8,9-dimethoxy-6-benzofurano[3,2-c]chromenone | CHEK1 |
| MOL004914 | 1,3-dihydroxy-8,9-dimethoxy-6-benzofurano[3,2-c]chromenone | ESR1 |
| MOL004914 | 1,3-dihydroxy-8,9-dimethoxy-6-benzofurano[3,2-c]chromenone | GSK3B |
| MOL004914 | 1,3-dihydroxy-8,9-dimethoxy-6-benzofurano[3,2-c]chromenone | HSP90AA |
| MOL004914 | 1,3-dihydroxy-8,9-dimethoxy-6-benzofurano[3,2-c]chromenone | MAPK14 |
| MOL004914 | 1,3-dihydroxy-8,9-dimethoxy-6-benzofurano[3,2-c]chromenone | PPARG |
| MOL004914 | 1,3-dihydroxy-8,9-dimethoxy-6-benzofurano[3,2-c]chromenone | PRKACA |
| MOL004915 | Eurycarpin A | AR |
| MOL004915 | Eurycarpin A | CCNA2 |
| MOL004915 | Eurycarpin A | CHEK1 |
| MOL004915 | Eurycarpin A | DPP4 |
| MOL004915 | Eurycarpin A | ESR1 |
| MOL004915 | Eurycarpin A | ESR2 |
| MOL004915 | Eurycarpin A | F10 |
| MOL004915 | Eurycarpin A | F2 |
| MOL004915 | Eurycarpin A | GSK3B |
| MOL004915 | Eurycarpin A | HSP90AA |
| MOL004915 | Eurycarpin A | MAPK14 |
| MOL004915 | Eurycarpin A | NOS2 |
| MOL004915 | Eurycarpin A | PPARG |
| MOL004915 | Eurycarpin A | PRSS1 |
| MOL004915 | Eurycarpin A | PTGS2 |
| MOL004915 | Eurycarpin A | SCN5A |
| MOL004924 | (-)-Medicocarpin | ACHE |
| MOL004924 | (-)-Medicocarpin | PTGS2 |
| MOL004935 | Sigmoidin-B | ESR1 |
| MOL004935 | Sigmoidin-B | F10 |
| MOL004935 | Sigmoidin-B | HSP90AA |
| MOL004935 | Sigmoidin-B | KDR |
| MOL004935 | Sigmoidin-B | PTGS2 |
| MOL004941 | (2R)-7-hydroxy-2-(4-hydroxyphenyl)chroman-4-one | ADRB2 |
| MOL004941 | (2R)-7-hydroxy-2-(4-hydroxyphenyl)chroman-4-one | ESR1 |
| MOL004941 | (2R)-7-hydroxy-2-(4-hydroxyphenyl)chroman-4-one | HSP90AA |
| MOL004941 | (2R)-7-hydroxy-2-(4-hydroxyphenyl)chroman-4-one | MAOB |
| MOL004941 | (2R)-7-hydroxy-2-(4-hydroxyphenyl)chroman-4-one | PDE3A |
| MOL004941 | (2R)-7-hydroxy-2-(4-hydroxyphenyl)chroman-4-one | PKIA |
| MOL004941 | (2R)-7-hydroxy-2-(4-hydroxyphenyl)chroman-4-one | PRKACA |
| MOL004941 | (2R)-7-hydroxy-2-(4-hydroxyphenyl)chroman-4-one | PTGS1 |
| MOL004941 | (2R)-7-hydroxy-2-(4-hydroxyphenyl)chroman-4-one | PTGS2 |
| MOL004941 | (2R)-7-hydroxy-2-(4-hydroxyphenyl)chroman-4-one | RXRA |
| MOL004941 | (2R)-7-hydroxy-2-(4-hydroxyphenyl)chroman-4-one | SLC6A4 |
| MOL004945 | (2S)-7-hydroxy-2-(4-hydroxyphenyl)-8-(3-methylbut-2-enyl)chroman-4-one | ADRA1B |
| MOL004945 | (2S)-7-hydroxy-2-(4-hydroxyphenyl)-8-(3-methylbut-2-enyl)chroman-4-one | ADRB2 |
| MOL004945 | (2S)-7-hydroxy-2-(4-hydroxyphenyl)-8-(3-methylbut-2-enyl)chroman-4-one | ESR1 |
| MOL004945 | (2S)-7-hydroxy-2-(4-hydroxyphenyl)-8-(3-methylbut-2-enyl)chroman-4-one | ESR2 |
| MOL004945 | (2S)-7-hydroxy-2-(4-hydroxyphenyl)-8-(3-methylbut-2-enyl)chroman-4-one | F10 |
| MOL004945 | (2S)-7-hydroxy-2-(4-hydroxyphenyl)-8-(3-methylbut-2-enyl)chroman-4-one | HSP90AA |
| MOL004945 | (2S)-7-hydroxy-2-(4-hydroxyphenyl)-8-(3-methylbut-2-enyl)chroman-4-one | NOS2 |
| MOL004945 | (2S)-7-hydroxy-2-(4-hydroxyphenyl)-8-(3-methylbut-2-enyl)chroman-4-one | PDE3A |
| MOL004945 | (2S)-7-hydroxy-2-(4-hydroxyphenyl)-8-(3-methylbut-2-enyl)chroman-4-one | PTGS1 |
| MOL004945 | (2S)-7-hydroxy-2-(4-hydroxyphenyl)-8-(3-methylbut-2-enyl)chroman-4-one | PTGS2 |
| MOL004945 | (2S)-7-hydroxy-2-(4-hydroxyphenyl)-8-(3-methylbut-2-enyl)chroman-4-one | SCN5A |
| MOL004948 | Isoglycyrol | AR |
| MOL004948 | Isoglycyrol | DPP4 |
| MOL004948 | Isoglycyrol | ESR1 |
| MOL004948 | Isoglycyrol | GSK3B |
| MOL004948 | Isoglycyrol | NOS2 |
| MOL004948 | Isoglycyrol | PTGS2 |
| MOL004949 | Isolicoflavonol | AR |
| MOL004949 | Isolicoflavonol | CCNA2 |
| MOL004949 | Isolicoflavonol | CYP19A1 |
| MOL004949 | Isolicoflavonol | ESR1 |
| MOL004949 | Isolicoflavonol | F10 |
| MOL004949 | Isolicoflavonol | F2 |
| MOL004949 | Isolicoflavonol | GSK3B |
| MOL004949 | Isolicoflavonol | HSP90AA |
| MOL004949 | Isolicoflavonol | NCOA2 |
| MOL004949 | Isolicoflavonol | NOS2 |
| MOL004949 | Isolicoflavonol | PPARG |
| MOL004949 | Isolicoflavonol | PRSS1 |
| MOL004949 | Isolicoflavonol | PTGS2 |
| MOL004957 | HMO | ADRB2 |
| MOL004957 | HMO | AR |
| MOL004957 | HMO | CCNA2 |
| MOL004957 | HMO | CHEK1 |
| MOL004957 | HMO | CHRM1 |
| MOL004957 | HMO | DPP4 |
| MOL004957 | HMO | ESR1 |
| MOL004957 | HMO | ESR2 |
| MOL004957 | HMO | GSK3B |
| MOL004957 | HMO | MAOB |
| MOL004957 | HMO | MAPK14 |
| MOL004957 | HMO | NOS2 |
| MOL004957 | HMO | PDE3A |
| MOL004957 | HMO | PKIA |
| MOL004957 | HMO | PPARG |
| MOL004957 | HMO | PRKACA |
| MOL004957 | HMO | PRSS1 |
| MOL004957 | HMO | PTGS1 |
| MOL004957 | HMO | PTGS2 |
| MOL004957 | HMO | RXRA |
| MOL004957 | HMO | SCN5A |
| MOL004957 | HMO | SLC6A3 |
| MOL004957 | HMO | SLC6A4 |
| MOL004959 | 1-Methoxyphaseollidin | ADRA1B |
| MOL004959 | 1-Methoxyphaseollidin | ADRA1D |
| MOL004959 | 1-Methoxyphaseollidin | ADRB2 |
| MOL004959 | 1-Methoxyphaseollidin | AR |
| MOL004959 | 1-Methoxyphaseollidin | CCNA2 |
| MOL004959 | 1-Methoxyphaseollidin | ESR1 |
| MOL004959 | 1-Methoxyphaseollidin | ESR2 |
| MOL004959 | 1-Methoxyphaseollidin | F10 |
| MOL004959 | 1-Methoxyphaseollidin | F2 |
| MOL004959 | 1-Methoxyphaseollidin | GSK3B |
| MOL004959 | 1-Methoxyphaseollidin | HSP90AA |
| MOL004959 | 1-Methoxyphaseollidin | KCNH2 |
| MOL004959 | 1-Methoxyphaseollidin | KDR |
| MOL004959 | 1-Methoxyphaseollidin | MAPK14 |
| MOL004959 | 1-Methoxyphaseollidin | NCOA1 |
| MOL004959 | 1-Methoxyphaseollidin | NCOA2 |
| MOL004959 | 1-Methoxyphaseollidin | NOS2 |
| MOL004959 | 1-Methoxyphaseollidin | PPARG |
| MOL004959 | 1-Methoxyphaseollidin | PRSS1 |
| MOL004959 | 1-Methoxyphaseollidin | PTGS1 |
| MOL004959 | 1-Methoxyphaseollidin | PTGS2 |
| MOL004959 | 1-Methoxyphaseollidin | RXRA |
| MOL004959 | 1-Methoxyphaseollidin | SCN5A |
| MOL004959 | 1-Methoxyphaseollidin | TOP2A |
| MOL004961 | Quercetin der. | AR |
| MOL004961 | Quercetin der. | DPP4 |
| MOL004961 | Quercetin der. | ESR1 |
| MOL004961 | Quercetin der. | ESR2 |
| MOL004961 | Quercetin der. | GSK3B |
| MOL004961 | Quercetin der. | HSP90AA |
| MOL004961 | Quercetin der. | MAPK14 |
| MOL004961 | Quercetin der. | NCOA2 |
| MOL004961 | Quercetin der. | NOS2 |
| MOL004961 | Quercetin der. | PPARG |
| MOL004961 | Quercetin der. | PRSS1 |
| MOL004961 | Quercetin der. | PTGS1 |
| MOL004961 | Quercetin der. | PTGS2 |
| MOL004961 | Quercetin der. | PTPN1 |
| MOL004961 | Quercetin der. | SCN5A |
| MOL004966 | 3'-Hydroxy-4'-O-Methylglabridin | ADRA1B |
| MOL004966 | 3'-Hydroxy-4'-O-Methylglabridin | ADRB2 |
| MOL004966 | 3'-Hydroxy-4'-O-Methylglabridin | AR |
| MOL004966 | 3'-Hydroxy-4'-O-Methylglabridin | CCNA2 |
| MOL004966 | 3'-Hydroxy-4'-O-Methylglabridin | CHEK1 |
| MOL004966 | 3'-Hydroxy-4'-O-Methylglabridin | ESR1 |
| MOL004966 | 3'-Hydroxy-4'-O-Methylglabridin | ESR2 |
| MOL004966 | 3'-Hydroxy-4'-O-Methylglabridin | F10 |
| MOL004966 | 3'-Hydroxy-4'-O-Methylglabridin | F7 |
| MOL004966 | 3'-Hydroxy-4'-O-Methylglabridin | GSK3B |
| MOL004966 | 3'-Hydroxy-4'-O-Methylglabridin | HSP90AA |
| MOL004966 | 3'-Hydroxy-4'-O-Methylglabridin | KCNH2 |
| MOL004966 | 3'-Hydroxy-4'-O-Methylglabridin | KDR |
| MOL004966 | 3'-Hydroxy-4'-O-Methylglabridin | MAPK14 |
| MOL004966 | 3'-Hydroxy-4'-O-Methylglabridin | NCOA1 |
| MOL004966 | 3'-Hydroxy-4'-O-Methylglabridin | NCOA2 |
| MOL004966 | 3'-Hydroxy-4'-O-Methylglabridin | NOS2 |
| MOL004966 | 3'-Hydroxy-4'-O-Methylglabridin | PPARG |
| MOL004966 | 3'-Hydroxy-4'-O-Methylglabridin | PRKACA |
| MOL004966 | 3'-Hydroxy-4'-O-Methylglabridin | PRSS1 |
| MOL004966 | 3'-Hydroxy-4'-O-Methylglabridin | PTGS1 |
| MOL004966 | 3'-Hydroxy-4'-O-Methylglabridin | PTGS2 |
| MOL004966 | 3'-Hydroxy-4'-O-Methylglabridin | SCN5A |
| MOL004966 | 3'-Hydroxy-4'-O-Methylglabridin | TOP2A |
| MOL004974 | 3'-Methoxyglabridin | ACHE |
| MOL004974 | 3'-Methoxyglabridin | ADRA1B |
| MOL004974 | 3'-Methoxyglabridin | ADRB2 |
| MOL004974 | 3'-Methoxyglabridin | AR |
| MOL004974 | 3'-Methoxyglabridin | CCNA2 |
| MOL004974 | 3'-Methoxyglabridin | CHEK1 |
| MOL004974 | 3'-Methoxyglabridin | ESR1 |
| MOL004974 | 3'-Methoxyglabridin | ESR2 |
| MOL004974 | 3'-Methoxyglabridin | F10 |
| MOL004974 | 3'-Methoxyglabridin | F7 |
| MOL004974 | 3'-Methoxyglabridin | GSK3B |
| MOL004974 | 3'-Methoxyglabridin | HSP90AA |
| MOL004974 | 3'-Methoxyglabridin | KCNH2 |
| MOL004974 | 3'-Methoxyglabridin | MAPK14 |
| MOL004974 | 3'-Methoxyglabridin | NCOA1 |
| MOL004974 | 3'-Methoxyglabridin | NCOA2 |
| MOL004974 | 3'-Methoxyglabridin | NOS2 |
| MOL004974 | 3'-Methoxyglabridin | PPARG |
| MOL004974 | 3'-Methoxyglabridin | PRSS1 |
| MOL004974 | 3'-Methoxyglabridin | PTGS1 |
| MOL004974 | 3'-Methoxyglabridin | PTGS2 |
| MOL004974 | 3'-Methoxyglabridin | RXRA |
| MOL004974 | 3'-Methoxyglabridin | SCN5A |
| MOL004974 | 3'-Methoxyglabridin | TOP2A |
| MOL004978 | 2-[(3R)-8,8-dimethyl-3,4-dihydro-2H-pyrano[6,5-f]chromen-3-yl]-5-methoxyphenol | ACHE |
| MOL004978 | 2-[(3R)-8,8-dimethyl-3,4-dihydro-2H-pyrano[6,5-f]chromen-3-yl]-5-methoxyphenol | ADRA1B |
| MOL004978 | 2-[(3R)-8,8-dimethyl-3,4-dihydro-2H-pyrano[6,5-f]chromen-3-yl]-5-methoxyphenol | ADRB2 |
| MOL004978 | 2-[(3R)-8,8-dimethyl-3,4-dihydro-2H-pyrano[6,5-f]chromen-3-yl]-5-methoxyphenol | AR |
| MOL004978 | 2-[(3R)-8,8-dimethyl-3,4-dihydro-2H-pyrano[6,5-f]chromen-3-yl]-5-methoxyphenol | CCNA2 |
| MOL004978 | 2-[(3R)-8,8-dimethyl-3,4-dihydro-2H-pyrano[6,5-f]chromen-3-yl]-5-methoxyphenol | CHEK1 |
| MOL004978 | 2-[(3R)-8,8-dimethyl-3,4-dihydro-2H-pyrano[6,5-f]chromen-3-yl]-5-methoxyphenol | CHRM1 |
| MOL004978 | 2-[(3R)-8,8-dimethyl-3,4-dihydro-2H-pyrano[6,5-f]chromen-3-yl]-5-methoxyphenol | CHRM3 |
| MOL004978 | 2-[(3R)-8,8-dimethyl-3,4-dihydro-2H-pyrano[6,5-f]chromen-3-yl]-5-methoxyphenol | ESR1 |
| MOL004978 | 2-[(3R)-8,8-dimethyl-3,4-dihydro-2H-pyrano[6,5-f]chromen-3-yl]-5-methoxyphenol | ESR2 |
| MOL004978 | 2-[(3R)-8,8-dimethyl-3,4-dihydro-2H-pyrano[6,5-f]chromen-3-yl]-5-methoxyphenol | F10 |
| MOL004978 | 2-[(3R)-8,8-dimethyl-3,4-dihydro-2H-pyrano[6,5-f]chromen-3-yl]-5-methoxyphenol | GSK3B |
| MOL004978 | 2-[(3R)-8,8-dimethyl-3,4-dihydro-2H-pyrano[6,5-f]chromen-3-yl]-5-methoxyphenol | KCNH2 |
| MOL004978 | 2-[(3R)-8,8-dimethyl-3,4-dihydro-2H-pyrano[6,5-f]chromen-3-yl]-5-methoxyphenol | MAPK14 |
| MOL004978 | 2-[(3R)-8,8-dimethyl-3,4-dihydro-2H-pyrano[6,5-f]chromen-3-yl]-5-methoxyphenol | NCOA1 |
| MOL004978 | 2-[(3R)-8,8-dimethyl-3,4-dihydro-2H-pyrano[6,5-f]chromen-3-yl]-5-methoxyphenol | NCOA2 |
| MOL004978 | 2-[(3R)-8,8-dimethyl-3,4-dihydro-2H-pyrano[6,5-f]chromen-3-yl]-5-methoxyphenol | NOS2 |
| MOL004978 | 2-[(3R)-8,8-dimethyl-3,4-dihydro-2H-pyrano[6,5-f]chromen-3-yl]-5-methoxyphenol | PPARG |
| MOL004978 | 2-[(3R)-8,8-dimethyl-3,4-dihydro-2H-pyrano[6,5-f]chromen-3-yl]-5-methoxyphenol | PRKACA |
| MOL004978 | 2-[(3R)-8,8-dimethyl-3,4-dihydro-2H-pyrano[6,5-f]chromen-3-yl]-5-methoxyphenol | PRSS1 |
| MOL004978 | 2-[(3R)-8,8-dimethyl-3,4-dihydro-2H-pyrano[6,5-f]chromen-3-yl]-5-methoxyphenol | PTGS1 |
| MOL004978 | 2-[(3R)-8,8-dimethyl-3,4-dihydro-2H-pyrano[6,5-f]chromen-3-yl]-5-methoxyphenol | PTGS2 |
| MOL004978 | 2-[(3R)-8,8-dimethyl-3,4-dihydro-2H-pyrano[6,5-f]chromen-3-yl]-5-methoxyphenol | RXRA |
| MOL004978 | 2-[(3R)-8,8-dimethyl-3,4-dihydro-2H-pyrano[6,5-f]chromen-3-yl]-5-methoxyphenol | SCN5A |
| MOL004978 | 2-[(3R)-8,8-dimethyl-3,4-dihydro-2H-pyrano[6,5-f]chromen-3-yl]-5-methoxyphenol | SLC6A3 |
| MOL004978 | 2-[(3R)-8,8-dimethyl-3,4-dihydro-2H-pyrano[6,5-f]chromen-3-yl]-5-methoxyphenol | UBE2O |
| MOL004980 | Inflacoumarin A | ADRB2 |
| MOL004980 | Inflacoumarin A | AR |
| MOL004980 | Inflacoumarin A | DPP4 |
| MOL004980 | Inflacoumarin A | ESR1 |
| MOL004980 | Inflacoumarin A | F10 |
| MOL004980 | Inflacoumarin A | F2 |
| MOL004980 | Inflacoumarin A | HSP90AA |
| MOL004980 | Inflacoumarin A | NCOA2 |
| MOL004980 | Inflacoumarin A | PPARG |
| MOL004980 | Inflacoumarin A | PRSS1 |
| MOL004980 | Inflacoumarin A | PTGS1 |
| MOL004980 | Inflacoumarin A | PTGS2 |
| MOL004980 | Inflacoumarin A | SCN5A |
| MOL004989 | 6-prenylated eriodictyol | ESR1 |
| MOL004989 | 6-prenylated eriodictyol | F10 |
| MOL004989 | 6-prenylated eriodictyol | F7 |
| MOL004989 | 6-prenylated eriodictyol | HSP90AA |
| MOL004989 | 6-prenylated eriodictyol | NOS2 |
| MOL004989 | 6-prenylated eriodictyol | PTGS2 |
| MOL004989 | 6-prenylated eriodictyol | SCN5A |
| MOL004990 | 7,2',4'-trihydroxy－5-methoxy-3－arylcoumarin | AR |
| MOL004990 | 7,2',4'-trihydroxy－5-methoxy-3－arylcoumarin | CHEK1 |
| MOL004990 | 7,2',4'-trihydroxy－5-methoxy-3－arylcoumarin | DPP4 |
| MOL004990 | 7,2',4'-trihydroxy－5-methoxy-3－arylcoumarin | ESR1 |
| MOL004990 | 7,2',4'-trihydroxy－5-methoxy-3－arylcoumarin | ESR2 |
| MOL004990 | 7,2',4'-trihydroxy－5-methoxy-3－arylcoumarin | GSK3B |
| MOL004990 | 7,2',4'-trihydroxy－5-methoxy-3－arylcoumarin | HSP90AA |
| MOL004990 | 7,2',4'-trihydroxy－5-methoxy-3－arylcoumarin | MAPK14 |
| MOL004990 | 7,2',4'-trihydroxy－5-methoxy-3－arylcoumarin | NOS2 |
| MOL004990 | 7,2',4'-trihydroxy－5-methoxy-3－arylcoumarin | PPARG |
| MOL004990 | 7,2',4'-trihydroxy－5-methoxy-3－arylcoumarin | PRKACA |
| MOL004990 | 7,2',4'-trihydroxy－5-methoxy-3－arylcoumarin | PTGS1 |
| MOL004990 | 7,2',4'-trihydroxy－5-methoxy-3－arylcoumarin | PTGS2 |
| MOL004991 | 7-Acetoxy-2-methylisoflavone | ACHE |
| MOL004991 | 7-Acetoxy-2-methylisoflavone | ADRA1B |
| MOL004991 | 7-Acetoxy-2-methylisoflavone | ADRA1D |
| MOL004991 | 7-Acetoxy-2-methylisoflavone | ADRB2 |
| MOL004991 | 7-Acetoxy-2-methylisoflavone | AR |
| MOL004991 | 7-Acetoxy-2-methylisoflavone | CHEK1 |
| MOL004991 | 7-Acetoxy-2-methylisoflavone | DPP4 |
| MOL004991 | 7-Acetoxy-2-methylisoflavone | ESR1 |
| MOL004991 | 7-Acetoxy-2-methylisoflavone | F2 |
| MOL004991 | 7-Acetoxy-2-methylisoflavone | GSK3B |
| MOL004991 | 7-Acetoxy-2-methylisoflavone | HSP90AA |
| MOL004991 | 7-Acetoxy-2-methylisoflavone | MAPK14 |
| MOL004991 | 7-Acetoxy-2-methylisoflavone | NCOA2 |
| MOL004991 | 7-Acetoxy-2-methylisoflavone | NOS2 |
| MOL004991 | 7-Acetoxy-2-methylisoflavone | PDE3A |
| MOL004991 | 7-Acetoxy-2-methylisoflavone | PPARG |
| MOL004991 | 7-Acetoxy-2-methylisoflavone | PRSS1 |
| MOL004991 | 7-Acetoxy-2-methylisoflavone | PTGS1 |
| MOL004991 | 7-Acetoxy-2-methylisoflavone | PTGS2 |
| MOL004991 | 7-Acetoxy-2-methylisoflavone | RXRA |
| MOL004991 | 7-Acetoxy-2-methylisoflavone | SCN5A |
| MOL004993 | 8-prenylated eriodictyol | ESR1 |
| MOL004993 | 8-prenylated eriodictyol | F10 |
| MOL004993 | 8-prenylated eriodictyol | F7 |
| MOL004993 | 8-prenylated eriodictyol | HSP90AA |
| MOL004993 | 8-prenylated eriodictyol | NCOA1 |
| MOL004993 | 8-prenylated eriodictyol | PTGS2 |
| MOL004993 | 8-prenylated eriodictyol | SCN5A |
| MOL005000 | Gancaonin G | AR |
| MOL005000 | Gancaonin G | CCNA2 |
| MOL005000 | Gancaonin G | CHEK1 |
| MOL005000 | Gancaonin G | DPP4 |
| MOL005000 | Gancaonin G | ESR1 |
| MOL005000 | Gancaonin G | ESR2 |
| MOL005000 | Gancaonin G | F10 |
| MOL005000 | Gancaonin G | F2 |
| MOL005000 | Gancaonin G | GSK3B |
| MOL005000 | Gancaonin G | HSP90AA |
| MOL005000 | Gancaonin G | MAPK14 |
| MOL005000 | Gancaonin G | NCOA2 |
| MOL005000 | Gancaonin G | NOS2 |
| MOL005000 | Gancaonin G | PPARG |
| MOL005000 | Gancaonin G | PRSS1 |
| MOL005000 | Gancaonin G | PTGS2 |
| MOL005000 | Gancaonin G | TOP2A |
| MOL005001 | Gancaonin H | AR |
| MOL005001 | Gancaonin H | CCNA2 |
| MOL005001 | Gancaonin H | ESR1 |
| MOL005001 | Gancaonin H | F10 |
| MOL005001 | Gancaonin H | HSP90AA |
| MOL005001 | Gancaonin H | KDR |
| MOL005001 | Gancaonin H | NCOA2 |
| MOL005001 | Gancaonin H | PRSS1 |
| MOL005001 | Gancaonin H | PTGS2 |
| MOL005001 | Gancaonin H | TOP2A |
| MOL005003 | Licoagrocarpin | ACHE |
| MOL005003 | Licoagrocarpin | ADRA1B |
| MOL005003 | Licoagrocarpin | ADRB2 |
| MOL005003 | Licoagrocarpin | AR |
| MOL005003 | Licoagrocarpin | CCNA2 |
| MOL005003 | Licoagrocarpin | CHRM1 |
| MOL005003 | Licoagrocarpin | CHRM3 |
| MOL005003 | Licoagrocarpin | CHRM5 |
| MOL005003 | Licoagrocarpin | ESR1 |
| MOL005003 | Licoagrocarpin | ESR2 |
| MOL005003 | Licoagrocarpin | F10 |
| MOL005003 | Licoagrocarpin | F2 |
| MOL005003 | Licoagrocarpin | GSK3B |
| MOL005003 | Licoagrocarpin | HSP90AA |
| MOL005003 | Licoagrocarpin | KCNH2 |
| MOL005003 | Licoagrocarpin | MAPK14 |
| MOL005003 | Licoagrocarpin | NCOA2 |
| MOL005003 | Licoagrocarpin | NOS2 |
| MOL005003 | Licoagrocarpin | PPARG |
| MOL005003 | Licoagrocarpin | PRSS1 |
| MOL005003 | Licoagrocarpin | PTGS1 |
| MOL005003 | Licoagrocarpin | PTGS2 |
| MOL005003 | Licoagrocarpin | RXRA |
| MOL005003 | Licoagrocarpin | SCN5A |
| MOL005003 | Licoagrocarpin | UBE2O |
| MOL005007 | Glyasperins M | ACHE |
| MOL005007 | Glyasperins M | AR |
| MOL005007 | Glyasperins M | CCNA2 |
| MOL005007 | Glyasperins M | ESR1 |
| MOL005007 | Glyasperins M | ESR2 |
| MOL005007 | Glyasperins M | F10 |
| MOL005007 | Glyasperins M | F7 |
| MOL005007 | Glyasperins M | GSK3B |
| MOL005007 | Glyasperins M | HSP90AA |
| MOL005007 | Glyasperins M | KCNH2 |
| MOL005007 | Glyasperins M | KDR |
| MOL005007 | Glyasperins M | NCOA1 |
| MOL005007 | Glyasperins M | NCOA2 |
| MOL005007 | Glyasperins M | NOS2 |
| MOL005007 | Glyasperins M | PPARD |
| MOL005007 | Glyasperins M | PPARG |
| MOL005007 | Glyasperins M | PRKACA |
| MOL005007 | Glyasperins M | PRSS1 |
| MOL005007 | Glyasperins M | PTGS1 |
| MOL005007 | Glyasperins M | PTGS2 |
| MOL005007 | Glyasperins M | SCN5A |
| MOL005007 | Glyasperins M | TOP2A |
| MOL005008 | Glycyrrhiza flavonol A | ACHE |
| MOL005008 | Glycyrrhiza flavonol A | AR |
| MOL005008 | Glycyrrhiza flavonol A | CCNA2 |
| MOL005008 | Glycyrrhiza flavonol A | DPP4 |
| MOL005008 | Glycyrrhiza flavonol A | ESR1 |
| MOL005008 | Glycyrrhiza flavonol A | ESR2 |
| MOL005008 | Glycyrrhiza flavonol A | F10 |
| MOL005008 | Glycyrrhiza flavonol A | F7 |
| MOL005008 | Glycyrrhiza flavonol A | GSK3B |
| MOL005008 | Glycyrrhiza flavonol A | HSP90AA |
| MOL005008 | Glycyrrhiza flavonol A | NOS2 |
| MOL005008 | Glycyrrhiza flavonol A | PRSS1 |
| MOL005008 | Glycyrrhiza flavonol A | PTGS2 |
| MOL005008 | Glycyrrhiza flavonol A | TOP2A |
| MOL005012 | Licoagroisoflavone | AR |
| MOL005012 | Licoagroisoflavone | CCNA2 |
| MOL005012 | Licoagroisoflavone | CHEK1 |
| MOL005012 | Licoagroisoflavone | DPP4 |
| MOL005012 | Licoagroisoflavone | ESR1 |
| MOL005012 | Licoagroisoflavone | ESR2 |
| MOL005012 | Licoagroisoflavone | F10 |
| MOL005012 | Licoagroisoflavone | F2 |
| MOL005012 | Licoagroisoflavone | GSK3B |
| MOL005012 | Licoagroisoflavone | MAPK14 |
| MOL005012 | Licoagroisoflavone | NOS2 |
| MOL005012 | Licoagroisoflavone | PPARG |
| MOL005012 | Licoagroisoflavone | PRSS1 |
| MOL005012 | Licoagroisoflavone | PTGS2 |
| MOL005012 | Licoagroisoflavone | SCN5A |
| MOL005016 | Odoratin | AR |
| MOL005016 | Odoratin | CCNA2 |
| MOL005016 | Odoratin | CHEK1 |
| MOL005016 | Odoratin | DPP4 |
| MOL005016 | Odoratin | ESR1 |
| MOL005016 | Odoratin | ESR2 |
| MOL005016 | Odoratin | GSK3B |
| MOL005016 | Odoratin | HSP90AA |
| MOL005016 | Odoratin | MAPK14 |
| MOL005016 | Odoratin | NCOA2 |
| MOL005016 | Odoratin | NOS2 |
| MOL005016 | Odoratin | PPARG |
| MOL005016 | Odoratin | PRSS1 |
| MOL005016 | Odoratin | PTGS1 |
| MOL005016 | Odoratin | PTGS2 |
| MOL005016 | Odoratin | RXRA |
| MOL005016 | Odoratin | SCN5A |
| MOL005017 | Phaseol | AR |
| MOL005017 | Phaseol | CCNA2 |
| MOL005017 | Phaseol | CHEK1 |
| MOL005017 | Phaseol | ESR1 |
| MOL005017 | Phaseol | F2 |
| MOL005017 | Phaseol | GSK3B |
| MOL005017 | Phaseol | HSP90AA |
| MOL005017 | Phaseol | KDR |
| MOL005017 | Phaseol | MAPK14 |
| MOL005017 | Phaseol | PPARG |
| MOL005017 | Phaseol | PRKACA |
| MOL005017 | Phaseol | PTGS2 |
| MOL005018 | Xambioona | ESR1 |
| MOL005018 | Xambioona | ESR2 |
| MOL005018 | Xambioona | F10 |
| MOL005018 | Xambioona | NCOA2 |
| MOL005018 | Xambioona | NOS2 |
| MOL005018 | Xambioona | PTGS2 |
| MOL005020 | dehydroglyasperins C | ADRB2 |
| MOL005020 | dehydroglyasperins C | AR |
| MOL005020 | dehydroglyasperins C | CCNA2 |
| MOL005020 | dehydroglyasperins C | CHEK1 |
| MOL005020 | dehydroglyasperins C | ESR1 |
| MOL005020 | dehydroglyasperins C | ESR2 |
| MOL005020 | dehydroglyasperins C | F10 |
| MOL005020 | dehydroglyasperins C | HSP90AA |
| MOL005020 | dehydroglyasperins C | MAPK14 |
| MOL005020 | dehydroglyasperins C | NCOA2 |
| MOL005020 | dehydroglyasperins C | NOS2 |
| MOL005020 | dehydroglyasperins C | PPARG |
| MOL005020 | dehydroglyasperins C | PRSS1 |
| MOL005020 | dehydroglyasperins C | PTGS2 |
| MOL005020 | dehydroglyasperins C | SCN5A |
| MOL005828 | nobiletin | AR |
| MOL005828 | nobiletin | BAX |
| MOL005828 | nobiletin | BCL2 |
| MOL005828 | nobiletin | CASP9 |
| MOL005828 | nobiletin | CD163 |
| MOL005828 | nobiletin | CHEK1 |
| MOL005828 | nobiletin | CREB1 |
| MOL005828 | nobiletin | DPP4 |
| MOL005828 | nobiletin | EPHB2 |
| MOL005828 | nobiletin | ESR1 |
| MOL005828 | nobiletin | ESR2 |
| MOL005828 | nobiletin | F10 |
| MOL005828 | nobiletin | F2 |
| MOL005828 | nobiletin | F7 |
| MOL005828 | nobiletin | GSK3B |
| MOL005828 | nobiletin | HSP90AA |
| MOL005828 | nobiletin | JUN |
| MOL005828 | nobiletin | KCNH2 |
| MOL005828 | nobiletin | MAPK8 |
| MOL005828 | nobiletin | MMP9 |
| MOL005828 | nobiletin | NCOA2 |
| MOL005828 | nobiletin | NOS2 |
| MOL005828 | nobiletin | PLA2G4A |
| MOL005828 | nobiletin | PPARG |
| MOL005828 | nobiletin | PRSS1 |
| MOL005828 | nobiletin | PTGS1 |
| MOL005828 | nobiletin | PTGS2 |
| MOL005828 | nobiletin | PTPN1 |
| MOL005828 | nobiletin | SCN5A |
| MOL005828 | nobiletin | TIMP1 |
| MOL005828 | nobiletin | TOP2A |
| MOL005828 | nobiletin | TP53 |
| MOL006992 | (2R,3R)-4-methoxyl-distylin | ESR1 |
| MOL006992 | (2R,3R)-4-methoxyl-distylin | HSP90AA |
| MOL006992 | (2R,3R)-4-methoxyl-distylin | NOS2 |
| MOL006992 | (2R,3R)-4-methoxyl-distylin | PTGS1 |
| MOL006992 | (2R,3R)-4-methoxyl-distylin | PTGS2 |
| MOL013187 | Cubebin | ADRB2 |
| MOL013187 | Cubebin | F10 |
| MOL013187 | Cubebin | HSP90AA |
| MOL013187 | Cubebin | PTGS1 |
| MOL013187 | Cubebin | PTGS2 |
| MOL013381 | Marmin | ADRB2 |
| MOL013381 | Marmin | CA2 |
| MOL013381 | Marmin | PDE3A |
| MOL013381 | Marmin | PTGS2 |

**Part 2 contains only Table S3.**

**Table S3 Targets of disease**

| Stable Angina Gene Symbol | | Unstable Angina Gene Symbol | |
| --- | --- | --- | --- |
| CaC | AURKB | CRP | GRIN2A |
| CRP | CDC20 | IL6 | GRIA2 |
| IL6 | AGO2 | ACE | SNCG |
| ACE | CCNT1 | F3 | MIR183 |
| NOS3 | GSTT1 | NOS3 | PSAP |
| F3 | INPP5D | PLAT | ADAM10 |
| ICAM1 | TLR3 | ICAM1 | PIK3CG |
| NPPB | PRKCE | TNNI3 | MAP2 |
| TNNI3 | FYN | NPPB | ETS2 |
| PLAT | ADRA1B | EDN1 | IGFBP3 |
| EDN1 | ATXN7 | APOA1 | PDP1 |
| ADIPOQ | FOXC2 | MB | UGT1A1 |
| INS | COX5A | KNG1 | HLA-DQB1 |
| KNG1 | CFP | VWF | FHIT |
| APOA1 | TRAF6 | INS | PPIA |
| MB | KIF11 | PIK3C2A | ACVRL1 |
| TNF | ABCA7 | ADIPOQ | FOXO1 |
| VWF | SPN | TNF | MAP2K1 |
| PIK3C2A | TET1 | SERPINC1 | CYP19A1 |
| APOE | UGT1A8 | FGA | INTS6 |
| F2 | MIRLET7A1 | SELP | NTRK2 |
| F7 | XRCC5 | APOB | LIMK1 |
| APOB | TNC | APOE | SLC6A3 |
| FGA | BLOC1S1 | IL10 | TSC1 |
| IL10 | AIRE | LPA | AQP4 |
| SERPINC1 | GNRHR | TNNT2 | MBP |
| LPA | KLKB1 | MIR126 | CRH |
| SELP | ADRA1D | HTR1B | PVALB |
| MPO | FST | F2 | H4-16 |
| HTR1B | GNS | PF4 | USF1 |
| MIR126 | IKZF1 | MMP9 | TNFRSF9 |
| MIR155 | DNM2 | MIR145 | FGFR3 |
| TNNT2 | TIE1 | MIR155 | PGK1 |
| PCSK9 | PLEC | SERPINE1 | NCOA3 |
| SERPINE1 | RARB | HBB | MTTP |
| MMP9 | CTNND1 | PCSK9 | CDK1 |
| TLR4 | NONO | VCAM1 | KIR3DL1 |
| MIR145 | PIK3C3 | P2RY12 | PAH |
| JAK2 | GSTO1 | TLR4 | FOSL1 |
| IFNG | ARNTL | MPO | TYK2 |
| NPPA | FCN3 | HBA1 | SETD2 |
| MIR17 | PDE7A | JAK2 | TOP1 |
| TP53 | UBE2E2 | ITGA2B | SERPINI1 |
| LDLR | FNDC5 | IL18 | PTGER4 |
| PF4 | ARG1 | F7 | CTSA |
| P2RY12 | BCL2L11 | LIPC | IGFBP2 |
| CCL2 | HTRA1 | CCL2 | TBK1 |
| VCAM1 | KLRK1 | APOA2 | PRKCD |
| VEGFA | HSPA9 | SERPINF2 | SOX2 |
| PTEN | SFPQ | VEGFA | HBS1L |
| IL1B | JUNB | CD40LG | OGDH |
| APOA2 | CAMKK2 | IFNG | PROC |
| LIPC | MAN2B1 | LDLR | KLF1 |
| ALB | UBE2E3 | NPPA | NCOA2 |
| TET2 | SLC31A1 | IL1B | FOXF1 |
| AGTR1 | TNXA | MMP3 | MIR135A1 |
| IL18 | FDX1 | AGTR1 | PLCG1 |
| MIR34A | MUC16 | PTEN | SP7 |
| SERPINF2 | HDAC6 | TP53 | MAP1B |
| CD40LG | PRKACA | NOS2 | AURKA |
| CXCL8 | CNGA3 | CX3CR1 | RB1 |
| ITGB3 | CASP6 | ITGB3 | TGFBI |
| IL2 | NPC1 | TTR | GH1 |
| RETN | MIR93 | TET2 | RARRES2 |
| MPL | ABCG2 | SELE | CYP2E1 |
| NOS2 | ANPEP | THBD | DKK1 |
| CST3 | CDC25C | PON1 | CHI3L1 |
| ITGA2B | CD9 | LOC107133510 | DBH |
| SELE | KLRB1 | CXCL8 | ADORA2A |
| MIR106B | CDKN1B | AGT | PROM1 |
| HBB | INHA | MPL | NAT2 |
| TERT | SPOP | LOC106099062 | VTN |
| TTR | KCNC3 | PPARG | CA2 |
| GATA4 | TREM1 | RETN | CSF1R |
| CTLA4 | PDPN | CST3 | PIK3R1 |
| AGT | RCAN1 | MIR140 | HSD11B1 |
| MMP3 | APLP2 | PAPPA | IL6R |
| FAS | RBP3 | HBA2 | WT1 |
| THBD | PNPLA3 | MIR106B | HSPA5 |
| HBA1 | AMBRA1 | F5 | TLR9 |
| PON1 | MCU | ITGA2 | AOC3 |
| MIR140 | SELENOS | OLR1 | DNTT |
| PPARG | HHEX | SERPINA1 | CYP7A1 |
| CX3CR1 | LYL1 | MMP1 | GRIN2B |
| MIR21 | KIF6 | PTX3 | GRIA1 |
| OLR1 | TREML1 | HMOX1 | GRM5 |
| F9 | CHRM3 | MIR21 | DLG4 |
| LMNA | MECOM | IL2 | CNTF |
| CD36 | TAL1 | LMNA | CTSL |
| HMOX1 | UCA1 | LPL | RARRES1 |
| PAPPA | MIR149 | ADAMTS13 | RYR1 |
| F5 | GNAT1 | MIR144 | RYR3 |
| PIK3CA | CLCN7 | FBN1 | SLAMF1 |
| MIR146A | AURKA | SAA4 | RAF1 |
| PLA2G7 | TCF7 | FCGR2A | ABCC1 |
| CELA2A | TCF7L1 | PLA2G7 | CCNA2 |
| MIR208A | GRM5 | BDNF | PML |
| LOC107133510 | GAB2 | FAS | HLA-C |
| KRAS | ITM2B | KCNJ5 | MIR149 |
| REN | REG1A | REN | FCN3 |
| CP | IFIH1 | HBG2 | MMP28 |
| LEP | MLLT1 | FGF2 | ROCK1 |
| PTPN11 | CD52 | ALB | TRIB3 |
| PTX3 | MAOA | CD36 | HLA-E |
| LOC106099062 | MCM2 | IL1RN | PCSK6 |
| SOD1 | NCF2 | LOC110006319 | ERVW-1 |
| APP | PROZ | TERT | SLC2A1 |
| FGF2 | SLC22A1 | GNB3 | EP300 |
| FBN1 | SUN2 | FABP3 | GC |
| BDNF | CPT2 | F9 | CYP1B1 |
| ITGA2 | PLS3 | RUNX1 | LMX1B |
| LDLRAP1 | GNAS | PPBP | ELAVL4 |
| MIR29A | GATA3 | PIK3CA | PARK7 |
| MIR182 | CDC6 | PECAM1 | PTHLH |
| FCGR2A | KRT10 | MIR34A | PPIG |
| ADAMTS13 | COL17A1 | LEP | C5 |
| PSEN1 | MIR27B | AHSP | IL9 |
| LOC110006319 | MECP2 | ABL1 | IL32 |
| MMP1 | NPR1 | ABCA1 | FTL |
| LPL | BCL11A | GLA | TREM2 |
| ESR1 | HLA-DQA1 | CYBA | ANG |
| CD4 | AIF1 | CETP | CBL |
| KCNJ5 | UBE2N | SOD1 | RPS6KB1 |
| RUNX1 | TAP2 | LCAT | IFNB1 |
| MT-ND1 | SERPIND1 | SMARCA4 | CPT2 |
| IL1RN | GYS1 | FGB | HELLS |
| SERPINA1 | OTX2 | CD14 | AKR1C3 |
| MAPT | KLF1 | CPB2 | MFGE8 |
| GP6 | MAF | MMP8 | TNFRSF14 |
| EGFR | TREM2 | MSR1 | ATP2B3 |
| GBA | GPBAR1 | GP6 | ZEB2 |
| GLA | MIR19A | HMGCR | IRF7 |
| MYH6 | EIF2S1 | LCN2 | SALL1 |
| PLAU | LAMA1 | MIR423 | MIR302D |
| ETV6 | ITGAE | CTLA4 | MIR367 |
| MIR144 | FCN2 | TEK | ERCC1 |
| ABCC6 | ATR | SELL | PRKCE |
| TGFB1 | ISG15 | COL4A1 | EPM2A |
| ABL1 | PCSK1 | MIR210 | CDK5 |
| HMGCR | PREP | CSF1 | LYN |
| LCN2 | HOXA9 | LTA | PTK2B |
| TEK | CEBPE | MIR223 | GFAP |
| GATA6 | UBE2D2 | MTHFR | AKR1B1 |
| PECAM1 | LYVE1 | SCN5A | KLK3 |
| TNFRSF11B | SRSF2 | PLA2G2A | EIF4EBP1 |
| ENPP1 | H3-3A | IL1R1 | NPR1 |
| IGF1 | CXCL11 | ADRB1 | MERTK |
| PPBP | RARRES2 | KRAS | AQP1 |
| KMT2A | EXTL3 | CX3CL1 | COL5A1 |
| GNB3 | SIRT6 | KMT2A | CDC25C |
| CETP | AKR1C3 | CP | CDX2 |
| AR | MIR31 | ENG | BNIP3 |
| PTPN22 | MIR451A | CCL5 | SPTB |
| CSF3 | MIR18A | HP | RHOD |
| MIR29B1 | CACNA1B | HIF1A | SNCA |
| LCAT | SNAI2 | SAA1 | BAD |
| SMARCA4 | FRMD4A | AR | CCNF |
| ABCA1 | NLRP12 | BCR | ANGPTL2 |
| ENG | PDPK1 | APOC3 | PRKAA2 |
| NBN | TGIF1 | HRAS | IKBKB |
| HRAS | MARK2 | TIMP1 | MAPK10 |
| FABP3 | C9orf72 | KCNJ11 | CTSD |
| SOD2 | RHOD | ADRB2 | ANPEP |
| SELL | NR1H3 | IL1A | ENO2 |
| CYBA | APOM | ESR1 | DSG2 |
| MIR223 | NANOG | EGFR | TRAF6 |
| F8 | NELFCD | GJA5 | POU5F1 |
| HFE | FLOT1 | NAMPT | ADRB3 |
| IFNA1 | CHRM2 | MMP2 | LIF |
| FGB | DRD4 | COL1A1 | EMD |
| TYR | OGG1 | PTPN11 | TSC2 |
| APOC3 | HPX | MIR15A | C1R |
| TNFRSF1A | TRIM21 | CAT | FKBP5 |
| MMP8 | GSN | NBN | CPS1 |
| IL1A | MIR181B1 | TNFRSF11B | SYNGAP1 |
| FOXP3 | MIR141 | FLT3 | DISC1 |
| FLT3 | MIR191 | HSPD1 | COL5A2 |
| KIT | TSC2 | MAPK1 | PLK1 |
| AHSP | DHCR24 | UQCRFS1 | PTS |
| USH2A | PRKCZ | BAX | FBXW7 |
| CD14 | P3H1 | CCND1 | RIPK3 |
| MAPK1 | DICER1 | TAF1 | SERPIND1 |
| IL1R1 | PTS | TGFB1 | LCK |
| TTN | ALOX12 | CSF3 | S100A8 |
| CPB2 | CDH3 | FOXP3 | TXNIP |
| HIF1A | MSTN | ELANE | FADS1 |
| MIR210 | HDAC4 | PPARA | PLA2G4A |
| ADRB1 | MTAP | CALCA | CDK6 |
| HNF4A | ASNS | SMAD4 | GNAS |
| LTA | ST14 | HLA-DPB1 | ALOX12 |
| TIMP1 | RAD21 | PTGS2 | HBE1 |
| SCN5A | USF1 | MYL4 | PRKACA |
| SMAD4 | PDS5A | MAPT | DCTN1 |
| CYP2C19 | SCNN1A | MIR499A | BAP1 |
| KMT2B | CS | GP1BA | LBR |
| NKX2-5 | TUSC3 | HSPA4 | FST |
| HBA2 | MT-CO2 | LOC106804612 | FURIN |
| ABCG5 | SFTPC | PLOD1 | MS4A1 |
| MMP2 | ABCA3 | NR3C2 | KLRD1 |
| PPARA | PHYH | ITIH4 | PLAG1 |
| PRTN3 | DNAJC6 | MYH7 | SIRT6 |
| MIR20A | ARR3 | TNFRSF1A | NGF |
| ADRB2 | MKS1 | CYP2C19 | PIK3CB |
| MIR320A | LYST | ANGPT2 | FANCC |
| SAA4 | DCN | PLG | GAL |
| TLR2 | DCK | HGF | CD68 |
| ELANE | ANK2 | CD40 | NANOG |
| BCR | SLC9A3 | CD4 | MT-CYB |
| MIR15A | SAT1 | ALDH2 | IRF1 |
| KCNQ1 | ANK1 | FGFR1 | RAB3IL1 |
| KCNJ11 | HBA-LCR | MT-ND1 | MORF4L2 |
| PLA2G2A | SERPINF1 | GATA1 | GRM1 |
| VDR | MCAM | F13A1 | ASS1 |
| A2M | MYOC | TLR2 | HTR2C |
| DOCK6 | GHSR | F12 | HNRNPK |
| CAT | CCKAR | BRCA2 | HNRNPA2B1 |
| BBS10 | ATF3 | PLAU | SLC4A4 |
| MIR107 | GHRH | PSEN1 | SERPINF1 |
| FGFR1 | MSBP1 | C1S | MTMR2 |
| NF1 | RAB5A | IL4 | COCH |
| TERC | FBXW7 | F11 | AGK |
| ALDH2 | GNAT2 | ABCC8 | LOXL1 |
| NAMPT | BEST1 | MLH1 | MMADHC |
| SETBP1 | ROM1 | ITGAM | MYOC |
| IL4 | MAP3K5 | MYBPC3 | SLC17A7 |
| CSF1 | DBN1 | CASP1 | NRXN3 |
| GJA5 | MARK3 | IL17A | MMAA |
| GATA1 | ARAF | ITGB2 | FKBP14 |
| DNMT3A | STAG2 | FGF1 | GTPBP1 |
| MSR1 | VPS35 | PGF | LMBRD1 |
| ABCG8 | GRPR | IGF1 | POU4F1 |
| CTNNB1 | IL15RA | MYH9 | DCAF17 |
| CCL5 | CLEC12A | PARP1 | BLOC1S1 |
| HGF | MRTFA | ATXN2 | HDAC2 |
| ASXL1 | FADS1 | AKT1 | CHUK |
| G6PD | MRAS | VDR | PSEN2 |
| PTGS2 | CD46 | GATA2 | LEF1 |
| CCND1 | RECK | G6PD | PINK1 |
| HSPA4 | CSNK1A1 | PITX2 | PNLIP |
| INSR | BAG3 | F10 | NEDD4 |
| NBEAL2 | PRPS1 | ADORA1 | BCL6 |
| AKT1 | GSTO2 | PTGIS | MAOB |
| MTHFR | FGF19 | GCK | EGR1 |
| PTGDS | CTNNA3 | LTF | TJP1 |
| LOC106560211 | STH | PKD1 | MT-ATP6 |
| PRF1 | MIR184 | ALOX5AP | CPOX |
| PRKN | MIR181C | FMR1 | TUBB |
| LDLR-AS1 | BACE1-AS | HTT | CACNA1A |
| ATHS | BAP1 | TNNT1 | MYCN |
| APLN | DUSP2 | IL1RAPL2 | STUB1 |
| MIR423 | STAP1 | LPXN | CDH2 |
| CYCS | TRPC3 | INSR | CSNK2A1 |
| HSPD1 | BRD4 | HFE | DYRK1A |
| BRCA1 | CTSA | MIR122 | BMPR1A |
| IRS1 | PENK | MT-TL1 | EIF2AK3 |
| TNFSF4 | WAS | SLC6A2 | SLC1A2 |
| ADORA1 | FHL2 | PMM2 | MAPK9 |
| RBP4 | DPYD | TNFSF4 | FANCA |
| SERPINA3 | ACD | SULT1A3 | SCD |
| ABCC8 | FSHR | MIR142 | FADD |
| MIR331 | STK11 | APP | ALDH7A1 |
| ELN | ACTN2 | MIR10A | FOLH1 |
| BBS12 | HPR | LRP6 | BIRC2 |
| F10 | SRSF6 | PTGDS | ADIPOR1 |
| TF | SERPINB1 | CR1 | DCK |
| BAX | PAH | F8 | HAVCR2 |
| CXCL12 | ENO1 | CKM | CEBPB |
| HP | ERN1 | PRF1 | NTF3 |
| CX3CL1 | KIF5B | PKP2 | GCKR |
| CDH1 | LRPAP1 | TYR | CXCR5 |
| MIR27A | VDAC2 | ZFHX3 | RECK |
| STAT3 | ATP5F1B | GP5 | RHD |
| GCK | CACNA2D1 | ELN | RUNX1T1 |
| CAV1 | RLBP1 | CDKN2A | CD58 |
| CYP3A5 | TUBA3D | DNMT3A | CDC25A |
| ITGAM | TP73 | TGFBR2 | NPR2 |
| NFKB1 | NCOR1 | CDH1 | SPTA1 |
| TNFRSF1B | HABP2 | HLA-B | YY1 |
| ABCA4 | TOP2A | GNAQ | HNRNPA1 |
| CD8A | FANCA | APLN | NEFH |
| MIR328 | ATP7A | IDH1 | POLG |
| FGFR2 | ABCC3 | BRCA1 | FUS |
| ALOX5AP | ULK1 | MIR132 | NDUFV2 |
| BRCA2 | GPC1 | COL1A2 | CHMP2B |
| GFI1B | TOMM40 | GSR | NDUFS4 |
| SLC6A2 | ADRA1A | NFKB1 | UBQLN2 |
| HLA-DPB1 | EDN3 | NAGLU | SLC39A4 |
| IL17A | SMS | MEN1 | LIPT1 |
| IL1RAPL2 | RNF14 | ATM | CHCHD10 |
| F11 | DHRS9 | IL12A | PRKDC |
| TAF1 | LUC7L | CD34 | TOP2A |
| PGF | GYPE | IFNA1 | EEF1A1 |
| MYH7 | SLC46A1 | FGF21 | MUC1 |
| COL1A1 | DMD | ANGPT1 | FERMT3 |
| GNAQ | PTN | HNF1A | TREML1 |
| IDH1 | TRIB1 | RBP4 | RPS19 |
| ABCB1 | FASN | IDUA | PKLR |
| ANGPT2 | GSK3A | WFS1 | NPR3 |
| PIGA | RB1CC1 | HTR2A | EPX |
| HLA-B | STMN1 | HSPA1A | TUBA1B |
| CALCA | HBD | IDH2 | ANXA2 |
| MIR221 | PDK4 | CYCS | PNMT |
| MIR203A | DCX | ACTG1 | IL12B |
| KCNJ2 | TRAF1 | APOH | IGFBP5 |
| SAA1 | HAPLN1 | H2AC18 | NES |
| CYP27A1 | FOLR2 | SMC1A | ATP13A2 |
| CSF2 | MIR106A | MIR208B | FGF4 |
| CASP1 | PTCH1 | MIR16-1 | IL34 |
| GSR | C3AR1 | ENTPD1 | PRKCA |
| ATM | MIRLET7I | SLC4A1 | DNMT3B |
| CR1 | RAB11A | PAX6 | NDE1 |
| LRP6 | GP9 | LOC106804613 | MT-CO1 |
| CD40 | NRGN | CTF1 | TYRO3 |
| NR3C2 | IRF8 | NF1 | HRG |
| MEFV | NEDD4 | SOD3 | MIR1-1 |
| F13A1 | AFF1 | GPT | FUCA1 |
| IL2RA | TAC3 | COL3A1 | ACAN |
| MIR499A | GLB1 | NPM1 | FLNC |
| RYR2 | KCNJ3 | AKT2 | CDC42 |
| NOTCH3 | PITX1 | PAX5 | ODC1 |
| MYH9 | ASIC1 | KCNA5 | JUP |
| SLC2A4 | FOXA1 | MIR378A | EPHX1 |
| MIR143 | GFI1 | HMGB1 | PROS1 |
| NPM1 | CDK5R1 | PRKN | RXRB |
| HNF1A | PPP3R1 | FLT4 | NCSTN |
| MYBPC3 | PPID | CCL18 | CRKL |
| TXN | TIMELESS | GDF15 | NPC1 |
| MIR222 | STUB1 | MIR11401 | OGT |
| PTGIS | NCOR2 | MIR11399 | GSTO1 |
| CASP3 | TBL2 | MIR11400 | TCN2 |
| CDKN2A | BTK | KMT2B | NCOR1 |
| CYP3A4 | FTO | CBFB | CCNA1 |
| XYLT2 | THY1 | DSP | KRIT1 |
| ACTC1 | CXCR5 | COL2A1 | IRS4 |
| FABP4 | CD58 | CYP3A5 | NCR1 |
| PKD1 | ELL | KDR | MIR330 |
| HNF1B | PMS2 | PTPN1 | ERCC4 |
| CD34 | FECH | SDHB | OPRM1 |
| MIR122 | UQCRC1 | TCF7L2 | CEL |
| EPO | AHR | HNF4A | PRG2 |
| ANGPT1 | KCNB1 | TXN | SELENOS |
| MTHFD1 | GOT2 | CD86 | ERBB3 |
| ZMPSTE24 | APPL1 | CHEK2 | HDAC1 |
| PMM2 | DHODH | CXCL12 | ETS1 |
| PLOD1 | MYLK3 | ANXA5 | LIPE |
| WFS1 | MIR26A2 | ADAMTS4 | BRIP1 |
| ITGB2 | PIK3R2 | EDNRB | DMD |
| AGER | ADIPOR1 | CDKN2B-AS1 | CFI |
| MT-TL1 | SOST | PRNP | CCNB1 |
| IDH2 | ID4 | DPP4 | CDK9 |
| ACTB | GRIN2D | SERPINA3 | XRCC6 |
| CBL | ABCC4 | LRRK2 | SLC19A1 |
| UNC5C | PIK3R4 | CEBPA | AFP |
| MIR298 | DPYSL2 | GYPA | NGFR |
| HFE-AS1 | EEA1 | SPINK1 | TP73 |
| SCN1B | UBQLN1 | MIR181A2 | KCNA2 |
| KCNA5 | RYR3 | HLA-DRB1 | CALM1 |
| SULT1A3 | ATP5F1C | GNB1 | TFAP2B |
| MIR10A | CCN1 | PSMA6 | PSENEN |
| APOH | FLNC | EPO | SKI |
| F12 | RDH5 | CYP2D6 | GSTO2 |
| LTF | PRPH2 | CXCL10 | FANCG |
| PKP2 | NT5E | PKD2 | ACP1 |
| TBP | ATP2A2 | MSH2 | UBB |
| LAMP2 | AKT3 | IGFBP1 | NRF1 |
| GPT | BMPR1A | MIR2861 | AREG |
| HTR2A | FBLN5 | MTHFD1 | NCL |
| KCNE1 | IL3RA | BMP6 | SFRP1 |
| ZFHX3 | BGN | LAMP2 | KLK6 |
| C4A | RHD | MDM2 | BPI |
| KDR | MIRLET7B | CSF2 | CHM |
| KCNE2 | SMAD1 | FGG | FANCB |
| EGF | GJC1 | GYPC | KIF6 |
| PAX5 | VPS29 | TAFAZZIN | SLC9A3R2 |
| TCF7L2 | CCL24 | CLCN1 | RHO |
| COL1A2 | ADK | FN1 | SUMF1 |
| SOD3 | GRIN1 | A2M | HTR1D |
| FGF1 | HTR2C | HMGCL | COX5A |
| ACTG1 | EYA4 | ALOX5 | ATP2A2 |
| NAGLU | PPIF | ABCA4 | ZAP70 |
| XYLT1 | ANXA7 | SNCAIP | PLCG2 |
| CBFB | MTHFD1L | SLC30A8 | RELA |
| GP5 | KLC1 | HBZ | HCK |
| RYR1 | RAB8A | ACKR1 | CAPN1 |
| PARP1 | BPTF | RHOA | FASN |
| MEN1 | PGK1 | CTNNB1 | NOTCH2 |
| BCHE | CRYAB | KLF6 | CASP6 |
| ADAMTS4 | SOX6 | TF | HLA-DRA |
| IRS2 | ARSA | SMAD3 | FES |
| IL12A | NCL | APOA5 | ALDH1A1 |
| LRP5 | CAPN2 | IL2RA | ABCC2 |
| MAPK8IP1 | COX4I1 | AGER | PNP |
| CEBPA | AXIN1 | FABP4 | PTK2 |
| MDM2 | ABCB4 | B2M | KDM1A |
| GATA2 | SCO1 | MSH6 | KIF11 |
| FGG | NEFH | MIR204 | ATF4 |
| CRLF2 | TGFBI | CDH5 | CFLAR |
| B2M | LINGO1 | MIR22 | AKR1C2 |
| CYP4F2 | MSRA | CYP3A4 | BIRC3 |
| PICALM | DKK4 | SLC2A9 | ATRX |
| IL3 | ATG13 | ATXN1 | XPO1 |
| IGFBP3 | FANCC | THPO | NRP1 |
| DRD2 | MC4R | COL17A1 | NME1 |
| MIAT | PDCD10 | MIR486-1 | KCND3 |
| CYP2C9 | PCA3 | ITGA3 | DSC2 |
| RNASE3 | GRIA3 | PDE5A | ACO1 |
| NR2F2 | WNT3 | BRAF | GIPR |
| IDUA | STX1B | MIR342 | FCER2 |
| SNCA | OPTC | IL11 | BAK1 |
| DPP4 | MATN1 | IL7 | PLIN1 |
| MIR16-1 | HTR7 | IL3 | REST |
| COL2A1 | DLST | SLC6A4 | NCOR2 |
| SDHB | ITPR2 | CD63 | APBB1 |
| PLG | NDUFV2 | SPP1 | KLK2 |
| ITIH4 | VDAC3 | COG2 | GPX3 |
| CD28 | RELN | MYD88 | GAP43 |
| LGALS2 | APOD | AMBP | CDA |
| XDH | MARK1 | NPY | APLNR |
| H2AC18 | NAE1 | TNFSF14 | STMN1 |
| PDE5A | TFCP2 | TBP | PPP1R12A |
| AKT2 | PHF6 | ZEB1 | NPC1L1 |
| PAX6 | GTPBP4 | SLC22A12 | ARNTL |
| RPL5 | NRBF2 | ATXN7 | CRTC2 |
| ALOX5 | MIR96 | MMP13 | SSB |
| LRRK2 | GAD1 | NLRP3 | MX1 |
| CXCL10 | ALDH7A1 | CNR1 | SRF |
| ABCC9 | ADIPOR2 | ATXN8OS | GIP |
| CDH5 | MEIS1 | MIR92A1 | PMAIP1 |
| MIR197 | HBE1 | RUNX2 | CCL26 |
| MIR23B | EVPL | MIR28 | NGB |
| NOD2 | MIR212 | MED12L | UGT1A8 |
| RHOA | MIR193A | NR3C1 | SCT |
| GRN | UBQLN2 | LGALS3 | H4C11 |
| FCGR2B | PUS3 | TIMP2 | CCL20 |
| LGALS3 | KLLN | STAT3 | IL27 |
| FLT4 | USF3 | MAD1L1 | MKI67 |
| GP1BA | LYZ | SDHD | XPA |
| ADD1 | CEL | USH2A | TAP2 |
| SMC1A | ADORA3 | KL | OXA1L |
| COG2 | GGCX | CHKA | IGFBP4 |
| FLI1 | MX1 | PON2 | CHEK1 |
| MYD88 | PDE3B | NR4A2 | TAP1 |
| SLPI | SLC1A3 | TRNT1 | CUL1 |
| MIR132 | SPTLC1 | PDYN | TIE1 |
| MIR486-1 | ADAM12 | CDK8 | NR1H3 |
| IFNA2 | BLVRA | MYH10 | TP53BP1 |
| GDF15 | NDUFA1 | BRF1 | TWIST2 |
| CDKN2B | CDON | LOC110806263 | LTA4H |
| ATXN2 | SLC20A1 | XDH | IFI27 |
| GYPC | OPTN | XRCC1 | CTCF |
| SPP1 | BLMH | THBS4 | CCNG1 |
| CREB1 | RTN3 | FGFR4 | UROS |
| POT1 | THOP1 | PRKCH | ADAM33 |
| ACKR1 | SIPA1 | TPM1 | NR1I2 |
| FN1 | INA | RPL5 | RAN |
| STAT4 | NCKAP1 | HMGA1 | SPG7 |
| PTPN1 | KCNIP3 | CISH | ICMT |
| AVP | RCVRN | COL4A2 | SYNE1 |
| PLN | CYP7B1 | RNASEL | AURKB |
| LPXN | ENHO | LPP | TPI1 |
| SLC6A4 | TUBB1 | TIRAP | KLF5 |
| NQO1 | MC3R | POLK | SNAI1 |
| PDX1 | GPM6B | MSMB | SKP2 |
| NTS | LOC105371049 | GYPB | COL10A1 |
| PCNA | SIRT2 | SEPTIN9 | LOC108663987 |
| NSD1 | PKM | MIR152 | EZR |
| NR4A2 | SRF | CDKN2B | CFD |
| CHEK2 | PRAME | PTGS1 | EPRS1 |
| SNCAIP | MIR34C | CASP3 | TNXA |
| ADH1C | MIR335 | TGFB2 | MIR92A2 |
| PROM1 | AQP2 | FABP1 | LINC01254 |
| LRP8 | AREG | AVP | ENSG00000284946 |
| TAFAZZIN | COL5A1 | CXCL13 | AIFM1 |
| MLLT10 | DKK2 | CCL3 | ATR |
| GAPDH | NRXN3 | GJA1 | USP7 |
| DSP | MAZ | S100A4 | OGG1 |
| COL4A1 | APBB2 | ABCB1 | CDC20 |
| EDNRB | FRAT1 | THBS1 | UBE2D2 |
| IL23R | PITRM1 | RPA1 | NEDD8 |
| CKM | GPR3 | MBL2 | LACTB |
| GZMB | ATG14 | PCNA | ELOC |
| MIR208B | WASHC5 | CYP2C9 | VIPR1 |
| ADM | WASHC4 | F2R | NAGA |
| GLUD2 | SOX2-OT | FABP12 | S100A1 |
| MAPK3 | LIPE | IL33 | H2AX |
| BRAF | PCBP2 | H19 | FCN2 |
| MIR378A | IDO1 | TNFSF11 | RPS6KA3 |
| ENTPD1 | CDC25A | CALR | HDAC4 |
| MIR92A1 | IREB2 | NQO1 | GSN |
| FGF21 | ACP3 | INTS11 | MAP3K5 |
| SLC34A1 | SH2B3 | GZMB | CD3G |
| ECE1 | CD22 | FGF23 | KAT5 |
| GYPA | SLC2A10 | LAG3 | ATP7A |
| MIR142 | TCL1A | ITLN1 | PPP2R2B |
| PITX2 | GCKR | CLU | RDH5 |
| CNR1 | FLVCR1 | GAPDH | SCO1 |
| HMGB1 | SUMO4 | WRN | CDC6 |
| TRB | PVT1 | ACADS | TGFA |
| CALCR | MIR154 | GSTP1 | E2F1 |
| ATP1B1 | TNFRSF21 | TGFBR1 | CCNE2 |
| COL11A2 | RDH11 | CASP9 | HLA-DMA |
| CLU | COL4A4 | ACHE | H2BC21 |
| CCR6 | DISC1 | RNASE3 | HLA-DRB3 |
| THPO | NFU1 | SOS1 | RMRP |
| FSCN2 | HPCAL1 | CCR6 | EPHA2 |
| SPINK1 | VSNL1 | MIR19B1 | ALK |
| PDLIM4 | BACE2 | IL2RB | PRKAR1A |
| MIR33A | IBTK | BMP4 | NTRK3 |
| LAG3 | LRRK1 | C4B | PTCH1 |
| TNNT1 | ADARB2 | MIF | CAMK2A |
| LRRC56 | MRC1 | APOA4 | AXIN2 |
| MAD1L1 | GTPBP1 | BDKRB2 | TP63 |
| XRCC1 | ISCA1 | APC | GCH1 |
| HSPA1A | CLSTN1 | TRB | CAMK2G |
| NRAS | MEPE | CCN2 | TUBB1 |
| MIPEP | FTH1 | MTR | PTGIR |
| AMBP | COL18A1 | C1QTNF1 | AXIN1 |
| MGP | MIR100 | BCHE | IL6ST |
| CDKN2B-AS1 | IFNGR1 | MIR181A1 | ALPP |
| MIR181A2 | ALDH1A1 | COMT | CRK |
| TTN-AS1 | MYCN | BGLAP | AICDA |
| GNB1 | ACO1 | BSG | BPGM |
| ANXA5 | SPTA1 | EXOSC10 | CHRNA7 |
| NPY | NGB | TFPI | UBE2L3 |
| H19 | TNFSF15 | HBEGF | PCBD1 |
| PDGFRB | C7 | SELPLG | ASPA |
| SRC | SLC9A3R2 | CCL11 | RASSF1 |
| MIR133B | GPX3 | NFKBIL1 | CAV3 |
| ACHE | CCNA1 | FASLG | RAD51C |
| SMAD6 | TRPC1 | IFNA2 | PALB2 |
| THBS1 | SNRNP70 | GJA4 | AGO2 |
| NLRP3 | LGALS9 | JUN | SCAP |
| HBZ | MAP2K2 | U2AF1 | RPGR |
| CPT1A | CASP2 | BCL2L1 | BLZF1 |
| BRF1 | NFATC1 | SOS2 | BIRC7 |
| PRKAG2 | SLC12A2 | SRC | SPPL2A |
| SMAD3 | CFI | JAK1 | WLS |
| CCL3 | AKR1C2 | SOCS1 | TRIM13 |
| PKD2 | ZBTB16 | CD163 | TUBA3D |
| SLC2A2 | PTPN2 | RPS27A | H4C14 |
| SLC4A1 | ATXN3 | TFRC | RAB40AL |
| WNT1 | PLIN1 | NPPC | ATXN8 |
| IGF2BP2 | SPTLC2 | CCR3 | EPHB4 |
| C1S | NR1D1 | MAPK14 | GSK3A |
| MIR2861 | GRIN2C | ARHGEF2 | CYP11B1 |
| IL33 | KCNJ10 | CTSB | WAS |
| NUP155 | PLAGL1 | IL13 | STK4 |
| NUP214 | LIAS | KITLG | SGK1 |
| TINF2 | APBA2 | GUSB | IDS |
| SOS1 | GALR2 | HABP2 | ITGB4 |
| BDKRB2 | CALML3 | CREB1 | CCNE1 |
| HMGA1 | XPNPEP1 | OPHN1 | NCF1 |
| MIR200B | RGR | ATXN10 | KAT2B |
| DDX41 | ASAH2 | HBD | GLS |
| GSTP1 | SLC17A7 | BBOX1 | CYP2B6 |
| GUCY2D | HKDC1 | PARN | CDH3 |
| MIR205 | SLC17A6 | CDKN3 | PIN1 |
| MT-ATP6 | RPGR | SOAT1 | MTAP |
| SBDS | COL25A1 | HDAC9 | COX4I1 |
| SH3GL1 | GP2 | DLC1 | LAT |
| HLA-DRB1 | LMBRD1 | TAC1 | ABCC4 |
| SLC12A4 | HMGCLL1 | SREBF1 | ALAD |
| ERAP1 | UBXN11 | CARD16 | EPHA3 |
| SCN3B | SAMD9L | CSTB | VAV1 |
| NEUROD1 | RALGPS2 | EDNRA | TGIF1 |
| SRD5A2 | GSAP | PLA2G6 | SIAH1 |
| TMEM43 | BCYRN1 | MIR133A1 | HSP90AB1 |
| VLDLR | PITX3 | MAPK3 | CDK5R1 |
| BBS1 | MIR449C | DRD2 | CAST |
| CDK8 | TYRO3 | PNOC | THBS2 |
| P2RX7 | PCK2 | EZH2 | GADD45A |
| TBC1D4 | ROCK2 | UCP2 | BMX |
| FRZB | SIRT3 | MALAT1 | GPR37 |
| IKZF3 | IGFBP7 | HBG1 | KCNN3 |
| MIR375 | GFPT1 | GDNF | CYP21A2 |
| LPP | ERG | PMS2 | CIITA |
| CCT7 | AZGP1 | NF2 | CYP2J2 |
| IL15 | SPI1 | SST | XRCC5 |
| TFRC | PPP1R12A | AGTR2 | PIAS1 |
| GPD2 | RING1 | PAWR | NTN1 |
| MTNR1B | SRA1 | PLA2G10 | LGALS1 |
| PAX4 | MIR25 | DICER1 | CD47 |
| MIR127 | KIR2DL2 | ATN1 | ENPP2 |
| CCR1 | KCNN4 | GPX1 | PLIN2 |
| APOA5 | RPS19 | POMC | SLC46A1 |
| IL2RB | LNPEP | MEF2C | SKIV2L |
| VKORC1 | SOCS2 | IGF1R | SFTPB |
| RBM8A | TCN2 | CPT1A | SH3GL2 |
| CTF1 | H6PD | MRE11 | SFTPC |
| NOTCH1 | KDM5A | CACNA1C | KCNC1 |
| RAC1 | BPI | GHRL | KDM5C |
| TNFAIP6 | TRDN | PLTP | FPGS |
| CYP2D6 | G6PC1 | TNFRSF1B | COMMD1 |
| PPP1R3A | ANG | ACE2 | DROSHA |
| SLC30A8 | CCL20 | NFE2L2 | DHPS |
| RTEL1 | CPOX | MIR483 | UBE2E3 |
| CEP85L | PROS1 | IGES | TSG101 |
| MIR29C | RXRB | MTOR | RB1CC1 |
| LOC106804613 | NPR2 | TNFRSF11A | UGT1A6 |
| KCNJ8 | KCND3 | TWIST1 | KMT2D |
| RNASEL | PTPRN | PDCD1 | ATOX1 |
| MYH10 | H4C11 | SGCB | F13B |
| HMGCL | TNFRSF14 | PDGFB | CCNC |
| EPHB2 | NUCB2 | PAFAH1B1 | CLPS |
| CRX | CLEC2B | CD247 | UGT1A10 |
| PSMA6 | IFI27 | CHIT1 | SRSF2 |
| CD63 | TRPC6 | TNFSF10 | PIAS3 |
| PDYN | BRIP1 | EGF | PSMC6 |
| ERBB2 | RBPJ | CFTR | DDX39B |
| PTGS1 | HSD11B2 | CTSK | TMEFF2 |
| IL7 | ATRX | CHGA | WDR77 |
| MIR22 | NPR3 | IGHE | STOM |
| BMP6 | ETS2 | MIR125A | MT3 |
| COL3A1 | FANCI | BMPR2 | CREB3 |
| EXOSC10 | KLK6 | SIRT1 | RAD1 |
| TGFBR2 | UGT2B17 | PRL | CLEC12A |
| MAPK14 | NOS1AP | MMP7 | MT-CO2 |
| CRB1 | NUP188 | CDK4 | H4C1 |
| SFTA3 | AFDN | MME | NOMO2 |
| NR3C1 | ID1 | CD69 | H4C6 |
| PRKG1 | TBK1 | PDGFRB | SPANXD |
| SERPINA12 | FES | SOCS3 | HBA-LCR |
| FGFR4 | APLNR | SMAD2 | CARM1 |
| APOA4 | KAT6A | CXCR4 | MUC16 |
| GUSB | CD70 | IGF2R | PPP3CA |
| GYPB | XIST | ERCC6 | ATP1A1 |
| STAT5A | CHRNA4 | MIR9-1 | HK1 |
| EIF4EBP1 | ATP2B3 | NHLRC1 | DDR2 |
| COL4A2 | ELOVL4 | VEGFC | PRKD1 |
| STAT1 | CHRNB2 | XIAP | PIM1 |
| KLF6 | SLC5A7 | IGF2 | ANXA1 |
| TOR1A | CLDN19 | PRKG1 | CHRNA4 |
| SDHD | CLDN16 | HSPG2 | CSNK1A1 |
| ATXN8OS | MEOX2 | HADHB | CHRM3 |
| FOLH1 | WNT8B | IL15 | TUBB3 |
| GCH1 | NRL | NOG | STK11 |
| CASP8 | PADI2 | GHR | MVK |
| MYC | KLK8 | BCL2 | MAPK7 |
| ESM1 | APLP1 | FLT1 | CYP2C8 |
| CCR5 | DOCK3 | HTRA1 | PRKCZ |
| MME | IMPG1 | TIAM1 | QDPR |
| SOS2 | VSX2 | RHOB | MYLK2 |
| SST | ZFP57 | F2RL1 | ATF6 |
| IGF2 | SEMA4G | NOS1 | FYN |
| JAK1 | APBA1 | SERPINA12 | AVPR2 |
| DAPK1 | SPPL2A | IL37 | GPX4 |
| NR2E3 | IMPG2 | MECP2 | KRT8 |
| YAP1 | BOLA3 | DHFR | KCNJ1 |
| BSG | RDH8 | KCNH2 | CD3D |
| CEP290 | LRRTM3 | FXN | CSK |
| MT-CYB | CH25H | NR1H2 | TNFAIP3 |
| MAPK7 | EYS | TARDBP | SLC18A2 |
| C1QTNF3 | NXNL1 | ANGPTL4 | ARAF |
| AGTR2 | RP1L1 | TH | ASNS |
| CXCR4 | PCDH11X | TSPO | CD81 |
| POMC | MARVELD3 | PDE4D | CCKAR |
| C1QTNF1 | EXOC3L2 | CDKN1A | ILK |
| DNMT1 | BHLHB9 | CTSS | MAP2K5 |
| IGF1R | CALHM1 | PON3 | HSD3B2 |
| GCG | TM2D1 | GAST | HSP90B1 |
| CCL18 | TMEM119 | MYC | CAMKK2 |
| GSTM1 | RAB7B | CXCR2 | FER |
| GHRL | C10orf55 | CTSG | EDN3 |
| AMPD1 | JCAD | C5AR1 | CD3E |
| PYGM | HYMAI | TRA | CCNH |
| CD86 | HAR1A | BACE1 | BMP1 |
| MKKS | HAR1B | ADAMTSL1 | C2 |
| MIR150 | SNHG3 | VCL | UBA1 |
| KL | MIR363 | MIR24-1 | TK1 |
| SORL1 | MIR511 | NRG1 | SORD |
| CISH | LINC01080 | HSPB1 | SPTLC1 |
| MED12L | LRP1-AS | LOC111365141 | PCSK1 |
| SIRT1 | LINC01772 | LIPG | SLC40A1 |
| RAF1 | LINC01616 | MIR33B | TNFRSF13B |
| TPM1 | AD5 | CREBBP | HRH1 |
| MSMB | AD8 | NFKBIA | MATK |
| TNFSF11 | AD9 | S100A9 | ERN1 |
| RPA1 | AD10 | NEAT1 | EIF2AK2 |
| CYP1A1 | AD11 | OXT | TNK2 |
| KITLG | AD12 | AHSG | RAG1 |
| ITLN1 | AD13 | BMP2 | SAT1 |
| PLD1 | AD14 | KLF4 | ADAM12 |
| SEPTIN9 | AD16 | UBC | FUT2 |
| THBS4 | AD17 | LEPR | CRAT |
| HBEGF | AD6 | CXCL16 | LRAT |
| ACSL4 | AD7 | FANCD2 | NDUFA1 |
| LOC110806263 | LMNB1 | LGALS3BP | EIF2S1 |
| CTNNA1 | MYF5 | HSP90AA1 | GLRX |
| BBS2 | SERPINB5 | C9orf72 | FADS2 |
| BBS7 | CCL19 | PPARGC1A | CPE |
| FARSB | IL4R | CYP1A1 | ATF3 |
| COL4A5 | PRKAB2 | BMP7 | TBX2 |
| RPS15 | TBX21 | CD274 | TICAM1 |
| CSNK1A1L | SNTA1 | VIP | SNAI2 |
| NCAM1 | FANCG | HLA-A | GRK4 |
| UQCRFS1 | CDA | TBXA2R | MCCC1 |
| CCL11 | PPP1R1B | AXL | LRPAP1 |
| IL13 | KMT2C | PRKCB | GGH |
| ANO2 | MIR125B1 | NR1H4 | CD9 |
| GCLC | MET | BIRC5 | CLCNKB |
| TIRAP | PRG2 | HPRT1 | DDAH1 |
| ELAC2 | BANF1 | TCF3 | DDIT4 |
| MXI1 | WNT3A | SERPING1 | ACD |
| RUNX2 | LRP2 | MTRR | ELK1 |
| CXCR3 | ACP5 | MUTYH | TBX1 |
| CASP9 | DSC2 | C3 | VPS35 |
| PSTPIP1 | SKI | CNTNAP2 | TTBK2 |
| MTM1 | PEA15 | RAD51 | WNK4 |
| GCLM | NRF1 | UCP1 | SOX6 |
| NODAL | FANCM | ATXN3 | PALLD |
| TRNT1 | CBFA2T3 | TYMS | SMAD7 |
| IGHG1 | KLRC1 | CXCR3 | NUDT1 |
| ADA | FLT3LG | EIF4E | RELN |
| U2AF1 | PMAIP1 | PPARD | APH1B |
| JAG1 | KLK10 | PTPA | CD209 |
| PRKCH | RBM15 | CCND2 | KLHL3 |
| ABCB6 | CBFA2T2 | FABP2 | MARK1 |
| PRMT7 | CHEK1 | HTR1A | HAPLN1 |
| POLK | MUC1 | PRODH | IGBP1 |
| MTX2 | TGFA | FOXC2 | JUNB |
| PACS1 | SKP2 | ERBB2 | NEUROG3 |
| ELP4 | INPPL1 | CD79A | GJC1 |
| UBAC2 | PCK1 | ADAMTS1 | E2F3 |
| HMCN1 | TAB2 | FLNA | ANK1 |
| DNAJC21 | SLC11A2 | CASR | ANKRD1 |
| MIR433 | ALAS2 | FBN2 | ALCAM |
| PALB2 | TRPS1 | GAS6 | CEBPE |
| RPE65 | MSX1 | BLK | CALM3 |
| SALL2 | FOXA2 | VASP | TERF1 |
| ANKRD26 | BCL10 | P2RY1 | RGS2 |
| DNAJC13 | AKAP9 | VCP | LYVE1 |
| EHBP1 | NPC1L1 | ADAMTS9 | NR2E3 |
| VPS13C | CA3 | CCL17 | MYLK3 |
| SLFN14 | BCL3 | PTPRC | IL15RA |
| MIR34B | GIP | LOX | HLA-DMB |
| MIR152 | NCR3 | NTRK1 | BTG1 |
| SELPLG | DYNLT1 | IAPP | BHLHE40 |
| PIK3CB | PPARGC1B | SREBF2 | ATG12 |
| PON2 | FENDRR | ORAI1 | TCF7L1 |
| MT-CO1 | MIR628 | G6PC2 | SRM |
| ACVR1 | KAT2B | SYP | NPC2 |
| CLCN1 | CIITA | TNC | RCC1 |
| DARS2 | CORO1B | TFAM | FUT1 |
| SLC13A5 | UGCG | ARSA | BCL2A1 |
| ARL11 | S100P | TBX4 | GFI1 |
| ERCC6L2 | LPIN1 | LOC109504728 | CLCA1 |
| GUCY1A1 | PKLR | GGT1 | CXCL11 |
| MIR449A | TGFBR3 | TRIB1 | BAG2 |
| MT-TK | AMACR | CCN1 | TERF2 |
| HYT3 | KCNA2 | IFNGR1 | ZBTB20 |
| ALL1 | DLK1 | TNFRSF4 | SSRP1 |
| LRP1 | ATP6AP2 | VKORC1 | RAD9A |
| C4B | IL12RB1 | MAPK8 | MCAM |
| IGFBP1 | TLR6 | IRF8 | KLK11 |
| BCL2 | TYROBP | RAD50 | TNNI3K |
| ADRA2C | STK39 | ADA | ACMSD |
| ITGAL | KLK4 | EPHX2 | METTL3 |
| ERCC6 | FANCE | FOS | FGF20 |
| TGFB2 | FANCF | PYGB | DHRS9 |
| GJA1 | CSRP3 | DDC | CENPA |
| LRAT | KCNIP2 | DES | BAIAP2L1 |
| CD69 | ECI2 | CYP4A11 | SPDEF |
| CNGB3 | IRS4 | PTPN6 | SFRP5 |
| SLC17A5 | CDKAL1 | ARNT | EN1 |
| CTSK | DAB2IP | CBS | SNX27 |
| HSPA5 | SRFBP1 | CCL19 | MUC5AC |
| HSP90AA1 | MIR330 | RUNX3 | EIF5B |
| NFE2L2 | SNHG5 | SELENOP | ZFPM1 |
| CALR | MIR29B2 | CCK | SUPT16H |
| ADCY10 | MIR186 | SQSTM1 | PIEZO1 |
| ITGA3 | H3-2 | BTK | RARS1 |
| FASLG | ANGPTL2 | DCN | H3-3A |
| MBL2 | ITGB4 | HBB-LCR | USP40 |
| F2R | RARS1 | OPTN | TFPT |
| GGT1 | ELK1 | TKT | KCTD13 |
| MTR | KCNK3 | SYK | CENPC |
| BGLAP | TRPV6 | LOC108663996 | CSN3 |
| CHI3L1 | CORIN | KRT18 | TAL2 |
| MIR199A1 | TCIRG1 | CAV1 | VEGFD |
| MMP13 | GFRA1 | MASP2 | CCDC62 |
| DNASE1 | BST1 | CRYAA | H3-3B |
| TIMP2 | CRTC2 | DIABLO | H3C3 |
| UTS2 | FLVCR2 | CDKN1B | H3C4 |
| CSNK1D | ROBO4 | DNMT1 | H4C2 |
| JUN | ARID5B | CRYAB | H4C8 |
| MT-ND5 | ONECUT1 | ARMS2 | H4C9 |
| MAP2 | ANO6 | MAOA | H4C3 |
| MTOR | FANCB | ACVR1 | H2AC4 |
| RS1 | ZRSR2 | PRKAA1 | MT-ATP8 |
| RDH12 | MIR30C1 | TMEM175 | H4C12 |
| HAMP | DANCR | XIST | H4C13 |
| MIF | MIR30D | ERBB4 | H4C5 |
| NPEPPS | MIR224 | PGR | H3C6 |
| SOAT1 | MIR532 | GSK3B | ENTR1 |
| IL5 | HNRNPA1 | FECH | INS-IGF2 |
| NPPC | HSP90AB1 | CDK2 | H3C15 |
| COCH | NUP153 | CXCL1 | H4C15 |
| MT-ND2 | SLC9A3R1 | C3AR1 | H4C4 |
| MYO7A | CRAT | SP1 | MIR148A |
| TSPO | CEACAM3 | MYB | CBSL |
| IAPP | CA2 | CD44 | MT-TT |
| STAT5B | TAP1 | HSPA8 | LOC105371049 |
| BCL2L1 | COL10A1 | MCL1 | MSBP1 |
| CFTR | RIPK3 | TPH1 | LOC106146143 |
| MT-ND6 | IL27 | ADCY10 | LOC106146144 |
| COMT | USP7 | ADORA3 | UGT2B7 |
| CCN2 | TNFAIP3 | CXCL9 | RIPK1 |
| ACE2 | HNRNPK | S100B | TNNI1 |
| PROCR | C1QBP | GAD1 | CYP1A2 |
| CYP19A1 | FUS | AHR | MLKL |
| MAPK8 | TERF2 | INTS2 | ADAMTS7 |
| FGF23 | H4C1 | MRAS | CLEC4A |
| SLC25A20 | VIPR1 | COL6A2 | UGT1A9 |
| ACTA2 | LMNB2 |  |  |
| MT-ND3 | M6PR |  |  |
| MIR23A | NAGA |  |  |
| PAFAH1B1 | SYNE1 |  |  |
| HSPG2 | SLC12A3 |  |  |
| ACADS | ARG2 |  |  |
| CCR3 | SLC19A2 |  |  |
| S100A9 | GIPR |  |  |
| VEGFC | TFAP2B |  |  |
| NCR1 | STEAP3 |  |  |
| FGFR3 | PNPLA2 |  |  |
| GFAP | HSD3B1 |  |  |
| SF3B1 | CCM2 |  |  |
| PDE6B | FOXF1 |  |  |
| KCNQ4 | HOXA10 |  |  |
| KCNH2 | LMO1 |  |  |
| UCP2 | BSCL2 |  |  |
| CTSD | KLK15 |  |  |
| S100A4 | CAPN10 |  |  |
| SP1 | HLF |  |  |
| TRA | GNPTAB |  |  |
| SYP | ADD2 |  |  |
| ELOC | DNAJC3 |  |  |
| SOCS3 | STEAP2 |  |  |
| KDM4C | THSD1 |  |  |
| SHBG | JAZF1 |  |  |
| TFPI | G6PC2 |  |  |
| TNFSF10 | CCL26 |  |  |
| ADAMTS14 | BAALC |  |  |
| IDE | TECRL |  |  |
| SCARB1 | ANGPTL8 |  |  |
| CHRNA7 | MIR10B |  |  |
| CYP2E1 | MIRLET7E | |  |
| TAC1 | MIR339 |  |  |
| IL11 | PCAT1 |  |  |
| CTSS | MIR338 |  |  |
| PDGFRA | MIR374A |  |  |
| CDK1 | CBR3-AS1 | |  |
| BCAM | ACTA2-AS1 | |  |
| SOCS1 | SLC22A2 |  |  |
| ARHGAP26 | AIFM1 |  |  |
| RARA | GPX4 |  |  |
| UGT1A1 | TPI1 |  |  |
| UBC | RASSF1 |  |  |
| HDAC9 | FERMT3 |  |  |
| GRK1 | NAT10 |  |  |
| PTPA | ITGA6 |  |  |
| C3 | MVK |  |  |
| ARHGEF12 | IRF3 |  |  |
| RB1 | HNRNPA2B1 | |  |
| INTS11 | POLG |  |  |
| JAK3 | SNAI1 |  |  |
| APOC1 | TERF1 |  |  |
| PRODH | H2BC21 |  |  |
| TH | CD209 |  |  |
| HBB-LCR | SYNE2 |  |  |
| ADAMTS5 | EIF4G1 |  |  |
| AGK | CFD |  |  |
| CD7 | YY1 |  |  |
| TNFSF14 | CSTB |  |  |
| BMP2 | RELB |  |  |
| FLT1 | RPS6 |  |  |
| AHSG | SMARCA5 | |  |
| CDKN3 | YBX1 |  |  |
| CTSB | AGA |  |  |
| FABP12 | ICMT |  |  |
| HADHB | SYNPO |  |  |
| FOXO1 | HK1 |  |  |
| GIGYF2 | CSK |  |  |
| EPOR | RNLS |  |  |
| FPR2 | SMARCA2 | |  |
| RAG1 | KAT5 |  |  |
| CDK4 | FHIT |  |  |
| CD163 | NDRG1 |  |  |
| VAV3 | SPTBN1 |  |  |
| GSK3B | RAD51C |  |  |
| TJP1 | CD82 |  |  |
| CSF1R | PCSK2 |  |  |
| FOS | H3C14 |  |  |
| LOC110973015 | TWIST2 |  |  |
| PDGFB | S100A1 |  |  |
| EDNRA | SUN1 |  |  |
| ITGB1 | ATP1A1 |  |  |
| CD19 | AVPR2 |  |  |
| MCF2L | EPCAM |  |  |
| SLC2A1 | ACLY |  |  |
| CA8 | BID |  |  |
| LMX1B | RAP1A |  |  |
| PER3 | CA9 |  |  |
| SORT1 | MIR92A2 |  |  |
| RHO | TSHR |  |  |
| MIR342 | MAP2K4 |  |  |
| DKK1 | DDX58 |  |  |
| PLAUR | VAV1 |  |  |
| NR1H2 | TG |  |  |
| CYBB | HLA-E |  |  |
| NTRK1 | ATOX1 |  |  |
| SLC9A1 | SCGB1A1 |  |  |
| IGHE | TRIM22 |  |  |
| VCL | EPHA2 |  |  |
| PNOC | PTH1R |  |  |
| TNFRSF8 | GLI1 |  |  |
| NFKBIA | PRLR |  |  |
| NOS1 | CD81 |  |  |
| GLUL | TK1 |  |  |
| NRG1 | CAST |  |  |
| EZH2 | DAXX |  |  |
| PIK3R1 | ETV4 |  |  |
| TLR1 | CXCR1 |  |  |
| SERPING1 | TRAP1 |  |  |
| GRB2 | NEDD8 |  |  |
| GH1 | CD24 |  |  |
| DENND11 | H4C14 |  |  |
| LOC100506071 | ITGA5 |  |  |
| CREBBP | CCNH |  |  |
| NAT2 | UBE2L3 |  |  |
| FCGR3B | DYNC1H1 | |  |
| TRIO | RAD9A |  |  |
| DLC1 | ADAM33 |  |  |
| MAPK10 | ERVW-1 |  |  |
| FADD | MIR340 |  |  |
| MAP2K1 | ERCC8 |  |  |
| KRT18 | NUDT1 |  |  |
| SMAD2 | PRKD1 |  |  |
| BBOX1 | CD3D |  |  |
| CCL17 | PCBD1 |  |  |
| APH1B | GP1BB |  |  |
| CMA1 | CALM3 |  |  |
| PTPRC | NUDC |  |  |
| TUBB | MLST8 |  |  |
| NF2 | SATB1 |  |  |
| HLA-C | LOC109504728 | |  |
| PRNP | EPHB4 |  |  |
| PARK7 | COMP |  |  |
| MYH11 | GADD45A | |  |
| VCP | NFATC2 |  |  |
| CTSG | ENPP2 |  |  |
| CACNA1A | FPGS |  |  |
| CD79A | TSG101 |  |  |
| SF3B2 | CCNC |  |  |
| ICOSLG | TSC22D3 |  |  |
| PINK1 | NCOA4 |  |  |
| LGALS3BP | VEGFD |  |  |
| FXN | H1-2 |  |  |
| ENO2 | SPG7 |  |  |
| CSNK2A1 | NISCH |  |  |
| EPHX2 | PIM1 |  |  |
| NR1I2 | AXIN2 |  |  |
| HLA-A | CD3G |  |  |
| MIR133A1 | TPT1 |  |  |
| CHGA | CPS1 |  |  |
| TGFBR1 | NPHS1 |  |  |
| MMP7 | RPS14 |  |  |
| BIRC5 | CCR8 |  |  |
| HBG2 | PDGFA |  |  |
| C5AR1 | DEK |  |  |
| APC | DDX39B |  |  |
| MLH1 | STARD13 |  |  |
| PGR | SRP72 |  |  |
| SREBF1 | PI3 |  |  |
| CCK | CSN3 |  |  |
| APAF1 | NUP43 |  |  |
| PPARGC1A | H4C9 |  |  |
| TUBB3 | GRM1 |  |  |
| OXT | GLS |  |  |
| TNFRSF11A | SOX9 |  |  |
| DHFR | FKBP5 |  |  |
| PLTP | UBA1 |  |  |
| CASR | DNAJC5 |  |  |
| CSF3R | TCAP |  |  |
| BMP4 | TGFB1I1 |  |  |
| GAS5 | RPS26 |  |  |
| SPARC | AIMP2 |  |  |
| TOP1 | BHLHE40 |  |  |
| PROC | BCL2A1 |  |  |
| TUBB4A | BLZF1 |  |  |
| BMI1 | BIRC7 |  |  |
| GAST | DEFB1 |  |  |
| CD247 | WDR77 |  |  |
| FABP1 | HARS1 |  |  |
| FABP2 | GOLGB1 |  |  |
| PDE4A | H4C3 |  |  |
| PSEN2 | H4C6 |  |  |
| CXCL13 | H4C12 |  |  |
| NFKBIL1 | SPHK1 |  |  |
| CD44 | FER |  |  |
| HSPA8 | EPHA3 |  |  |
| HDAC1 | TEC |  |  |
| GHR | CALCRL |  |  |
| DSG2 | NTN1 |  |  |
| CFH | NOTCH4 |  |  |
| GJA4 | HIRA |  |  |
| MYOD1 | LCT |  |  |
| HPRT1 | CCNG1 |  |  |
| GPX1 | NKX3-1 |  |  |
| SQSTM1 | SRM |  |  |
| MIR483 | NPHS2 |  |  |
| PPIG | KMT2D |  |  |
| SAG | FUT1 |  |  |
| MIR26B | BAG2 |  |  |
| RPS27A | SPTB |  |  |
| SET | SLC30A1 |  |  |
| EZR | RAD1 |  |  |
| CD38 | H4C8 |  |  |
| MIR195 | H4C13 |  |  |
| MARK4 | H4C5 |  |  |
| KLK3 | H3C15 |  |  |
| IGES | H4C4 |  |  |
| LEF1 | MIR146B |  |  |
| RUNX1T1 | IDS |  |  |
| SHC1 | CYP2C8 |  |  |
| CD5 | TPO |  |  |
| NCOA1 | RPL11 |  |  |
| BACE1 | RHEB |  |  |
| CHKA | MFN2 |  |  |
| ITGA4 | LTA4H |  |  |
| MIR28 | HYAL1 |  |  |
| RAD51 | LIFR |  |  |
| PLK1 | EEF1A2 |  |  |
| ARHGEF2 | DCTN1 |  |  |
| CCND3 | WNK1 |  |  |
| LOX | SIAH1 |  |  |
| RHOC | ABL2 |  |  |
| ANGPTL4 | TNK2 |  |  |
| CACNA1D | KLF5 |  |  |
| CDKN1A | STK24 |  |  |
| PPARD | PIAS1 |  |  |
| NCOA3 | RICTOR |  |  |
| NCSTN | DUOX2 |  |  |
| CD55 | KDM5C |  |  |
| ORAI1 | DOK1 |  |  |
| XIAP | LILRB1 |  |  |
| ALPP | E2F3 |  |  |
| PML | BAG1 |  |  |
| CTCF | ST3GAL4 |  |  |
| HCN4 | RPS5 |  |  |
| ADAMTS1 | MICB |  |  |
| CD80 | ATG12 |  |  |
| JUP | TPD52 |  |  |
| OGN | KLRD1 |  |  |
| S100A12 | INF2 |  |  |
| ABCG1 | FIBP |  |  |
| TIAM1 | CCNF |  |  |
| FTL | SORBS3 |  |  |
| CDH2 | RARRES1 |  |  |
| PDE4D | GH2 |  |  |
| TIMP3 | RCN1 |  |  |
| CHAT | MICA |  |  |
| IFNB1 | NSL1 |  |  |
| DES | PGAM5 |  |  |
| MALAT1 | H3-3B |  |  |
| ARHGEF1 | PRRC2A |  |  |
| S100B | H4C2 |  |  |
| MBP | H3C6 |  |  |
| NPY1R | H4C15 |  |  |
| MYB | H2BC13 |  |  |
| AKAP13 | CCAT2 |  |  |
| RHOB | ATP5F1E |  |  |
| GRIA2 | NTRK3 |  |  |
| CACNA1C | DDR2 |  |  |
| LAMP1 | ADAM9 |  |  |
| MIR590 | CD79B |  |  |
| ADCYAP1 | CDKN2C |  |  |
| ARHGAP1 | ALAD |  |  |
| TBXA2R | MS4A1 |  |  |
| CFB | SORD |  |  |
| VTN | PLA2G1B |  |  |
| FCGR3A | ASPA |  |  |
| MYO9B | DOT1L |  |  |
| AOC3 | UBE2A |  |  |
| ESR2 | SDC4 |  |  |
| CEBPB | PALLD |  |  |
| IL32 | SELENBP1 | |  |
| PRKCB | STX4 |  |  |
| CARD16 | RGS2 |  |  |
| LUC7L2 | HLA-DMB | |  |
| CIB1 | DUOX1 |  |  |
| TUBA1B | UBE2S |  |  |
| VAV2 | CEACAM6 | |  |
| PREX1 | SPINT1 |  |  |
| ITSN2 | PPY |  |  |
| ARHGEF17 | SCAP |  |  |
| NOX4 | PDLIM1 |  |  |
| GLUD1 | NIPBL |  |  |
| CR2 | METTL3 |  |  |
| SGCD | NEXN |  |  |
| ARHGEF6 | TOMM20 |  |  |
| OPHN1 | EIF5B |  |  |
| CCR4 | PRPF40A |  |  |
| CDC42 | NUP37 |  |  |
| PLA2G10 | SEPTIN6 |  |  |
| RAD50 | H2AC4 |  |  |
| DIO2 | HLA-DRB3 | |  |
| RCC1 | LOC109951029 | |  |
| DLG4 | CTH |  |  |
| AFP | MYL2 |  |  |
| LEPQTL1 | TLR7 |  |  |
| IL7R | CD3E |  |  |
| ARHGAP29 | PPP2R2B |  |  |
| ARHGEF16 | ATP2B1 |  |  |
| ARHGEF15 | DGUOK |  |  |
| ARHGAP10 | BRD2 |  |  |
| PLEKHG2 | CHD1 |  |  |
| MAPK9 | NDUFS4 |  |  |
| FIP1L1 | SYNGAP1 | |  |
| TCF3 | HPN |  |  |
| TWIST1 | FCER1G |  |  |
| CBX5 | FAF1 |  |  |
| TRIM33 | CD47 |  |  |
| KLF4 | CDH13 |  |  |
| ADORA2A | SKIV2L |  |  |
| MIR151A | RAB10 |  |  |
| EP300 | SERPINA6 | |  |
| ABCC1 | SH3GL2 |  |  |
| TXNIP | TNFRSF13C | |  |
| PON3 | DHPS |  |  |
| IL37 | ALOX15B |  |  |
| BECN1 | CCNE2 |  |  |
| CXCL9 | UROS |  |  |
| OPN4 | TNFRSF10D | |  |
| HDAC2 | ATF7 |  |  |
| PPIA | AAK1 |  |  |
| MIR9-1 | KTN1 |  |  |
| GRIA1 | SMARCA1 | |  |
| PLA2G6 | CREB3 |  |  |
| MRE11 | AEBP2 |  |  |
| WT1 | GPER1 |  |  |
| IGF2R | CCAR1 |  |  |
| WRN | RANGRF |  |  |
| CALM1 | TOX4 |  |  |
| TNFSF13B | MLF2 |  |  |
| ERCC4 | HTN3 |  |  |
| ABCC2 | MLN |  |  |
| NES | H3C3 |  |  |
| ADAMTSL1 | H3C4 |  |  |
| PTH | ADCY1 |  |  |
| LEPR | CARM1 |  |  |
| LGALS1 | UGT2B7 |  |  |
| DYRK1A | RPS6KA3 |  |  |
| LIPG | FZD4 |  |  |
| SLCO1B1 | HMOX2 |  |  |
| MSN | ZEB2 |  |  |
| DNTT | PSAT1 |  |  |
| CHIT1 | C1R |  |  |
| ORC1 | MAP2K5 |  |  |
| SH2D1A | HSD3B2 |  |  |
| CYGB | HTRA2 |  |  |
| VIP | FANCL |  |  |
| ATXN1 | AICDA |  |  |
| HBG1 | CHD7 |  |  |
| SNCB | TBX3 |  |  |
| SOX2 | SYNJ1 |  |  |
| LIF | OAS1 |  |  |
| SLC8A1 | NQO2 |  |  |
| CD274 | PRKD3 |  |  |
| MIR30A | GAK |  |  |
| ABCB7 | ATP2B4 |  |  |
| PBX1 | EFEMP1 |  |  |
| CYP11B2 | SATB2 |  |  |
| PLD3 | ABCC5 |  |  |
| PCSK1N | MCFD2 |  |  |
| DBH | BDH1 |  |  |
| CAMK2G | TRPM8 |  |  |
| ATN1 | TRIM24 |  |  |
| ITPKC | WNK4 |  |  |
| TLR9 | SSTR1 |  |  |
| CTSL | SNX3 |  |  |
| HOTAIR | PDGFC |  |  |
| PRL | SLC39A4 |  |  |
| UCHL1 | COL9A1 |  |  |
| TKT | LOXL1 |  |  |
| LIMK1 | GDF2 |  |  |
| HK2 | DGKQ |  |  |
| DNM1L | BNIP3 |  |  |
| PTK2B | ADD3 |  |  |
| POU2F3 | SNX5 |  |  |
| EPHX1 | UGT1A6 |  |  |
| AXL | HOXA5 |  |  |
| SLC24A3 | DPM3 |  |  |
| MYOG | INSL3 |  |  |
| MAP1B | UBE3C |  |  |
| MIR196A1 | UGT1A10 |  |  |
| MLLT3 | PEG10 |  |  |
| GAP43 | MTCH2 |  |  |
| SORCS1 | ISG20 |  |  |
| P2RY1 | MED13L |  |  |
| ADAM10 | BAIAP2L1 |  |  |
| CASP7 | ASF1A |  |  |
| PRKCD | TNFRSF10C | |  |
| MTRR | STOM |  |  |
| CDK2 | PHF19 |  |  |
| ADRA2A | TNS4 |  |  |
| DKC1 | URI1 |  |  |
| IL9 | RHOF |  |  |
| NGFR | PIEZO1 |  |  |
| FCER2 | AUP1 |  |  |
| POU5F1 | VPS26B |  |  |
| HAVCR2 | TFPT |  |  |
| NFAT5 | STBD1 |  |  |
| MERTK | MLLT11 |  |  |
| MMP12 | CCDC86 |  |  |
| RUNX3 | CFAP20 |  |  |
| LBP | ATP5IF1 |  |  |
| GNRH1 | CNOT11 |  |  |
| PTPN6 | MIR373 |  |  |
| MEF2A | SPR |  |  |
| CASQ2 | CYLD |  |  |
| SLC25A24 | SLC2A3 |  |  |
| GCGR | QDPR |  |  |
| SLAMF1 | MYLK2 |  |  |
| TUBB2A | GRIK2 |  |  |
| NOX1 | SLC1A1 |  |  |
| PTGIR | CA1 |  |  |
| ERCC1 | GDF5 |  |  |
| GDNF | KDM6A |  |  |
| C5 | SLC4A4 |  |  |
| CYP7A1 | PARN |  |  |
| KDM1A | THBS2 |  |  |
| CYP17A1 | PHKB |  |  |
| FCGR1A | NNMT |  |  |
| EMD | FADS2 |  |  |
| TRPV1 | CLN3 |  |  |
| LACTB | CPE |  |  |
| TP63 | TNFSF13 |  |  |
| IRF4 | TMPRSS2 |  |  |
| MIR19B1 | ARSB |  |  |
| VPS26A | MCCC1 |  |  |
| MIR214 | EFNB2 |  |  |
| CDK5 | USP24 |  |  |
| MFGE8 | TRIB3 |  |  |
| CFLAR | PLIN2 |  |  |
| CCL4 | PIK3R3 |  |  |
| PDCD1 | COL11A1 |  |  |
| TGFB3 | MFN1 |  |  |
| CENPA | CNNM2 |  |  |
| SFTPB | ANXA3 |  |  |
| MEG3 | BCL11B |  |  |
| LCK | E2F2 |  |  |
| FMR1 | MMADHC | |  |
| APOC2 | PROX1 |  |  |
| ADAMTS9 | SEMA3C |  |  |
| TMPO | SARS2 |  |  |
| SLC39A14 | BTG1 |  |  |
| CCR2 | CLDN7 |  |  |
| CD59 | TRPC5 |  |  |
| PIK3CG | SNX1 |  |  |
| EIF3H | HSD17B8 |  |  |
| GAR1 | NPSR1 |  |  |
| SREBF2 | NCR2 |  |  |
| ARNT | ETV5 |  |  |
| HSP90B1 | SEC14L2 |  |  |
| MMP14 | KCTD15 |  |  |
| PTK2 | MTF2 |  |  |
| IKBKB | GORAB |  |  |
| NR1H4 | ESCO2 |  |  |
| SYK | TBC1D15 |  |  |
| PXN | SLX4 |  |  |
| KIR2DL3 | HSPBP1 |  |  |
| ODC1 | CRISP3 |  |  |
| SMAD7 | NAGPA |  |  |
| FUT4 | WBP11 |  |  |
| BAD | TRIM13 |  |  |
| HLA-DQB1 | SNX6 |  |  |
| SLC6A3 | TTLL12 |  |  |
| PLCB1 | COBL |  |  |
| MUTYH | PCNP |  |  |
| HMGA2 | PRPF40B |  |  |
| GAS6 | SLN |  |  |
| CXCR2 | SEPTIN4 |  |  |
| DRD1 | MIR17HG |  |  |
| EGR1 | H3C13 |  |  |
| BMP7 | MIR192 |  |  |
| ETS1 | MIR139 |  |  |
| PRKAA2 | MIR345 |  |  |
| GLRX | MIR190A |  |  |
| HRH1 | MIR148A |  |  |
| SLC20A2 | ZFAS1 |  |  |
| IGF2-AS | MIR497 |  |  |
| KIR2DL1 | BMPR1B |  |  |
| CRH | GK |  |  |
| NGF | ANTXR2 |  |  |
| SLC26A4 | CDH11 |  |  |
| NHP2 | PRKAR1B |  |  |
| NFE2 | IRF7 |  |  |
| GRP | KYNU |  |  |
| RXRA | LAT |  |  |
| EIF2AK2 | ETV1 |  |  |
| TMSB4X | REL |  |  |
| ADRB3 | SCNN1G |  |  |
| GRK2 | MATK |  |  |
| PRKDC | TRPM7 |  |  |
| PYGB | CHRNA3 |  |  |
| DDC | GALNS |  |  |
| PRKAR1A | CDH15 |  |  |
| ISL1 | EIF2AK4 |  |  |
| MEF2C | TBX2 |  |  |
| ATP13A2 | SLC26A2 |  |  |
| ADAM17 | ROBO1 |  |  |
| UMOD | CYP4A11 |  |  |
| MSH6 | JARID2 |  |  |
| CDK6 | CLCNKB |  |  |
| MIR125A | CNDP1 |  |  |
| XPO1 | TNFRSF12A | |  |
| KRIT1 | SOX17 |  |  |
| PRKCA | NTF4 |  |  |
| SORCS3 | SEC63 |  |  |
| HTT | KCNC1 |  |  |
| PRKAA1 | TNFRSF17 | |  |
| AKR1B1 | ATXN10 |  |  |
| SERPINI1 | SUMF1 |  |  |
| GRIN2A | RTN4R |  |  |
| CBS | PODXL |  |  |
| PLCG1 | RAB5B |  |  |
| E2F1 | BRD3 |  |  |
| ATP12A | PPA2 |  |  |
| MCL1 | DSCAM |  |  |
| CRYAA | F13B |  |  |
| APOL1 | CLCA1 |  |  |
| SCD | CRTAP |  |  |
| IKBKG | ANKH |  |  |
| LAMC2 | MMAA |  |  |
| SNAP25 | WRAP53 |  |  |
| CHUK | SLC27A1 |  |  |
| HCRT | PIAS3 |  |  |
| SGCB | PHF1 |  |  |
| SMC3 | MYH1 |  |  |
| IBSP | NRTN |  |  |
| IRF1 | BAZ2A |  |  |
| MUC5AC | CD200 |  |  |
| SFTPD | LTB |  |  |
| CCNB1 | LGALS13 |  |  |
| FOXO3 | CPVL |  |  |
| PLEK | CYBRD1 |  |  |
| CD2 | ARIH1 |  |  |
| PIN1 | DACT1 |  |  |
| BIRC2 | EGFL7 |  |  |
| FLOT2 | SYT7 |  |  |
| TNFRSF10B | MMRN1 |  |  |
| BMPR2 | PIGH |  |  |
| CHRM1 | LGR4 |  |  |
| MYL3 | SNX2 |  |  |
| PSAP | SNX27 |  |  |
| RAB7A | LCOR |  |  |
| MIR181A1 | HMG20A |  |  |
| CLLU1 | FAM13A |  |  |
| CYP1B1 | CELA3B |  |  |
| TAGLN | CHCHD2 |  |  |
| PTGER4 | UFC1 |  |  |
| OPRM1 | TPPP3 |  |  |
| ITPR1 | REPS2 |  |  |
| CENPC | PDZD2 |  |  |
| FGF8 | BUD13 |  |  |
| NDUFS5 | NAV1 |  |  |
| UCP1 | TBC1D5 |  |  |
| CD177 | METRNL |  |  |
| ANXA2 | BCORL1 |  |  |
| ASS1 | DNPH1 |  |  |
| MYOCD | RAB12 |  |  |
| PSMD4 | ADM2 |  |  |
| GLP1R | BRWD3 |  |  |
| MAOB | PRAM1 |  |  |
| TGM2 | MYDGF |  |  |
| MYLK | EMC10 |  |  |
| HSD17B10 | NOMO1 |  |  |
| PSMC6 | CIP2A |  |  |
| MT3 | ENTR1 |  |  |
| CCR7 | MIR615 |  |  |
| TPH1 | GATA6-AS1 | |  |
| H2AX | SPRY4-IT1 | |  |
| ALPL | SCNN1B |  |  |
| HAVCR1 | HSD17B3 |  |  |
| NME1 | KCNJ1 |  |  |
| CDKN1C | BLNK |  |  |
| TBX20 | SLC11A1 |  |  |
| PNLIP | SCN2A |  |  |
| MGAM | IL5RA |  |  |
| PPP3CA | ACACB |  |  |
| ITPR3 | BMP1 |  |  |
| GAL | IL10RA |  |  |
| CCNA2 | FDPS |  |  |
| EEF1A1 | BPGM |  |  |
| SUZ12 | TRPM4 |  |  |
| CDK9 | ICAM2 |  |  |
| SRD5A1 | LAMA4 |  |  |
| UCP3 | ADCY9 |  |  |
| POSTN | CHMP2B |  |  |
| LMO2 | TFR2 |  |  |
| IGFBP5 | TICAM1 |  |  |
| PLA2G4A | KCNJ4 |  |  |
| XRCC6 | GYS2 |  |  |
| PDP1 | HSD17B1 |  |  |
| ERBB4 | IL12RB2 |  |  |
| SNCG | MAGI2 |  |  |
| PAWR | CITED2 |  |  |
| ATF6 | ELOVL5 |  |  |
| NEFM | MTMR2 |  |  |
| PSMC2 | TTBK2 |  |  |
| ALDH1A2 | XPR1 |  |  |
| MASP2 | PRKCSH |  |  |
| RELA | GPNMB |  |  |
| TYK2 | COL5A2 |  |  |
| CYP1A2 | KLHL3 |  |  |
| MTTP | HPGDS |  |  |
| IL17F | MAP3K13 | |  |
| CXCL1 | FBXO7 |  |  |
| PRPF31 | DCTD |  |  |
| HLA-G | EPM2A |  |  |
| IGFBP2 | MATN3 |  |  |
| UGT1A9 | DMP1 |  |  |
| MKI67 | ST8SIA2 |  |  |
| PSMC1 | SLC47A1 |  |  |
| HSPB1 | PLEKHA1 |  |  |
| ALCAM | NPC2 |  |  |
| SDC1 | ARAP1 |  |  |
| HTR1A | COQ2 |  |  |
| HS3ST1 | HOXB9 |  |  |
| TCF21 | LRBA |  |  |
| HOTTIP | GLRX3 |  |  |
| CNMD | GDF11 |  |  |
| NOG | FKBP1B |  |  |
| PNP | AGGF1 |  |  |
| TFAM | EBF1 |  |  |
| HLA-DRA | UBASH3B |  |  |
| EDN2 | NAB2 |  |  |
| IL12B | WDFY3 |  |  |
| KCNQ1OT1 | TET3 |  |  |
| MIR183 | PMM1 |  |  |
| CYP2B6 | RHBDF2 |  |  |
| MSH2 | COX17 |  |  |
| KRT8 | CCRL2 |  |  |
| FETUB | LRRC8A |  |  |
| DIABLO | JPH2 |  |  |
| TMEM67 | DEPDC5 |  |  |
| LBR | AKT1S1 |  |  |
| ACAN | ARIH2 |  |  |
| KCNMA1 | TRIM9 |  |  |
| NEAT1 | SPDEF |  |  |
| SETD2 | TMEFF2 |  |  |
| COL8A1 | RNF19A |  |  |
| HEPH | WNK3 |  |  |
| NTF3 | PRG4 |  |  |
| H4-16 | PIGT |  |  |
| FGF4 | SEMA6A |  |  |
| SMTN | SACM1L |  |  |
| SLC18A3 | TNS1 |  |  |
| ZAP70 | FIS1 |  |  |
| DNMT3B | WLS |  |  |
| LOC111365141 | DENR |  |  |
| CYP11B1 | MAN2C1 |  |  |
| OGDH | ERAP2 |  |  |
| CXCL16 | VPREB1 |  |  |
| GAD2 | ZFPM1 |  |  |
| BCL6 | SOX8 |  |  |
| TARDBP | PKHD1 |  |  |
| CASP10 | NPRL3 |  |  |
| IGBP1 | NOX5 |  |  |
| PDE3A | SIPA1L2 |  |  |
| CAV3 | SAP30L |  |  |
| GRIN2B | ZBTB40 |  |  |
| EIF2AK3 | CLEC16A |  |  |
| SLC1A2 | ANO7 |  |  |
| CD27 | ATP13A3 |  |  |
| F2RL1 | MORC3 |  |  |
| ACP1 | TMEM230 | |  |
| CCNE1 | SGMS2 |  |  |
| CYP27B1 | SLC39A2 |  |  |
| TYMS | ARMC1 |  |  |
| ERBB3 | FUNDC1 |  |  |
| CRKL | AFF3 |  |  |
| SSB | LRCH1 |  |  |
| CFHR2 | HJV |  |  |
| TNFRSF9 | CDC123 |  |  |
| ACVRL1 | BET1L |  |  |
| DRD3 | KCTD13 |  |  |
| CAMK2A | SLC9B2 |  |  |
| TNFRSF10A | MYRF |  |  |
| RAN | AGBL4 |  |  |
| IL6R | MFHAS1 |  |  |
| ALK | MIEN1 |  |  |
| HTR2B | GUCY1B1 |  |  |
| CRK | SMG9 |  |  |
| INTS6 | YRDC |  |  |
| INHBA | PAGE4 |  |  |
| CSF2RA | RETREG1 |  |  |
| BCL2L2 | SEPTIN8 |  |  |
| STAT6 | TBC1D3 |  |  |
| MYO9A | ABRAXAS2 | |  |
| BIRC3 | MIRLET7D | |  |
| BAK1 | MIR206 |  |  |
| PVALB | EGOT |  |  |
| FUCA1 | MIR196B |  |  |
| VCAN | MIR101-1 | |  |
| EPX | MIR32 |  |  |
| PYY | MIR16-2 |  |  |
| MVP | MIR367 |  |  |
| GREM1 | MIR708 |  |  |
| SNHG1 | MIR503 |  |  |
| MYOM2 | MIR198 |  |  |
| NRP1 | TBC1D3D |  |  |
| HELLS | MIR376A1 | |  |
| CACNB2 | PINK1-AS | |  |
| CKB | LOC108663987 | |  |
| KLRG1 | LOC108663996 | |  |
| NECTIN2 | RIPK1 |  |  |
| RPS6KB1 | IL34 |  |  |
| CNTF | SLC12A1 |  |  |
| ZEB1 | ROR1 |  |  |
| ILK | DGAT1 |  |  |
| CENPB | NEDD4L |  |  |
| CYP46A1 | TNFRSF13B | |  |
| NCOA2 | PRCP |  |  |
| IL22 | RORC |  |  |
| SCT | RASGRP2 |  |  |
| OGT | FUT2 |  |  |
| VASP | KCNN3 |  |  |
| LYN | ENPEP |  |  |
| MIR128-1 | PTPRN2 |  |  |
| NEU1 | VEGFB |  |  |
| GGH | SLC44A1 |  |  |
| ROCK1 | PDGFD |  |  |
| SDHC | KMO |  |  |
| ALMS1 | CD226 |  |  |
| LPAR3 | GRB14 |  |  |
| UGT2B15 | GRK4 |  |  |
| OXA1L | KREMEN1 | |  |
| KIR3DL1 | BAMBI |  |  |
| FOSL1 | TMPRSS6 |  |  |
| MMP28 | NOL3 |  |  |
| EPAS1 | COMMD1 | |  |
| HCK | HAND1 |  |  |
| PRKAB1 | LMX1A |  |  |
| SLC19A1 | NEUROG3 | |  |
| SELENOP | BRDT |  |  |
| SSRP1 | BCL9 |  |  |
| EIF4E | ANKRD1 |  |  |
| RIC3 | RHOT1 |  |  |
| VDAC1 | CD84 |  |  |
| HSD11B1 | ITGB8 |  |  |
| HLA-DRB5 | MAFA |  |  |
| CD68 | BACH2 |  |  |
| AQP4 | CAMK1D |  |  |
| KIR2DS2 | FUCA2 |  |  |
| TPMT | GALNT10 |  |  |
| TAF1L | GREM2 |  |  |
| ANXA1 | HLA-DOA | |  |
| ATP7B | GLMN |  |  |
| CDX2 | CLPS |  |  |
| PNMT | CREB5 |  |  |
| TSC1 | TCN1 |  |  |
| FANCD2 | TSEN2 |  |  |
| CALB1 | ZBTB20 |  |  |
| TP53BP1 | STEAP4 |  |  |
| FLNA | MNDA |  |  |
| WNT5A | PIGL |  |  |
| BLVRB | NHLRC1 |  |  |
| IFNAR1 | ARTN |  |  |
| ADRA2B | ASPN |  |  |
| BIN1 | EN2 |  |  |
| PTHLH | FKBP14 |  |  |
| TNFRSF4 | GNPTG |  |  |
| S100A8 | GPR119 |  |  |
| DROSHA | SPG21 |  |  |
| HLA-DMA | TTC3 |  |  |
| HBS1L | SOX18 |  |  |
| MIR24-1 | STEAP1 |  |  |
| ITGAX | PKIB |  |  |
| MIR103A1 | PLAG1 |  |  |
| IGFBP4 | SLC30A4 |  |  |
| PSENEN | SLC45A3 |  |  |
| RTN4 | RAB39B |  |  |
| TIA1 | RIT2 |  |  |
| SLC18A2 | CD48 |  |  |
| TUBA4A | IRX1 |  |  |
| CYP2J2 | LMCD1 |  |  |
| HIP1R | DYNC2H1 | |  |
| FGF7 | TMEM106B | |  |
| ANK3 | TSEN34 |  |  |
| FBN2 | POU4F1 |  |  |
| MIR124-1 | MYH15 |  |  |
| CNTNAP2 | INPP5F |  |  |
| RFC1 | MFAP3 |  |  |
| EPRS1 | CDAN1 |  |  |
| CYP21A2 | TRIM39 |  |  |
| PLCG2 | PMEPA1 |  |  |
| NEFL | PRUNE2 |  |  |
| DDIT3 | SLC12A9 |  |  |
| PEBP1 | OTULIN |  |  |
| UBB | MYBPH |  |  |
| CHM | RANBP17 |  |  |
| SP7 | SLC41A1 |  |  |
| INTS2 | KCNE4 |  |  |
| TBX18 | HLA-DQA2 | |  |
| BMPER | DCAF17 |  |  |
| MIR371A | RNF11 |  |  |
| MIR379 | RBPJL |  |  |
| ATF4 | SENP7 |  |  |
| REST | SEC62 |  |  |
| PRDX3 | NEU4 |  |  |
| SFRP1 | NAALADL2 | |  |
| NUP98 | TNFSF8 |  |  |
| KLK2 | FBXL5 |  |  |
| OGA | ASPG |  |  |
| FURIN | RBFOX3 |  |  |
| TBX4 | U2AF1L4 |  |  |
| HRH2 | GMEB1 |  |  |
| RASA1 | CDH24 |  |  |
| HM13 | SRCIN1 |  |  |
| PIK3CD | SLC48A1 |  |  |
| CNR2 | PBXIP1 |  |  |
| LAMC1 | LRRC8C |  |  |
| CCL7 | FAM114A2 | |  |
| PCSK6 | CTAG2 |  |  |
| PSCA | TBRG1 |  |  |
| NOP10 | OR51E2 |  |  |
| MIR15B | ANKRD50 | |  |
| CAPN1 | METTL8 |  |  |
| NOTCH2 | JRKL |  |  |
| CYP24A1 | DENND6A | |  |
| APH1A | CMC2 |  |  |
| NDE1 | POU5F1B |  |  |
| SUPT16H | MITD1 |  |  |
| SGK1 | RAB29 |  |  |
| KLK1 | FAAP24 |  |  |
| CUL1 | SEPTIN3 |  |  |
| IGHV4-38-2 | HLA-DRB4 | |  |
| GC | FAM199X |  |  |
| XPA | HCP5 |  |  |
| NTRK2 | CIAO3 |  |  |
| APBB1 | GET1 |  |  |
| AQP1 | PRAC1 |  |  |
| STK4 | WASHC1 |  |  |
| KLK11 | MIR30B |  |  |
| ELAVL4 | CRNDE |  |  |
| CCND2 | MIR219A1 | |  |
| IL6ST | PRAC2 |  |  |
| LEMD3 | MIR324 |  |  |
| MT-TT | MIR217 |  |  |
| LOC106146150 | MIR100HG | |  |
| LOC106146151 | MIR372 |  |  |
| CLEC4A | MIR675 |  |  |
| MLKL | ATXN8 |  |  |
| ADAMTS7 | RNU1-1 |  |  |
| TNNI1 | MORF4L2 | |  |
| ACACA | HTR1D |  |  |
| PI4KA | MLYCD |  |  |
| C5AR2 | MIR215 |  |  |
| COL6A2 | RAB3IL1 |  |  |
| SPON1 |  |  |  |

**Part 3 contains Table S4 and Table S5.**

**Table S4 Approved Western drugs for stable angina pectoris**

| Drug | Genename | Evidence |
| --- | --- | --- |
| Amiodarone | KCNH2 | 10.1111/j.1440-1681.2008.04964.x\|10.2174/0929867043456296\|10.1038/bjp.2008.287 |
| Amiodarone | KCNH6 | 10.1111/j.1440-1681.2008.04964.x\|10.2174/0929867043456296\|10.1038/bjp.2008.287 |
| Amiodarone | KCNH7 | 10.1111/j.1440-1681.2008.04964.x\|10.2174/0929867043456296\|10.1038/bjp.2008.287 |
| Amiodarone | ADRB1 | 10.1006/bbrc.1998.0138\|10.1016/0006-2952(91)90212-n |
| Amiodarone | ADRB2 | 10.1006/bbrc.1998.0138\|10.1016/0006-2952(91)90212-n |
| Amiodarone | ADRB3 | 10.1006/bbrc.1998.0138\|10.1016/0006-2952(91)90212-n |
| Amiodarone | CACNA1I | 10.1085/jgp.100.4.703\|10.1055/s-0030-1249208\|10.1097/00005344-199424050-00004 |
| Amiodarone | CACNA1C | 10.1085/jgp.100.4.703\|10.1055/s-0030-1249208\|10.1097/00005344-199424050-00004\|10.1038/sj.bjp.0703527 |
| Amiodarone | CACNA1D | 10.1085/jgp.100.4.703\|10.1055/s-0030-1249208\|10.1097/00005344-199424050-00004\|10.1038/sj.bjp.0703527 |
| Amiodarone | CACNA1F | 10.1085/jgp.100.4.703\|10.1055/s-0030-1249208\|10.1097/00005344-199424050-00004\|10.1038/sj.bjp.0703527 |
| Amiodarone | CACNA1S | 10.1085/jgp.100.4.703\|10.1055/s-0030-1249208\|10.1097/00005344-199424050-00004\|10.1038/sj.bjp.0703527 |
| Amiodarone | CACNB1 | 10.1085/jgp.100.4.703\|10.1055/s-0030-1249208\|10.1097/00005344-199424050-00004\|10.1038/sj.bjp.0703527 |
| Amiodarone | CACNB2 | 10.1085/jgp.100.4.703\|10.1055/s-0030-1249208\|10.1097/00005344-199424050-00004\|10.1038/sj.bjp.0703527 |
| Amiodarone | CACNB3 | 10.1085/jgp.100.4.703\|10.1055/s-0030-1249208\|10.1097/00005344-199424050-00004\|10.1038/sj.bjp.0703527 |
| Amiodarone | CACNB4 | 10.1085/jgp.100.4.703\|10.1055/s-0030-1249208\|10.1097/00005344-199424050-00004\|10.1038/sj.bjp.0703527 |
| Amiodarone | THRA | 10.1021/jm001126+\|10.1172/jci113874\|10.1177/2042018811398516 |
| Amiodarone | THRB | 10.1021/jm001126+\|10.1172/jci113874\|10.1177/2042018811398516 |
| Amiodarone | PPARG | 10.1371/journal.pone.0086795\|10.1093/toxsci/kfq361 |
| Amiodarone | PPARA | 10.1371/journal.pone.0086795\|10.1124/jpet.104.072785 |
| Amiodarone | PPARGC1B | 10.1371/journal.pone.0086795 |
| Amiodarone | CYP3A4 | 10.2174/092986709789057635\|10.18433/j3sg66\|\|10.1046/j.1365-2125.2000.00134.x |
| Amiodarone | CYP2C8 | 10.2174/092986709789057635\| |
| Amiodarone | CYP2D6 | 10.2174/092986709789057635\|10.2165/00002018-200326060-00004\|10.1046/j.1365-2125.2000.00134.x\|10.2133/dmpk.21.501\|10.1111/j.2042-7158.1994.tb05721.x |
| Amiodarone | CYP2C9 | 10.2174/092986709789057635\|10.2165/00002018-200326060-00004\|10.1177/107424840100600405\|10.1038/clpt.1992.39 |
| Amiodarone | CYP1A2 | 10.1177/107424840100600405\|10.1124/dmd.115.065623 |
| Amiodarone | CYP2C19 | 10.1046/j.1365-2125.2000.00134.x |
| Amiodarone | CYP1A1 | 10.18433/j3sg66\|10.1016/j.toxlet.2016.04.016 |
| Amiodarone | CYP2A6 | 10.1046/j.1365-2125.2000.00134.x |
| Amiodarone | CYP2J2 | 10.1016/j.bcp.2016.03.005\|10.1016/j.bcp.2017.09.012 |
| Amiodarone | ABCB1 | 10.1016/s0928-0987(00)00215-3\|10.1124/jpet.102.037549\|10.1097/00001813-199607000-00012\|10.1023/a:1018877803319 |
| Amiodarone | ABCB11 | 10.1093/toxsci/kft197 |
| Amiodarone | ALB | 10.1111/j.1365-2125.1988.tb05311.x\|10.1007/bf02004505\|10.1111/j.2042-7158.1984.tb04400.x |
| Pantoprazole | ATP4A | 10.2165/1153121-s0-000000000-00000\|10.2165/00003495-200363010-00006\|10.1517/17425255.4.4.471 |
| Pantoprazole | DDAH1 | 10.1161/circulationaha.113.003602\|10.1038/s41598-017-03069-1 |
| Pantoprazole | CYP2C19 | 10.1124/dmd.111.041293\|\|10.2147/tcrm.s43151\|10.1080/17425255.2018.1461835 |
| Pantoprazole | ABCB1 | 10.1007/s00210-001-0489-7\|10.1007/s40264-014-0144-0\|10.1007/s10637-008-9138-z\|10.1124/dmd.112.050286 |
| Pantoprazole | ABCG2 | 10.1158/0008-5472.can-03-4062\|10.1111/j.1365-2125.2008.03303.x\|10.1152/ajpgi.00102.2009 |
| Pantoprazole | SLC22A8 | 10.1124/dmd.108.024901\|10.1124/dmd.114.058529 |
| Omeprazole | ATP4A | 10.1007/s00228-008-0538-y\|10.1007/s00228-008-0576-5\|10.1007/s00424-008-0495-4\|10.1021/bi062305h\|10.1016/0010-440x(78)90019-6\|10.1111/j.1365-2036.2006.02943.x |
| Omeprazole | AHR | 10.1074/jbc.272.19.12705 |
| Omeprazole | CYP1A1 | 10.1016/s0940-2993(99)80018-9\|10.1211/jpp.60.7.0005 |
| Omeprazole | CYP1A2 | 10.1055/s-2003-39980\|10.1080/0049825021000023978\|10.2174/138920005774330620\|10.1046/j.1365-2125.2002.01686.x\|\|10.1038/clpt.1992.126\|10.1016/0006-291x(92)91130-i |
| Omeprazole | CYP1B1 | 10.1080/0049825021000023978 |
| Omeprazole | CYP2C9 | 10.1006/abbi.1998.0615\|10.3389/fphar.2016.00098 |
| Omeprazole | CYP2D6 | 10.1080/00498250110035615\|10.1124/dmd.32.8.821 |
| Omeprazole | CYP3A4 | 10.1124/jpet.105.090928\|10.2174/092986709789057635\|\|10.1016/j.ejps.2004.10.006\|10.1007/bf02972986\|10.2174/138920005774330620\|10.1016/j.bcp.2003.09.022\|\|10.1046/j.1365-2125.2000.00122.x |
| Omeprazole | CYP2C19 | 10.1124/dmd.107.019265\|10.1124/dmd.32.8.821\|10.1124/jpet.105.090928\|10.2174/092986709789057635\|\|\|\|10.1016/s0009-9236(96)90004-1 |
| Omeprazole | ABCG2 | 10.1158/0008-5472.can-03-4062\|10.1111/j.1365-2125.2008.03303.x |
| Omeprazole | ABCC3 | 10.1124/jpet.102.043547 |
| Omeprazole | ABCB1 | 10.1007/s00210-001-0489-7\|10.2147/tcrm.s43151\|10.1097/mjt.0000000000000221 |
| Bisoprolol | ADRB1 | 10.1016/j.ejphar.2003.11.063\|10.1016/j.jacc.2005.08.041\|10.1007/bf00280067\|10.1097/00005344-199003000-00012\|10.1093/nar/30.1.412 |
| Bisoprolol | CYP3A4 | 10.1021/js970316d\|10.2174/1381612820666141024151119 |
| Bisoprolol | ABCB1 | 10.1111/j.1472-8206.2006.00408.x\|10.2133/dmpk.23.340\|10.1097/mjt.0000000000000786\|10.3390/pharmaceutics3040680 |
| Ephedrine | SLC6A2 | 10.1213/01.ane.0000092917.96558.3c\|10.1016/s0376-8716(03)00011-5\|10.1146/annurev.pharmtox.47.120505.105140\|10.1016/j.pneurobio.2005.04.003 |
| Ephedrine | ADRA1A | 10.1124/jpet.107.120709\|10.1002/syn.10182\|10.1093/nar/28.1.235 |
| Ephedrine | ACHE | 10.1016/s0742-8413(97)00196-5 |
| Ephedrine | SLC18A2 | 10.1002/syn.20896\|10.1124/jpet.110.175117\|10.1016/j.pneurobio.2005.04.003\|\|10.1016/j.neulet.2009.03.049\|10.1146/annurev.pharmtox.47.120505.105140 |
| Ephedrine | ALB | 10.1016/j.ejps.2018.10.027 |
| Oxymetazoline | ADRA1A | 10.1016/0014-2999(96)00442-6\|\|\|10.1111/j.1476-5381.1995.tb16380.x\|10.1016/s0014-2999(01)01217-1 |
| Oxymetazoline | ADRA2A | 10.1111/j.1476-5381.1992.tb14449.x\|10.1152/ajpendo.00477.2006\|10.1007/bf00374320 |
| Oxymetazoline | ADRA2C | 10.1111/j.1472-8206.2009.00805.x |
| Oxymetazoline | ADRA2B | 10.1016/0014-2999(91)90299-6\|10.1093/nar/gkr777 |
| Oxymetazoline | ADRA1B | 10.1111/j.1472-8206.2009.00805.x\|10.1124/mol.112.082313\|10.1093/nar/gkr777 |
| Oxymetazoline | ADRA1D | 10.1111/j.1472-8206.2009.00805.x\|10.1124/mol.112.082313\|10.1093/nar/gkr777 |
| Pseudoephedrine | SLC6A2 | 10.1016/j.ejphar.2004.04.030\|10.1007/s00702-002-0695-6 |
| Pseudoephedrine | SLC6A3 | 10.1016/j.ejphar.2004.04.030\|10.1007/s00702-002-0695-6 |
| Pseudoephedrine | ADRA1A | 10.1038/nrd2199\|10.1038/nrd2132\|10.2165/00003495-200161150-00009\| |
| Pseudoephedrine | ADRA2A | 10.1038/nrd2199\|10.1038/nrd2132\| |
| Pseudoephedrine | SLC6A4 | 10.1016/j.ejphar.2004.04.030 |
| Pseudoephedrine | ADRB2 | 10.1038/nrd2199\|10.1038/nrd2132\|10.1016/s0006-2952(99)00152-5 |
| Pseudoephedrine | ADRB1 | 10.1016/s0006-2952(99)00152-5 |
| Pseudoephedrine | NFATC1 | 10.3109/08923973.2011.582118 |
| Pseudoephedrine | TNF | 10.1016/j.ejphar.2013.11.032\|10.3109/08923973.2011.582118 |
| Pseudoephedrine | NFKB1 | 10.3109/08923973.2011.582118 |
| Pseudoephedrine | ATF1 | 10.3109/08923973.2011.582118 |
| Pseudoephedrine | ATF2 | 10.3109/08923973.2011.582118 |
| Pseudoephedrine | ATF3 | 10.3109/08923973.2011.582118 |
| Pseudoephedrine | ATF4 | 10.3109/08923973.2011.582118 |
| Pseudoephedrine | ATF5 | 10.3109/08923973.2011.582118 |
| Pseudoephedrine | ATF6 | 10.3109/08923973.2011.582118 |
| Pseudoephedrine | ATF7 | 10.3109/08923973.2011.582118 |
| Pseudoephedrine | JDP2 | 10.3109/08923973.2011.582118 |
| Pseudoephedrine | FOS | 10.3109/08923973.2011.582118 |
| Pseudoephedrine | JUN | 10.3109/08923973.2011.582118 |
| Pseudoephedrine | IL2 | 10.3109/08923973.2011.582118 |
| Pseudoephedrine | MAOA | 10.1016/s0006-2952(00)00306-3 |
| Pseudoephedrine | SLC6A2 | 10.1016/j.ejphar.2004.04.030\|10.1007/s00702-002-0695-6 |
| Pseudoephedrine | SLC6A3 | 10.1016/j.ejphar.2004.04.030\|10.1007/s00702-002-0695-6 |
| Pseudoephedrine | SLC6A4 | 10.1016/j.ejphar.2004.04.030\|10.1007/s00702-002-0695-6 |
| Pseudoephedrine | ALB | 10.1016/j.ejps.2018.10.027 |
| Cilostazol | PDE3A | 10.1093/nar/30.1.412\|10.2174/138161206775474323\|10.1046/j.1463-1326.2002.0040s2s14.x\|\|10.1016/j.pupt.2007.11.003 |
| Cilostazol | CYP3A5 | 10.2165/00003088-199937002-00007 |
| Cilostazol | CYP3A7 | 10.2165/00003088-199937002-00007 |
| Cilostazol | CYP3A7-CYP3A51P | 10.2165/00003088-199937002-00007 |
| Cilostazol | CYP2C19 | 10.2165/00003088-199937002-00007 |
| Cilostazol | CYP3A4 | 10.2165/00003088-199937002-00007 |
| Cilostazol | CYP1A2 | 10.2165/00003088-199937002-00007 |
| Cilostazol | CYP2D6 | 10.2165/00003088-199937002-00007 |
| Amlodipine | CACNA1C | 10.1172/jci27167\|10.1038/nature19102 |
| Amlodipine | CACNA1B | 10.1038/sj.bjp.0701226\|\|10.1016/j.ejphar.2010.01.006\|10.1016/s0014-2999(01)00985-2\|10.1055/s-0031-1296398\| |
| Amlodipine | CACNB1 | 10.1038/sj.bjp.0701226\|\|10.1016/j.ejphar.2010.01.006\|10.1016/s0014-2999(01)00985-2\|10.1055/s-0031-1296398\| |
| Amlodipine | CACNA2D3 | 10.1038/sj.bjp.0701226\|\|10.1016/j.ejphar.2010.01.006\|10.1016/s0014-2999(01)00985-2\|10.1055/s-0031-1296398\| |
| Amlodipine | CA1 | 10.1067/mcp.2000.110559\|10.1023/a:1007893207279\| |
| Amlodipine | SMPD1 | 10.1371/journal.pone.0023852 |
| Amlodipine | CACNA1I | 10.1016/j.ejphar.2011.08.005\|10.2174/1874467208666150507105845\|10.1055/s-0031-1300433\|10.2174/157340206776877370 |
| Amlodipine | CACNA1B | 10.1016/j.ejphar.2010.01.006\|10.3389/fphar.2017.00286\|10.1038/sj.bjp.0701226 |
| Amlodipine | CYP3A4 | 10.1007/s002280050706\|10.1124/dmd.113.055400 |
| Amlodipine | CYP1A1 | 10.1007/s002280050706 |
| Amlodipine | CYP2B6 | 10.1007/s002280050706\|10.1021/bi300894z |
| Amlodipine | CYP3A5 | 10.1124/dmd.113.055400\|10.1159/000258688\|10.3892/mmr.2017.6214\|10.1038/jhh.2013.67 |
| Amlodipine | CYP2C8 | 10.1038/clpt.2011.295\|10.1124/pr.115.011411 |
| Amlodipine | CYP2D6 | 10.1097/jcp.0000000000000929\| |
| Amlodipine | ABCB1 | 10.1023/a:1007568811691\|10.1211/0022357043941\|10.1097/01.tp.0000084873.20157.67\|10.1111/j.1365-2125.2006.02733.x |
| Acetylcholine | ACHE | 10.1038/nrd2199\|10.1038/nrd2132\|10.1093/nar/28.1.235 |
| Acetylcholine | CHRFAM7A | 10.1124/jpet.103.048777 |
| Acetylcholine | CHRNA7 | 10.1124/jpet.103.048777 |
| Acetylcholine | SLC22A5 | 10.1124/mol.59.2.358 |
| Acetylcholine | SLC22A1 | 10.1016/0014-5793(96)01030-7\|10.1186/1465-9921-7-65 |
| Methyltestosterone | AR | 10.1016/j.juro.2006.06.071\|\|10.14670/hh-22.107\|10.1159/000098402\|10.1530/eje-06-0607\|10.1093/nar/30.1.412 |
| Methyltestosterone | ESR1 | 10.1038/srep05664 |
| Methyltestosterone | CYP2B6 | 10.1081/dmr-120001392 |
| Methyltestosterone | CYP3A4 | 10.1016/s0378-4347(99)00400-4 |
| Methyltestosterone | CYP19A1 | 10.1159/000054699 |
| Methyltestosterone | SLCO1A2 | 10.1152/ajprenal.1996.270.2.f332\|10.1152/ajprenal.1996.270.2.f326\| |
| Methyltestosterone | SLC22A8 | 10.1006/bbrc.2001.6180 |
| Methyltestosterone | ALB | 10.1016/s0300-595x(86)80024-x |
| Methyltestosterone | SHBG | 10.1016/s0300-595x(86)80024-x |
| Testosterone | AR | 10.1016/j.juro.2006.06.071\|\|10.14670/hh-22.107\|10.1159/000098402\|10.1530/eje-06-0607\|10.1093/nar/30.1.412 |
| Testosterone | ESR1 | 10.1289/ehp.5724\|10.1096/fj.99-0863com |
| Testosterone | NR3C2 | 10.1124/mol.106.031112 |
| Testosterone | CYP3A4 | 10.1016/s1570-0232(02)00548-2\|10.1124/dmd.117.078055\|10.1002/0471140856.tx0413s20\|10.4103/1008-682x.133320 |
| Testosterone | CYP3A5 | 10.18433/jpps30558\|10.4103/1008-682x.133320 |
| Testosterone | CYP3A7 | 10.4103/1008-682x.133320 |
| Testosterone | CYP3A7-CYP3A51P | 10.4103/1008-682x.133320 |
| Testosterone | CYP3A43 | 10.4103/1008-682x.133320 |
| Testosterone | CYP19A1 | 10.1016/j.brainresrev.2007.10.011\|10.1073/pnas.141543298 |
| Testosterone | MAOA | 10.1038/sj.npp.1301417\|10.1016/0306-3623(77)90066-0 |
| Testosterone | CYP11A1 | 10.1093/toxsci/kfr063 |
| Testosterone | CYP1A1 | 10.1097/00008571-200008000-00005\|10.1155/2014/764102 |
| Testosterone | CYP1B1 | 10.1093/carcin/20.8.1607\|10.1371/journal.pone.0068634\|10.1161/hypertensionaha.115.06936 |
| Testosterone | CYP2A13 | 10.1093/carcin/bgh348 |
| Testosterone | CYP2B6 | 10.1016/s0378-4347(99)00400-4\|10.1016/s0006-2952(98)00018-5 |
| Testosterone | CYP2C19 | 10.1124/dmd.105.006569\|10.1006/abbi.1997.0302 |
| Testosterone | CYP2C9 | 10.1081/dmr-120001392 |
| Testosterone | CYP2C8 | 10.1124/pr.115.011411 |
| Testosterone | SLC22A8 | 10.1006/bbrc.2001.6180 |
| Testosterone | SLC22A7 | 10.1006/bbrc.2001.6180 |
| Testosterone | SLC10A1 | 10.1152/ajpgi.1998.274.2.g370 |
| Testosterone | ABCG2 | 10.1074/jbc.m301358200 |
| Testosterone | SLCO1B3 | 10.1210/en.2014-1337\|10.1111/j.1464-410x.2008.07629.x |
| Testosterone | ALB | 10.1016/s0300-595x(86)80024-x\|10.1210/jcem-69-1-200 |
| Testosterone | SHBG | 10.1016/s0300-595x(86)80024-x\|10.1210/jcem-69-1-200 |
| Ramipril | ACE | 10.1093/nar/30.1.412\|10.1186/1472-6904-6-1\|10.1093/ajhp/57.suppl_1.s3\|10.2165/00003088-200241030-00005 |
| Ramipril | BDKRB1 | 10.1074/jbc.m200355200 |
| Ramipril | BCHE | 10.1097/00005344-200008000-00005 |
| Ramipril | SLC15A1 | 10.1124/jpet.108.143339 |
| Ramipril | SLC15A2 | 10.1124/jpet.108.143339 |
| Atenolol | ADRB1 | 10.1152/ajpcell.2000.279.2.c495\|10.1016/s0014-2999(02)01533-9\|10.1016/j.lfs.2007.06.003\|10.1097/00005344-199709000-00008\|10.1093/nar/30.1.412 |
| Atenolol | ADRB2 | 10.1046/j.1365-2710.2003.00477.x |
| Atenolol | CYP2D6 | 10.1517/17425255.2.6.981\|10.1055/s-0031-1299835 |
| Atenolol | ABCB11 | 10.1093/toxsci/kft197 |
| Atenolol | ALB | 10.1002/jmr.2715 |
| Pravastatin | HMGCR | 10.1093/nar/30.1.412\|10.4103/0366-6999.149226\| |
| Pravastatin | HDAC2 | 10.1158/0008-5472.can-07-5807 |
| Pravastatin | SLCO1B1 | 10.1124/dmd.104.002477\|10.1074/jbc.274.52.37161\|10.1124/jpet.105.085589\|10.1097/01.fpc.0000170913.73780.5f\|10.3109/00498250903351013 |
| Pravastatin | SLCO2B1 | 10.1097/fpc.0b013e32831bd98c\|10.1124/jpet.103.051300\|10.1124/jpet.103.060194 |
| Pravastatin | ABCB1 | 10.1124/dmd.104.002477\|10.1124/jpet.105.085589\|10.1155/2017/3418204 |
| Pravastatin | SLCO1A2 | 10.1074/jbc.274.52.37161 |
| Pravastatin | SLC22A6 | 10.1254/jphs.94.197 |
| Pravastatin | SLC22A8 | 10.1254/jphs.94.197\|10.1124/jpet.103.063370\|10.1124/jpet.300.3.746 |
| Pravastatin | ABCC2 | 10.1074/jbc.m109081200\|10.1124/jpet.105.085589 |
| Pravastatin | SLC22A11 | 10.1254/jphs.94.197 |
| Pravastatin | ABCG2 | 10.1074/jbc.m212399200\|10.1124/jpet.105.085589 |
| Pravastatin | SLC22A7 | 10.1254/jphs.94.197 |
| Pravastatin | SLC16A1 | 10.1023/a:1016269806840 |
| Pravastatin | ABCB11 | 10.1111/j.1872-034x.2009.00493.x |
| Pravastatin | SLCO1B3 | 10.1074/jbc.274.52.37161 |
| Hydrochlorothiazide | SLC12A3 | 10.1093/nar/30.1.412\|\|10.1161/01.hyp.0000186240.81996.57\|10.1681/asn.v95819\|10.1152/ajpcell.1997.272.1.c109\|10.1055/s-2003-39456\|10.1152/ajprenal.2000.279.1.f161 |
| Hydrochlorothiazide | KCNMA1 | 10.1161/01.hyp.32.6.1071 |
| Hydrochlorothiazide | SLC22A6 | 10.1006/bbrc.1998.9978\| |
| Hydrochlorothiazide | SLC22A8 | 10.1681/asn.2005090966 |
| Hydrochlorothiazide | ABCC4 | 10.1681/asn.2005090966 |
| Hydrochlorothiazide | ALB | 10.1016/j.jpba.2018.09.009 |
| Dopamine | DRD2 | 03.04/ijaai.169174\|10.1093/nar/30.1.412 |
| Dopamine | DRD1 | 03.04/ijaai.169174\|10.1002/ajmg.b.30544\|10.1007/bf03033483\|10.1007/s10899-007-9060-x\|10.1529/biophysj.106.088500 |
| Dopamine | DRD5 | 03.04/ijaai.169174 |
| Dopamine | DRD3 | 03.04/ijaai.169174 |
| Dopamine | DRD4 | 03.04/ijaai.169174 |
| Dopamine | SLC6A3 | 03.04/ijaai.169174 |
| Dopamine | DBH | 10.1016/j.ntt.2006.11.011\|10.1111/j.1460-9568.2007.05557.x\|10.1152/ajpheart.01389.2006\|10.1016/j.cbpc.2006.12.013\|10.1016/j.physbeh.2007.02.011 |
| Dopamine | HTR1A | 10.1016/s0742-8413(97)00614-2\| |
| Dopamine | HTR7 | 10.1016/0896-6273(93)90149-l\| |
| Dopamine | DRD1 | 10.1021/jm991098z |
| Dopamine | DRD5 | 10.1021/jm991098z |
| Dopamine | SLC6A2 | 10.1002/1098-2396(20010101)39:1<32::aid-syn5>3.0.co;2-3 |
| Dopamine | SLC6A4 | 10.1002/1098-2396(20010101)39:1<32::aid-syn5>3.0.co;2-3 |
| Dopamine | HTR3A | 10.1124/jpet.106.118752 |
| Dopamine | HTR3B | 10.1124/jpet.106.118752 |
| Dopamine | SOD1 | 10.1038/ncomms2750 |
| Dopamine | SLC18A2 | 10.1016/0169-328x(94)90050-7 |
| Dopamine | MAOA | 10.1016/j.addr.2008.06.002\|10.1016/j.bbamcr.2010.09.010\|10.1196/annals.1330.023 |
| Dopamine | MAOB | 10.1016/j.addr.2008.06.002\|10.1016/j.bbamcr.2010.09.010 |
| Dopamine | COMT | 10.1002/ajmg.b.31205\|10.1177/0269881111400644\|10.1196/annals.1330.023 |
| Dopamine | DBH | 10.1016/j.ntt.2006.11.011\|10.1111/j.1460-9568.2007.05557.x\|10.1152/ajpheart.01389.2006\|10.1016/j.cbpc.2006.12.013\|10.1016/j.physbeh.2007.02.011 |
| Dopamine | SLC22A2 | 10.1097/01.asn.0000019413.78751.46\|10.1074/jbc.273.49.32776\|10.1023/a:1013070128668\|10.1124/mol.54.2.342\|10.1074/jbc.273.47.30915\|10.1006/geno.1998.5639\|10.1124/mol.56.1.1 |
| Dopamine | SLC22A1 | 10.1124/mol.63.3.489\|\|10.1023/a:1013070128668\|10.1016/0014-5793(96)01030-7\|10.1038/sj.bjp.0702065\|10.1124/dmd.113.055095\|10.1124/mol.56.1.1 |
| Dopamine | SLC22A3 | 10.1074/jbc.273.49.32776 |
| Dopamine | SLC22A5 | 10.1124/mol.59.2.358\| |
| Dopamine | POU5F1 | 10.1111/j.1471-4159.2010.06738.x |
| Metoclopramide | DRD2 | 10.1016/0306-4522(95)00540-4\|10.1021/jm00073a017\|10.1254/jjp.67.45\|10.1006/phrs.1996.9999\|10.1046/j.1468-2982.1998.1809593.x\|10.1891/0730-0832.24.2.51\|10.2174/157488911795933901 |
| Metoclopramide | CHRM1 | 10.1586/eem.10.41 |
| Metoclopramide | HTR4 | 10.1677/erc-08-0190\|10.1586/eem.10.41\|10.2174/157488911795933901 |
| Metoclopramide | HTR3A | 10.1111/j.1476-5381.1987.tb10280.x\|10.1586/eem.10.41 |
| Metoclopramide | CYP2D6 | 10.3109/00498254.2013.835885\|10.1124/dmd.30.3.336\|10.1097/mcg.0b013e3182549528 |
| Metoclopramide | CYP3A4 | 10.1586/eem.10.41\|10.3109/00498254.2013.835885\|10.1503/cmaj.120951 |
| Metoclopramide | CYP1A2 | 10.1586/eem.10.41\|10.3109/00498254.2013.835885\|10.1097/mcg.0b013e3182549528 |
| Metoclopramide | ABCB1 | 10.2967/jnumed.115.164350\|10.1155/2018/7310146\|10.1002/cpt.1402 |
| Metoclopramide | ORM1 | 10.1111/j.1365-2125.1986.tb05201.x |
| Pioglitazone | PPARG | 10.1073/pnas.0912487106\|10.1210/jc.2004-0190\|10.2337/diabetes.47.4.507\|10.1021/jm950395a |
| Pioglitazone | MAOB | 10.1021/ml200196p |
| Pioglitazone | CYP2C8 | 10.1111/j.1742-7843.2009.00457.x\|10.1111/j.1742-7843.2006.pto_437.x\|10.1124/dmd.31.4.439\|10.2174/092986709789057635 |
| Pioglitazone | CYP3A4 | 10.1111/j.1742-7843.2006.pto_437.x\|10.1124/dmd.31.4.439\|10.1177/0091270002042012009\|10.2174/092986709789057635 |
| Pioglitazone | SLCO1B3 | 10.1124/dmd.32.3.291 |
| Pioglitazone | SLCO1B1 | 10.1124/dmd.32.3.291 |
| Erythromycin | MLNR | 10.1152/ajpgi.1989.257.3.g470\|10.3945/ajcn.115.113456 |
| Erythromycin | KCNH2 | 10.1016/j.bmcl.2004.06.070\|10.1023/a:1027309703313\|10.1111/bph.12575 |
| Erythromycin | CYP3A4 | 10.1046/j.1365-2125.2003.01718.x\|10.1046/j.1365-2125.1999.00073.x |
| Erythromycin | CYP3A7 | 10.1124/dmd.106.011304 |
| Erythromycin | CYP3A7-CYP3A51P | 10.1124/dmd.106.011304 |
| Erythromycin | CYP3A5 | 10.1124/dmd.32.10.\|10.2174/1389200218666170531112038 |
| Erythromycin | SLCO1B3 | 10.1111/j.1476-5381.2009.00430.x\|10.1007/978-3-642-14541-4_1\|10.1038/clpt.2008.94 |
| Erythromycin | ABCB11 | 10.1093/toxsci/kft197\|10.1124/dmd.113.054304\|10.1002/hep.24229 |
| Erythromycin | ABCB1 | 10.1016/s0014-2999(98)00607-4\|10.5414/cpp38161\|10.1128/aac.44.6.1697-1700.2000\|10.1016/j.ijpharm.2011.08.009 |
| Erythromycin | SLCO1B1 | 10.1124/dmd.104.000521\|10.1111/j.1476-5381.2009.00430.x\|10.1007/978-3-642-14541-4_1\|10.1038/clpt.2008.94\|10.1038/clpt.2012.106 |
| Erythromycin | ABCC2 | 10.1038/clpt.2011.25\|10.1007/s40262-013-0069-2\|10.1016/j.ijpharm.2011.08.009 |
| Erythromycin | SLCO1A2 | 10.1038/clpt.2008.94\|10.1124/dmd.109.028522 |
| Cangrelor | P2RY12 | 10.1185/03007995.2015.1098600 |
| Bupropion | SLC6A3 | 10.1124/jpet.102.033852\|10.1007/s00213-002-1166-3\|10.1038/sj.npp.1300036\|10.1016/s0006-3223(02)01834-6\|\|10.1093/nar/30.1.412\|10.1016/s0014-2999(97)01393-9\|10.4088/pcc.v06n0403\|10.1021/acschemneuro.7b00055\|10.1111/bcpt.12653\|10.1111/j.1471-4159.2006.04060.x |
| Bupropion | SLC6A2 | 10.1016/s0014-2999(03)02010-7\|10.1016/j.biopsych.2006.03.057\|10.1093/nar/30.1.412\|10.4088/pcc.v06n0403\|10.1021/acschemneuro.7b00055\|10.1111/j.1471-4159.2006.04060.x |
| Bupropion | HTR3A | 10.1016/j.neuropharm.2016.09.021 |
| Bupropion | CYP2B6 | 10.1177/0091270006293753\|10.1081/dmr-120001391\|\|10.1016/j.clinthera.2005.11.011\|10.1586/14737175.6.9.1249\|10.1081/dmr-120001392 |
| Bupropion | CYP2D6 | 10.1586/14737175.6.9.1249\|10.1097/01.jcp.0000162805.46453.e3\|10.1016/j.bcp.2016.11.007 |
| Bupropion | SLC22A2 | 10.1093/ntr/ntu161 |
| Bupropion | ORM1 | 10.1002/jps.2600750208 |
| Estradiol | ESR1 | 10.1016/j.mce.2006.10.013\|\|10.1016/j.steroids.2014.06.012\|10.3390/molecules18077389 |
| Estradiol | ESR2 | 10.1139/o06-144\|\|10.1016/j.steroids.2014.06.012\|10.1096/fj.13-234617 |
| Estradiol | NR1I2 | 10.1210/me.2006-0323\|10.3390/molecules18077389\| |
| Estradiol | CHRNA4 | 10.1523/jneurosci.4802-10.2011 |
| Estradiol | GPER1 | 10.1016/j.jsbmb.2006.09.017\|10.1016/j.mce.2006.12.010 |
| Estradiol | ATP6 | 10.1016/j.tiv.2010.10.012\|\|10.1007/s10863-012-9497-1 |
| Estradiol | BECN1 | 10.1016/j.tiv.2010.10.012\|10.1007/s10863-012-9497-1 |
| Estradiol | CYP3A4 | 10.1210/en.2003-0192\|10.1016/j.canlet.2004.10.007\|\|10.1124/dmd.112.046276\|10.1002/j.1552-4604.1995.tb04143.x |
| Estradiol | CYP1A2 | 10.1210/en.2003-0192\|10.1186/bcr798\|10.1371/journal.pone.0153863\|10.1177/00912709922008560 |
| Estradiol | UGT1A1 | 10.1016/j.lfs.2010.07.001\|10.1186/bcr936\|10.1124/dmd.110.035030 |
| Estradiol | CYP1A1 | 10.1210/jc.2003-032154\|10.1161/01.hyp.0000048862.28501.72 |
| Estradiol | CYP1B1 | 10.1210/jc.2003-032154\|10.1161/01.hyp.0000048862.28501.72\|10.1124/mol.113.087700 |
| Estradiol | CYP2C19 | 10.1124/mol.110.065540\|10.1007/s10549-008-0076-4\|\|10.1186/1471-2407-14-902 |
| Estradiol | CYP2C8 | 10.1124/dmd.104.002097 |
| Estradiol | CYP2C9 | 10.1021/tx970217f\|10.1002/j.1552-4604.1997.tb04781.x\| |
| Estradiol | UGT2B15 | 10.1210/en.2006-0358 |
| Estradiol | COMT | 10.1210/jc.2003-032154\|10.1093/humrep/deg059\|10.1161/01.hyp.0000048862.28501.72 |
| Estradiol | UGT1A1 | 10.1016/j.apsb.2016.04.005\|10.1124/dmd.30.11.1266\|10.1124/dmd.109.026609\| |
| Estradiol | UGT1A10 | 10.1016/j.apsb.2016.04.005\|10.1124/dmd.30.11.1266\|10.1124/dmd.109.026609\| |
| Estradiol | UGT1A3 | 10.1016/j.apsb.2016.04.005\|10.1124/dmd.30.11.1266\|10.1124/dmd.109.026609\| |
| Estradiol | UGT1A4 | 10.1016/j.apsb.2016.04.005\|10.1124/dmd.30.11.1266\|10.1124/dmd.109.026609\| |
| Estradiol | UGT1A6 | 10.1016/j.apsb.2016.04.005\|10.1124/dmd.30.11.1266\|10.1124/dmd.109.026609\| |
| Estradiol | UGT1A7 | 10.1016/j.apsb.2016.04.005\|10.1124/dmd.30.11.1266\|10.1124/dmd.109.026609\| |
| Estradiol | UGT1A8 | 10.1016/j.apsb.2016.04.005\|10.1124/dmd.30.11.1266\|10.1124/dmd.109.026609\| |
| Estradiol | UGT1A9 | 10.1016/j.apsb.2016.04.005\|10.1124/dmd.30.11.1266\|10.1124/dmd.109.026609\| |
| Estradiol | UGT2B15 | 10.1016/j.apsb.2016.04.005\|10.1124/dmd.30.11.1266\|10.1124/dmd.109.026609\| |
| Estradiol | UGT2B4 | 10.1016/j.apsb.2016.04.005\|10.1124/dmd.30.11.1266\|10.1124/dmd.109.026609\| |
| Estradiol | UGT2B7 | 10.1016/j.apsb.2016.04.005\|10.1124/dmd.30.11.1266\|10.1124/dmd.109.026609\| |
| Estradiol | SLC22A2 | 10.1038/sj.bjp.0704785\|10.1016/s0014-5793(00)01525-8 |
| Estradiol | SLC22A3 | 10.1074/jbc.273.49.32776 |
| Estradiol | ABCC10 | 10.1124/mol.63.2.351\|10.1158/0008-5472.can-03-3111 |
| Estradiol | SLC22A11 | 10.1074/jbc.275.6.4507 |
| Estradiol | ABCG2 | 10.1124/mol.64.3.610\|10.1111/j.1349-7006.2002.tb02162.x\|10.1208/s12248-014-9668-6\|10.1038/jcbfm.2010.36 |
| Estradiol | SLCO1B1 | 10.1023/a:1013077609227\|10.1053/gast.2001.21176 |
| Estradiol | ABCB1 | 10.1023/b:pham.0000033017.52484.81\|\|10.1111/j.1349-7006.2006.00300.x\|10.1016/j.bbrc.2019.09.021 |
| Estradiol | SLC22A8 | 10.1124/mol.59.5.1277 |
| Estradiol | SLCO1B3 | 10.1053/gast.2001.21176 |
| Estradiol | SLCO1C1 | 10.1210/me.2001-0309 |
| Estradiol | SHBG | 10.4067/s0034-98872006000900006\|10.1111/j.1528-1167.2006.00851.x\|10.1016/j.jsbmb.2006.10.002\|\|\|10.1002/j.1552-4604.1995.tb04143.x\|10.1016/s0300-595x(86)80024-x\|10.1210/jc.2005-0352\| |
| Estradiol | ALB | 10.1002/j.1552-4604.1995.tb04143.x |
| Estradiol | FABP2 | 10.1124/dmd.109.027656 |
| Ethinyl Estradiol | ESR1 | 10.1210/en.2006-0774\|10.1159/000097485\|10.1016/j.mce.2006.10.013\|10.1210/en.2006-1179\|10.1159/000097747\|10.1016/j.aquatox.2009.01.001\|10.1016/j.maturitas.2008.11.015\|10.1093/nar/30.1.412 |
| Ethinyl Estradiol | NR1I2 | 10.1210/me.2006-0323 |
| Ethinyl Estradiol | CYP3A4 | 10.1124/dmd.104.000182\|10.1002/cpt.1085 |
| Ethinyl Estradiol | CYP2C8 | 10.1177/0091270004270642\|10.1002/cpt.1085 |
| Ethinyl Estradiol | CYP2C9 | 10.1002/cpt.1085 |
| Ethinyl Estradiol | CYP1A2 | 10.1002/cpt.1085 |
| Ethinyl Estradiol | CYP3A5 | 10.1016/j.ejim.2017.05.019 |
| Ethinyl Estradiol | UGT1A3 | 10.1016/j.ejim.2017.05.019 |
| Ethinyl Estradiol | UGT1A1 | 10.1124/dmd.104.000794\|10.1002/cpt.1085\|10.1515/hmbci-2014-0028\| |
| Ethinyl Estradiol | UGT2B7 | 10.1016/j.ejim.2017.05.019 |
| Ethinyl Estradiol | SULT1A3 | 10.1124/dmd.32.11. |
| Ethinyl Estradiol | SULT1A4 | 10.1124/dmd.32.11. |
| Ethinyl Estradiol | SULT1A3 | 10.1124/dmd.32.11. |
| Ethinyl Estradiol | SULT1A4 | 10.1124/dmd.32.11. |
| Ethinyl Estradiol | SULT1E1 | 10.1124/dmd.32.11. |
| Ethinyl Estradiol | COMT | 10.1016/j.ejim.2017.05.019 |
| Ethinyl Estradiol | CYP2C19 | 10.1111/1440-1681.12153\|10.1046/j.1365-2125.2003.01868.x\|10.1124/dmd.109.026997 |
| Ethinyl Estradiol | ABCB11 | 10.1016/s0016-5085(00)70425-2\|10.1053/jhep.2000.8263\|10.1016/s0016-5085(00)70224-1 |
| Ethinyl Estradiol | SLC10A1 | 10.1016/s0016-5085(00)70425-2 |
| Ethinyl Estradiol | ABCC2 | 10.1006/bbrc.1998.8340\|10.1016/s0016-5085(97)70103-3 |
| Ethinyl Estradiol | ABCB1 | 10.1023/b:pham.0000033017.52484.81\|10.1053/jhep.2000.8263 |
| Ethinyl Estradiol | SLCO1A2 | 10.1016/s0016-5085(00)70425-2 |
| Ethinyl Estradiol | ALB | 10.1016/0022-4731(90)90290-9 |
| Ethinyl Estradiol | SHBG | 10.1093/toxsci/kfu231 |
| Moxifloxacin | TOP2A | 10.1093/nar/30.1.412\|10.3892/ijo_00000695\|10.1038/nsmb.1892 |
| Moxifloxacin | CYP1A2 | 10.1208/s12248-009-9127-y |
| Morphine | OPRM1 | 10.1016/j.brainres.2006.01.095\|10.1016/j.bbrc.2006.03.084\|10.1016/j.joca.2006.01.013\|10.1124/mol.106.022376\|10.1093/nar/30.1.412 |
| Morphine | OPRK1 | 10.1038/nrd2199\|10.1038/nrd2132\|\|10.1006/geno.1994.1331\|10.1016/j.neuroscience.2006.04.071 |
| Morphine | OPRD1 | 10.1016/j.brainres.2006.01.095\|10.1159/000091993\|10.1111/j.1471-4159.2006.03736.x\|10.1016/j.pain.2006.03.008\|10.1016/j.ejphar.2006.04.001\|10.1093/nar/30.1.412\|10.1016/s0301-0082(02)00008-4 |
| Morphine | LY96 | 10.1016/j.neuroscience.2010.02.011 |
| Morphine | CYP2C8 | 10.1080/0049825031000121608\|10.2217/pgs.09.82 |
| Morphine | CYP3A4 | 10.1080/0049825031000121608\|10.1248/bpb.28.2026\|10.2217/pgs.09.82 |
| Morphine | UGT2B7 | 10.1124/mol.104.007641\|10.2131/jts.28.395\|10.1124/dmd.109.030635 |
| Morphine | UGT1A1 | 10.1124/dmd.107.019281 |
| Morphine | UGT1A8 | 10.1124/dmd.107.019281 |
| Morphine | UGT2B15 | 10.1124/dmd.109.030635 |
| Morphine | UGT2B4 | 10.1124/dmd.109.030635 |
| Morphine | ABCB1 | 10.1021/jm021012t\|10.1023/a:1025001131513 |
| Morphine | ALB | 10.1213/ane.0b013e318232e922 |
| Nitric Oxide | GUCY1A2 | 10.1111/j.1471-4159.2010.06606.x |
| Nitric Oxide | MT1A | 10.1042/0264-6021:3440253 |
| Nitric Oxide | IDO1 | 10.1021/bi060143j |
| Nitric Oxide | ALDH2 | 10.1016/j.febslet.2005.09.082 |
| Nitric Oxide | CYP1A2 | 10.1074/jbc.271.15.8570\|10.1046/j.1460-9568.2003.02972.x\| |
| Nitric Oxide | CYP2B6 | 10.1016/j.freeradbiomed.2007.12.010\|10.1016/j.freeradbiomed.2017.04.015 |
| Nitric Oxide | CYP3A4 | 10.1016/j.lfs.2003.07.006 |
| Metoprolol | ADRB1 | 10.1152/ajpcell.2000.279.2.c495\|10.1016/s0014-2999(01)01113-x\|10.1016/s0014-2999(03)01431-6\|10.1016/s0009-9236(03)00068-7\|10.1016/s0009-9236(03)00224-8\|10.1093/nar/30.1.412 |
| Metoprolol | ADRB2 | 10.1592/phco.29.8.883 |
| Metoprolol | CYP2D6 | 10.1007/s002280050456\|\|10.1111/bcp.13741 |
| Metoprolol | SLC22A2 | 10.1038/sj.bjp.0703518 |
| Nitroglycerin | NPR1 | 10.1038/sj.bjp.0705365 |
| Nitroglycerin | ALDH2 | 10.1152/ajpheart.00959.2009\|10.1073/pnas.122225199 |
| Ticagrelor | P2RY12 | 10.1007/s40262-015-0290-2 |
| Ticagrelor | CYP3A4 | 10.1124/dmd.110.032250 |
| Ticagrelor | CYP2C9 | 10.1124/dmd.110.037143\|10.1007/s40262-015-0290-2 |
| Ticagrelor | ALB | 10.1016/j.jacbts.2017.01.007 |
| Vitamin C | PLOD2 | 10.1006/abbi.1997.0319 |
| Vitamin C | PHYH | 10.1093/hmg/10.18.1971 |
| Vitamin C | PLOD3 | 10.1006/abbi.1997.0319 |
| Vitamin C | BBOX1 | 10.1093/ajcn/54.6.1147s |
| Vitamin C | DBH | 10.3177/jnsv.43.491\|\|\| |
| Vitamin C | PAM | 10.1016/j.jplph.2006.12.011\|10.1007/s00425-006-0321-1\|10.1021/ja062876x\|10.1016/s0076-6879(97)79007-4 |
| Vitamin C | P3H1 | 10.1016/j.biochi.2017.12.011 |
| Vitamin C | P3H2 | 10.1016/j.biochi.2017.12.011 |
| Vitamin C | P3H3 | 10.1016/j.biochi.2017.12.011 |
| Vitamin C | P4HA1 | 10.1016/j.biochi.2017.12.011 |
| Vitamin C | OGFOD1 | 10.3389/fonc.2014.00359 |
| Vitamin C | OGFOD2 | 10.3389/fonc.2014.00359 |
| Vitamin C | ALKBH2 | 10.1073/pnas.262589799 |
| Vitamin C | ALKBH3 | 10.1073/pnas.262589799 |
| Vitamin C | KDM5D | 10.1016/j.cell.2007.02.004 |
| Vitamin C | PLOD1 | 10.1093/nar/30.1.412\|10.1006/abbi.1997.0319 |
| Vitamin C | TMLHE | 10.1111/j.1742-4658.2007.06108.x |
| Vitamin C | P4HTM | 10.1021/acs.biochem.6b00251 |
| Vitamin C | EGLN1 | 10.1016/j.freeradbiomed.2018.03.033 |
| Vitamin C | EGLN2 | 10.1089/ars.2007.1683\|10.2174/092986707781058850\|10.1016/j.freeradbiomed.2018.03.033 |
| Vitamin C | EGLN3 | 10.1016/j.freeradbiomed.2018.03.033 |
| Vitamin C | TXNRD1 | 10.3389/fphys.2015.00397 |
| Vitamin C | SLC23A1 | 10.1038/19986\|10.1146/annurev.nutr.25.050304.092647\|10.1038/sj.jid.5700572\|10.1016/j.cbpa.2006.11.025\|10.1007/s00726-007-0555-7\|10.1111/j.1600-079x.2007.00453.x\|10.1016/j.freeradbiomed.2007.05.001 |
| Vitamin C | SLC23A2 | 10.1146/annurev.nutr.25.050304.092647 |
| Vitamin C | SLC2A1 | 10.1146/annurev.nutr.25.050304.092647 |
| Vitamin C | SLC2A3 | 10.1146/annurev.nutr.25.050304.092647 |
| Vitamin C | SLC2A4 | 10.1146/annurev.nutr.25.050304.092647 |
| Vitamin C | ALB | 10.1016/s0304-4165(02)00257-x\| |
| Candesartan | AGTR1 | 10.1093/nar/30.1.412\|\|10.1016/s0895-7061(99)00264-2\|10.1291/hypres.19.75\|10.1016/j.neulet.2006.07.022\|10.1016/s0014-2999(02)02819-4\|10.1038/sj.jhh.1000997\|10.1097/01.hjh.0000220403.61493.18\|10.1517/14656560903092197\|10.1185/030079907x210723\|10.2147/vhrm.s4650 |
| Candesartan | CYP2C8 | 10.1177/0091270004270642\|10.1124/pr.115.011411 |
| Candesartan | CYP2C9 | 10.2165/00003495-199856050-00013\|10.2174/092986709789057635\|10.1081/dmr-120001392\|10.1007/s002280050731 |
| Candesartan | UGT1A3 | 10.2174/092986709789057635 |
| Candesartan | PTGS1 | 10.2174/092986709789057635 |
| Candesartan | ABCB1 | 10.1016/s0024-3205(01)01494-1 |
| Candesartan | SLCO1B1 | 10.1007/s11095-011-0564-9 |
| Acetylcysteine | GSS | 10.1007/bf01800713 |
| Acetylcysteine | SLC7A11 | 10.1503/jpn.100057 |
| Acetylcysteine | ACY1 | 10.1021/tx980018b |
| Acetylcysteine | IKBKB | 10.1016/s0014-5793(00)01464-2 |
| Acetylcysteine | CHUK | 10.1016/s0014-5793(00)01464-2 |
| Acetylcysteine | GRIN2B | 10.1016/s0166-2236(02)02245-2 |
| Acetylcysteine | GRIN1 | 10.1016/s0166-2236(02)02245-2 |
| Acetylcysteine | GRIN2A | 10.1016/s0166-2236(02)02245-2 |
| Acetylcysteine | GRIN2D | 10.1016/s0166-2236(02)02245-2 |
| Acetylcysteine | GRIN3A | 10.1016/s0166-2236(02)02245-2 |
| Acetylcysteine | SLCO1B1 | 10.1016/j.toxicon.2009.11.019 |
| Acetylcysteine | SLC22A6 | 10.1124/mol.63.3.590 |
| Ezetimibe | NPC1L1 | 10.1126/science.1093131\|10.1111/j.1399-0004.2004.00388.x\|10.1016/j.pharmthera.2004.10.011\|10.1073/pnas.0500269102\|10.1016/j.amjcard.2005.03.014\|10.1093/nar/30.1.412 |
| Ezetimibe | SOAT1 | 10.1007/s10295-009-0622-z\|10.1016/j.pharmthera.2010.02.006\|10.1053/j.gastro.2008.03.011 |
| Ezetimibe | ANPEP | 10.1074/jbc.m406309200 |
| Ezetimibe | UGT1A1 | 10.1038/clpt.2011.4\|10.2165/00003088-200544050-00002 |
| Ezetimibe | UGT1A3 | 10.1038/clpt.2011.4\|10.2165/00003088-200544050-00002 |
| Ezetimibe | UGT2B15 | 10.1124/dmd.32.3.314\|10.2165/00003088-200544050-00002 |
| Ezetimibe | UGT2B7 | 10.1124/dmd.32.3.314\|10.2165/00003088-200544050-00002 |
| Ezetimibe | CYP3A4 | 10.2165/00003088-200544050-00002 |
| Ezetimibe | CYP2C8 | 10.2165/00003088-200544050-00002 |
| Ezetimibe | ABCB1 | 10.1038/clpt.2011.4 |
| Ezetimibe | ABCC2 | 10.1038/clpt.2011.4\|10.1124/dmd.108.026146 |
| Ezetimibe | ABCC3 | 10.1124/dmd.108.026146 |
| Ezetimibe | SLCO1B1 | 10.1038/clpt.2011.4 |
| Ezetimibe | ABCG2 | 10.1124/dmd.108.026146 |
| Ezetimibe | ABCB11 | 10.1093/toxsci/kft197 |
| Benzocaine | SCN10A | 10.1038/nrd2199\|10.1038/nrd2132\|10.1161/circresaha.107.160663\|10.1161/circresaha.109.198572\|10.1124/mol.105.014803 |
| Benzocaine | CES1 | 10.1016/0021-9150(81)90106-4 |
| Benzocaine | ABCB1 | 10.1016/j.bbrc.2004.01.156 |
| Dipyridamole | PDE10A | 10.1038/nrd2199\|10.1038/nrd2132\|10.1074/jbc.274.26.18438 |
| Dipyridamole | ADA | 10.1093/nar/30.1.412 |
| Dipyridamole | PDE5A | 10.1358/mf.2004.26.10.872561\|10.1016/j.ijcard.2004.08.050\|10.1111/j.1468-2982.2006.01137.x\|10.1124/jpet.106.119057\|10.1093/nar/30.1.412 |
| Dipyridamole | PDE4A | 10.1093/nar/30.1.412 |
| Dipyridamole | RCAN1 | 10.1074/jbc.m805889200 |
| Dipyridamole | ORM1 | 10.1097/00008571-199610000-00004 |
| Dipyridamole | ABCC4 | 10.1681/asn.v133595\|10.1124/mol.63.5.1094\|10.1053/jhep.2003.50331 |
| Dipyridamole | ABCC5 | 10.1124/mol.63.5.1094 |
| Dipyridamole | ABCB1 | 10.1006/bbrc.2001.6000 |
| Dipyridamole | SLCO1B1 | 10.1021/jm300212s |
| Dipyridamole | SLCO1B3 | 10.1021/jm300212s |
| Dipyridamole | SLCO2B1 | 10.1021/jm300212s |
| Dipyridamole | ABCB11 | 10.1093/toxsci/kft197 |
| Dobutamine | ADRB1 | 10.1016/s0014-2999(02)01814-9\|10.1097/00008571-200411000-00001\|10.1016/j.jacc.2005.08.041\|10.1164/rccm.200508-1221oc\|10.1093/nar/30.1.412 |
| Dobutamine | ADRB2 | 10.1164/ajrccm.156.2.9609141 |
| Dobutamine | ESR1 | 10.1093/toxsci/kfu114 |
| Dobutamine | COMT | 10.3109/00498258609043504\|10.1124/jpet.301.1.315 |
| Ticlopidine | P2RY12 | 10.1080/09537100120058739\|\|10.1016/j.pharmthera.2005.03.009\|10.1016/j.jmgm.2006.09.006\|10.1093/nar/30.1.412 |
| Ticlopidine | CYP2C19 | 10.2174/092986709789057635\|10.1124/jpet.103.056127\|10.1016/s0009-9236(97)90054-0\|10.1053/cp.1999.v66.103277001\|10.1007/s002280100268\|10.1046/j.1365-2125.1999.00914.x |
| Ticlopidine | CYP2D6 | 10.2174/092986709789057635\|10.1046/j.1365-2125.2000.00175.x\|10.1186/s40780-017-0083-x |
| Ticlopidine | MPO | 10.2174/092986709789057635 |
| Ticlopidine | CYP2C9 | 10.1007/s002280100268\|10.1016/s0009-9236(97)90054-0\|10.1038/aps.2011.32 |
| Ticlopidine | CYP2C8 | 10.1124/pr.115.011411 |
| Ticlopidine | CYP1A2 | 10.1124/jpet.103.056127\|10.1111/j.1538-7836.2007.02338.x\|10.1081/dmr-120001392\|10.1046/j.1365-2125.2000.00175.x |
| Ticlopidine | CYP2B6 | 10.1177/0091270006293753\|10.1124/jpet.103.056127 |
| Ticlopidine | CYP2E1 | 10.1046/j.1365-2125.2000.00175.x |
| Progesterone | PGR | 10.1002/med.20083\|10.1128/mcb.00326-06\|10.1210/me.2006-0337\|10.1016/s1472-6483(10)60655-4\|10.1210/jcem.83.3.4672\|10.1002/rmb2.12088 |
| Progesterone | ESR1 | 10.1186/1477-7827-4-s1-s9\|10.1186/bcr1660\|10.1038/nature14583\|10.1111/j.1365-2826.2008.01801.x\|10.1210/endo.141.10.7734 |
| Progesterone | NR3C2 | 10.1016/0922-4106(93)90072-h\|10.1530/eje.0.1460789\|10.1152/ajpendo.1996.270.4.e601\|10.1210/endo.136.12.7588320 |
| Progesterone | CYP17A1 | 10.1002/ardp.200290006\|10.1016/j.jsbmb.2010.11.005\|10.1016/s0003-9861(02)00491-5 |
| Progesterone | OPRK1 | 10.1016/0304-3959(95)00092-5\|10.1006/hbeh.1996.0029 |
| Progesterone | ORM1 | 10.1016/j.carres.2006.07.012\|10.1016/s0008-6215(02)00165-9\|10.1016/s0304-4165(97)00043-3\|10.1016/s1570-9639(02)00465-x |
| Progesterone | NR3C1 | 10.1016/j.ajog.2007.05.024\|10.1016/0022-4731(90)90089-b\|10.1210/en.2003-0732 |
| Progesterone | AR | 10.1016/0016-5085(92)91496-q\|10.1016/s0303-7207(99)00109-4\|10.1016/j.urolonc.2008.03.021 |
| Progesterone | SHBG | 10.1093/toxsci/kfu231\|10.1016/0022-4731(84)90249-8\|10.1016/s0960-0760(97)00045-9 |
| Progesterone | ESR2 | 10.1111/j.1471-4159.2010.07038.x\|10.1111/j.1365-2826.2008.01801.x\|10.1210/endo.141.10.7734\|10.1186/bcr1660 |
| Progesterone | CYP3A5 | 10.1080/15513815.2017.1354411\|10.1016/j.ajog.2017.05.019 |
| Progesterone | CYP2C9 | 10.1006/abbi.1997.0302\|10.3389/fphar.2016.00098 |
| Progesterone | CYP3A7 | 10.1080/15513815.2017.1354411\|10.1124/dmd.109.029918 |
| Progesterone | CYP3A7-CYP3A51P | 10.1080/15513815.2017.1354411\|10.1124/dmd.109.029918 |
| Progesterone | CYP2C19 | 10.1006/abbi.1997.0302\|10.1210/jc.2008-1174\|10.1093/jb/mvj093\|10.1006/abbi.1995.0013 |
| Progesterone | CYP3A4 | 10.1016/0006-2952(96)00357-7\|10.1039/c2dt31833d\|10.1021/acs.bioconjchem.6b00604\|10.1126/science.1099736\|\|10.1124/dmd.112.046276 |
| Progesterone | CYP17A1 | 10.1074/jbc.m114.610998 |
| Progesterone | CYP1A1 | 10.1097/00008571-200008000-00005\|10.1080/004982598239290 |
| Progesterone | CYP1B1 | 10.1093/carcin/20.8.1607\|10.1016/j.pharmthera.2017.03.007\|10.1097/00008571-200112000-00007 |
| Progesterone | CYP2A6 | 10.1124/dmd.112.046276 |
| Progesterone | CYP2D6 | 10.1016/j.molbrainres.2004.06.030\|10.1248/bpb.31.348\|10.1111/bph.12652 |
| Progesterone | ABCB1 | 10.1006/bbrc.2001.6000\|10.1021/jm010126m\|\|10.1016/j.bbrc.2004.01.156\|\|10.1023/a:1013358126640\|10.1074/jbc.271.6.3163\|10.1023/b:pham.0000033017.52484.81 |
| Progesterone | SLC22A2 | 10.1038/sj.bjp.0704785\|10.1074/jbc.273.49.32776 |
| Progesterone | SLC22A1 | 10.1038/sj.bjp.0704785\|10.1074/jbc.273.49.32776\|10.1155/2013/692071 |
| Progesterone | SLC22A3 | 10.1038/sj.bjp.0704785\|10.1074/jbc.273.49.32776 |
| Progesterone | ABCB11 | 10.1023/a:1023278211849\|10.1159/000371565 |
| Progesterone | ABCC1 | 10.1006/bbrc.1999.0671 |
| Progesterone | SLC10A1 | 10.1152/ajpgi.1998.274.2.g370\|10.1159/000371565\|10.1074/jbc.m109.072140\| |
| Progesterone | SLCO1B1 | 10.2174/1875397301004010001 |
| Progesterone | ABCG2 | 10.1208/s12248-014-9668-6\|10.1007/s11095-008-9537-z\|10.1159/000354442 |
| Progesterone | SLCO1B3 | 10.1038/s41598-018-20815-1\|10.2174/1875397301004010001 |
| Sertraline | SLC6A4 | 20026626\|\|10.1007/s00213-003-1562-3\|10.1111/j.1471-4159.2004.02835.x\|10.1016/s0014-2999(97)01393-9\|10.1007/s002130000588\|\|10.1007/s11064-008-9818-2 |
| Sertraline | SLC6A3 | 10.1016/s0014-2999(97)01393-9\|\|10.1007/s00115-006-2104-0\|10.1017/s1092852900025475\|10.1177/0269881198012003021 |
| Sertraline | PGRMC1 | 10.1016/0014-2999(96)00254-3\|10.1016/j.pharmthera.2010.04.003 |
| Sertraline | SIGMAR1 | 10.1016/0014-2999(96)00254-3\|10.1016/j.pharmthera.2010.04.003 |
| Sertraline | SLC6A2 | 10.1016/s0014-2999(97)01393-9\|10.1074/jbc.m112.342212 |
| Sertraline | SLC29A4 | 10.1016/j.pharmthera.2008.10.004\|10.1016/j.bcp.2006.09.008\|10.1007/s00210-009-0479-8 |
| Sertraline | CYP3A4 | 10.1016/s0013-7006(06)76153-x\|\|10.1097/01.jcp.0000104908.75206.26\|10.1124/dmd.104.002428\|10.2147/tcrm.s49063 |
| Sertraline | CYP2D6 | 10.2165/00003088-199631060-00004\|10.1016/s0013-7006(06)76153-x\|\|10.1124/dmd.104.002428 |
| Sertraline | CYP2C19 | 10.1016/s0013-7006(06)76153-x\|\|10.1124/dmd.104.002428\|10.1111/j.1600-0773.1996.tb00206.x\|10.1007/s11096-016-0259-8 |
| Sertraline | CYP2C9 | 10.1124/dmd.104.002428\|10.1046/j.1365-2125.1997.00601.x\|10.2165/00003088-199700321-00005\|10.2165/00003088-199700321-00006 |
| Sertraline | CYP2B6 | 10.1124/dmd.104.002428\|10.1177/0091270006293753\|10.1081/dmr-120001392\|10.1007/s11096-016-0259-8\|10.1007/s13318-011-0065-6 |
| Sertraline | MAOB | 10.1124/dmd.104.002428 |
| Sertraline | MAOA | 10.1124/dmd.104.002428 |
| Sertraline | ABCB1 | 10.1124/jpet.102.046532\|10.1371/journal.pone.0056525 |
| Sertraline | ALB | 10.1016/j.cbi.2015.10.006 |
| Aspirin | PTGS1 | 10.1016/s0065-2423(06)42003-5\|10.1016/j.prostaglandins.2006.10.003\|10.1111/j.1538-7836.2007.02387.x |
| Aspirin | PTGS2 | 10.1158/1055-9965.epi-06-0346\|10.1016/s0049-3848(03)00379-7 |
| Aspirin | AKR1C1 | 10.2174/157340607782360399 |
| Aspirin | PRKAA1 | 10.1053/j.gastro.2012.02.050\|10.1126/science.1215327\|10.1016/j.tem.2013.06.002 |
| Aspirin | PRKAA2 | 10.1053/j.gastro.2012.02.050\|10.1126/science.1215327\|10.1016/j.tem.2013.06.002 |
| Aspirin | PRKAB1 | 10.1053/j.gastro.2012.02.050\|10.1126/science.1215327\|10.1016/j.tem.2013.06.002 |
| Aspirin | PRKAB2 | 10.1053/j.gastro.2012.02.050\|10.1126/science.1215327\|10.1016/j.tem.2013.06.002 |
| Aspirin | PRKAG1 | 10.1053/j.gastro.2012.02.050\|10.1126/science.1215327\|10.1016/j.tem.2013.06.002 |
| Aspirin | PRKAG2 | 10.1053/j.gastro.2012.02.050\|10.1126/science.1215327\|10.1016/j.tem.2013.06.002 |
| Aspirin | PRKAG3 | 10.1053/j.gastro.2012.02.050\|10.1126/science.1215327\|10.1016/j.tem.2013.06.002 |
| Aspirin | EDNRA | 10.1124/mol.57.4.797\|10.1124/mol.58.6.1461\|10.1124/pr.115.011833 |
| Aspirin | TP53 | 10.3892/mmr_00000132\|10.1007/s13277-015-4438-3\|10.1016/j.ejphar.2014.03.009\|10.1016/s0006-291x(02)02987-x\|10.1093/carcin/bgm101 |
| Aspirin | HSPA5 | 10.1096/fj.01-0259com |
| Aspirin | RPS6KA3 | 10.1517/14728222.2011.537656\|10.1038/23810 |
| Aspirin | TNFAIP6 | 10.1124/mol.52.3.421\|\|10.1016/j.freeradbiomed.2017.01.010\|10.1016/j.cyto.2003.11.007 |
| Aspirin | CASP1 | 10.1038/bjc.2014.271\|10.1371/journal.pone.0048208\|10.3389/fonc.2018.00679\|10.1093/emph/eow009 |
| Aspirin | CASP3 | 10.1038/bjc.2014.271\|10.1371/journal.pone.0048208 |
| Aspirin | IKBKB | 10.1038/bjc.2014.271\|10.1128/mcb.20.10.3655-3666.2000\|10.1016/j.brainres.2017.10.020\|10.1111/j.1476-5381.2011.01608.x\|10.1172/jci11914\|10.1172/jci18712 |
| Aspirin | MAPK1 | 10.1038/bjc.2014.271\|10.3892/mmr.2013.1676\|10.1007/s11010-015-2613-x\|10.1007/978-1-4615-0193-0_87 |
| Aspirin | MAPK15 | 10.1038/bjc.2014.271\|10.3892/mmr.2013.1676\|10.1007/s11010-015-2613-x\|10.1007/978-1-4615-0193-0_87 |
| Aspirin | MAPK3 | 10.1038/bjc.2014.271\|10.3892/mmr.2013.1676\|10.1007/s11010-015-2613-x\|10.1007/978-1-4615-0193-0_87 |
| Aspirin | MAPK4 | 10.1038/bjc.2014.271\|10.3892/mmr.2013.1676\|10.1007/s11010-015-2613-x\|10.1007/978-1-4615-0193-0_87 |
| Aspirin | MAPK6 | 10.1038/bjc.2014.271\|10.3892/mmr.2013.1676\|10.1007/s11010-015-2613-x\|10.1007/978-1-4615-0193-0_87 |
| Aspirin | MAPK7 | 10.1038/bjc.2014.271\|10.3892/mmr.2013.1676\|10.1007/s11010-015-2613-x\|10.1007/978-1-4615-0193-0_87 |
| Aspirin | CCND1 | 10.1074/jbc.m005545200\|10.18632/oncotarget.16325\| |
| Aspirin | MYC | 10.1074/jbc.m005545200\|10.18632/oncotarget.16325\|10.1007/s13277-015-3959-0 |
| Aspirin | PCNA | 10.1074/jbc.m005545200\|10.1016/j.cdp.2004.01.001\|10.1053/j.gastro.2012.02.050 |
| Aspirin | CYP2C19 | 10.1067/mcp.2003.14 |
| Aspirin | CYP2C9 | 10.4168/aair.2011.3.4.273 |
| Aspirin | UGT1A6 | 10.4168/aair.2011.3.4.273\|10.1093/jnci/dji066\|10.1097/01.fpc.0000236339.79916.07 |
| Aspirin | NAT2 | 10.4168/aair.2011.3.4.273\|10.1097/fpc.0000000000000062 |
| Aspirin | SLC22A6 | 10.1128/aac.02392-16 |
| Aspirin | ABCB1 | 10.1016/s0378-5173(03)00372-7\|10.1002/cpt.32\|10.1038/clpt.2014.49\|10.1159/000354497\| |
| Aspirin | SLC22A8 | 10.1128/aac.02392-16\|10.1124/dmd.113.055194 |
| Capsaicin | TRPV1 | 10.1586/ern.10.182\|10.1093/bja/aer260 |
| Capsaicin | PHB2 | 10.1016/j.bbrc.2008.12.103 |
| Capsaicin | CYP3A4 | 10.1177/1091581809360282 |
| Capsaicin | PTGS2 | 10.1016/s0024-3205(00)00890-0\|10.1111/j.1749-6632.2009.04696.x |
| Capsaicin | MPO | 10.1007/s00795-007-0374-7\|10.1016/s0024-3205(00)00890-0 |
| Capsaicin | CYP1A2 | 10.3109/00498254.2010.520044\|10.1021/tx025599q |
| Capsaicin | CYP2E1 | 10.1016/0024-3205(95)00159-4 |
| Capsaicin | MAOA | 10.1111/j.1460-9568.2008.06425.x |
| Capsaicin | DBH | 10.1111/j.1460-9568.2008.06425.x |
| Capsaicin | GLUL | 10.1097/00001756-200402090-00008 |
| Capsaicin | BCHE | 10.1515/znc-2007-9-1010 |
| Hydroxyurea | RRM1 | 10.1105/tpc.018903\|\|10.1021/bi100037b\|10.1016/j.molcel.2009.11.024 |
| Hydroxyurea | CYP2D6 | 10.1016/j.jpeds.2007.01.049 |
| Nebivolol | ADRB1 | 10.5414/cpp44344\|10.1093/nar/30.1.412\|10.1517/14656566.8.10.1539 |
| Nebivolol | ADRB2 | 10.1046/j.1365-2710.2003.00477.x |
| Nebivolol | ADRB3 | 10.5414/cpp44344\|10.1007/s40265-015-0435-5\|10.1177/000331979004100202\|10.1097/00005344-198805000-00007\|10.1097/hjh.0000000000001412 |
| Nebivolol | CYP2D6 | 10.5414/cpp44344 |
| Nebivolol | CYP3A4 | 10.1517/17425250903397381 |
| Nebivolol | CYP2C19 | 10.1124/dmd.116.071811 |
| Fluoxetine | SLC6A4 | 10.1016/j.lfs.2007.01.020\|10.1016/j.pnpbp.2007.01.020\|10.1038/sj.npp.1301368\|10.1007/s11064-007-9357-2\|10.1093/nar/30.1.412\|10.1016/s0014-2999(97)01393-9 |
| Fluoxetine | HTR2C | 10.1124/mol.107.041574\|\|10.1073/pnas.94.5.2036 |
| Fluoxetine | CHRNA2 | 10.1073/pnas.94.5.2041 |
| Fluoxetine | CHRNA3 | 10.1073/pnas.94.5.2041 |
| Fluoxetine | CHRNB4 | 10.1073/pnas.94.5.2041 |
| Fluoxetine | CKS1B | 10.1016/j.bcp.2008.02.013 |
| Fluoxetine | KCNH2 | 10.1254/jphs.fpe0040101\|10.1038/sj.bjp.0706892 |
| Fluoxetine | CYP2C19 | 10.1097/00004714-200104000-00007\|10.1046/j.0306-5251.2001.01402.x\| |
| Fluoxetine | CYP2C9 | 10.2174/157340609788185954\|10.1046/j.1365-2125.1997.00601.x\|10.1007/s002280050580 |
| Fluoxetine | CYP1A2 | 10.1016/s0013-7006(06)76153-x\|10.1046/j.1365-2125.1997.00601.x\|10.1007/s11920-012-0284-9\| |
| Fluoxetine | CYP3A4 | 10.1016/s0013-7006(06)76153-x\|10.1097/01.jcp.0000104908.75206.26\| |
| Fluoxetine | CYP2B6 | 10.2174/138920008784746346 |
| Fluoxetine | ABCB1 | 10.1124/jpet.102.046532\|10.1111/j.1476-5381.2011.01557.x |
| Regadenoson | ADORA2A | 10.1007/s12350-011-9474-9 |
| Orlistat | PNLIP | 10.1097/00041433-199902000-00002\|10.1007/s003940050040\|10.1053/beem.1999.0011\|10.1016/s0899-9007(00)00424-x\|\|10.1093/nar/30.1.412\|\|\|10.1517/14656566.6.14.2483\|10.1592/phco.20.4.270.34882\|10.2165/00003495-200666120-00012\|10.1016/s0014-2999(02)01422-x\|10.2165/00003495-199856020-00007 |
| Orlistat | LIPF | 10.1210/jc.2008-1294\|10.1517/14656566.6.14.2483\|10.1592/phco.20.4.270.34882\|10.2165/00003495-200666120-00012\|10.2165/00003495-200464240-00010\|10.1016/s0014-2999(02)01422-x |
| Orlistat | FASN | 10.1158/0008-5472.can-03-3645\|10.1074/jbc.m405061200\|10.1093/annonc/mdi239\|10.1111/j.1365-2958.2005.04717.x\|10.1021/jo060392d\|10.1093/nar/30.1.412 |
| Orlistat | CYP3A4 | 10.1016/j.ejps.2010.06.019 |
| Orlistat | PLA2G4A | 10.1016/j.atherosclerosis.2006.07.010 |
| Topiramate | GABRA1 | 10.1111/j.1528-1157.2000.tb02174.x\|10.1155/2014/891348 |
| Topiramate | SCN1A | 10.1016/s0920-1211(02)00010-4\|10.1177/08830738040190070701\| |
| Topiramate | SCN10A | 10.1016/s0920-1211(02)00010-4\|10.1177/08830738040190070701\| |
| Topiramate | SCN11A | 10.1016/s0920-1211(02)00010-4\|10.1177/08830738040190070701\| |
| Topiramate | SCN2A | 10.1016/s0920-1211(02)00010-4\|10.1177/08830738040190070701\| |
| Topiramate | SCN3A | 10.1016/s0920-1211(02)00010-4\|10.1177/08830738040190070701\| |
| Topiramate | SCN4A | 10.1016/s0920-1211(02)00010-4\|10.1177/08830738040190070701\| |
| Topiramate | SCN5A | 10.1016/s0920-1211(02)00010-4\|10.1177/08830738040190070701\| |
| Topiramate | SCN7A | 10.1016/s0920-1211(02)00010-4\|10.1177/08830738040190070701\| |
| Topiramate | SCN8A | 10.1016/s0920-1211(02)00010-4\|10.1177/08830738040190070701\| |
| Topiramate | SCN9A | 10.1016/s0920-1211(02)00010-4\|10.1177/08830738040190070701\| |
| Topiramate | GRIK1 | 10.1111/j.1749-6632.2003.tb07079.x\|\|10.1016/j.neuropharm.2004.02.010\|10.1124/jpet.109.153908 |
| Topiramate | GRIK2 | 10.1111/j.1749-6632.2003.tb07079.x\|\|10.1016/j.neuropharm.2004.02.010\|10.1124/jpet.109.153908 |
| Topiramate | GRIK3 | 10.1111/j.1749-6632.2003.tb07079.x\|\|10.1016/j.neuropharm.2004.02.010\|10.1124/jpet.109.153908 |
| Topiramate | GRIK4 | 10.1111/j.1749-6632.2003.tb07079.x\|\|10.1016/j.neuropharm.2004.02.010\|10.1124/jpet.109.153908 |
| Topiramate | GRIK5 | 10.1111/j.1749-6632.2003.tb07079.x\|\|10.1016/j.neuropharm.2004.02.010\|10.1124/jpet.109.153908 |
| Topiramate | CA2 | 10.1021/jm040124c\|10.1016/j.lfs.2007.04.018\|10.1016/j.bmcl.2006.12.099\|10.1016/s0960-894x(03)00029-5\|10.1002/med.20021 |
| Topiramate | CA4 | 10.1016/j.bmcl.2003.09.062\|10.1111/j.1528-1157.2000.tb06047.x\|10.1021/jm0109199\|10.1021/jm031057+ |
| Topiramate | CA1 | 10.1016/j.bmc.2007.08.037\|10.1111/j.1528-1157.2000.tb06047.x |
| Topiramate | CA3 | 10.1016/j.bmc.2007.08.037\|\|10.1111/j.1528-1157.2000.tb06047.x |
| Topiramate | CACNA1C | 10.2147/nedt.2006.2.4.475 |
| Topiramate | CACNA1D | 10.2147/nedt.2006.2.4.475 |
| Topiramate | CACNA1F | 10.2147/nedt.2006.2.4.475 |
| Topiramate | CACNA1S | 10.2147/nedt.2006.2.4.475 |
| Topiramate | CACNB1 | 10.2147/nedt.2006.2.4.475 |
| Topiramate | CACNB2 | 10.2147/nedt.2006.2.4.475 |
| Topiramate | CACNB3 | 10.2147/nedt.2006.2.4.475 |
| Topiramate | CACNB4 | 10.2147/nedt.2006.2.4.475 |
| Topiramate | CACNA1E | 10.2174/1874205x01610010099\|10.1111/j.0013-9580.2005.35304.x\|10.1021/acsmedchemlett.6b00176 |
| Topiramate | CYP2C19 | 10.1111/j.1528-1157.2000.tb02174.x\|10.1046/j.1528-1157.2002.41701.x |
| Topiramate | CYP3A4 | 10.1111/j.0013-9580.2003.06203.x\|10.1111/j.1472-8206.2000.tb00411.x |
| Topiramate | ABCB1 | 10.1007/s11095-009-9961-8\|10.1016/s1525-5050(02)00511-5 |
| Topiramate | PRKAA1 | 10.1038/sj.tpj.6500366\|10.1152/ajpendo.00169.2005\|10.1111/bph.12338 |
| Topiramate | PRKAA2 | 10.1038/sj.tpj.6500366\|10.1152/ajpendo.00169.2005\|10.1111/bph.12338 |
| Topiramate | PRKAB1 | 10.1038/sj.tpj.6500366\|10.1152/ajpendo.00169.2005\|10.1111/bph.12338 |
| Topiramate | PRKAB2 | 10.1038/sj.tpj.6500366\|10.1152/ajpendo.00169.2005\|10.1111/bph.12338 |
| Topiramate | PRKAG1 | 10.1038/sj.tpj.6500366\|10.1152/ajpendo.00169.2005\|10.1111/bph.12338 |
| Topiramate | PRKAG2 | 10.1038/sj.tpj.6500366\|10.1152/ajpendo.00169.2005\|10.1111/bph.12338 |
| Topiramate | PRKAG3 | 10.1038/sj.tpj.6500366\|10.1152/ajpendo.00169.2005\|10.1111/bph.12338 |
| Topiramate | ALB | 10.2165/00003088-200443120-00001 |
| Carvedilol | ADRB1 | 10.1111/j.1472-8206.1991.tb00698.x\|10.1159/000138616\|10.1002/chir.530010404\|10.1038/clpt.1994.34 |
| Carvedilol | ADRA1A | 10.1124/pr.112.007203\|10.1161/circheartfailure.108.846212 |
| Carvedilol | NDUFC2 | 10.1006/abbi.1999.1624 |
| Carvedilol | ADRB2 | 10.1345/aph.1a476\|10.1124/jpet.103.061150\|10.1111/j.1472-8206.1991.tb00698.x |
| Carvedilol | VEGFA | 10.1016/s1388-9842(01)00129-5\|10.1097/00005344-200405000-00002\|10.1016/j.cardfail.2004.06.433\|10.1007/s11373-005-3008-x |
| Carvedilol | NPPB | 10.1097/00005344-200000006-00006\|10.1016/s0735-1097(01)01269-4\|10.5414/cpp41578\|10.1016/j.amjcard.2004.05.004\|10.1016/j.ahj.2004.07.036 |
| Carvedilol | GJA1 | 10.1016/j.amjhyper.2005.08.020\| |
| Carvedilol | KCNH2 | 10.1016/s0008-6363(00)00265-0\|10.1038/sj.bjp.0706508 |
| Carvedilol | VCAM1 | 10.1038/nrd2199\|10.1038/nrd2132\|10.1161/01.atv.0000145016.69181.fa |
| Carvedilol | ADRA1D | 10.1016/j.cardiores.2004.05.014\|10.1016/j.ahj.2008.04.004 |
| Carvedilol | ADRA1B | 10.1016/j.cardiores.2004.05.014 |
| Carvedilol | ADRA2C | 10.1016/j.cardiores.2004.05.014 |
| Carvedilol | ADRA2B | 10.1016/j.cardiores.2004.05.014 |
| Carvedilol | ADRA2A | 10.1016/j.cardiores.2004.05.014 |
| Carvedilol | SELE | 10.1161/01.atv.0000145016.69181.fa |
| Carvedilol | HIF1A | 10.1016/j.cardfail.2004.06.433 |
| Carvedilol | KCNJ4 | 10.1016/j.ejphar.2011.05.067 |
| Carvedilol | KCNJ2 | 10.1016/j.ejphar.2011.05.067 |
| Carvedilol | ADRA1A | 10.1124/pr.112.007203 |
| Carvedilol | ADRA1B | 10.1124/pr.112.007203 |
| Carvedilol | ADRA1D | 10.1124/pr.112.007203 |
| Carvedilol | XDH | 10.1093/cvr/28.3.400 |
| Carvedilol | CYP2C9 | 10.1007/s13318-014-0245-2 |
| Carvedilol | CYP2D6 | 10.2174/092986709789057635 |
| Carvedilol | CYP1A2 | 10.2217/pgs-2018-0115\|10.15386/mpr-1225 |
| Carvedilol | CYP3A4 | 10.2147/dddt.s106175\| |
| Carvedilol | CYP1A1 | 10.2174/092986709789057635 |
| Carvedilol | UGT1A1 | 10.1124/dmd.104.000794 |
| Carvedilol | UGT2B4 | 10.1124/dmd.104.000794 |
| Carvedilol | UGT2B7 | 10.1124/dmd.104.000794 |
| Carvedilol | ABCB1 | 10.1006/bbrc.2001.6000\|10.1016/s0024-3205(01)01494-1\|10.1016/s0006-2952(99)00262-2\|10.5414/cpp38168\|10.1211/0022357021778998\|10.1111/j.1349-7006.2003.tb01356.x\|10.1055/s-0031-1299835 |
| Glyburide | ABCC8 | 10.2337/diabetes.51.6.1896\|10.1152/ajpcell.00083.2002\|10.1038/sj.bjp.0704801\|10.1021/jm0208121\|10.1074/jbc.m208085200\|10.1073/pnas.96.4.1268\|10.1128/aac.00617-06 |
| Glyburide | KCNJ11 | 10.2337/diabetes.51.6.1896\|10.1152/ajpcell.00083.2002\|10.1038/sj.bjp.0704801\|10.1021/jm0208121\|10.1074/jbc.m208085200\|10.1073/pnas.96.4.1268\|10.1128/aac.00617-06 |
| Glyburide | ABCC9 | 10.1038/sj.bjp.0704801\|10.1042/bj20031087\|10.1038/sj.bjp.0705718\|10.1097/00019501-200608000-00010\| |
| Glyburide | ABCB11 | 10.1053/gast.2002.36591\|10.1016/j.tiv.2004.04.014\|10.2133/dmpk.18.16 |
| Glyburide | ABCA1 | 10.1161/01.atv.0000035700.82829.2a\|10.1016/j.ejphar.2003.09.062\|10.1165/rcmb.2004-0038oc\|10.1194/jlr.m300358-jlr200\|10.1074/jbc.m413993200\|10.1083/jcb.200903124 |
| Glyburide | CPT1A | 10.1152/ajpendo.1986.251.2.e241\| |
| Glyburide | CFTR | 10.1007/s00232-001-0192-0\|10.1152/japplphysiol.00562.2002\|10.1085/jgp.20028685\|10.1016/j.bbamem.2003.09.016\|10.1124/jpet.104.074369 |
| Glyburide | TRPM4 | 10.2147/dddt.s150043 |
| Glyburide | CYP3A4 | 10.2174/092986709789057635\|10.1124/dmd.31.9.1090 |
| Glyburide | CYP2C9 | 10.1016/j.clpt.2005.06.006\|10.1002/bdd.706\|10.1124/dmd.31.9.1090 |
| Glyburide | CYP2C19 | 10.2174/092986709789057635 |
| Glyburide | CYP3A7 | 10.1016/j.bcp.2014.09.025 |
| Glyburide | CYP3A7-CYP3A51P | 10.1016/j.bcp.2014.09.025 |
| Glyburide | CYP3A5 | 10.1016/j.bcp.2014.09.025 |
| Glyburide | ABCC3 | 10.1016/j.placenta.2005.11.012 |
| Glyburide | ABCB11 | 10.1053/gast.2002.36591\|10.1023/a:1023278211849\|10.1053/jhep.2001.24171\|10.1016/s0300-483x(01)00460-7\|10.1016/s0016-5085(00)70224-1\|10.1093/toxsci/kft197 |
| Glyburide | ABCB1 | 10.1007/s004240050829 |
| Glyburide | ABCC1 | 10.1038/sj.bjp.0703863\|10.1016/j.placenta.2005.11.012 |
| Glyburide | SLC15A1 | 10.1038/sj.bjp.0702895 |
| Glyburide | SLCO1A2 | 10.1023/a:1014264614637\|10.1016/j.ejps.2012.08.017 |
| Glyburide | SLC15A2 | 10.1038/sj.bjp.0702895 |
| Glyburide | SLC22A6 | 10.1016/s0014-2999(00)00324-1 |
| Glyburide | ABCC2 | 10.1016/j.placenta.2005.11.012\|10.1038/sj.bjp.0703863 |
| Glyburide | ABCG2 | 10.1016/j.placenta.2005.11.012\|10.1124/dmd.109.030791 |
| Glyburide | SLCO2B1 | 10.1124/dmd.104.002337 |
| Glyburide | ALB | 10.1111/j.2042-7158.1974.tb09280.x\|10.1002/jps.2600630416\|10.1016/0006-2952(76)90365-8 |
| Lidocaine | SCN10A | 10.1073/pnas.0601819103\|10.1085/jgp.200810103\|10.1371/journal.pcbi.1000818 |
| Lidocaine | SCN9A | 10.1113/jphysiol.2006.127027\|10.1085/jgp.200810103\|10.1371/journal.pcbi.1000818 |
| Lidocaine | SCN5A | 10.1016/j.ijcard.2007.02.007\|10.1111/j.1540-8167.2006.00386.x\|10.1038/sj.bjp.0706897\|10.1093/humrep/del271\|10.1085/jgp.200810103\|10.1371/journal.pcbi.1000818 |
| Lidocaine | EGFR | 10.1213/01.ane.0000198330.84341.35 |
| Lidocaine | SCN4A | 10.1038/sj.bjp.0705594 |
| Lidocaine | ORM1 | 10.1097/00008571-199610000-00004 |
| Lidocaine | ORM2 | 10.1097/00008571-199610000-00004 |
| Lidocaine | CYP3A4 | 10.1159/000443332\|10.1038/clpt.1994.94 |
| Lidocaine | CYP1A2 | 10.2174/092986709789378198\|\|\|10.1016/j.clpt.2003.09.007 |
| Lidocaine | CYP2C9 | 10.1081/dmr-120001392\| |
| Lidocaine | CYP2C8 | 10.1081/dmr-120001392 |
| Lidocaine | CYP2A6 | 10.1081/dmr-120001392 |
| Lidocaine | CYP2B6 | 10.1016/j.apsb.2016.07.016 |
| Lidocaine | SLC22A5 | 10.1124/mol.59.2.358 |
| Lidocaine | ABCB1 | 10.1021/tx010125x\|10.1016/j.bbrc.2004.01.156\|10.1097/cad.0000000000000455 |
| Atropine | CHRM1 | 10.1007/bf02244985\|10.1038/nrd2199\|10.1038/nrd2132 |
| Atropine | CHRM2 | 10.1016/j.brainres.2005.07.042\|10.1111/j.1742-4658.2006.05542.x\|10.1124/jpet.104.073767\|10.1016/j.ejphar.2007.04.048\|10.1124/jpet.105.094383\|10.1007/bf02244985 |
| Atropine | CHRM3 | 10.1007/bf02244985\|10.1038/nrd2199\|10.1038/nrd2132 |
| Atropine | CHRM4 | 10.1007/bf02244985\|10.1038/nrd2199\|10.1038/nrd2132 |
| Atropine | CHRM5 | 10.1007/bf02244985\|10.1038/nrd2199\|10.1038/nrd2132\|10.1093/nar/30.1.412 |
| Atropine | GLRA1 | 10.1016/j.bcp.2003.12.037 |
| Atropine | CHRNA4 | 10.1016/j.ejphar.2004.12.037 |
| Atropine | CHRNB2 | 10.1016/j.ejphar.2004.12.037 |
| Atropine | ABCB11 | 10.1093/toxsci/kft197 |
| Glycerol | PLA2G2E | 10.1038/nrd2199\|10.1038/nrd2132\|10.1093/nar/28.1.235 |
| Glycerol | ISYNA1 | 10.1038/nrd2199\|10.1038/nrd2132\|10.1093/nar/28.1.235 |
| Glycerol | ADH1B | 10.1038/nrd2199\|10.1038/nrd2132\|10.1093/nar/28.1.235 |
| Glycerol | ITPR1 | 10.1038/nrd2199\|10.1038/nrd2132\|10.1093/nar/28.1.235 |
| Glycerol | PAEP | 10.1038/nrd2199\|10.1038/nrd2132\|10.1093/nar/28.1.235 |
| Glycerol | NAGA | 10.1038/nrd2199\|10.1038/nrd2132\|10.1093/nar/28.1.235 |
| Glycerol | ARF1 | 10.1038/nrd2199\|10.1038/nrd2132\|10.1093/nar/28.1.235 |
| Glycerol | PAPSS1 | 10.1038/nrd2199\|10.1038/nrd2132\|10.1093/nar/28.1.235 |
| Glycerol | ALDH1A1 | 10.1128/aem.00848-16 |
| Glycerol | AQP7 | 10.1016/j.orcp.2015.12.001 |
| Glycerol | AQP9 | 10.1016/j.orcp.2015.12.001 |
| Aripiprazole | HTR1A | 10.1016/s0014-2999(02)01532-7\|10.1007/s00213-003-1677-6\|10.1016/j.ejphar.2003.10.025\|10.2165/00003495-200464150-00010\|10.1016/j.brainres.2005.02.072\|10.1007/s40263-015-0278-3 |
| Aripiprazole | DRD2 | 10.2152/jmi.52.284\|10.1016/s0014-2999(96)00920-x\|10.1016/j.ejphar.2006.07.008\|10.1002/hup.806\|10.1517/13543784.16.6.771\|10.1093/nar/30.1.412\|10.1007/s40263-015-0278-3 |
| Aripiprazole | HTR2A | 10.1016/j.pnpbp.2003.09.010\|10.1007/s00213-006-0621-y\|10.1007/s00213-007-0698-y\|10.1007/s40263-015-0278-3 |
| Aripiprazole | ADRA1A | 10.1038/sj.mp.4002066\|10.1007/s40263-015-0278-3 |
| Aripiprazole | ADRA1B | 10.1038/sj.mp.4002066\|10.1007/s40263-015-0278-3 |
| Aripiprazole | DRD3 | 10.1038/sj.mp.4002066\|10.1007/s40263-015-0278-3 |
| Aripiprazole | HTR1D | 10.1038/sj.mp.4002066\|10.1007/s40263-015-0278-3 |
| Aripiprazole | HTR7 | 10.1038/sj.mp.4002066\|10.1007/s40263-015-0278-3 |
| Aripiprazole | ADRA2A | 10.1038/sj.mp.4002066\|10.1007/s40263-015-0278-3 |
| Aripiprazole | ADRA2C | 10.1038/sj.mp.4002066\|10.1007/s40263-015-0278-3 |
| Aripiprazole | HRH1 | 10.1038/sj.mp.4002066\|10.1007/s40263-015-0278-3 |
| Aripiprazole | HTR1B | 10.1038/sj.mp.4002066\|10.1007/s40263-015-0278-3 |
| Aripiprazole | HTR2C | 10.1038/sj.mp.4002066\|10.1007/s40263-015-0278-3 |
| Aripiprazole | HTR3A | 10.1038/sj.mp.4002066 |
| Aripiprazole | HTR6 | 10.1038/sj.mp.4002066\|10.1007/s40263-015-0278-3 |
| Aripiprazole | DRD1 | 10.1038/sj.mp.4002066\|10.1007/s40263-015-0278-3 |
| Aripiprazole | DRD4 | 10.1038/sj.mp.4002066\|10.1007/s40263-015-0278-3 |
| Aripiprazole | ADRA2B | 10.1038/sj.mp.4002066\|10.1007/s40263-015-0278-3 |
| Aripiprazole | HTR1E | 10.1038/sj.mp.4002066\|10.1007/s40263-015-0278-3 |
| Aripiprazole | DRD5 | 10.1038/sj.mp.4002066\|10.1007/s40263-015-0278-3 |
| Aripiprazole | HTR2B | 10.1007/s40263-015-0278-3 |
| Aripiprazole | ADRB1 | 10.1007/s40263-015-0278-3 |
| Aripiprazole | ADRB2 | 10.1007/s40263-015-0278-3 |
| Aripiprazole | HRH2 | 10.1007/s40263-015-0278-3 |
| Aripiprazole | HRH3 | 10.1007/s40263-015-0278-3 |
| Aripiprazole | HRH4 | 10.1007/s40263-015-0278-3 |
| Aripiprazole | CHRM1 | 10.1007/s40263-015-0278-3 |
| Aripiprazole | CHRM2 | 10.1007/s40263-015-0278-3 |
| Aripiprazole | CHRM3 | 10.1007/s40263-015-0278-3 |
| Aripiprazole | CHRM4 | 10.1007/s40263-015-0278-3 |
| Aripiprazole | CHRM5 | 10.1007/s40263-015-0278-3 |
| Aripiprazole | OPRK1 | 10.1007/s40263-015-0278-3 |
| Aripiprazole | OPRM1 | 10.1007/s40263-015-0278-3 |
| Aripiprazole | OPRD1 | 10.1007/s40263-015-0278-3 |
| Aripiprazole | GRIN1 | 10.1007/s40263-015-0278-3 |
| Aripiprazole | GRIN2A | 10.1007/s40263-015-0278-3 |
| Aripiprazole | GRIN2B | 10.1007/s40263-015-0278-3 |
| Aripiprazole | GRIN2C | 10.1007/s40263-015-0278-3 |
| Aripiprazole | GRIN2D | 10.1007/s40263-015-0278-3 |
| Aripiprazole | GRIN3A | 10.1007/s40263-015-0278-3 |
| Aripiprazole | GRIN3B | 10.1007/s40263-015-0278-3 |
| Aripiprazole | SLC6A3 | 10.1007/s40263-015-0278-3 |
| Aripiprazole | SLC6A4 | 10.1007/s40263-015-0278-3 |
| Aripiprazole | CYP3A4 | 10.1097/01.ftd.0000249944.42859.bf\|10.1007/s00228-011-1094-4 |
| Aripiprazole | CYP2D6 | 10.1111/j.1742-7843.2007.00017.x\|10.2165/00003495-200464150-00010\|10.2133/dmpk.20.55 |
| Amisulpride | DRD2 | 10.1002/hup.320\|10.2165/00023210-200620050-00004\| |
| Amisulpride | DRD3 | 10.1002/hup.320\|10.2165/00023210-200620050-00004\| |
| Amisulpride | HTR7 | 10.1007/s00213-009-1521-8 |
| Amisulpride | HTR2A | 10.1080/00207450490430552 |
| Adenosine | ADORA2A | 10.2174/156802610791268756\|10.2174/156802610791268729\|10.1016/j.intimp.2006.12.001\|10.1124/jpet.105.096016\|10.1124/jpet.109.157651\|10.1111/j.1476-5381.2009.00614.x\|10.1161/circulationaha.107.694596\|10.4049/jimmunol.178.9.5921 |
| Adenosine | ADORA2B | 10.2174/156802610791268756\|10.2174/156802610791268729\|10.1016/j.intimp.2006.12.001\|10.1124/jpet.105.096016\|10.1111/j.1476-5381.2009.00614.x\|10.1161/circulationaha.107.694596\|10.4049/jimmunol.178.9.5921 |
| Adenosine | ADORA3 | 10.1093/nar/30.1.412\|10.2174/156802610791268756\|10.2174/156802610791268729\|10.1016/j.intimp.2006.12.001\|10.1124/jpet.105.096016\|10.1111/j.1476-5381.2009.00614.x\|10.1161/circulationaha.107.694596\|10.4049/jimmunol.178.9.5921 |
| Adenosine | ADORA1 | 10.2174/156802610791268756\|10.2174/156802610791268729\|10.1016/j.intimp.2006.12.001\|10.1111/j.1476-5381.2009.00614.x\|10.1161/circulationaha.107.694596\|10.4049/jimmunol.178.9.5921 |
| Adenosine | ADK | 10.1016/j.tips.2006.10.008 |
| Adenosine | SLC28A3 | 10.1038/sj.tpj.6500303 |
| Ethanol | GABRA1 | 10.1038/nrd2199\|10.1038/nrd2132\|10.1016/j.alcohol.2007.04.011\|10.1093/nar/30.1.412\|10.1124/jpet.110.178244\| |
| Ethanol | GRIN3A | 10.1038/nrd2199\|10.1038/nrd2132\|10.4161/chan.5.3.14856\|10.1016/j.alcohol.2010.08.015 |
| Ethanol | GLRA1 | 10.1038/nrd2199\|10.1038/nrd2132\|10.1016/j.neuropharm.2010.10.023\|10.1016/j.neuropharm.2010.08.007\|10.1074/jbc.273.6.3314 |
| Ethanol | GLRA2 | 10.1038/nrd2199\|10.1038/nrd2132\|10.1074/jbc.273.6.3314\|10.1016/j.neuropharm.2010.08.007 |
| Ethanol | CACNB1 | 10.1124/jpet.103.055137 |
| Ethanol | HTR3A | 10.1124/pr.108.000430 |
| Ethanol | GABRA2 | 10.1016/j.alcohol.2007.04.011\| |
| Ethanol | CHRNA10 | 10.3389/fpsyt.2013.00029\|10.1124/pr.108.000430 |
| Ethanol | CACNA1C | 10.1124/jpet.103.055137 |
| Ethanol | GABRA5 | 10.1016/j.alcohol.2007.04.011\| |
| Ethanol | GABRA4 | 10.1016/j.alcohol.2007.04.011\| |
| Ethanol | GABRA3 | 10.1016/j.alcohol.2007.04.011\| |
| Ethanol | GRIA1 | 10.1124/pr.108.000430 |
| Ethanol | CACNG1 | 10.1124/jpet.103.055137 |
| Ethanol | CHRNA2 | 10.3389/fpsyt.2013.00029\|10.1124/pr.108.000430 |
| Ethanol | GABRA6 | 10.1016/j.alcohol.2007.04.011\| |
| Ethanol | GRIA2 | 10.1124/pr.108.000430 |
| Ethanol | GRIA4 | 10.1124/pr.108.000430 |
| Ethanol | GRIA3 | 10.1124/pr.108.000430 |
| Ethanol | CHRNA4 | 10.3389/fpsyt.2013.00029\|10.1124/pr.108.000430 |
| Ethanol | CHRNB2 | 10.3389/fpsyt.2013.00029\|10.1124/pr.108.000430 |
| Ethanol | KCNJ3 | 10.1152/physrev.00013.2008\|10.1073/pnas.1311406110 |
| Ethanol | KCNJ6 | 10.1152/physrev.00013.2008\|10.1073/pnas.1311406110 |
| Ethanol | VCAM1 | 10.1073/pnas.0707815105 |
| Ethanol | SLC29A1 | 10.1111/j.1530-0277.2009.00897.x |
| Ethanol | KCNJ5 | 10.1152/physrev.00013.2008\|10.1073/pnas.1311406110 |
| Ethanol | CHRFAM7A | 10.3389/fpsyt.2013.00029\|10.1124/pr.108.000430 |
| Ethanol | CHRNA7 | 10.3389/fpsyt.2013.00029\|10.1124/pr.108.000430 |
| Ethanol | CHRNA9 | 10.3389/fpsyt.2013.00029\|10.1124/pr.108.000430 |
| Ethanol | GABRB1 | 10.1016/j.alcohol.2007.04.011\| |
| Ethanol | GABRB3 | 10.1016/j.alcohol.2007.04.011\| |
| Ethanol | GABRB2 | 10.1016/j.alcohol.2007.04.011\| |
| Ethanol | CACNA1S | 10.1124/jpet.103.055137 |
| Ethanol | CACNA1D | 10.1124/jpet.103.055137 |
| Ethanol | CHRNB4 | 10.3389/fpsyt.2013.00029\|10.1124/pr.108.000430 |
| Ethanol | CHRNA3 | 10.3389/fpsyt.2013.00029\|10.1124/pr.108.000430 |
| Ethanol | CHRNA5 | 10.3389/fpsyt.2013.00029\|10.1124/pr.108.000430 |
| Ethanol | CHRNA6 | 10.3389/fpsyt.2013.00029\|10.1124/pr.108.000430 |
| Ethanol | CHRNB3 | 10.3389/fpsyt.2013.00029\|10.1124/pr.108.000430 |
| Ethanol | GABRG1 | 10.1016/j.alcohol.2007.04.011\| |
| Ethanol | GABRG3 | 10.1016/j.alcohol.2007.04.011\| |
| Ethanol | GABRE | 10.1016/j.alcohol.2007.04.011\| |
| Ethanol | GABRP | 10.1016/j.alcohol.2007.04.011\| |
| Ethanol | GABRQ | 10.1016/j.alcohol.2007.04.011\| |
| Ethanol | GABRD | 10.1016/j.alcohol.2007.04.011\| |
| Ethanol | SLC29A2 | 10.1111/j.1530-0277.2009.00897.x |
| Ethanol | HTR3E | 10.1124/pr.108.000430 |
| Ethanol | HTR3B | 10.1124/pr.108.000430 |
| Ethanol | HTR3D | 10.1124/pr.108.000430 |
| Ethanol | HTR3C | 10.1124/pr.108.000430 |
| Ethanol | CACNG2 | 10.1124/jpet.103.055137 |
| Ethanol | KCNJ9 | 10.1152/physrev.00013.2008\|10.1073/pnas.1311406110 |
| Ethanol | L1CAM | 10.1073/pnas.0707815105 |
| Ethanol | CYP1A2 | 10.1081/dmr-120001392 |
| Ethanol | CYP2B6 | 10.1081/dmr-120001392\| |
| Ethanol | CYP2C9 | 10.1248/bpb.32.517\|10.1002/prp2.324 |
| Ethanol | CYP2C19 | 10.1081/dmr-120001392\| |
| Ethanol | CYP2E1 | 10.1081/dmr-120001391\|10.1081/dmr-120001392\|10.1159/000070014\|10.1111/j.1530-0277.1996.tb01722.x\|10.1006/bbrc.1994.2774\|10.1067/mcp.2000.106574 |
| Ethanol | CYP3A4 | 10.1081/dmr-120001392\|\|10.1097/01.alc.0000071738.53337.f4\|10.1006/abbi.1995.1495\|\|10.1211/0022357043950\| |
| Ethanol | ADH1A | 10.1016/j.cbi.2011.02.008\|10.1111/j.1530-0277.2010.01319.x\|\|10.1007/s10620-008-0422-8 |
| Ethanol | ADH1B | 10.1016/j.cbi.2011.02.008\|10.1111/j.1530-0277.2010.01319.x\|\|10.1007/s10620-008-0422-8 |
| Ethanol | ADH1C | 10.1016/j.cbi.2011.02.008\|10.1111/j.1530-0277.2010.01319.x\|\|10.1007/s10620-008-0422-8 |
| Ethanol | ADH5 | 10.1111/j.1530-0277.2006.00139.x |
| Ethanol | ADH4 | 10.1111/j.1530-0277.2006.00139.x |
| Ethanol | ADH7 | 10.1111/j.1530-0277.2006.00139.x |
| Ethanol | ADH6 | 10.1111/j.1530-0277.2006.00139.x |
| Ethanol | AKR1A1 | 10.1111/j.1530-0277.2006.00139.x |
| Salicylic acid | PTGS1 | 10.1016/j.neulet.2003.11.038\|10.1163/156856003322699573\|10.1152/ajprenal.00050.2005\|10.1081/jas-51326\|10.1213/01.ane.0000189102.09347.2e\|10.1038/newbio231232a0\|10.1093/nar/30.1.412 |
| Salicylic acid | PTGS2 | 10.1163/156856003322699573\|10.1078/0944-7113-00338\|10.1081/iph-120029946\|10.1055/s-2003-40668\|10.1023/a:1011223814666\|10.1038/newbio231232a0\|10.2337/db06-0789 |
| Salicylic acid | AKR1C1 | 10.2174/157340607782360399 |
| Salicylic acid | CYP2C9 | 10.2174/092986709789057635\|10.1016/j.ejps.2015.03.015 |
| Salicylic acid | SLC22A6 | 10.1006/abio.2000.4633\|10.1124/jpet.102.034330\|10.1006/bbrc.1998.9978\|10.1016/s0014-2999(00)00837-2\| |
| Salicylic acid | SLC22A10 | 10.1152/ajprenal.00012.2004 |
| Salicylic acid | SLC22A8 | 10.1124/mol.59.5.1277\|10.1124/jpet.102.034330\|10.1124/jpet.103.063370 |
| Salicylic acid | SLC22A11 | 10.1074/jbc.275.6.4507 |
| Salicylic acid | SLCO2B1 | 10.1124/dmd.104.002337 |
| Salicylic acid | SLC16A1 | 10.1211/0022357991776804 |
| Salicylic acid | SLC22A7 | 10.1124/mol.62.1.7\|10.1016/s0014-5793(98)00585-7\| |
| Salicylic acid | ALB | 10.1002/(sici)1520-636x(1997)9:4<335::aid-chir4>3.0.co;2-c\|\|10.1016/j.jbbm.2007.01.009\| |
| Simvastatin | HMGCR | 10.1194/jlr.m200002-jlr200\|10.1093/nar/30.1.412\|10.1124/dmd.30.12.1400\|10.1016/s0731-7085(03)00023-2\|10.1067/mlc.2003.31\|\|10.1021/bi050905v |
| Simvastatin | ITGAL | 10.1073/pnas.0305149101\|10.1038/89058\|10.1146/annurev.pharmtox.45.120403.095748\|10.1016/j.jneuroim.2006.05.029 |
| Simvastatin | HDAC2 | 10.1158/0008-5472.can-07-5807 |
| Simvastatin | CYP3A4 | 10.1016/j.clpt.2006.09.003\|10.1002/bdd.249\|10.2174/092986709789057635\|10.1124/dmd.30.12.1512\|10.1038/clpt.2012.163\|10.1177/0091270006295063\|10.1007/s11095-007-9519-6 |
| Simvastatin | CYP3A5 | 10.1177/0091270006295063\|10.1007/s11095-007-9519-6 |
| Simvastatin | CYP2C8 | 10.1016/j.clpt.2006.09.003\|10.1111/j.1742-7843.2005.pto_134.x\|10.2217/pgs.09.82\|10.1177/0091270004270642 |
| Simvastatin | CYP2C9 | 10.1002/bdd.249\|10.1007/s002280050094\|10.1007/s11095-007-9519-6 |
| Simvastatin | CYP2D6 | 10.2165/00129784-200404040-00005 |
| Simvastatin | CYP2B6 | 10.2174/092986709789057635\|10.1177/0091270006293753\|10.1124/dmd.30.12.1400 |
| Simvastatin | UGT1A1 | 10.2147/pgpm.s86013 |
| Simvastatin | UGT1A3 | 10.2147/pgpm.s86013 |
| Simvastatin | UGT2B7 | 10.2147/pgpm.s86013 |
| Simvastatin | CYP2C19 | 10.2147/pgpm.s86013 |
| Simvastatin | ABCB1 | 10.1023/a:1011036428972\|10.1006/bbrc.2001.6000\|10.1023/b:pham.0000041466.84653.8c\|10.1002/ijc.24885\|10.2147/pgpm.s86013 |
| Simvastatin | SLCO1A2 | 10.1074/jbc.274.52.37161 |
| Simvastatin | SLCO1B1 | 10.1074/jbc.274.52.37161\|10.1097/01.fpc.0000170913.73780.5f\|10.2147/pgpm.s86013\|10.1038/clpt.2012.163\|10.1097/01.fpc.0000230416.82349.90 |
| Simvastatin | ABCC2 | 10.1038/tpj.2011.59\|10.2147/pgpm.s86013 |
| Simvastatin | ABCB11 | 10.1093/toxsci/kft197 |
| Simvastatin | SLCO2B1 | 10.2147/pgpm.s86013 |
| Simvastatin | SLCO1B3 | 10.2147/pgpm.s86013 |
| Lisinopril | ACE | 10.1016/j.febslet.2007.06.048\|10.1093/nar/30.1.412\|10.1093/ajhp/57.suppl_1.s3\|10.2165/00003088-200241030-00005\|10.1016/s0014-5793(98)00069-6 |
| Lisinopril | REN | 10.1161/circresaha.116.303587 |
| Lisinopril | SLC15A1 | 10.1124/jpet.108.143339\|10.1002/jps.22277 |
| Lisinopril | SLC15A2 | 10.1124/jpet.108.143339 |
| Valsartan | AGTR1 | 10.1093/nar/30.1.412\|10.1097/01.asn.0000146686.35541.29\|10.1111/j.1476-5381.1993.tb13877.x\|10.1016/s0895-7061(02)03134-5\|10.1016/0167-0115(95)00085-p\|10.1097/00004872-200206000-00028 |
| Valsartan | CYP2C9 | 10.1080/00498250500158175\|10.2133/dmpk.22.267\|10.1124/dmd.112.046292\| |
| Valsartan | SLCO1B3 | 10.1007/s10928-009-9139-3\|10.1002/cbdv.200900116\|10.1080/00498254.2017.1295171\|10.3390/ijms19030855 |
| Valsartan | SLCO1B1 | 10.1007/s10928-009-9139-3\|10.1002/cbdv.200900116\|10.1007/s11095-011-0564-9\|10.3390/ijms19030855\|10.1080/00498254.2017.1295171 |
| Valsartan | ABCC2 | 10.1124/dmd.105.008938\|10.1080/00498254.2017.1295171 |
| Doxazosin | ADRA1A | 10.1139/y04-098\|10.1111/j.1474-8673.1996.tb00352.x\|10.1097/01.ju.0000097026.43866.cc\|10.1007/pl00005014\|10.1111/j.1464-410x.2005.05812.x\|10.1038/ijir.2008.51\|10.1016/j.ahj.2006.05.017\|10.1159/000052316\|10.1111/j.1442-2042.2007.01956.x\| |
| Doxazosin | ADRA1D | 10.1111/j.1442-2042.2007.01956.x |
| Doxazosin | KCNH2 | 10.1007/s00210-004-0931-8\|10.1016/j.ejphar.2007.10.051\|10.1038/cddis.2011.77 |
| Doxazosin | KCNH6 | 10.1007/s00210-004-0931-8\|10.1016/j.ejphar.2007.10.051\|10.1038/cddis.2011.77 |
| Doxazosin | KCNH7 | 10.1007/s00210-004-0931-8\|10.1016/j.ejphar.2007.10.051\|10.1038/cddis.2011.77 |
| Doxazosin | ADRA1B | 10.1111/j.1442-2042.2007.01956.x\| |
| Doxazosin | CYP2C19 | 10.1093/cid/cit673 |
| Doxazosin | CYP2D6 | 10.1093/cid/cit673 |
| Doxazosin | CYP3A4 | 10.1093/cid/cit673 |
| Doxazosin | ABCB1 | 10.1016/s0024-3205(01)01494-1\|10.3727/096504009789745601 |
| Doxazosin | SLC22A1 | 10.1007/s40262-015-0270-6\|10.2337/db14-1388 |
| Amiloride | SCNN1A | 10.1152/ajprenal.00094.2003\|10.1074/jbc.m401143200\|10.1165/rcmb.2007-0055oc\|10.1093/nar/30.1.412 |
| Amiloride | SCNN1B | 10.1152/ajprenal.00366.2001\|10.1152/ajplung.00332.2004\|10.1080/01902140290103062\|10.1161/hy02t2.102959\|10.1291/hypres.27.333 |
| Amiloride | SCNN1G | 10.1210/jcem.86.5.7449\|10.1161/01.hyp.38.1.86\|10.1080/01902140290103062\|10.1515/jpem.2002.15.9.1557\| |
| Amiloride | SCNN1D | 10.1074/jbc.m400274200\|10.1074/jbc.m312012200\|10.1074/jbc.m512293200\|10.1124/mol.104.010850\|10.1124/jpet.105.092775 |
| Amiloride | ASIC2 | 10.1074/jbc.m300991200\|\|10.1046/j.0022-7722.2003.00062.x\|10.1523/jneurosci.3196-04.2004\|10.1074/jbc.m603100200 |
| Amiloride | ASIC1 | 10.1016/s0306-4522(02)00409-8\|10.1523/jneurosci.2619-04.2004\|10.1523/jneurosci.2894-04.2005\|10.1074/jbc.m507123200\|10.1007/s00232-005-0840-x |
| Amiloride | SLC9A1 | 10.1172/jci9207\|10.1016/s0303-7207(01)00639-6\|\|10.1113/jphysiol.2003.046482\|10.1152/ajpgi.00092.2003 |
| Amiloride | PLAU | 10.4049/jimmunol.167.6.3406\|10.3892/ijmm.8.4.365\|10.1016/s0167-4889(02)00255-0\|10.1139/y03-066\|10.1016/j.acthis.2004.11.005\|10.1093/nar/30.1.412 |
| Amiloride | SLC22A2 | 10.1023/a:1013070128668 |
| Amiloride | SLC22A4 | 10.1016/s0005-2736(00)00189-9 |
| Lercanidipine | CACNG1 | 10.1038/nrd2199\|10.1038/nrd2132\|10.1517/17425250903085135 |
| Lercanidipine | CYP3A4 | 10.1081/dmr-120001392\|10.1055/s-0031-1299873\| |
| Lercanidipine | CYP2D6 | 10.1081/dmr-120001392 |
| Moxonidine | ADRA2A | 10.1038/sj.bjp.0702429\|10.1196/annals.1304.051\| |
| Pindolol | ADRB1 | 10.1093/nar/30.1.412\|10.1007/s00210-003-0835-z\|10.1016/0306-3623(91)90597-y\|10.1016/0014-2999(90)90344-6\|10.1016/0165-1838(96)00025-2\|10.1097/00004872-198812040-00157 |
| Pindolol | ADRB2 | 10.1016/j.jmgm.2006.02.008\|10.3109/00365519109104578\|10.1111/j.1365-2125.1991.tb05603.x\|10.1016/0306-3623(91)90597-y\|10.1111/j.2042-7158.1990.tb06590.x\|10.1093/nar/30.1.412 |
| Pindolol | HTR1A | 10.1016/s0893-133x(98)00129-8\|10.1016/s0006-3223(98)00354-0\|10.1016/s0893-133x(99)00035-4\|10.1007/s002130051009\|\|10.1093/nar/30.1.412 |
| Pindolol | HTR1B | 10.1016/s0028-3908(99)00192-6\|10.1007/bf00964812\|10.1111/j.1471-4159.1989.tb07357.x\|10.1111/j.1471-4159.1988.tb01176.x\| |
| Pindolol | ADRB3 | 10.1007/s00210-003-0860-y\| |
| Pindolol | CYP2D6 | 10.1016/0006-2952(92)90333-e\|10.1016/0024-3205(91)90341-8 |
| Telmisartan | AGTR1 | 10.1093/nar/30.1.412\|10.1097/00004872-199917020-00015\|10.1097/00005344-200107000-00015\|\|10.1016/s0300-9572(97)00023-3\|10.1016/j.febslet.2004.09.027\|10.2165/00003495-200161100-00009\|10.2165/00003495-199856060-00007\|10.2147/vhrm.s7857\|10.1210/en.2008-0502\|10.1016/j.cardiores.2006.07.014\|10.1038/ncpcardio0805\|10.1016/j.mehy.2004.09.015\|10.2174/156652407781387073 |
| Telmisartan | PPARG | 10.1210/en.2008-0502\|10.1016/j.cardiores.2006.07.014\|10.1038/ncpcardio0805\|10.1016/j.mehy.2004.09.015\|10.1007/s00592-005-0176-0\|10.2174/156652407781387073\|10.1016/j.mehy.2005.07.018 |
| Telmisartan | CYP2C19 | 10.1371/journal.pone.0058135 |
| Telmisartan | UGT1A3 | 10.1097/fpc.0b013e3283489ce2\|10.1097/fpc.0b013e3283482502\|10.1124/dmd.111.043984 |
| Telmisartan | ABCB1 | 10.1002/bdd.699 |
| Telmisartan | ABCC2 | 10.1002/bdd.699 |
| Telmisartan | ABCG2 | 10.1002/bdd.699 |
| Telmisartan | ABCB11 | 10.1093/toxsci/kft197 |
| Citric acid | fumC | 10.1021/bi9614702 |
| Citric acid | IL4I1 | 10.1038/nrd2199\|10.1038/nrd2132\|10.1093/nar/28.1.235 |
| Citric acid | CTDSP1 | 10.1038/nrd2199\|10.1038/nrd2132\|10.1093/nar/28.1.235 |
| Citric acid | HGS | 10.1093/nar/28.1.235 |
| Citric acid | MDH2 | 10.1093/nar/28.1.235 |
| Citric acid | LSM6 | 10.1093/nar/28.1.235 |
| Citric acid | APRT | 10.1093/nar/28.1.235 |
| Citric acid | AKR1B1 | 10.1021/bi00174a006\|10.1107/s0907444907011997 |
| Citric acid | ITPA | 10.1093/nar/28.1.235 |
| Citric acid | CS | 10.1038/nrd2199\|10.1038/nrd2132\|10.1093/nar/28.1.235 |
| Citric acid | SRC | 10.1038/nrd2199\|10.1038/nrd2132\|10.1093/nar/28.1.235 |
| Citric acid | RNASE1 | 10.1038/nrd2199\|10.1038/nrd2132\|10.1093/nar/28.1.235 |
| Citric acid | blaP | 10.1016/j.bmcl.2008.05.045 |
| Citric acid | SLCO2B1 | 10.1124/jpet.103.051300 |

**Table S5 Approved Western drugs for unstable angina pectoris**

| Drug | Genename | Evidence |
| --- | --- | --- |
| Nicorandil | ABCC9 | 10.1038/sj.bjp.0705238 |
| Dopamine | DRD2 | 03.04/ijaai.169174\|10.1093/nar/30.1.412 |
| Dopamine | DRD1 | 03.04/ijaai.169174\|10.1002/ajmg.b.30544\|10.1007/bf03033483\|10.1007/s10899-007-9060-x\|10.1529/biophysj.106.088500 |
| Dopamine | DRD5 | 03.04/ijaai.169174 |
| Dopamine | DRD3 | 03.04/ijaai.169174 |
| Dopamine | DRD4 | 03.04/ijaai.169174 |
| Dopamine | SLC6A3 | 03.04/ijaai.169174 |
| Dopamine | DBH | 10.1016/j.ntt.2006.11.011\|10.1111/j.1460-9568.2007.05557.x\|10.1152/ajpheart.01389.2006\|10.1016/j.cbpc.2006.12.013\|10.1016/j.physbeh.2007.02.011 |
| Dopamine | HTR1A | 10.1016/s0742-8413(97)00614-2\| |
| Dopamine | HTR7 | 10.1016/0896-6273(93)90149-l\| |
| Dopamine | DRD1 | 10.1021/jm991098z |
| Dopamine | DRD5 | 10.1021/jm991098z |
| Dopamine | SLC6A2 | 10.1002/1098-2396(20010101)39:1<32::aid-syn5>3.0.co;2-3 |
| Dopamine | SLC6A4 | 10.1002/1098-2396(20010101)39:1<32::aid-syn5>3.0.co;2-3 |
| Dopamine | HTR3A | 10.1124/jpet.106.118752 |
| Dopamine | HTR3B | 10.1124/jpet.106.118752 |
| Dopamine | SOD1 | 10.1038/ncomms2750 |
| Dopamine | SLC18A2 | 10.1016/0169-328x(94)90050-7 |
| Dopamine | MAOA | 10.1016/j.addr.2008.06.002\|10.1016/j.bbamcr.2010.09.010\|10.1196/annals.1330.023 |
| Dopamine | MAOB | 10.1016/j.addr.2008.06.002\|10.1016/j.bbamcr.2010.09.010 |
| Dopamine | COMT | 10.1002/ajmg.b.31205\|10.1177/0269881111400644\|10.1196/annals.1330.023 |
| Dopamine | DBH | 10.1016/j.ntt.2006.11.011\|10.1111/j.1460-9568.2007.05557.x\|10.1152/ajpheart.01389.2006\|10.1016/j.cbpc.2006.12.013\|10.1016/j.physbeh.2007.02.011 |
| Dopamine | SLC22A2 | 10.1097/01.asn.0000019413.78751.46\|10.1074/jbc.273.49.32776\|10.1023/a:1013070128668\|10.1124/mol.54.2.342\|10.1074/jbc.273.47.30915\|10.1006/geno.1998.5639\|10.1124/mol.56.1.1 |
| Dopamine | SLC22A1 | 10.1124/mol.63.3.489\|\|10.1023/a:1013070128668\|10.1016/0014-5793(96)01030-7\|10.1038/sj.bjp.0702065\|10.1124/dmd.113.055095\|10.1124/mol.56.1.1 |
| Dopamine | SLC22A3 | 10.1074/jbc.273.49.32776 |
| Dopamine | SLC22A5 | 10.1124/mol.59.2.358\| |
| Dopamine | POU5F1 | 10.1111/j.1471-4159.2010.06738.x |
| Metoclopramide | DRD2 | 10.1016/0306-4522(95)00540-4\|10.1021/jm00073a017\|10.1254/jjp.67.45\|10.1006/phrs.1996.9999\|10.1046/j.1468-2982.1998.1809593.x\|10.1891/0730-0832.24.2.51\|10.2174/157488911795933901 |
| Metoclopramide | CHRM1 | 10.1586/eem.10.41 |
| Metoclopramide | HTR4 | 10.1677/erc-08-0190\|10.1586/eem.10.41\|10.2174/157488911795933901 |
| Metoclopramide | HTR3A | 10.1111/j.1476-5381.1987.tb10280.x\|10.1586/eem.10.41 |
| Metoclopramide | CYP2D6 | 10.3109/00498254.2013.835885\|10.1124/dmd.30.3.336\|10.1097/mcg.0b013e3182549528 |
| Metoclopramide | CYP3A4 | 10.1586/eem.10.41\|10.3109/00498254.2013.835885\|10.1503/cmaj.120951 |
| Metoclopramide | CYP1A2 | 10.1586/eem.10.41\|10.3109/00498254.2013.835885\|10.1097/mcg.0b013e3182549528 |
| Metoclopramide | ABCB1 | 10.2967/jnumed.115.164350\|10.1155/2018/7310146\|10.1002/cpt.1402 |
| Metoclopramide | ORM1 | 10.1111/j.1365-2125.1986.tb05201.x |
| Adenosine | ADORA2A | 10.2174/156802610791268756\|10.2174/156802610791268729\|10.1016/j.intimp.2006.12.001\|10.1124/jpet.105.096016\|10.1124/jpet.109.157651\|10.1111/j.1476-5381.2009.00614.x\|10.1161/circulationaha.107.694596\|10.4049/jimmunol.178.9.5921 |
| Adenosine | ADORA2B | 10.2174/156802610791268756\|10.2174/156802610791268729\|10.1016/j.intimp.2006.12.001\|10.1124/jpet.105.096016\|10.1111/j.1476-5381.2009.00614.x\|10.1161/circulationaha.107.694596\|10.4049/jimmunol.178.9.5921 |
| Adenosine | ADORA3 | 10.1093/nar/30.1.412\|10.2174/156802610791268756\|10.2174/156802610791268729\|10.1016/j.intimp.2006.12.001\|10.1124/jpet.105.096016\|10.1111/j.1476-5381.2009.00614.x\|10.1161/circulationaha.107.694596\|10.4049/jimmunol.178.9.5921 |
| Adenosine | ADORA1 | 10.2174/156802610791268756\|10.2174/156802610791268729\|10.1016/j.intimp.2006.12.001\|10.1111/j.1476-5381.2009.00614.x\|10.1161/circulationaha.107.694596\|10.4049/jimmunol.178.9.5921 |
| Adenosine | ADK | 10.1016/j.tips.2006.10.008 |
| Adenosine | SLC28A3 | 10.1038/sj.tpj.6500303 |
| Cangrelor | P2RY12 | 10.1185/03007995.2015.1098600 |
| Pioglitazone | PPARG | 10.1073/pnas.0912487106\|10.1210/jc.2004-0190\|10.2337/diabetes.47.4.507\|10.1021/jm950395a |
| Pioglitazone | MAOB | 10.1021/ml200196p |
| Pioglitazone | CYP2C8 | 10.1111/j.1742-7843.2009.00457.x\|10.1111/j.1742-7843.2006.pto_437.x\|10.1124/dmd.31.4.439\|10.2174/092986709789057635 |
| Pioglitazone | CYP3A4 | 10.1111/j.1742-7843.2006.pto_437.x\|10.1124/dmd.31.4.439\|10.1177/0091270002042012009\|10.2174/092986709789057635 |
| Pioglitazone | SLCO1B3 | 10.1124/dmd.32.3.291 |
| Pioglitazone | SLCO1B1 | 10.1124/dmd.32.3.291 |
| Pravastatin | HMGCR | 10.1093/nar/30.1.412\|10.4103/0366-6999.149226\| |
| Pravastatin | HDAC2 | 10.1158/0008-5472.can-07-5807 |
| Pravastatin | SLCO1B1 | 10.1124/dmd.104.002477\|10.1074/jbc.274.52.37161\|10.1124/jpet.105.085589\|10.1097/01.fpc.0000170913.73780.5f\|10.3109/00498250903351013 |
| Pravastatin | SLCO2B1 | 10.1097/fpc.0b013e32831bd98c\|10.1124/jpet.103.051300\|10.1124/jpet.103.060194 |
| Pravastatin | ABCB1 | 10.1124/dmd.104.002477\|10.1124/jpet.105.085589\|10.1155/2017/3418204 |
| Pravastatin | SLCO1A2 | 10.1074/jbc.274.52.37161 |
| Pravastatin | SLC22A6 | 10.1254/jphs.94.197 |
| Pravastatin | SLC22A8 | 10.1254/jphs.94.197\|10.1124/jpet.103.063370\|10.1124/jpet.300.3.746 |
| Pravastatin | ABCC2 | 10.1074/jbc.m109081200\|10.1124/jpet.105.085589 |
| Pravastatin | SLC22A11 | 10.1254/jphs.94.197 |
| Pravastatin | ABCG2 | 10.1074/jbc.m212399200\|10.1124/jpet.105.085589 |
| Pravastatin | SLC22A7 | 10.1254/jphs.94.197 |
| Pravastatin | SLC16A1 | 10.1023/a:1016269806840 |
| Pravastatin | ABCB11 | 10.1111/j.1872-034x.2009.00493.x |
| Pravastatin | SLCO1B3 | 10.1074/jbc.274.52.37161 |
| Simvastatin | HMGCR | 10.1194/jlr.m200002-jlr200\|10.1093/nar/30.1.412\|10.1124/dmd.30.12.1400\|10.1016/s0731-7085(03)00023-2\|10.1067/mlc.2003.31\|\|10.1021/bi050905v |
| Simvastatin | ITGAL | 10.1073/pnas.0305149101\|10.1038/89058\|10.1146/annurev.pharmtox.45.120403.095748\|10.1016/j.jneuroim.2006.05.029 |
| Simvastatin | HDAC2 | 10.1158/0008-5472.can-07-5807 |
| Simvastatin | CYP3A4 | 10.1016/j.clpt.2006.09.003\|10.1002/bdd.249\|10.2174/092986709789057635\|10.1124/dmd.30.12.1512\|10.1038/clpt.2012.163\|10.1177/0091270006295063\|10.1007/s11095-007-9519-6 |
| Simvastatin | CYP3A5 | 10.1177/0091270006295063\|10.1007/s11095-007-9519-6 |
| Simvastatin | CYP2C8 | 10.1016/j.clpt.2006.09.003\|10.1111/j.1742-7843.2005.pto_134.x\|10.2217/pgs.09.82\|10.1177/0091270004270642 |
| Simvastatin | CYP2C9 | 10.1002/bdd.249\|10.1007/s002280050094\|10.1007/s11095-007-9519-6 |
| Simvastatin | CYP2D6 | 10.2165/00129784-200404040-00005 |
| Simvastatin | CYP2B6 | 10.2174/092986709789057635\|10.1177/0091270006293753\|10.1124/dmd.30.12.1400 |
| Simvastatin | UGT1A1 | 10.2147/pgpm.s86013 |
| Simvastatin | UGT1A3 | 10.2147/pgpm.s86013 |
| Simvastatin | UGT2B7 | 10.2147/pgpm.s86013 |
| Simvastatin | CYP2C19 | 10.2147/pgpm.s86013 |
| Simvastatin | ABCB1 | 10.1023/a:1011036428972\|10.1006/bbrc.2001.6000\|10.1023/b:pham.0000041466.84653.8c\|10.1002/ijc.24885\|10.2147/pgpm.s86013 |
| Simvastatin | SLCO1A2 | 10.1074/jbc.274.52.37161 |
| Simvastatin | SLCO1B1 | 10.1074/jbc.274.52.37161\|10.1097/01.fpc.0000170913.73780.5f\|10.2147/pgpm.s86013\|10.1038/clpt.2012.163\|10.1097/01.fpc.0000230416.82349.90 |
| Simvastatin | ABCC2 | 10.1038/tpj.2011.59\|10.2147/pgpm.s86013 |
| Simvastatin | ABCB11 | 10.1093/toxsci/kft197 |
| Simvastatin | SLCO2B1 | 10.2147/pgpm.s86013 |
| Simvastatin | SLCO1B3 | 10.2147/pgpm.s86013 |
| Bupropion | SLC6A3 | 10.1124/jpet.102.033852\|10.1007/s00213-002-1166-3\|10.1038/sj.npp.1300036\|10.1016/s0006-3223(02)01834-6\|\|10.1093/nar/30.1.412\|10.1016/s0014-2999(97)01393-9\|10.4088/pcc.v06n0403\|10.1021/acschemneuro.7b00055\|10.1111/bcpt.12653\|10.1111/j.1471-4159.2006.04060.x |
| Bupropion | SLC6A2 | 10.1016/s0014-2999(03)02010-7\|10.1016/j.biopsych.2006.03.057\|10.1093/nar/30.1.412\|10.4088/pcc.v06n0403\|10.1021/acschemneuro.7b00055\|10.1111/j.1471-4159.2006.04060.x |
| Bupropion | HTR3A | 10.1016/j.neuropharm.2016.09.021 |
| Bupropion | CYP2B6 | 10.1177/0091270006293753\|10.1081/dmr-120001391\|\|10.1016/j.clinthera.2005.11.011\|10.1586/14737175.6.9.1249\|10.1081/dmr-120001392 |
| Bupropion | CYP2D6 | 10.1586/14737175.6.9.1249\|10.1097/01.jcp.0000162805.46453.e3\|10.1016/j.bcp.2016.11.007 |
| Bupropion | SLC22A2 | 10.1093/ntr/ntu161 |
| Bupropion | ORM1 | 10.1002/jps.2600750208 |
| Morphine | OPRM1 | 10.1016/j.brainres.2006.01.095\|10.1016/j.bbrc.2006.03.084\|10.1016/j.joca.2006.01.013\|10.1124/mol.106.022376\|10.1093/nar/30.1.412 |
| Morphine | OPRK1 | 10.1038/nrd2199\|10.1038/nrd2132\|\|10.1006/geno.1994.1331\|10.1016/j.neuroscience.2006.04.071 |
| Morphine | OPRD1 | 10.1016/j.brainres.2006.01.095\|10.1159/000091993\|10.1111/j.1471-4159.2006.03736.x\|10.1016/j.pain.2006.03.008\|10.1016/j.ejphar.2006.04.001\|10.1093/nar/30.1.412\|10.1016/s0301-0082(02)00008-4 |
| Morphine | LY96 | 10.1016/j.neuroscience.2010.02.011 |
| Morphine | CYP2C8 | 10.1080/0049825031000121608\|10.2217/pgs.09.82 |
| Morphine | CYP3A4 | 10.1080/0049825031000121608\|10.1248/bpb.28.2026\|10.2217/pgs.09.82 |
| Morphine | UGT2B7 | 10.1124/mol.104.007641\|10.2131/jts.28.395\|10.1124/dmd.109.030635 |
| Morphine | UGT1A1 | 10.1124/dmd.107.019281 |
| Morphine | UGT1A8 | 10.1124/dmd.107.019281 |
| Morphine | UGT2B15 | 10.1124/dmd.109.030635 |
| Morphine | UGT2B4 | 10.1124/dmd.109.030635 |
| Morphine | ABCB1 | 10.1021/jm021012t\|10.1023/a:1025001131513 |
| Morphine | ALB | 10.1213/ane.0b013e318232e922 |
| Metoprolol | ADRB1 | 10.1152/ajpcell.2000.279.2.c495\|10.1016/s0014-2999(01)01113-x\|10.1016/s0014-2999(03)01431-6\|10.1016/s0009-9236(03)00068-7\|10.1016/s0009-9236(03)00224-8\|10.1093/nar/30.1.412 |
| Metoprolol | ADRB2 | 10.1592/phco.29.8.883 |
| Metoprolol | CYP2D6 | 10.1007/s002280050456\|\|10.1111/bcp.13741 |
| Metoprolol | SLC22A2 | 10.1038/sj.bjp.0703518 |
| Atorvastatin | HMGCR | 10.1177/107424840300800205\|10.1016/j.tem.2004.03.008\|10.1016/j.bbrc.2004.09.122\|10.1111/j.1471-4159.2004.02980.x\|10.1093/nar/30.1.412 |
| Atorvastatin | DPP4 | 10.1016/j.bmcl.2007.11.107 |
| Atorvastatin | AHR | 10.1124/mol.106.032748\|10.1007/s40262-013-0075-4\|10.1371/journal.pone.0137720 |
| Atorvastatin | HDAC2 | 10.1158/0008-5472.can-07-5807 |
| Atorvastatin | NR1I3 | 10.1124/dmd.117.075523 |
| Atorvastatin | CYP3A4 | 10.1016/j.clpt.2006.09.003\|10.1002/bdd.249\|10.2174/092986709789057635\| |
| Atorvastatin | CYP2C8 | 10.1124/pr.115.011411\|10.1111/j.1742-7843.2005.pto_134.x |
| Atorvastatin | CYP2D6 | 10.1002/bdd.249 |
| Atorvastatin | CYP2C9 | 10.1002/bdd.249 |
| Atorvastatin | CYP2C19 | 10.1002/bdd.249 |
| Atorvastatin | CYP2B6 | 10.2174/092986709789057635\|10.1124/dmd.104.002741\|10.1124/dmd.30.12.1400 |
| Atorvastatin | UGT1A1 | 10.1007/s40291-013-0031-x\|10.2165/00003088-200342130-00005 |
| Atorvastatin | UGT1A3 | 10.1007/s40291-013-0031-x\|10.2165/00003088-200342130-00005 |
| Atorvastatin | ABCB1 | 10.1023/a:1011036428972\|10.1002/ijc.24885\|10.2165/00003088-200342130-00005 |
| Atorvastatin | SLCO1A2 | 10.1074/jbc.274.52.37161\|10.1016/j.bcp.2010.08.008 |
| Atorvastatin | SLCO1B1 | 10.1074/jbc.274.52.37161\|10.1097/01.fpc.0000170913.73780.5f\|10.2165/00003088-200342130-00005 |
| Atorvastatin | ABCC4 | 10.1161/circresaha.109.203596 |
| Atorvastatin | ABCC5 | 10.1161/circresaha.109.203596 |
| Atorvastatin | ABCC1 | 10.1161/circresaha.109.203596 |
| Atorvastatin | SLCO2B1 | 10.1016/j.clpt.2006.09.010\|10.1161/circresaha.109.203596 |
| Atorvastatin | SLCO1B3 | 10.1111/bcpt.12031\|10.2165/00003088-200342130-00005 |
| Atorvastatin | ABCC2 | 10.1038/tpj.2011.59 |
| Atorvastatin | ABCB11 | 10.1093/toxsci/kft197 |
| Nitric Oxide | GUCY1A2 | 10.1111/j.1471-4159.2010.06606.x |
| Nitric Oxide | MT1A | 10.1042/0264-6021:3440253 |
| Nitric Oxide | IDO1 | 10.1021/bi060143j |
| Nitric Oxide | ALDH2 | 10.1016/j.febslet.2005.09.082 |
| Nitric Oxide | CYP1A2 | 10.1074/jbc.271.15.8570\|10.1046/j.1460-9568.2003.02972.x\| |
| Nitric Oxide | CYP2B6 | 10.1016/j.freeradbiomed.2007.12.010\|10.1016/j.freeradbiomed.2017.04.015 |
| Nitric Oxide | CYP3A4 | 10.1016/j.lfs.2003.07.006 |
| Nitroglycerin | NPR1 | 10.1038/sj.bjp.0705365 |
| Nitroglycerin | ALDH2 | 10.1152/ajpheart.00959.2009\|10.1073/pnas.122225199 |
| Ticagrelor | P2RY12 | 10.1007/s40262-015-0290-2 |
| Ticagrelor | CYP3A4 | 10.1124/dmd.110.032250 |
| Ticagrelor | CYP2C9 | 10.1124/dmd.110.037143\|10.1007/s40262-015-0290-2 |
| Ticagrelor | ALB | 10.1016/j.jacbts.2017.01.007 |
| Progesterone | PGR | 10.1002/med.20083\|10.1128/mcb.00326-06\|10.1210/me.2006-0337\|10.1016/s1472-6483(10)60655-4\|10.1210/jcem.83.3.4672\|10.1002/rmb2.12088 |
| Progesterone | ESR1 | 10.1186/1477-7827-4-s1-s9\|10.1186/bcr1660\|10.1038/nature14583\|10.1111/j.1365-2826.2008.01801.x\|10.1210/endo.141.10.7734 |
| Progesterone | NR3C2 | 10.1016/0922-4106(93)90072-h\|10.1530/eje.0.1460789\|10.1152/ajpendo.1996.270.4.e601\|10.1210/endo.136.12.7588320 |
| Progesterone | CYP17A1 | 10.1002/ardp.200290006\|10.1016/j.jsbmb.2010.11.005\|10.1016/s0003-9861(02)00491-5 |
| Progesterone | OPRK1 | 10.1016/0304-3959(95)00092-5\|10.1006/hbeh.1996.0029 |
| Progesterone | ORM1 | 10.1016/j.carres.2006.07.012\|10.1016/s0008-6215(02)00165-9\|10.1016/s0304-4165(97)00043-3\|10.1016/s1570-9639(02)00465-x |
| Progesterone | NR3C1 | 10.1016/j.ajog.2007.05.024\|10.1016/0022-4731(90)90089-b\|10.1210/en.2003-0732 |
| Progesterone | AR | 10.1016/0016-5085(92)91496-q\|10.1016/s0303-7207(99)00109-4\|10.1016/j.urolonc.2008.03.021 |
| Progesterone | SHBG | 10.1093/toxsci/kfu231\|10.1016/0022-4731(84)90249-8\|10.1016/s0960-0760(97)00045-9 |
| Progesterone | ESR2 | 10.1111/j.1471-4159.2010.07038.x\|10.1111/j.1365-2826.2008.01801.x\|10.1210/endo.141.10.7734\|10.1186/bcr1660 |
| Progesterone | CYP3A5 | 10.1080/15513815.2017.1354411\|10.1016/j.ajog.2017.05.019 |
| Progesterone | CYP2C9 | 10.1006/abbi.1997.0302\|10.3389/fphar.2016.00098 |
| Progesterone | CYP3A7 | 10.1080/15513815.2017.1354411\|10.1124/dmd.109.029918 |
| Progesterone | CYP3A7-CYP3A51P | 10.1080/15513815.2017.1354411\|10.1124/dmd.109.029918 |
| Progesterone | CYP2C19 | 10.1006/abbi.1997.0302\|10.1210/jc.2008-1174\|10.1093/jb/mvj093\|10.1006/abbi.1995.0013 |
| Progesterone | CYP3A4 | 10.1016/0006-2952(96)00357-7\|10.1039/c2dt31833d\|10.1021/acs.bioconjchem.6b00604\|10.1126/science.1099736\|\|10.1124/dmd.112.046276 |
| Progesterone | CYP17A1 | 10.1074/jbc.m114.610998 |
| Progesterone | CYP1A1 | 10.1097/00008571-200008000-00005\|10.1080/004982598239290 |
| Progesterone | CYP1B1 | 10.1093/carcin/20.8.1607\|10.1016/j.pharmthera.2017.03.007\|10.1097/00008571-200112000-00007 |
| Progesterone | CYP2A6 | 10.1124/dmd.112.046276 |
| Progesterone | CYP2D6 | 10.1016/j.molbrainres.2004.06.030\|10.1248/bpb.31.348\|10.1111/bph.12652 |
| Progesterone | ABCB1 | 10.1006/bbrc.2001.6000\|10.1021/jm010126m\|\|10.1016/j.bbrc.2004.01.156\|\|10.1023/a:1013358126640\|10.1074/jbc.271.6.3163\|10.1023/b:pham.0000033017.52484.81 |
| Progesterone | SLC22A2 | 10.1038/sj.bjp.0704785\|10.1074/jbc.273.49.32776 |
| Progesterone | SLC22A1 | 10.1038/sj.bjp.0704785\|10.1074/jbc.273.49.32776\|10.1155/2013/692071 |
| Progesterone | SLC22A3 | 10.1038/sj.bjp.0704785\|10.1074/jbc.273.49.32776 |
| Progesterone | ABCB11 | 10.1023/a:1023278211849\|10.1159/000371565 |
| Progesterone | ABCC1 | 10.1006/bbrc.1999.0671 |
| Progesterone | SLC10A1 | 10.1152/ajpgi.1998.274.2.g370\|10.1159/000371565\|10.1074/jbc.m109.072140\| |
| Progesterone | SLCO1B1 | 10.2174/1875397301004010001 |
| Progesterone | ABCG2 | 10.1208/s12248-014-9668-6\|10.1007/s11095-008-9537-z\|10.1159/000354442 |
| Progesterone | SLCO1B3 | 10.1038/s41598-018-20815-1\|10.2174/1875397301004010001 |
| Sertraline | SLC6A4 | 20026626\|\|10.1007/s00213-003-1562-3\|10.1111/j.1471-4159.2004.02835.x\|10.1016/s0014-2999(97)01393-9\|10.1007/s002130000588\|\|10.1007/s11064-008-9818-2 |
| Sertraline | SLC6A3 | 10.1016/s0014-2999(97)01393-9\|\|10.1007/s00115-006-2104-0\|10.1017/s1092852900025475\|10.1177/0269881198012003021 |
| Sertraline | PGRMC1 | 10.1016/0014-2999(96)00254-3\|10.1016/j.pharmthera.2010.04.003 |
| Sertraline | SIGMAR1 | 10.1016/0014-2999(96)00254-3\|10.1016/j.pharmthera.2010.04.003 |
| Sertraline | SLC6A2 | 10.1016/s0014-2999(97)01393-9\|10.1074/jbc.m112.342212 |
| Sertraline | SLC29A4 | 10.1016/j.pharmthera.2008.10.004\|10.1016/j.bcp.2006.09.008\|10.1007/s00210-009-0479-8 |
| Sertraline | CYP3A4 | 10.1016/s0013-7006(06)76153-x\|\|10.1097/01.jcp.0000104908.75206.26\|10.1124/dmd.104.002428\|10.2147/tcrm.s49063 |
| Sertraline | CYP2D6 | 10.2165/00003088-199631060-00004\|10.1016/s0013-7006(06)76153-x\|\|10.1124/dmd.104.002428 |
| Sertraline | CYP2C19 | 10.1016/s0013-7006(06)76153-x\|\|10.1124/dmd.104.002428\|10.1111/j.1600-0773.1996.tb00206.x\|10.1007/s11096-016-0259-8 |
| Sertraline | CYP2C9 | 10.1124/dmd.104.002428\|10.1046/j.1365-2125.1997.00601.x\|10.2165/00003088-199700321-00005\|10.2165/00003088-199700321-00006 |
| Sertraline | CYP2B6 | 10.1124/dmd.104.002428\|10.1177/0091270006293753\|10.1081/dmr-120001392\|10.1007/s11096-016-0259-8\|10.1007/s13318-011-0065-6 |
| Sertraline | MAOB | 10.1124/dmd.104.002428 |
| Sertraline | MAOA | 10.1124/dmd.104.002428 |
| Sertraline | ABCB1 | 10.1124/jpet.102.046532\|10.1371/journal.pone.0056525 |
| Sertraline | ALB | 10.1016/j.cbi.2015.10.006 |
| Aspirin | PTGS1 | 10.1016/s0065-2423(06)42003-5\|10.1016/j.prostaglandins.2006.10.003\|10.1111/j.1538-7836.2007.02387.x |
| Aspirin | PTGS2 | 10.1158/1055-9965.epi-06-0346\|10.1016/s0049-3848(03)00379-7 |
| Aspirin | AKR1C1 | 10.2174/157340607782360399 |
| Aspirin | PRKAA1 | 10.1053/j.gastro.2012.02.050\|10.1126/science.1215327\|10.1016/j.tem.2013.06.002 |
| Aspirin | PRKAA2 | 10.1053/j.gastro.2012.02.050\|10.1126/science.1215327\|10.1016/j.tem.2013.06.002 |
| Aspirin | PRKAB1 | 10.1053/j.gastro.2012.02.050\|10.1126/science.1215327\|10.1016/j.tem.2013.06.002 |
| Aspirin | PRKAB2 | 10.1053/j.gastro.2012.02.050\|10.1126/science.1215327\|10.1016/j.tem.2013.06.002 |
| Aspirin | PRKAG1 | 10.1053/j.gastro.2012.02.050\|10.1126/science.1215327\|10.1016/j.tem.2013.06.002 |
| Aspirin | PRKAG2 | 10.1053/j.gastro.2012.02.050\|10.1126/science.1215327\|10.1016/j.tem.2013.06.002 |
| Aspirin | PRKAG3 | 10.1053/j.gastro.2012.02.050\|10.1126/science.1215327\|10.1016/j.tem.2013.06.002 |
| Aspirin | EDNRA | 10.1124/mol.57.4.797\|10.1124/mol.58.6.1461\|10.1124/pr.115.011833 |
| Aspirin | TP53 | 10.3892/mmr_00000132\|10.1007/s13277-015-4438-3\|10.1016/j.ejphar.2014.03.009\|10.1016/s0006-291x(02)02987-x\|10.1093/carcin/bgm101 |
| Aspirin | HSPA5 | 10.1096/fj.01-0259com |
| Aspirin | RPS6KA3 | 10.1517/14728222.2011.537656\|10.1038/23810 |
| Aspirin | TNFAIP6 | 10.1124/mol.52.3.421\|\|10.1016/j.freeradbiomed.2017.01.010\|10.1016/j.cyto.2003.11.007 |
| Aspirin | CASP1 | 10.1038/bjc.2014.271\|10.1371/journal.pone.0048208\|10.3389/fonc.2018.00679\|10.1093/emph/eow009 |
| Aspirin | CASP3 | 10.1038/bjc.2014.271\|10.1371/journal.pone.0048208 |
| Aspirin | IKBKB | 10.1038/bjc.2014.271\|10.1128/mcb.20.10.3655-3666.2000\|10.1016/j.brainres.2017.10.020\|10.1111/j.1476-5381.2011.01608.x\|10.1172/jci11914\|10.1172/jci18712 |
| Aspirin | MAPK1 | 10.1038/bjc.2014.271\|10.3892/mmr.2013.1676\|10.1007/s11010-015-2613-x\|10.1007/978-1-4615-0193-0_87 |
| Aspirin | MAPK15 | 10.1038/bjc.2014.271\|10.3892/mmr.2013.1676\|10.1007/s11010-015-2613-x\|10.1007/978-1-4615-0193-0_87 |
| Aspirin | MAPK3 | 10.1038/bjc.2014.271\|10.3892/mmr.2013.1676\|10.1007/s11010-015-2613-x\|10.1007/978-1-4615-0193-0_87 |
| Aspirin | MAPK4 | 10.1038/bjc.2014.271\|10.3892/mmr.2013.1676\|10.1007/s11010-015-2613-x\|10.1007/978-1-4615-0193-0_87 |
| Aspirin | MAPK6 | 10.1038/bjc.2014.271\|10.3892/mmr.2013.1676\|10.1007/s11010-015-2613-x\|10.1007/978-1-4615-0193-0_87 |
| Aspirin | MAPK7 | 10.1038/bjc.2014.271\|10.3892/mmr.2013.1676\|10.1007/s11010-015-2613-x\|10.1007/978-1-4615-0193-0_87 |
| Aspirin | CCND1 | 10.1074/jbc.m005545200\|10.18632/oncotarget.16325\| |
| Aspirin | MYC | 10.1074/jbc.m005545200\|10.18632/oncotarget.16325\|10.1007/s13277-015-3959-0 |
| Aspirin | PCNA | 10.1074/jbc.m005545200\|10.1016/j.cdp.2004.01.001\|10.1053/j.gastro.2012.02.050 |
| Aspirin | CYP2C19 | 10.1067/mcp.2003.14 |
| Aspirin | CYP2C9 | 10.4168/aair.2011.3.4.273 |
| Aspirin | UGT1A6 | 10.4168/aair.2011.3.4.273\|10.1093/jnci/dji066\|10.1097/01.fpc.0000236339.79916.07 |
| Aspirin | NAT2 | 10.4168/aair.2011.3.4.273\|10.1097/fpc.0000000000000062 |
| Aspirin | SLC22A6 | 10.1128/aac.02392-16 |
| Aspirin | ABCB1 | 10.1016/s0378-5173(03)00372-7\|10.1002/cpt.32\|10.1038/clpt.2014.49\|10.1159/000354497\| |
| Aspirin | SLC22A8 | 10.1128/aac.02392-16\|10.1124/dmd.113.055194 |
| Lidocaine | SCN10A | 10.1073/pnas.0601819103\|10.1085/jgp.200810103\|10.1371/journal.pcbi.1000818 |
| Lidocaine | SCN9A | 10.1113/jphysiol.2006.127027\|10.1085/jgp.200810103\|10.1371/journal.pcbi.1000818 |
| Lidocaine | SCN5A | 10.1016/j.ijcard.2007.02.007\|10.1111/j.1540-8167.2006.00386.x\|10.1038/sj.bjp.0706897\|10.1093/humrep/del271\|10.1085/jgp.200810103\|10.1371/journal.pcbi.1000818 |
| Lidocaine | EGFR | 10.1213/01.ane.0000198330.84341.35 |
| Lidocaine | SCN4A | 10.1038/sj.bjp.0705594 |
| Lidocaine | ORM1 | 10.1097/00008571-199610000-00004 |
| Lidocaine | ORM2 | 10.1097/00008571-199610000-00004 |
| Lidocaine | CYP3A4 | 10.1159/000443332\|10.1038/clpt.1994.94 |
| Lidocaine | CYP1A2 | 10.2174/092986709789378198\|\|\|10.1016/j.clpt.2003.09.007 |
| Lidocaine | CYP2C9 | 10.1081/dmr-120001392\| |
| Lidocaine | CYP2C8 | 10.1081/dmr-120001392 |
| Lidocaine | CYP2A6 | 10.1081/dmr-120001392 |
| Lidocaine | CYP2B6 | 10.1016/j.apsb.2016.07.016 |
| Lidocaine | SLC22A5 | 10.1124/mol.59.2.358 |
| Lidocaine | ABCB1 | 10.1021/tx010125x\|10.1016/j.bbrc.2004.01.156\|10.1097/cad.0000000000000455 |
| Dobutamine | ADRB1 | 10.1016/s0014-2999(02)01814-9\|10.1097/00008571-200411000-00001\|10.1016/j.jacc.2005.08.041\|10.1164/rccm.200508-1221oc\|10.1093/nar/30.1.412 |
| Dobutamine | ADRB2 | 10.1164/ajrccm.156.2.9609141 |
| Dobutamine | ESR1 | 10.1093/toxsci/kfu114 |
| Dobutamine | COMT | 10.3109/00498258609043504\|10.1124/jpet.301.1.315 |
| Ethanol | GABRA1 | 10.1038/nrd2199\|10.1038/nrd2132\|10.1016/j.alcohol.2007.04.011\|10.1093/nar/30.1.412\|10.1124/jpet.110.178244\| |
| Ethanol | GRIN3A | 10.1038/nrd2199\|10.1038/nrd2132\|10.4161/chan.5.3.14856\|10.1016/j.alcohol.2010.08.015 |
| Ethanol | GLRA1 | 10.1038/nrd2199\|10.1038/nrd2132\|10.1016/j.neuropharm.2010.10.023\|10.1016/j.neuropharm.2010.08.007\|10.1074/jbc.273.6.3314 |
| Ethanol | GLRA2 | 10.1038/nrd2199\|10.1038/nrd2132\|10.1074/jbc.273.6.3314\|10.1016/j.neuropharm.2010.08.007 |
| Ethanol | CACNB1 | 10.1124/jpet.103.055137 |
| Ethanol | HTR3A | 10.1124/pr.108.000430 |
| Ethanol | GABRA2 | 10.1016/j.alcohol.2007.04.011\| |
| Ethanol | CHRNA10 | 10.3389/fpsyt.2013.00029\|10.1124/pr.108.000430 |
| Ethanol | CACNA1C | 10.1124/jpet.103.055137 |
| Ethanol | GABRA5 | 10.1016/j.alcohol.2007.04.011\| |
| Ethanol | GABRA4 | 10.1016/j.alcohol.2007.04.011\| |
| Ethanol | GABRA3 | 10.1016/j.alcohol.2007.04.011\| |
| Ethanol | GRIA1 | 10.1124/pr.108.000430 |
| Ethanol | CACNG1 | 10.1124/jpet.103.055137 |
| Ethanol | CHRNA2 | 10.3389/fpsyt.2013.00029\|10.1124/pr.108.000430 |
| Ethanol | GABRA6 | 10.1016/j.alcohol.2007.04.011\| |
| Ethanol | GRIA2 | 10.1124/pr.108.000430 |
| Ethanol | GRIA4 | 10.1124/pr.108.000430 |
| Ethanol | GRIA3 | 10.1124/pr.108.000430 |
| Ethanol | CHRNA4 | 10.3389/fpsyt.2013.00029\|10.1124/pr.108.000430 |
| Ethanol | CHRNB2 | 10.3389/fpsyt.2013.00029\|10.1124/pr.108.000430 |
| Ethanol | KCNJ3 | 10.1152/physrev.00013.2008\|10.1073/pnas.1311406110 |
| Ethanol | KCNJ6 | 10.1152/physrev.00013.2008\|10.1073/pnas.1311406110 |
| Ethanol | VCAM1 | 10.1073/pnas.0707815105 |
| Ethanol | SLC29A1 | 10.1111/j.1530-0277.2009.00897.x |
| Ethanol | KCNJ5 | 10.1152/physrev.00013.2008\|10.1073/pnas.1311406110 |
| Ethanol | CHRFAM7A | 10.3389/fpsyt.2013.00029\|10.1124/pr.108.000430 |
| Ethanol | CHRNA7 | 10.3389/fpsyt.2013.00029\|10.1124/pr.108.000430 |
| Ethanol | CHRNA9 | 10.3389/fpsyt.2013.00029\|10.1124/pr.108.000430 |
| Ethanol | GABRB1 | 10.1016/j.alcohol.2007.04.011\| |
| Ethanol | GABRB3 | 10.1016/j.alcohol.2007.04.011\| |
| Ethanol | GABRB2 | 10.1016/j.alcohol.2007.04.011\| |
| Ethanol | CACNA1S | 10.1124/jpet.103.055137 |
| Ethanol | CACNA1D | 10.1124/jpet.103.055137 |
| Ethanol | CHRNB4 | 10.3389/fpsyt.2013.00029\|10.1124/pr.108.000430 |
| Ethanol | CHRNA3 | 10.3389/fpsyt.2013.00029\|10.1124/pr.108.000430 |
| Ethanol | CHRNA5 | 10.3389/fpsyt.2013.00029\|10.1124/pr.108.000430 |
| Ethanol | CHRNA6 | 10.3389/fpsyt.2013.00029\|10.1124/pr.108.000430 |
| Ethanol | CHRNB3 | 10.3389/fpsyt.2013.00029\|10.1124/pr.108.000430 |
| Ethanol | GABRG1 | 10.1016/j.alcohol.2007.04.011\| |
| Ethanol | GABRG3 | 10.1016/j.alcohol.2007.04.011\| |
| Ethanol | GABRE | 10.1016/j.alcohol.2007.04.011\| |
| Ethanol | GABRP | 10.1016/j.alcohol.2007.04.011\| |
| Ethanol | GABRQ | 10.1016/j.alcohol.2007.04.011\| |
| Ethanol | GABRD | 10.1016/j.alcohol.2007.04.011\| |
| Ethanol | SLC29A2 | 10.1111/j.1530-0277.2009.00897.x |
| Ethanol | HTR3E | 10.1124/pr.108.000430 |
| Ethanol | HTR3B | 10.1124/pr.108.000430 |
| Ethanol | HTR3D | 10.1124/pr.108.000430 |
| Ethanol | HTR3C | 10.1124/pr.108.000430 |
| Ethanol | CACNG2 | 10.1124/jpet.103.055137 |
| Ethanol | KCNJ9 | 10.1152/physrev.00013.2008\|10.1073/pnas.1311406110 |
| Ethanol | L1CAM | 10.1073/pnas.0707815105 |
| Ethanol | CYP1A2 | 10.1081/dmr-120001392 |
| Ethanol | CYP2B6 | 10.1081/dmr-120001392\| |
| Ethanol | CYP2C9 | 10.1248/bpb.32.517\|10.1002/prp2.324 |
| Ethanol | CYP2C19 | 10.1081/dmr-120001392\| |
| Ethanol | CYP2E1 | 10.1081/dmr-120001391\|10.1081/dmr-120001392\|10.1159/000070014\|10.1111/j.1530-0277.1996.tb01722.x\|10.1006/bbrc.1994.2774\|10.1067/mcp.2000.106574 |
| Ethanol | CYP3A4 | 10.1081/dmr-120001392\|\|10.1097/01.alc.0000071738.53337.f4\|10.1006/abbi.1995.1495\|\|10.1211/0022357043950\| |
| Ethanol | ADH1A | 10.1016/j.cbi.2011.02.008\|10.1111/j.1530-0277.2010.01319.x\|\|10.1007/s10620-008-0422-8 |
| Ethanol | ADH1B | 10.1016/j.cbi.2011.02.008\|10.1111/j.1530-0277.2010.01319.x\|\|10.1007/s10620-008-0422-8 |
| Ethanol | ADH1C | 10.1016/j.cbi.2011.02.008\|10.1111/j.1530-0277.2010.01319.x\|\|10.1007/s10620-008-0422-8 |
| Ethanol | ADH5 | 10.1111/j.1530-0277.2006.00139.x |
| Ethanol | ADH4 | 10.1111/j.1530-0277.2006.00139.x |
| Ethanol | ADH7 | 10.1111/j.1530-0277.2006.00139.x |
| Ethanol | ADH6 | 10.1111/j.1530-0277.2006.00139.x |
| Ethanol | AKR1A1 | 10.1111/j.1530-0277.2006.00139.x |
| Vitamin C | PLOD2 | 10.1006/abbi.1997.0319 |
| Vitamin C | PHYH | 10.1093/hmg/10.18.1971 |
| Vitamin C | PLOD3 | 10.1006/abbi.1997.0319 |
| Vitamin C | BBOX1 | 10.1093/ajcn/54.6.1147s |
| Vitamin C | DBH | 10.3177/jnsv.43.491\|\|\| |
| Vitamin C | PAM | 10.1016/j.jplph.2006.12.011\|10.1007/s00425-006-0321-1\|10.1021/ja062876x\|10.1016/s0076-6879(97)79007-4 |
| Vitamin C | P3H1 | 10.1016/j.biochi.2017.12.011 |
| Vitamin C | P3H2 | 10.1016/j.biochi.2017.12.011 |
| Vitamin C | P3H3 | 10.1016/j.biochi.2017.12.011 |
| Vitamin C | P4HA1 | 10.1016/j.biochi.2017.12.011 |
| Vitamin C | OGFOD1 | 10.3389/fonc.2014.00359 |
| Vitamin C | OGFOD2 | 10.3389/fonc.2014.00359 |
| Vitamin C | ALKBH2 | 10.1073/pnas.262589799 |
| Vitamin C | ALKBH3 | 10.1073/pnas.262589799 |
| Vitamin C | KDM5D | 10.1016/j.cell.2007.02.004 |
| Vitamin C | PLOD1 | 10.1093/nar/30.1.412\|10.1006/abbi.1997.0319 |
| Vitamin C | TMLHE | 10.1111/j.1742-4658.2007.06108.x |
| Vitamin C | P4HTM | 10.1021/acs.biochem.6b00251 |
| Vitamin C | EGLN1 | 10.1016/j.freeradbiomed.2018.03.033 |
| Vitamin C | EGLN2 | 10.1089/ars.2007.1683\|10.2174/092986707781058850\|10.1016/j.freeradbiomed.2018.03.033 |
| Vitamin C | EGLN3 | 10.1016/j.freeradbiomed.2018.03.033 |
| Vitamin C | TXNRD1 | 10.3389/fphys.2015.00397 |
| Vitamin C | SLC23A1 | 10.1038/19986\|10.1146/annurev.nutr.25.050304.092647\|10.1038/sj.jid.5700572\|10.1016/j.cbpa.2006.11.025\|10.1007/s00726-007-0555-7\|10.1111/j.1600-079x.2007.00453.x\|10.1016/j.freeradbiomed.2007.05.001 |
| Vitamin C | SLC23A2 | 10.1146/annurev.nutr.25.050304.092647 |
| Vitamin C | SLC2A1 | 10.1146/annurev.nutr.25.050304.092647 |
| Vitamin C | SLC2A3 | 10.1146/annurev.nutr.25.050304.092647 |
| Vitamin C | SLC2A4 | 10.1146/annurev.nutr.25.050304.092647 |
| Vitamin C | ALB | 10.1016/s0304-4165(02)00257-x\| |

**Part 4 contains Table S6 and Table S7.**

**Table S6 Literature validation of therapeutic effects**

| Mol_ID | Name | Disease | PMID |
| --- | --- | --- | --- |
| MOL000006 | luteolin | Atherosclerosis | 29056912\|23686014 |
| MOL000006 | luteolin | Cardiovascular Diseases | 33720053\|29056912 |
| MOL000098 | quercetin | Cardiovascular Diseases | 33860002\|34225730 |
| MOL000098 | quercetin | Atherosclerosis | 33860002\|32565865 |
| MOL000098 | quercetin | Hypertension | 33860002\|33398980 |
| MOL000173 | wogonin | Atherosclerosis | 31265843\|21986573 |
| MOL000173 | wogonin | Cardiovascular Diseases | 31298148\|27274287 |
| MOL000239 | Jaranol | Blood Platelet Disorders | 17516329 |
| MOL000354 | isorhamnetin | Cardiovascular Diseases | 33922903\|33117805 |
| MOL000354 | isorhamnetin | Atherosclerosis | 33732368\|32046625 |
| MOL000392 | formononetin | Cardiovascular Diseases | 29722068\|31276751 |
| MOL000392 | formononetin | Dyslipidemias | 32993069\|31938053 |
| MOL000392 | formononetin | Atherosclerosis | 31938053\|27588108 |
| MOL000417 | Calycosin | Cardiovascular Diseases | 33357721\|33647882 |
| MOL000417 | Calycosin | Myocardial Ischemia | 33357721\|25975048 |
| MOL000422 | kaempferol | Cardiovascular Diseases | 31557798\|33761622 |
| MOL000422 | kaempferol | Atherosclerosis | 33761622\|25982933 |
| MOL000490 | petunidin | Cardiovascular Diseases | 32476138 |
| MOL000490 | petunidin | Coronary Disease | 18789665 |
| MOL000785 | palmatine | Hyperlipidemias | 32926925 |
| MOL000785 | palmatine | Hypertension | 31496018 |
| MOL000785 | palmatine | Cardiovascular Diseases | 33235437 |
| MOL001002 | ellagic acid | Thrombosis | 24418625 |
| MOL001002 | ellagic acid | Cardiovascular Diseases | 32380485 |
| MOL001002 | ellagic acid | Atherosclerosis | 27633111 |
| MOL001454 | berberine | Cardiovascular Diseases | 33775071\|33815112 |
| MOL001454 | berberine | Hyperlipidemias | 33186794\|32105754 |
| MOL001454 | berberine | Dyslipidemias | 33815112\|28656091 |
| MOL001454 | berberine | Atherosclerosis | 33775071\|34305616 |
| MOL001454 | berberine | Hypercholesterolemia | 33775071\|32540763 |
| MOL001458 | coptisine | Hyperlipidemias | 31028858 |
| MOL001458 | coptisine | Hypertension | 28867709 |
| MOL001458 | coptisine | Cardiovascular Diseases | 32636749 |
| MOL001458 | coptisine | Hypercholesterolemia | 27957743\|25547428 |
| MOL001458 | coptisine | Myocardial Ischemia | 32933715\|24267256 |
| MOL001689 | acacetin | Atrial Fibrillation | 29081746\|21906601 |
| MOL001689 | acacetin | Cardiovascular Diseases | 34175838\|33241629 |
| MOL001689 | acacetin | Heart Diseases | 32918384\|27819271 |
| MOL002135 | Myricanone | Essential Hypertension | 32187175 |
| MOL002135 | Myricanone | Cardiovascular Diseases | 32187175 |
| MOL002341 | Hesperetin | Cardiovascular Diseases | 34212313\|33491467 |
| MOL002341 | Hesperetin | Atherosclerosis | 34212313\|22429094 |
| MOL002695 | lignan | Cardiovascular Diseases | 33761598 |
| MOL002695 | lignan | Atherosclerosis | 26215288 |
| MOL002695 | lignan | Hypertension | 23895153 |
| MOL002712 | 6-Hydroxykaempferol | Blood Platelet Disorders | 27286914 |
| MOL002712 | 6-Hydroxykaempferol | Myocardial Ischemia | 27286914 |
| MOL002712 | 6-Hydroxykaempferol | Thrombosis | 27286914 |
| MOL002712 | 6-Hydroxykaempferol | Heart Diseases | 12016905 |
| MOL002897 | epiberberine | Hyperlipidemias | 33571918 |
| MOL002897 | epiberberine | Hypertension | 30155694 |
| MOL002897 | epiberberine | Dyslipidemias | 33152460 |
| MOL002897 | epiberberine | Cardiovascular Diseases | 29343943 |
| MOL002897 | epiberberine | Hypercholesterolemia | 26593426 |
| MOL002897 | epiberberine | Atherosclerosis | 31291876 |
| MOL000490 | petunidin | Cardiovascular Diseases | 32476138 |
| MOL000490 | petunidin | Coronary Disease | 18789665 |
| MOL004841 | Licochalcone B | Heart Injuries | 25215172 |
| MOL004883 | Licoisoflavone | Cardiomegaly | 32018211 |
| MOL004883 | Licoisoflavone | Heart Failure | 32018211 |
| MOL004883 | Licoisoflavone | Cardiovascular Diseases | 32018211 |
| MOL004949 | Isolicoflavonol | Hyperlipidemias | 28920350 |
| MOL004904 | licopyranocoumarin | Blood Platelet Disorders | 2392489 |
| MOL002695 | lignan | Cardiovascular Diseases | 33761598 |
| MOL002695 | lignan | Atherosclerosis | 26215288 |
| MOL002695 | lignan | Hypertension | 23895153 |
| MOL013187 | Cubebin | Vascular Ring | 23401173 |
| MOL004903 | liquiritin | Heart Diseases | 34054527 |
| MOL004855 | Licoricone | Cardiomegaly | 32018211 |
| MOL004855 | Licoricone | Heart Failure | 32018211 |
| MOL004855 | Licoricone | Cardiovascular Diseases | 32018211 |
| MOL004911 | Glabrene | Vascular System Injuries | 15276622 |
| MOL004911 | Glabrene | Heart Diseases | 15276622 |
| MOL004911 | Glabrene | Atherosclerosis | 15276622 |
| MOL004911 | Glabrene | Cardiovascular Diseases | 15276622 |
| MOL000492 | (+)-catechin | Cardiovascular Diseases | 32637711 |
| MOL000492 | (+)-catechin | Atherosclerosis | 27689985 |
| MOL004908 | Glabridin | Atherosclerosis | 23850540 |
| MOL004908 | Glabridin | Cardiovascular Diseases | 26526087 |
| MOL004908 | Glabridin | Hyperlipidemias | 30651770 |
| MOL004879 | Glycyrin | Dyslipidemias | 12127165 |
| MOL004879 | Glycyrin | Hypertension | 14608046 |
| MOL005828 | nobiletin | Atherosclerosis | 32761293 |
| MOL013381 | Marmin | Hypercholesterolemia | 32851864 |
| MOL013381 | Marmin | Atherosclerosis | 23351957 |
| MOL013381 | Marmin | Cardiovascular Diseases | 23434131 |

**Table S7 Literature validation of mechanism of action**

| SA-mechanisms of action | | |  | UA-mechanisms of action | | |
| --- | --- | --- | --- | --- | --- | --- |
| Mol_ID | gene | PMID/DOI |  | Mol_ID | gene | PMID/DOI |
| MOL000006 | AKT1 | 25448439 |  | MOL000006 | AKT1 | 25448439 |
| MOL000006 | AR | 31805186\|33352494 | | MOL000006 | AR | 31805186\|33352494 |
| MOL000006 | BCL2L1 | 25448439 |  | MOL000006 | BCL2L1 | 25448439 |
| MOL000006 | CASP3 | 27474067 |  | MOL000006 | CASP3 | 27474067 |
| MOL000006 | CASP7 | 21074525 |  | MOL000006 | CASP9 | 21601631\|27489195 |
| MOL000006 | CASP9 | 21601631\|27489195 | | MOL000006 | CCNB1 | 18331776\|19397994 |
| MOL000006 | CCNB1 | 18331776\|19397994 | | MOL000006 | CCND1 | 31288002 |
| MOL000006 | CCND1 | 31288002 |  | MOL000006 | CD40LG | 16343431 |
| MOL000006 | CD40LG | 16343431 |  | MOL000006 | CDKN1A | 19397994 |
| MOL000006 | CDKN1A | 19397994 |  | MOL000006 | EGFR | 12168845 |
| MOL000006 | EGFR | 12168845 |  | MOL000006 | HMOX1 | 32268164 |
| MOL000006 | HMOX1 | 32268164 |  | MOL000006 | ICAM1 | 15113938\|15322261 |
| MOL000006 | ICAM1 | 15113938\|15322261 | | MOL000006 | IFNG | 12755373 |
| MOL000006 | IFNG | 12755373 |  | MOL000006 | IL10 | 30016632 |
| MOL000006 | IL10 | 30016632 |  | MOL000006 | IL4 | 16601352 |
| MOL000006 | IL4 | 16601352 |  | MOL000006 | IL6 | 25448439 |
| MOL000006 | IL6 | 25448439 |  | MOL000006 | INSR | 18591783 |
| MOL000006 | INSR | 18591783 |  | MOL000006 | JUN | 30133131 |
| MOL000006 | JUN | 30133131 |  | MOL000006 | MAPK1 | 27474067 |
| MOL000006 | MAPK1 | 27474067 |  | MOL000006 | MDM2 | 27525270 |
| MOL000006 | MDM2 | 27525270 |  | MOL000006 | MMP1 | 21112745 |
| MOL000006 | MMP1 | 21112745 |  | MOL000006 | MMP9 | 30133131 |
| MOL000006 | MMP9 | 30133131 |  | MOL000006 | NFKBIA | 25448439 |
| MOL000006 | NFKBIA | 25448439 |  | MOL000006 | PCNA | 29574863\|30298006 |
| MOL000006 | PCNA | 29574863\|30298006 | | MOL000006 | PTGS2 | 25448439 |
| MOL000006 | PTGS2 | 25448439 |  | MOL000006 | RELA | 30133131 |
| MOL000006 | RELA | 30133131 |  | MOL000006 | TNF | 25448439 |
| MOL000006 | SLC2A4 | 18591783 |  | MOL000006 | TOP1 | 12027807 |
| MOL000006 | TNF | 25448439 |  | MOL000006 | TP53 | 27525270 |
| MOL000006 | TOP1 | 12027807 |  | MOL000006 | TYR | 21071833 |
| MOL000006 | TP53 | 27525270 |  | MOL000098 | ACHE | 24952260 |
| MOL000006 | TYR | 21071833 |  | MOL000098 | AHR | 29584932 |
| MOL000098 | ACACA | 20596804 |  | MOL000098 | AKT1 | 18359480 |
| MOL000098 | ACHE | 24952260 |  | MOL000098 | AR | 27132804 |
| MOL000098 | ACP3 | 12391264 |  | MOL000098 | BAX | 24594009 |
| MOL000098 | AHR | 29584932 |  | MOL000098 | BCL2 | 24594009 |
| MOL000098 | AKT1 | 18359480 |  | MOL000098 | BCL2L1 | 18359480 |
| MOL000098 | AR | 27132804 |  | MOL000098 | BIRC5 | 18377872 |
| MOL000098 | BAX | 24594009 |  | MOL000098 | CASP3 | 24594009 |
| MOL000098 | BCL2 | 24594009 |  | MOL000098 | CASP9 | 17640809 |
| MOL000098 | BCL2L1 | 18359480 |  | MOL000098 | CAV1 | 17876056 |
| MOL000098 | BIRC5 | 18377872 |  | MOL000098 | CCNB1 | 27514524 |
| MOL000098 | CASP3 | 24594009 |  | MOL000098 | CCND1 | 27514524 |
| MOL000098 | CASP8 | 21165570 |  | MOL000098 | CD40LG | 15611226 |
| MOL000098 | CASP9 | 17640809 |  | MOL000098 | CDKN1A | 27132804 |
| MOL000098 | CAV1 | 17876056 |  | MOL000098 | CHEK2 | 19009557 |
| MOL000098 | CCNB1 | 27514524 |  | MOL000098 | CHUK | 20386985 |
| MOL000098 | CCND1 | 27514524 |  | MOL000098 | CTSD | 10376965 |
| MOL000098 | CD40LG | 15611226 |  | MOL000098 | CXCL10 | 21967610 |
| MOL000098 | CDKN1A | 27132804 |  | MOL000098 | CXCL8 | 17717114 |
| MOL000098 | CHEK2 | 19009557 |  | MOL000098 | CYP1A1 | 29584932 |
| MOL000098 | CHUK | 20386985 |  | MOL000098 | CYP1B1 | 21053930 |
| MOL000098 | CTSD | 10376965 |  | MOL000098 | CYP3A4 | 19766177 |
| MOL000098 | CXCL10 | 21967610 |  | MOL000098 | DPP4 | 17639512 |
| MOL000098 | CXCL8 | 17717114 |  | MOL000098 | E2F1 | 21632981\|27514524 |
| MOL000098 | CYP1A1 | 29584932 |  | MOL000098 | EGFR | 27514524 |
| MOL000098 | CYP1B1 | 21053930 |  | MOL000098 | ELK1 | 12888923 |
| MOL000098 | CYP3A4 | 19766177 |  | MOL000098 | ERBB2 | 18655187 |
| MOL000098 | DPP4 | 17639512 |  | MOL000098 | ERBB3 | 12888923 |
| MOL000098 | DUOX2 | 22996356 |  | MOL000098 | F10 | 21632981 |
| MOL000098 | E2F1 | 21632981\|27514524 | | MOL000098 | F2 | 21356098 |
| MOL000098 | E2F2 | 14715546\|23727915 | | MOL000098 | F3 | 12871381 |
| MOL000098 | EGFR | 27514524 |  | MOL000098 | F7 | 21632981 |
| MOL000098 | ELK1 | 12888923 |  | MOL000098 | FOS | 15090535 |
| MOL000098 | ERBB2 | 18655187 |  | MOL000098 | GJA1 | 27260466 |
| MOL000098 | ERBB3 | 12888923 |  | MOL000098 | HIF1A | 17364964 |
| MOL000098 | F10 | 21632981 |  | MOL000098 | HMOX1 | 10942197 |
| MOL000098 | F2 | 21356098 |  | MOL000098 | HSPB1 | 24126416 |
| MOL000098 | F3 | 12871381 |  | MOL000098 | ICAM1 | 18394220 |
| MOL000098 | F7 | 21632981 |  | MOL000098 | IFNG | 18394220 |
| MOL000098 | FOS | 15090535 |  | MOL000098 | IGF2 | 20658310 |
| MOL000098 | GJA1 | 27260466 |  | MOL000098 | IGFBP3 | 17103110 |
| MOL000098 | HIF1A | 17364964 |  | MOL000098 | IL10 | 17717114 |
| MOL000098 | HK2 | 15661808 |  | MOL000098 | IL1A | 16532021 |
| MOL000098 | HMOX1 | 10942197 |  | MOL000098 | IL1B | 21447443 |
| MOL000098 | HSPB1 | 24126416 |  | MOL000098 | IL2 | 19880376 |
| MOL000098 | ICAM1 | 18394220 |  | MOL000098 | IL6 | 16150460 |
| MOL000098 | IFNG | 18394220 |  | MOL000098 | INSR | 21439094 |
| MOL000098 | IGF2 | 20658310 |  | MOL000098 | JUN | 21356098 |
| MOL000098 | IGFBP3 | 17103110 |  | MOL000098 | MAOB | 21632981 |
| MOL000098 | IL10 | 17717114 |  | MOL000098 | MAPK1 | 16611395 |
| MOL000098 | IL1A | 16532021 |  | MOL000098 | MMP1 | 11414687 |
| MOL000098 | IL1B | 21447443 |  | MOL000098 | MMP2 | 24001813 |
| MOL000098 | IL2 | 19880376 |  | MOL000098 | MMP3 | 22592909 |
| MOL000098 | IL6 | 16150460 |  | MOL000098 | MMP9 | 32530119 |
| MOL000098 | INSR | 21439094 |  | MOL000098 | MPO | 24908156 |
| MOL000098 | JUN | 21356098 |  | MOL000098 | MYC | 15670774\|21632981\|23727915\|9381980 |
| MOL000098 | MAOB | 21632981 |  | MOL000098 | NCOA2 | 21632981 |
| MOL000098 | MAPK1 | 16611395 |  | MOL000098 | NFE2L2 | 17433488 |
| MOL000098 | MMP1 | 11414687 |  | MOL000098 | NFKBIA | 18852136 |
| MOL000098 | MMP2 | 24001813 |  | MOL000098 | NOS3 | 22488414 |
| MOL000098 | MMP3 | 22592909 |  | MOL000098 | NR1I2 | 26238175 |
| MOL000098 | MMP9 | 32530119 |  | MOL000098 | ODC1 | 11238180 |
| MOL000098 | MPO | 24908156 |  | MOL000098 | PARP1 | 14688022\|21165570 |
| MOL000098 | MYC | 15670774\|21632981\|23727915\|9381980 | | MOL000098 | PLAT | 18419748 |
| MOL000098 | NCOA2 | 21632981 |  | MOL000098 | PLAU | 11236827 |
| MOL000098 | NFE2L2 | 17433488 |  | MOL000098 | PON1 | 15169886 |
| MOL000098 | NFKBIA | 18852136 |  | MOL000098 | PPARA | 23647015 |
| MOL000098 | NKX3-1 | 25380086 |  | MOL000098 | PPARD | 18393431 |
| MOL000098 | NOS3 | 22488414 |  | MOL000098 | PPARG | 18586010 |
| MOL000098 | NR1I2 | 26238175 |  | MOL000098 | PRKACA | 22174042 |
| MOL000098 | ODC1 | 11238180 |  | MOL000098 | PRKCA | 26311153 |
| MOL000098 | PARP1 | 14688022\|21165570 | | MOL000098 | PRKCB | 14750173 |
| MOL000098 | PLAT | 18419748 |  | MOL000098 | PTGS1 | 22996356 |
| MOL000098 | PLAU | 11236827 |  | MOL000098 | PTGS2 | 14988445\|15225597 |
| MOL000098 | PON1 | 15169886 |  | MOL000098 | RAF1 | 12888923 |
| MOL000098 | PPARA | 23647015 |  | MOL000098 | RASSF1 | 21632981 |
| MOL000098 | PPARD | 18393431 |  | MOL000098 | RB1 | 25070040 |
| MOL000098 | PPARG | 18586010 |  | MOL000098 | RELA | 20386985 |
| MOL000098 | PRKACA | 22174042 |  | MOL000098 | RUNX2 | 16996034 |
| MOL000098 | PRKCA | 26311153 |  | MOL000098 | SCN5A | 19207037 |
| MOL000098 | PRKCB | 14750173 |  | MOL000098 | SELE | 18394220 |
| MOL000098 | PTGS1 | 22996356 |  | MOL000098 | SOD1 | 17640809 |
| MOL000098 | PTGS2 | 14988445\|15225597 | | MOL000098 | SPP1 | 20596804 |
| MOL000098 | RAF1 | 12888923 |  | MOL000098 | TNF | 26752173 |
| MOL000098 | RASA1 | 21632981 |  | MOL000098 | TOP2A | 21632981 |
| MOL000098 | RASSF1 | 21632981 |  | MOL000098 | TP53 | 15795422 |
| MOL000098 | RB1 | 25070040 |  | MOL000098 | VCAM1 | 18394220 |
| MOL000098 | RELA | 20386985 |  | MOL000173 | BAX | 20570612 |
| MOL000098 | RUNX2 | 16996034 |  | MOL000173 | BCL2 | 28688942 |
| MOL000098 | SCN5A | 19207037 |  | MOL000173 | CASP3 | 20570612 |
| MOL000098 | SELE | 18394220 |  | MOL000173 | CASP9 | 28688942 |
| MOL000098 | SLC2A4 | 24343960 |  | MOL000173 | CCND1 | 21457722\|23872260 |
| MOL000098 | SOD1 | 17640809 |  | MOL000173 | CDKN1A | 32671444 |
| MOL000098 | SPP1 | 20596804 |  | MOL000173 | CXCL8 | 32671444 |
| MOL000098 | STAT1 | 16077199 |  | MOL000173 | GSK3B | 23872260 |
| MOL000098 | TNF | 26752173 |  | MOL000173 | IL6 | 32671444 |
| MOL000098 | TOP2A | 21632981 |  | MOL000173 | JUN | 17570322 |
| MOL000098 | TP53 | 15795422 |  | MOL000173 | MAPK14 | 22057676\|26073326 |
| MOL000098 | VCAM1 | 18394220 |  | MOL000173 | NOS2 | 26892447 |
| MOL000173 | BAX | 20570612 |  | MOL000173 | PPARG | 31103702 |
| MOL000173 | BCL2 | 28688942 |  | MOL000173 | PTGS2 | 26892447 |
| MOL000173 | CASP3 | 20570612 |  | MOL000173 | RELA | 32671444 |
| MOL000173 | CASP9 | 28688942 |  | MOL000173 | TNF | 26213241 |
| MOL000173 | CCND1 | 21457722\|23872260 | | MOL000173 | TP53 | 21457722 |
| MOL000173 | CDKN1A | 32671444 |  | MOL000354 | ACHE | 31698018\|28767066 |
| MOL000173 | CXCL8 | 32671444 |  | MOL000354 | CYP1B1 | 19794518 |
| MOL000173 | GSK3B | 23872260 |  | MOL000354 | ESR1 | 14706564 |
| MOL000173 | IL6 | 32671444 |  | MOL000354 | MAPK14 | 31586635\|31467486 |
| MOL000173 | JUN | 17570322 |  | MOL000354 | NOS2 | 20579867 |
| MOL000173 | MAPK14 | 22057676\|26073326 | | MOL000354 | OLR1 | 16891912 |
| MOL000173 | NOS2 | 26892447 |  | MOL000354 | PPARD | 22992727 |
| MOL000173 | PPARG | 31103702 |  | MOL000354 | PPARG | 22992727 |
| MOL000173 | PTGS2 | 26892447 |  | MOL000354 | PTGS2 | 15292928 |
| MOL000173 | RELA | 32671444 |  | MOL000354 | RELA | 18274639 |
| MOL000173 | TNF | 26213241 |  | MOL000392 | AR | 23982890\|12711012 |
| MOL000173 | TP53 | 21457722 |  | MOL000392 | ESR1 | 18980245 |
| MOL000354 | ACHE | 31698018\|28767066 | | MOL000392 | IL4 | 16108819 |
| MOL000354 | CYP1B1 | 19794518 |  | MOL000392 | MAPK14 | 30243826\|31175839 |
| MOL000354 | ESR1 | 14706564 |  | MOL000392 | PPARG | 19924040 |
| MOL000354 | ESR2 | 14706564 |  | MOL000417 | ESR1 | 20686605 |
| MOL000354 | MAPK14 | 31586635\|31467486 | | MOL000417 | MAPK14 | 32901836\|32449282 |
| MOL000354 | NOS2 | 20579867 |  | MOL000422 | ACHE | 30497708 |
| MOL000354 | OLR1 | 16891912 |  | MOL000422 | AHR | 17012224 |
| MOL000354 | PPARD | 22992727 |  | MOL000422 | AKT1 | 19244381 |
| MOL000354 | PPARG | 22992727 |  | MOL000422 | AR | 33049310 |
| MOL000354 | PTGS2 | 15292928 |  | MOL000422 | BAX | 19028473 |
| MOL000354 | RELA | 18274639 |  | MOL000422 | BCL2 | 19028473 |
| MOL000392 | AR | 23982890\|12711012 | | MOL000422 | CASP3 | 16014620 |
| MOL000392 | ESR1 | 18980245 |  | MOL000422 | CYP1A1 | 21256954\|21329749 |
| MOL000392 | ESR2 | 16076101\|9751507 | | MOL000422 | CYP1B1 | 21053930 |
| MOL000392 | IL4 | 16108819 |  | MOL000422 | CYP3A4 | 15266218 |
| MOL000392 | MAPK14 | 30243826\|31175839 | | MOL000422 | HMOX1 | 25111660 |
| MOL000392 | PPARG | 19924040 |  | MOL000422 | ICAM1 | 18394220 |
| MOL000417 | ESR1 | 20686605 |  | MOL000422 | JUN | 20594614 |
| MOL000417 | ESR2 | 20686605 |  | MOL000422 | MAPK8 | 30499162 |
| MOL000417 | MAPK14 | 32901836\|32449282 | | MOL000422 | NOS2 | 18394220 |
| MOL000422 | ACHE | 30497708 |  | MOL000422 | NR1I2 | 33049310 |
| MOL000422 | AHR | 17012224 |  | MOL000422 | PGR | 23384675 |
| MOL000422 | AKT1 | 19244381 |  | MOL000422 | PPARG | 33049310 |
| MOL000422 | AR | 33049310 |  | MOL000422 | PTGS2 | 18394220 |
| MOL000422 | BAX | 19028473 |  | MOL000422 | RELA | 20438634 |
| MOL000422 | BCL2 | 19028473 |  | MOL000422 | SELE | 18394220 |
| MOL000422 | CASP3 | 16014620 |  | MOL000422 | TNF | 18394220 |
| MOL000422 | CYP1A1 | 21256954\|21329749 | | MOL000422 | VCAM1 | 18394220 |
| MOL000422 | CYP1B1 | 21053930 |  | MOL000492 | PTGS1 | 11962253 |
| MOL000422 | CYP3A4 | 15266218 |  | MOL000492 | PTGS2 | 19557821 |
| MOL000422 | HMOX1 | 25111660 |  | MOL000497 | BCL2 | 30055311 |
| MOL000422 | ICAM1 | 18394220 |  | MOL000497 | CCND1 | 30055311 |
| MOL000422 | JUN | 20594614 |  | MOL000497 | MAPK1 | 30983163 |
| MOL000422 | MAPK8 | 30499162 |  | MOL000497 | NOS2 | 26134484 |
| MOL000422 | NOS2 | 18394220 |  | MOL000497 | STAT3 | 18848530 |
| MOL000422 | NR1I2 | 33049310 |  | MOL001002 | CDKN1A | 15735102 |
| MOL000422 | PGR | 23384675 |  | MOL001002 | CSNK2A1 | 10.1016/j.bmcl.2009.04.076\|10.1021/jm060112m\|10.1093/nar/28.1.235 |
| MOL000422 | PPARG | 33049310 |  | MOL001002 | CXCL8 | 18377686 |
| MOL000422 | PTGS2 | 18394220 |  | MOL001002 | MMP2 | 21573219 |
| MOL000422 | RELA | 20438634 |  | MOL001002 | MMP9 | 22485181 |
| MOL000422 | SELE | 18394220 |  | MOL001002 | PRKCB | 10.1055/s-2006-957407 |
| MOL000422 | SLC2A4 | 18591783 |  | MOL001002 | RELA | 12002526 |
| MOL000422 | STAT1 | 18274639 |  | MOL001002 | SYK | 10.1021/jm060112m |
| MOL000422 | TNF | 18394220 |  | MOL001454 | ESR1 | 30818834 |
| MOL000422 | VCAM1 | 18394220 |  | MOL001454 | KCNH2 | 30086269 |
| MOL000492 | PTGS1 | 11962253 |  | MOL001454 | NOS2 | 21095217 |
| MOL000492 | PTGS2 | 19557821 |  | MOL001454 | PTGS2 | 21095217 |
| MOL000497 | BCL2 | 30055311 |  | MOL001454 | TERT | 25265580 |
| MOL000497 | CCND1 | 30055311 |  | MOL001689 | CYP1B1 | 21053930 |
| MOL000497 | MAPK1 | 30983163 |  | MOL001689 | RELA | 10.1016/j.bmcl.2014.07.093 |
| MOL000497 | NOS2 | 26134484 |  | MOL001792 | ESR1 | 25106122 |
| MOL000497 | STAT3 | 18848530 |  | MOL002135 | KCNH2 | 32922187 |
| MOL001002 | CDKN1A | 15735102 |  | MOL002311 | NOS2 | 18666753 |
| MOL001002 | CSNK2A1 | 10.1016/j.bmcl.2009.04.076\|10.1021/jm060112m\|10.1093/nar/28.1.235 | | MOL002311 | PTGS2 | 18666753 |
| MOL001002 | CXCL8 | 18377686 |  | MOL002341 | PTGS2 | 16101151 |
| MOL001002 | MMP2 | 21573219 |  | MOL002714 | AHR | 22820424 |
| MOL001002 | MMP9 | 22485181 |  | MOL002714 | AKT1 | 22820424 |
| MOL001002 | PRKCB | 10.1055/s-2006-957407 | | MOL002714 | BAX | 21457722 |
| MOL001002 | RELA | 12002526 |  | MOL002714 | BCL2 | 20850421 |
| MOL001002 | SYK | 10.1021/jm060112m | | MOL002714 | CASP3 | 20850421 |
| MOL001454 | ESR1 | 30818834 |  | MOL002714 | CCNB1 | 18025287 |
| MOL001454 | KCNH2 | 30086269 |  | MOL002714 | CYCS | 20850421 |
| MOL001454 | NOS2 | 21095217 |  | MOL002714 | MMP9 | 19804834 |
| MOL001454 | PTGS2 | 21095217 |  | MOL002714 | MPO | 27780710 |
| MOL001454 | TERT | 25265580 |  | MOL002714 | PTGS2 | 19804834 |
| MOL001689 | CYP1B1 | 21053930 |  | MOL002714 | RELA | 29481769 |
| MOL001689 | RELA | 10.1016/j.bmcl.2014.07.093 | | MOL002714 | TNF | 22369883 |
| MOL001792 | ESR1 | 25106122 |  | MOL002714 | TP53 | 21457722 |
| MOL002135 | KCNH2 | 32922187 |  | MOL004328 | ABCC1 | 11306701 |
| MOL002135 | NCOA1 | 32922187 |  | MOL004328 | AKT1 | 22692793 |
| MOL002311 | NOS2 | 18666753 |  | MOL004328 | BCL2 | 19124070 |
| MOL002311 | PTGS2 | 18666753 |  | MOL004328 | CASP3 | 31254498 |
| MOL002341 | PTGS2 | 16101151 |  | MOL004328 | CYP19A1 | 9435150 |
| MOL002565 | ESR2 | 21333515 |  | MOL004328 | CYP1B1 | 21053930 |
| MOL002714 | AHR | 22820424 |  | MOL004328 | ESR1 | 11162928 |
| MOL002714 | AKT1 | 22820424 |  | MOL004328 | GSR | 24561720 |
| MOL002714 | BAX | 21457722 |  | MOL004328 | MAPK1 | 25866363 |
| MOL002714 | BCL2 | 20850421 |  | MOL004328 | MAPK3 | 25866363 |
| MOL002714 | CASP3 | 20850421 |  | MOL004328 | PPARA | 31063766 |
| MOL002714 | CCNB1 | 18025287 |  | MOL004328 | PPARG | 31063766 |
| MOL002714 | CYCS | 20850421 |  | MOL004328 | PTGS1 | 24561720 |
| MOL002714 | MMP9 | 19804834 |  | MOL004328 | PTGS2 | 21341175 |
| MOL002714 | MPO | 27780710 |  | MOL004328 | RELA | 18274639 |
| MOL002714 | PTGS2 | 19804834 |  | MOL004328 | SOD1 | 23192364 |
| MOL002714 | RELA | 29481769 |  | MOL004814 | MAPK14 | 28192731 |
| MOL002714 | TNF | 22369883 |  | MOL004820 | PPARG | 10.1016/j.bmc.2009.11.027 |
| MOL002714 | TP53 | 21457722 |  | MOL004841 | ADRB2 | 33647349 |
| MOL004328 | ABCC1 | 11306701 |  | MOL004841 | CCNA2 | 24384411 |
| MOL004328 | AKT1 | 22692793 |  | MOL004841 | NOS2 | 25451593 |
| MOL004328 | BCL2 | 19124070 |  | MOL004908 | MAPK14 | 33178017 |
| MOL004328 | CASP3 | 31254498 |  | MOL004912 | ACHE | 32614708 |
| MOL004328 | CYP19A1 | 9435150 |  | MOL005017 | MAPK14 | 28192731 |
| MOL004328 | CYP1B1 | 21053930 |  | MOL005828 | BAX | 29687528 |
| MOL004328 | ESR1 | 11162928 |  | MOL005828 | BCL2 | 29687528 |
| MOL004328 | ESR2 | 9751507 |  | MOL005828 | CASP9 | 33058988 |
| MOL004328 | GSR | 24561720 |  | MOL005828 | MAPK8 | 33660406 |
| MOL004328 | MAPK1 | 25866363 |  |  |  |  |
| MOL004328 | MAPK3 | 25866363 |  |  |  |  |
| MOL004328 | PPARA | 31063766 |  |  |  |  |
| MOL004328 | PPARG | 31063766 |  |  |  |  |
| MOL004328 | PTGS1 | 24561720 |  |  |  |  |
| MOL004328 | PTGS2 | 21341175 |  |  |  |  |
| MOL004328 | RELA | 18274639 |  |  |  |  |
| MOL004328 | SOD1 | 23192364 |  |  |  |  |
| MOL004814 | MAPK14 | 28192731 |  |  |  |  |
| MOL004820 | PPARG | 10.1016/j.bmc.2009.11.027 | | |  |  |
| MOL004841 | ADRB2 | 33647349 |  |  |  |  |
| MOL004841 | CCNA2 | 24384411 |  |  |  |  |
| MOL004841 | NOS2 | 25451593 |  |  |  |  |
| MOL004841 | PDE3A | 33647349 |  |  |  |  |
| MOL004908 | ESR2 | 21573846 |  |  |  |  |
| MOL004908 | MAPK14 | 33178017 |  |  |  |  |
| MOL004911 | ESR2 | 21810473 |  |  |  |  |
| MOL004912 | ACHE | 32614708 |  |  |  |  |
| MOL005017 | MAPK14 | 28192731 |  |  |  |  |
| MOL005828 | BAX | 29687528 |  |  |  |  |
| MOL005828 | BCL2 | 29687528 |  |  |  |  |
| MOL005828 | CASP9 | 33058988 |  |  |  |  |
| MOL005828 | MAPK8 | 33660406 |  |  |  |  |

**Part 5 contains Table S8, Table S9, Table S10, Table S11, Figure S1 and Figure S2.**

**Table S8 Modularity index**

|  | algorithm | modularity |
| --- | --- | --- |
| SA | cluster_fast_greedy | 0.19762 |
|  | cluster_infomap | 0.01907742 |
|  | cluster_walktrap | 0.1715528 |
| UA | cluster_fast_greedy | 0.1883773 |
|  | cluster_infomap | 4.6629E-15 |
|  | cluster_walktrap | 0.1667689 |

**Table S9 Score for Stable angina pathways**

| SA-ID | FoldEnrichment | S | RWR Score |
| --- | --- | --- | --- |
| hsa00140 | 0.190463393 | 0.013778 | 0.2349932 |
| hsa00330 | 0.099784176 | 0 | 0.1298634 |
| hsa00590 | 0.108611356 | 0.012859 | 0.3265124 |
| hsa01521 | 0.441200012 | 0.368754 | 0.0467921 |
| hsa01522 | 0.540840224 | 0.513078 | 0.0906744 |
| hsa01523 | 0.467704163 | 0.068888 | 0.0336509 |
| hsa01524 | 0.519553431 | 0.350053 | 0.1634205 |
| hsa03320 | 0.133920868 | 0.041333 | 0.2021401 |
| hsa04010 | 0.201182112 | 0.825738 | 0.060872 |
| hsa04012 | 0.373908252 | 0.31344 | 0.0169897 |
| hsa04014 | 0.151477833 | 0.50458 | 0.061576 |
| hsa04015 | 0.034749638 | 0.225034 | 0.0446802 |
| hsa04020 | 0.112021858 | 0.360055 | 0.4039518 |
| hsa04022 | 0.128216635 | 0.23575 | 0.21622 |
| hsa04024 | 0.131970956 | 0.385773 | 0.3265124 |
| hsa04060 | 0.013995951 | 0.089025 | 0.0228563 |
| hsa04061 | 0.078735363 | 0.013778 | 0.0127658 |
| hsa04062 | 0.125024395 | 0.368754 | 0.027315 |
| hsa04064 | 0.336065574 | 0.400061 | 0.0613413 |
| hsa04066 | 0.362052296 | 0.385773 | 0.0782372 |
| hsa04068 | 0.30418149 | 0.390365 | 0.0357629 |
| hsa04071 | 0.260622282 | 0.34417 | 0.0376402 |
| hsa04072 | 0.089530983 | 0.204577 | 0.0763599 |
| hsa04110 | 0.205975674 | 0.2829 | 0.1089783 |
| hsa04114 | 0.058802171 | 0.112517 | 0.3781386 |
| hsa04115 | 0.41695807 | 0.2829 | 0.0723706 |
| hsa04137 | 0.234398678 | 0.142879 | 0.0282536 |
| hsa04140 | 0.122651669 | 0.23575 | 0.0221523 |
| hsa04150 | 0.081151318 | 0.204577 | 0.0108884 |
| hsa04151 | 0.164913534 | 0.860169 | 0.181255 |
| hsa04152 | 0.174434036 | 0.244817 | 0.2054254 |
| hsa04210 | 0.399607384 | 0.602473 | 0.0399869 |
| hsa04211 | 0.156435018 | 0.125019 | 0.067208 |
| hsa04215 | 0.762148712 | 0.163661 | 0.0247337 |
| hsa04217 | 0.139498917 | 0.212175 | 0.0397522 |
| hsa04218 | 0.304059329 | 0.567086 | 0.0787065 |
| hsa04261 | 0.136986729 | 0.229627 | 0.3053926 |
| hsa04270 | 0.110457643 | 0.143204 | 0.1223542 |
| hsa04310 | 0.144606529 | 0.337551 | 0.0794105 |
| hsa04350 | 0.063331506 | 0.032148 | 0.0073685 |
| hsa04360 | 0.027433924 | 0.160739 | 0.0676773 |
| hsa04370 | 0.496368039 | 0.275552 | 0.0686159 |
| hsa04371 | 0.104429136 | 0.143204 | 0.0451495 |
| hsa04380 | 0.352568794 | 0.531137 | 0.0505468 |
| hsa04510 | 0.152387943 | 0.411491 | 0.0510161 |
| hsa04520 | 0.220437378 | 0.142879 | 0.1530952 |
| hsa04540 | 0.159303811 | 0.160739 | 0.0327123 |
| hsa04550 | 0.061102832 | 0.142879 | 0.021683 |
| hsa04610 | 0.109574322 | 0.082666 | 0.6268832 |
| hsa04611 | 0.105310871 | 0.173598 | 0.1831323 |
| hsa04613 | 0.048514729 | 0.184119 | 0.0498428 |
| hsa04620 | 0.408079625 | 0.390365 | 0.020275 |
| hsa04621 | 0.166043448 | 0.406077 | 0.0193364 |
| hsa04622 | 0.296286383 | 0.163661 | 0.0127658 |
| hsa04623 | 0.181368722 | 0.068888 | 0 |
| hsa04625 | 0.528103044 | 0.544039 | 0.0404562 |
| hsa04630 | 0.150547894 | 0.241108 | 0.0327123 |
| hsa04650 | 0.151724261 | 0.200305 | 0.020275 |
| hsa04657 | 0.594498979 | 0.462433 | 0.0599334 |
| hsa04658 | 0.365288667 | 0.319586 | 0.0193364 |
| hsa04659 | 0.463940992 | 0.482216 | 0.0552401 |
| hsa04660 | 0.432084309 | 0.420843 | 0.0263763 |
| hsa04662 | 0.360655738 | 0.353625 | 0.0214484 |
| hsa04664 | 0.344537815 | 0.171455 | 0.0221523 |
| hsa04666 | 0.13561409 | 0.142879 | 0.0172244 |
| hsa04668 | 0.505812981 | 0.492932 | 0.0496081 |
| hsa04670 | 0.057274333 | 0.05511 | 0.0404562 |
| hsa04672 | 0.158724848 | 0.00643 | 0.0148777 |
| hsa04713 | 0.10987711 | 0.128591 | 0.1176609 |
| hsa04720 | 0.202069279 | 0.128591 | 0.1148449 |
| hsa04722 | 0.260622282 | 0.34417 | 0.0247337 |
| hsa04723 | 0.072662827 | 0.173598 | 0.1324447 |
| hsa04724 | 0.079173343 | 0.112517 | 0.1204768 |
| hsa04725 | 0.213280554 | 0.298515 | 0.4790444 |
| hsa04726 | 0.164484268 | 0.23575 | 0.3241658 |
| hsa04728 | 0.168760202 | 0.298515 | 0.1702257 |
| hsa04730 | 0.195238095 | 0.082666 | 0.1188342 |
| hsa04750 | 0.13325049 | 0.125019 | 0.0214484 |
| hsa04910 | 0.231986872 | 0.400061 | 0.1786737 |
| hsa04912 | 0.306640477 | 0.259325 | 0.0294269 |
| hsa04913 | 0.442439271 | 0.081831 | 0.2016708 |
| hsa04914 | 0.178594848 | 0.184119 | 0.2537664 |
| hsa04915 | 0.283881478 | 0.41639 | 0.448538 |
| hsa04916 | 0.126440513 | 0.160739 | 0.0550054 |
| hsa04917 | 0.474606892 | 0.31344 | 0.0939597 |
| hsa04918 | 0.103700234 | 0.042864 | 0.0848078 |
| hsa04919 | 0.295991639 | 0.482216 | 0.2256066 |
| hsa04920 | 0.37433391 | 0.200305 | 0.0535974 |
| hsa04921 | 0.179567505 | 0.34417 | 0.0390482 |
| hsa04922 | 0.067302852 | 0.082666 | 0.060168 |
| hsa04923 | 0.210567402 | 0.082666 | 0.22326 |
| hsa04924 | 0.121067101 | 0.042864 | 0.2157507 |
| hsa04925 | 0.082301773 | 0.068888 | 0.0646266 |
| hsa04926 | 0.291033531 | 0.385773 | 0.0526588 |
| hsa04927 | 0.096018735 | 0.019289 | 0.0751866 |
| hsa04929 | 0.216042155 | 0.096443 | 0.060872 |
| hsa04930 | 0.500967315 | 0.163661 | 0.0345896 |
| hsa04931 | 0.366293694 | 0.411491 | 0.136434 |
| hsa04932 | 0.322746846 | 0.489635 | 0.0824612 |
| hsa04933 | 0.702857143 | 0.703232 | 0.0550054 |
| hsa04934 | 0.145576792 | 0.2829 | 0.0740132 |
| hsa04940 | 0.252328305 | 0.021432 | 0.0030037 |
| hsa04960 | 0.308817014 | 0.064295 | 1 |
| hsa04961 | 0.092395387 | 0 | 0.0878585 |
| hsa04970 | 0.145576792 | 0.089299 | 0.2396865 |
| hsa04971 | 0.068223838 | 0.025718 | 0.4837377 |
| hsa04976 | 0.072283767 | 0.05358 | 0.5072042 |
| hsa05010 | 0.093416602 | 0.63377 | 0.1706951 |
| hsa05012 | 0.03431995 | 0.244817 | 0.107101 |
| hsa05014 | 0 | 0.252589 | 0.1723377 |
| hsa05016 | 0.010041175 | 0.244817 | 0.183367 |
| hsa05020 | 0.105163377 | 0.447146 | 0.0848078 |
| hsa05022 | 0.082677185 | 0.765873 | 0.2514197 |
| hsa05030 | 0.260622282 | 0.096443 | 0.2349932 |
| hsa05031 | 0.265790992 | 0.192886 | 0.1941615 |
| hsa05120 | 0.331950485 | 0.23575 | 0.0148777 |
| hsa05130 | 0.195449304 | 0.451466 | 0.0599334 |
| hsa05131 | 0.147540984 | 0.562585 | 0.0284883 |
| hsa05132 | 0.144606529 | 0.509006 | 0.0214484 |
| hsa05133 | 0.49525453 | 0.275042 | 0.0364669 |
| hsa05134 | 0.385759481 | 0.163661 | 0.0165204 |
| hsa05135 | 0.286654473 | 0.41639 | 0.0268456 |
| hsa05140 | 0.487575656 | 0.300046 | 0.0519548 |
| hsa05142 | 0.442439271 | 0.368238 | 0.0329469 |
| hsa05143 | 0.646180138 | 0.102288 | 0.023091 |
| hsa05144 | 0.503138173 | 0.128591 | 0.0428028 |
| hsa05145 | 0.43894279 | 0.509006 | 0.0430375 |
| hsa05146 | 0.246636359 | 0.183701 | 0.0463228 |
| hsa05152 | 0.26458496 | 0.516837 | 0.0402215 |
| hsa05160 | 0.396918212 | 0.671992 | 0.0472615 |
| hsa05161 | 0.535808252 | 0.928364 | 0.0592294 |
| hsa05162 | 0.388910417 | 0.602473 | 0.0359976 |
| hsa05163 | 0.3256102 | 0.920441 | 0.0460881 |
| hsa05164 | 0.266842765 | 0.486074 | 0.020275 |
| hsa05165 | 0.122706722 | 0.609699 | 0.0824612 |
| hsa05166 | 0.302963224 | 0.683369 | 0.0916131 |
| hsa05167 | 0.382582422 | 0.917514 | 0.0498428 |
| hsa05168 | 0.004241792 | 0.385773 | 0.0240297 |
| hsa05169 | 0.287105526 | 0.671992 | 0.0463228 |
| hsa05170 | 0.233705979 | 0.661325 | 0.0540667 |
| hsa05171 | 0.194520714 | 0.520317 | 0.0728399 |
| hsa05202 | 0.177034543 | 0.364341 | 0.1120289 |
| hsa05203 | 0.136497222 | 0.431457 | 0.0819918 |
| hsa05204 | 0.084886128 | 0.012859 | 0.1671751 |
| hsa05205 | 0.281498829 | 0.671992 | 0.097949 |
| hsa05206 | 0.129470424 | 0.68888 | 0.0873891 |
| hsa05207 | 0.292585392 | 0.734527 | 0.3499789 |
| hsa05210 | 0.513588584 | 0.442414 | 0.0315389 |
| hsa05211 | 0.193429047 | 0.112517 | 0.0101844 |
| hsa05212 | 0.659497103 | 0.50458 | 0.0221523 |
| hsa05213 | 0.506581604 | 0.252589 | 0.0179284 |
| hsa05214 | 0.403278689 | 0.306475 | 0.0127658 |
| hsa05215 | 0.598879741 | 0.602473 | 0.0906744 |
| hsa05216 | 0.511234888 | 0.125019 | 0.067208 |
| hsa05218 | 0.389409316 | 0.275552 | 0.0132351 |
| hsa05219 | 1 | 0.350053 | 0.0388135 |
| hsa05220 | 0.528103044 | 0.406077 | 0.0186324 |
| hsa05221 | 0.42563529 | 0.298515 | 0.0819918 |
| hsa05222 | 0.500967315 | 0.499751 | 0.0686159 |
| hsa05223 | 0.597449909 | 0.462927 | 0.0362322 |
| hsa05224 | 0.260622282 | 0.442414 | 0.2654996 |
| hsa05225 | 0.275482324 | 0.513078 | 0.0831652 |
| hsa05226 | 0.239080207 | 0.411491 | 0.066504 |
| hsa05230 | 0.296286383 | 0.225034 | 0.0139391 |
| hsa05231 | 0.235147923 | 0.244817 | 0.0130004 |
| hsa05235 | 0.436939189 | 0.456836 | 0.0296616 |
| hsa05310 | 0.226108635 | 0 | 0.0134698 |
| hsa05320 | 0.139498917 | 0.00643 | 0.0183977 |
| hsa05321 | 0.441686183 | 0.160739 | 0.0226217 |
| hsa05323 | 0.199264687 | 0.102288 | 0.022387 |
| hsa05330 | 0.363860471 | 0.013778 | 0.0179284 |
| hsa05332 | 0.320062451 | 0.027555 | 0.0040128 |
| hsa05415 | 0.149940587 | 0.360055 | 0.0885624 |
| hsa05416 | 0.236846214 | 0.096443 | 0.0207444 |
| hsa05417 | 0.449724961 | 1 | 0.1190689 |
| hsa05418 | 0.406870756 | 0.523549 | 0.0850425 |


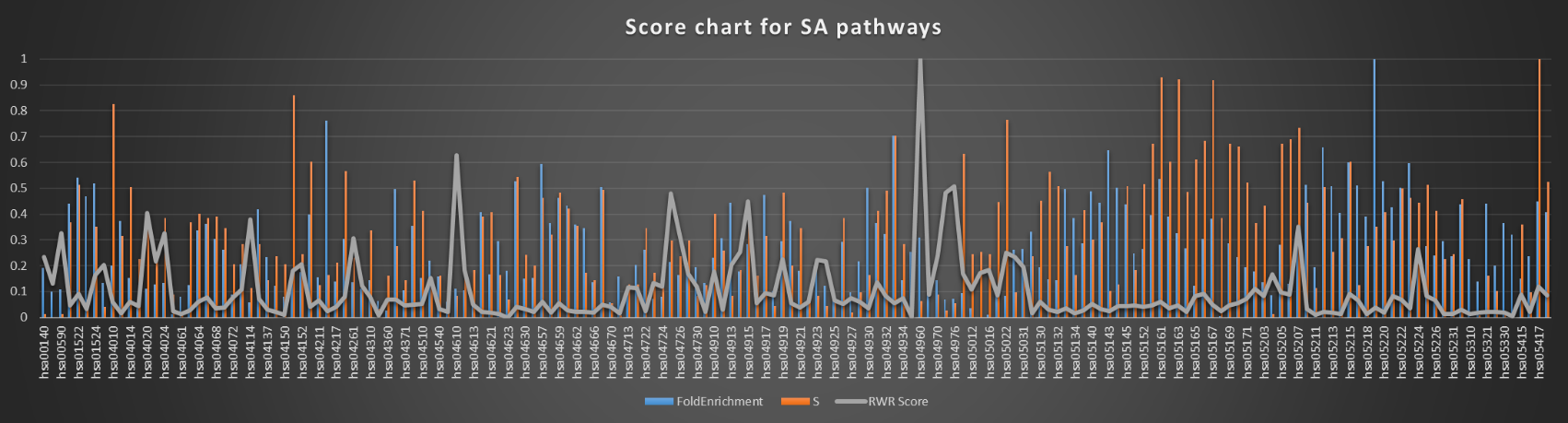


**Figure S1 Score chart of the pathways for stable angina pectoris**

**Table S10 Score for Unstable angina pathways**

| UA-ID | RWR Score | FoldEnrichment | S |
| --- | --- | --- | --- |
| hsa00140 | 0.289524564 | 0.165329 | 0.032353 |
| hsa00330 | 0.151389504 | 0.112734 | 0 |
| hsa00590 | 0.387360526 | 0.122065 | 0.019412 |
| hsa01521 | 0.049127629 | 0.440244 | 0.339706 |
| hsa01522 | 0.070791307 | 0.525123 | 0.534527 |
| hsa01523 | 0.034685178 | 0.501669 | 0.069328 |
| hsa01524 | 0.171655524 | 0.484176 | 0.339706 |
| hsa03320 | 0.210556966 | 0.114096 | 0.032353 |
| hsa04010 | 0.063570081 | 0.192994 | 0.792647 |
| hsa04012 | 0.018379184 | 0.402515 | 0.363971 |
| hsa04014 | 0.064268909 | 0.144629 | 0.497619 |
| hsa04015 | 0.046565259 | 0.031418 | 0.194118 |
| hsa04020 | 0.266230287 | 0.059693 | 0.277311 |
| hsa04022 | 0.067297165 | 0.063776 | 0.174706 |
| hsa04024 | 0.142770621 | 0.098557 | 0.339706 |
| hsa04060 | 0.026299238 | 0.022046 | 0.134389 |
| hsa04061 | 0.014186214 | 0.090483 | 0.041597 |
| hsa04062 | 0.034685178 | 0.139416 | 0.420588 |
| hsa04064 | 0.068228936 | 0.362511 | 0.402614 |
| hsa04066 | 0.08453493 | 0.36577 | 0.383127 |
| hsa04068 | 0.033520464 | 0.30866 | 0.414118 |
| hsa04071 | 0.040042861 | 0.282759 | 0.371107 |
| hsa04072 | 0.082438445 | 0.101895 | 0.226471 |
| hsa04110 | 0.125299914 | 0.203708 | 0.277311 |
| hsa04114 | 0.394348808 | 0.069411 | 0.113235 |
| hsa04115 | 0.082205502 | 0.411872 | 0.277311 |
| hsa04137 | 0.032122807 | 0.255037 | 0.161765 |
| hsa04140 | 0.025134524 | 0.136907 | 0.258824 |
| hsa04150 | 0.011157958 | 0.093037 | 0.205882 |
| hsa04151 | 0.081273731 | 0.151763 | 0.796078 |
| hsa04152 | 0.214982879 | 0.12567 | 0.194118 |
| hsa04210 | 0.041906404 | 0.390872 | 0.597882 |
| hsa04211 | 0.073120734 | 0.17262 | 0.143791 |
| hsa04213 | 0.099210324 | 0.07601 | 0 |
| hsa04215 | 0.023270982 | 0.647989 | 0.143791 |
| hsa04217 | 0.021873326 | 0.104923 | 0.172549 |
| hsa04218 | 0.089426728 | 0.294842 | 0.534527 |
| hsa04261 | 0.284865708 | 0.09928 | 0.205882 |
| hsa04270 | 0.016515642 | 0.064489 | 0.129412 |
| hsa04310 | 0.092454984 | 0.144218 | 0.332157 |
| hsa04340 | 0.028628666 | 0.094253 | 0 |
| hsa04350 | 0.007663817 | 0.074199 | 0.032353 |
| hsa04370 | 0.074984276 | 0.53197 | 0.30042 |
| hsa04371 | 0.049593515 | 0.117644 | 0.144118 |
| hsa04380 | 0.061473596 | 0.359339 | 0.529412 |
| hsa04510 | 0.054019427 | 0.155212 | 0.408669 |
| hsa04520 | 0.168627268 | 0.203108 | 0.113235 |
| hsa04540 | 0.037247548 | 0.175653 | 0.161765 |
| hsa04550 | 0.023270982 | 0.071843 | 0.143791 |
| hsa04610 | 0.690186121 | 0.123083 | 0.083193 |
| hsa04611 | 0.204733397 | 0.118576 | 0.194118 |
| hsa04613 | 0.063337138 | 0.072426 | 0.215686 |
| hsa04620 | 0.021174497 | 0.387887 | 0.408669 |
| hsa04621 | 0.019310955 | 0.153617 | 0.371107 |
| hsa04622 | 0.013021501 | 0.282759 | 0.174706 |
| hsa04623 | 0 | 0.198978 | 0.083193 |
| hsa04625 | 0.044002888 | 0.48939 | 0.481074 |
| hsa04630 | 0.035849892 | 0.150106 | 0.237255 |
| hsa04650 | 0.020242726 | 0.147495 | 0.172549 |
| hsa04657 | 0.06939365 | 0.60763 | 0.489176 |
| hsa04658 | 0.020242726 | 0.336032 | 0.284706 |
| hsa04659 | 0.047962916 | 0.423697 | 0.419048 |
| hsa04660 | 0.028628666 | 0.438638 | 0.419048 |
| hsa04662 | 0.02140744 | 0.356322 | 0.323529 |
| hsa04664 | 0.025367467 | 0.371467 | 0.215686 |
| hsa04666 | 0.025367467 | 0.177817 | 0.194118 |
| hsa04668 | 0.054718256 | 0.494828 | 0.516353 |
| hsa04670 | 0.051457057 | 0.090946 | 0.080882 |
| hsa04672 | 0.017913299 | 0.175041 | 0.006471 |
| hsa04713 | 0.126464628 | 0.123403 | 0.129412 |
| hsa04720 | 0.123669314 | 0.220861 | 0.129412 |
| hsa04722 | 0.027231009 | 0.282759 | 0.371107 |
| hsa04723 | 0.145565934 | 0.084063 | 0.194118 |
| hsa04724 | 0.129958769 | 0.090946 | 0.129412 |
| hsa04725 | 0.375713387 | 0.186003 | 0.258824 |
| hsa04726 | 0.394348808 | 0.181129 | 0.388235 |
| hsa04728 | 0.197046286 | 0.18565 | 0.323529 |
| hsa04730 | 0.127163456 | 0.21364 | 0.097059 |
| hsa04750 | 0.023969811 | 0.148112 | 0.161765 |
| hsa04910 | 0.18143912 | 0.175434 | 0.30042 |
| hsa04911 | 0.112022176 | 0.059182 | 0.032353 |
| hsa04912 | 0.032122807 | 0.331405 | 0.308431 |
| hsa04913 | 0.2336183 | 0.423214 | 0.135882 |
| hsa04914 | 0.261571432 | 0.196046 | 0.205882 |
| hsa04915 | 0.440937362 | 0.269099 | 0.383127 |
| hsa04916 | 0.059144168 | 0.140913 | 0.161765 |
| hsa04917 | 0.06520068 | 0.433563 | 0.30042 |
| hsa04918 | 0.072654849 | 0.081686 | 0.025882 |
| hsa04919 | 0.190290946 | 0.254716 | 0.388235 |
| hsa04920 | 0.030026323 | 0.32647 | 0.185294 |
| hsa04921 | 0.042605232 | 0.179937 | 0.363971 |
| hsa04923 | 0.259242004 | 0.229845 | 0.083193 |
| hsa04924 | 0.102937408 | 0.096985 | 0.025882 |
| hsa04925 | 0.065899508 | 0.067323 | 0.064706 |
| hsa04926 | 0.05844534 | 0.314907 | 0.414118 |
| hsa04929 | 0.00114142 | 0.194397 | 0.083193 |
| hsa04930 | 0.024668639 | 0.422089 | 0.143791 |
| hsa04931 | 0.127862284 | 0.345594 | 0.402614 |
| hsa04932 | 0.077080761 | 0.297353 | 0.481074 |
| hsa04933 | 0.060541825 | 0.697471 | 0.672941 |
| hsa04934 | 0.082205502 | 0.127089 | 0.291176 |
| hsa04940 | 0.00370379 | 0.273991 | 0.021569 |
| hsa04960 | 1 | 0.333706 | 0.064706 |
| hsa04961 | 0.097579725 | 0.104923 | 0 |
| hsa04970 | 0.180507349 | 0.07601 | 0.053922 |
| hsa04971 | 0.538773323 | 0.079371 | 0.025882 |
| hsa04976 | 0.585361877 | 0.05401 | 0.025882 |
| hsa05010 | 0.16792844 | 0.077395 | 0.566176 |
| hsa05012 | 0.122970486 | 0.032932 | 0.237255 |
| hsa05014 | 0.170956696 | 0 | 0.223982 |
| hsa05016 | 0.190523888 | 0.000616 | 0.205882 |
| hsa05017 | 0.127629341 | 0.034933 | 0.097059 |
| hsa05020 | 0.079876074 | 0.11842 | 0.476471 |
| hsa05022 | 0.261571432 | 0.072426 | 0.70098 |
| hsa05030 | 0.27321857 | 0.282759 | 0.097059 |
| hsa05031 | 0.224300589 | 0.288223 | 0.194118 |
| hsa05120 | 0.015117986 | 0.32046 | 0.226471 |
| hsa05130 | 0.064967737 | 0.18707 | 0.445238 |
| hsa05131 | 0.028861609 | 0.15249 | 0.561253 |
| hsa05132 | 0.020242726 | 0.138919 | 0.502941 |
| hsa05133 | 0.038645205 | 0.496068 | 0.296886 |
| hsa05134 | 0.012555615 | 0.322444 | 0.125817 |
| hsa05135 | 0.02816278 | 0.291014 | 0.414118 |
| hsa05140 | 0.064268909 | 0.522675 | 0.301961 |
| hsa05142 | 0.036315777 | 0.449087 | 0.366667 |
| hsa05143 | 0.026532181 | 0.690339 | 0.102941 |
| hsa05144 | 0.050292343 | 0.486345 | 0.102941 |
| hsa05145 | 0.048428801 | 0.424138 | 0.476471 |
| hsa05146 | 0.051224114 | 0.267974 | 0.184874 |
| hsa05152 | 0.044235831 | 0.257625 | 0.485294 |
| hsa05160 | 0.039576976 | 0.359602 | 0.606318 |
| hsa05161 | 0.065433623 | 0.5085 | 0.86566 |
| hsa05162 | 0.038878148 | 0.380402 | 0.570706 |
| hsa05163 | 0.050292343 | 0.316271 | 0.859664 |
| hsa05164 | 0.020708612 | 0.258647 | 0.454348 |
| hsa05165 | 0.092687927 | 0.12102 | 0.578758 |
| hsa05166 | 0.104102122 | 0.303417 | 0.682353 |
| hsa05167 | 0.055417084 | 0.356989 | 0.852941 |
| hsa05168 | 0.024901582 | 0.001136 | 0.327124 |
| hsa05169 | 0.05169 | 0.27156 | 0.665546 |
| hsa05170 | 0.059842997 | 0.229408 | 0.629638 |
| hsa05171 | 0.081972559 | 0.201506 | 0.547511 |
| hsa05202 | 0.113652776 | 0.166906 | 0.332043 |
| hsa05203 | 0.091756156 | 0.138607 | 0.427778 |
| hsa05205 | 0.108062149 | 0.291954 | 0.672941 |
| hsa05206 | 0.100840923 | 0.127089 | 0.657014 |
| hsa05207 | 0.364066249 | 0.279202 | 0.72902 |
| hsa05210 | 0.034685178 | 0.550174 | 0.497619 |
| hsa05211 | 0.009061474 | 0.17348 | 0.097059 |
| hsa05212 | 0.023736868 | 0.634967 | 0.497619 |
| hsa05213 | 0.01884507 | 0.542767 | 0.277311 |
| hsa05214 | 0.013021501 | 0.398375 | 0.30042 |
| hsa05215 | 0.093619698 | 0.585923 | 0.597882 |
| hsa05216 | 0.047962916 | 0.476359 | 0.113235 |
| hsa05217 | 0.018612127 | 0.073308 | 0 |
| hsa05218 | 0.011856787 | 0.345594 | 0.237255 |
| hsa05219 | 0.042838175 | 1 | 0.346367 |
| hsa05220 | 0.020009784 | 0.530792 | 0.402614 |
| hsa05221 | 0.095017354 | 0.457197 | 0.30042 |
| hsa05222 | 0.06263831 | 0.47946 | 0.465882 |
| hsa05223 | 0.020941555 | 0.528863 | 0.395848 |
| hsa05224 | 0.208460481 | 0.2289 | 0.402614 |
| hsa05225 | 0.079876074 | 0.251341 | 0.476471 |
| hsa05226 | 0.057280626 | 0.20685 | 0.346367 |
| hsa05230 | 0.008595588 | 0.245057 | 0.161765 |
| hsa05231 | 0.014885043 | 0.255829 | 0.291176 |
| hsa05235 | 0.031191036 | 0.409841 | 0.395848 |
| hsa05310 | 0.016515642 | 0.246274 | 0 |
| hsa05320 | 0.022106268 | 0.154717 | 0.006471 |
| hsa05321 | 0.023270982 | 0.392962 | 0.107843 |
| hsa05323 | 0.024901582 | 0.217896 | 0.102941 |
| hsa05330 | 0.021174497 | 0.391894 | 0.013866 |
| hsa05332 | 0.004635561 | 0.345594 | 0.027731 |
| hsa05415 | 0.0870973 | 0.152755 | 0.357585 |
| hsa05416 | 0.022339211 | 0.21364 | 0.069328 |
| hsa05417 | 0.126930513 | 0.445838 | 1 |
| hsa05418 | 0.084767873 | 0.43736 | 0.582353 |

**
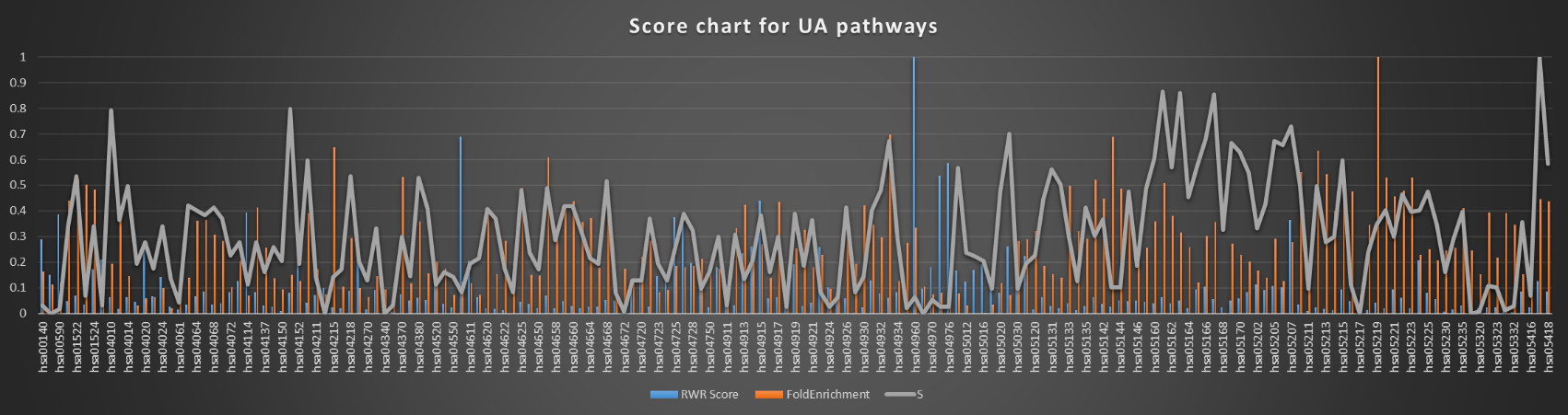
**

**Figure S2 Score chart of the pathways for unstable angina pectoris**

**Table S11 Duplication of pathways**

| SA-Exclusive pathways shared by S, D, F and T | whether it is present in miRNA functional enrichment | UA-Exclusive pathways shared by S, D, F and T | whether it is present in miRNA functional enrichment |
| --- | --- | --- | --- |
| hsa04020 | 1 | hsa05210 | 1 |
| hsa04024 | 1 | hsa04659 | 1 |
| hsa05224 | 1 | hsa04930 | 0 |
| hsa04970 | 0 | hsa04664 | 1 |
| hsa00140 | 0 | hsa04064 | 1 |
| hsa04919 | 1 | hsa05134 | 1 |
| hsa04022 | 1 | hsa04071 | 1 |
| hsa04611 | 0 | hsa04722 | 1 |
| hsa04728 | 1 | hsa04137 | 1 |
| hsa05204 | 0 | hsa04919 | 1 |
| hsa04270 | 1 | hsa05225 | 1 |
| hsa04724 | 1 | hsa05230 | 1 |
| hsa05020 | 1 | hsa05224 | 1 |
| hsa05225 | 1 | hsa05226 | 1 |
| hsa04072 | 1 | hsa04929 | 0 |
| hsa04927 | 0 | hsa04728 | 1 |
| hsa04360 | 1 | hsa04666 | 1 |
| hsa05226 | 0 | hsa00140 | 0 |
| hsa04064 | 1 | hsa04750 | 1 |
| hsa04929 | 1 | hsa04062 | 1 |
| hsa04922 | 0 | hsa04140 | 1 |
| hsa04659 | 1 | hsa04611 | 1 |
| hsa04613 | 1 | hsa05020 | 0 |
| hsa04670 | 1 | hsa04217 | 0 |
| hsa04217 | 1 | hsa04072 | 1 |
| hsa04071 | 1 | hsa04024 | 0 |
| hsa04930 | 1 | hsa04340 | 1 |
| hsa05210 | 1 | hsa04724 | 0 |
| hsa04137 | 1 | hsa04670 | 1 |
| hsa04062 | 1 | hsa04970 | 0 |
| hsa04722 | 1 | hsa04213 | 1 |
| hsa04140 | 1 | hsa05217 | 1 |
| hsa04664 | 1 | hsa04613 | 0 |
| hsa04750 | 1 | hsa04270 | 0 |
| hsa04666 | 1 | hsa04022 | 1 |
| hsa05134 | 1 | hsa04020 | 0 |
| hsa05230 | 1 | hsa04911 | 0 |
|  |  | hsa05017 | 1 |

**Part 6 contains Table S12 and Table S13.**

**Table S12 Upregulated compounds and selected upregulated compounds for stable angina**

| Mol_ID | Name | Ranking of importance | Mol_ID | Name | The ranking of importance is greater than the median 4 |
| --- | --- | --- | --- | --- | --- |
| MOL004624 | Longikaurin A | unique | MOL004624 | Longikaurin A | unique |
| MOL004814 | Isotrifoliol | 15 | MOL004814 | Isotrifoliol | 15 |
| MOL004914 | 1,3-dihydroxy-8,9-dimethoxy-6-benzofurano[3,2-c]chromenone | 14 | MOL004914 | 1,3-dihydroxy-8,9-dimethoxy-6-benzofurano[3,2-c]chromenone | 14 |
| MOL004990 | 7,2',4'-trihydroxy－5-methoxy-3－arylcoumarin | 12 | MOL004990 | 7,2',4'-trihydroxy－5-methoxy-3－arylcoumarin | 12 |
| MOL005017 | Phaseol | 12 | MOL005017 | Phaseol | 12 |
| MOL004907 | Glyzaglabrin | 11 | MOL004907 | Glyzaglabrin | 11 |
| MOL004913 | 1,3-dihydroxy-9-methoxy-6-benzofurano[3,2-c]chromenone | 10 | MOL004913 | 1,3-dihydroxy-9-methoxy-6-benzofurano[3,2-c]chromenone | 10 |
| MOL004966 | 3'-Hydroxy-4'-O-Methylglabridin | 9 | MOL004966 | 3'-Hydroxy-4'-O-Methylglabridin | 9 |
| MOL004824 | (2S)-6-(2,4-dihydroxyphenyl)-2-(2-hydroxypropan-2-yl)-4-methoxy-2,3-dihydrofuro[3,2-g]chromen-7-one | 9 | MOL004824 | (2S)-6-(2,4-dihydroxyphenyl)-2-(2-hydroxypropan-2-yl)-4-methoxy-2,3-dihydrofuro[3,2-g]chromen-7-one | 9 |
| MOL004863 | 3-(3,4-dihydroxyphenyl)-5,7-dihydroxy-8-(3-methylbut-2-enyl)chromone | 9 | MOL004863 | 3-(3,4-dihydroxyphenyl)-5,7-dihydroxy-8-(3-methylbut-2-enyl)chromone | 9 |
| MOL004898 | (E)-3-[3,4-dihydroxy-5-(3-methylbut-2-enyl)phenyl]-1-(2,4-dihydroxyphenyl)prop-2-en-1-one | 9 | MOL004898 | (E)-3-[3,4-dihydroxy-5-(3-methylbut-2-enyl)phenyl]-1-(2,4-dihydroxyphenyl)prop-2-en-1-one | 9 |
| MOL000490 | petunidin | 9 | MOL000490 | petunidin | 9 |
| MOL004841 | Licochalcone B | 8 | MOL004841 | Licochalcone B | 8 |
| MOL002311 | Glycyrol | 8 | MOL002311 | Glycyrol | 8 |
| MOL004810 | glyasperin F | 7 | MOL004810 | glyasperin F | 7 |
| MOL004989 | 6-prenylated eriodictyol | 7 | MOL004989 | 6-prenylated eriodictyol | 7 |
| MOL006992 | (2R,3R)-4-methoxyl-distylin | 7 | MOL006992 | (2R,3R)-4-methoxyl-distylin | 7 |
| MOL004849 | 3-(2,4-dihydroxyphenyl)-8-(1,1-dimethylprop-2-enyl)-7-hydroxy-5-methoxy-coumarin | 6 | MOL004849 | 3-(2,4-dihydroxyphenyl)-8-(1,1-dimethylprop-2-enyl)-7-hydroxy-5-methoxy-coumarin | 6 |
| MOL003656 | Lupiwighteone | 6 | MOL003656 | Lupiwighteone | 6 |
| MOL005000 | Gancaonin G | 6 | MOL005000 | Gancaonin G | 6 |
| MOL004827 | Semilicoisoflavone B | 6 | MOL004827 | Semilicoisoflavone B | 6 |
| MOL004948 | Isoglycyrol | 6 | MOL004948 | Isoglycyrol | 6 |
| MOL001458 | coptisine | 6 | MOL001458 | coptisine | 6 |
| MOL004835 | Glypallichalcone | 5 | MOL004835 | Glypallichalcone | 5 |
| MOL004883 | Licoisoflavone | 5 | MOL004883 | Licoisoflavone | 5 |
| MOL005020 | dehydroglyasperins C | 5 | MOL005020 | dehydroglyasperins C | 5 |
| MOL004856 | Gancaonin A | 5 | MOL004856 | Gancaonin A | 5 |
| MOL004949 | Isolicoflavonol | 5 | MOL004949 | Isolicoflavonol | 5 |
| MOL004904 | licopyranocoumarin | 5 | MOL004904 | licopyranocoumarin | 5 |
| MOL004935 | Sigmoidin-B | 5 | MOL004935 | Sigmoidin-B | 5 |
| MOL004961 | Quercetin der. | 4 |  |  |  |
| MOL004848 | licochalcone G | 4 |  |  |  |
| MOL004910 | Glabranin | 4 |  |  |  |
| MOL001368 | 3-O-p-coumaroylquinic acid | 4 |  |  |  |
| MOL005008 | Glycyrrhiza flavonol A | 4 |  |  |  |
| MOL005001 | Gancaonin H | 4 |  |  |  |
| MOL002695 | lignan | 4 |  |  |  |
| MOL013187 | Cubebin | 4 |  |  |  |
| MOL004903 | liquiritin | 4 |  |  |  |
| MOL001355 | GA63 | 4 |  |  |  |
| MOL000497 | licochalcone a | 3 |  |  |  |
| MOL000354 | isorhamnetin | 3 |  |  |  |
| MOL000417 | Calycosin | 3 |  |  |  |
| MOL004864 | 5,7-dihydroxy-3-(4-methoxyphenyl)-8-(3-methylbut-2-enyl)chromone | 3 |  |  |  |
| MOL004808 | glyasperin B | 3 |  |  |  |
| MOL002757 | 7,8-dimethyl-1H-pyrimido[5,6-g]quinoxaline-2,4-dione | 3 |  |  |  |
| MOL003847 | Inophyllum E | 3 |  |  |  |
| MOL004866 | 2-(3,4-dihydroxyphenyl)-5,7-dihydroxy-6-(3-methylbut-2-enyl)chromone | 3 |  |  |  |
| MOL002712 | 6-Hydroxykaempferol | 3 |  |  |  |
| MOL001328 | 2,3-didehydro GA70 | 3 |  |  |  |
| MOL002157 | wallichilide | 3 |  |  |  |
| MOL004957 | HMO | 2 |  |  |  |
| MOL004884 | Licoisoflavone B | 2 |  |  |  |
| MOL004855 | Licoricone | 2 |  |  |  |
| MOL004882 | Licocoumarone | 2 |  |  |  |
| MOL003896 | 7-Methoxy-2-methyl isoflavone | 1 |  |  |  |
| MOL004598 | 3,5,6,7-tetramethoxy-2-(3,4,5-trimethoxyphenyl)chromone | 1 |  |  |  |
| MOL005018 | Xambioona | 1 |  |  |  |
| MOL001352 | GA54 | 1 |  |  |  |
| MOL001329 | 2,3-didehydro GA77 | 1 |  |  |  |
| MOL002710 | Pyrethrin II | 1 |  |  |  |
| MOL002717 | qt_carthamone | 1 |  |  |  |
| MOL001353 | GA60 | 1 |  |  |  |
| MOL001360 | GA77 | 1 |  |  |  |
| MOL001342 | GA121-isolactone | 1 |  |  |  |
| MOL001344 | GA122-isolactone | 1 |  |  |  |
| MOL001349 | 4a-formyl-7alpha-hydroxy-1-methyl-8-methylidene-4aalpha,4bbeta-gibbane-1alpha,10beta-dicarboxylic acid | 1 |  |  |  |

**Table S13 Upregulated compounds and selected upregulated compounds for unstable angina**

| Mol_ID | Name | Ranking of importance | Mol_ID | Name | The ranking of importance is greater than the median 5 |
| --- | --- | --- | --- | --- | --- |
| MOL004829 | Glepidotin B | 21 | MOL004829 | Glepidotin B | 21 |
| MOL005007 | Glyasperins M | 20 | MOL005007 | Glyasperins M | 20 |
| MOL004833 | Phaseolinisoflavan | 19 | MOL004833 | Phaseolinisoflavan | 19 |
| MOL001484 | Inermine | 19 | MOL001484 | Inermine | 19 |
| MOL004580 | cis-Dihydroquercetin | 19 | MOL004580 | cis-Dihydroquercetin | 19 |
| MOL000785 | palmatine | 14 | MOL000785 | palmatine | 14 |
| MOL001002 | ellagic acid | 13 | MOL001002 | ellagic acid | 13 |
| MOL004838 | 8-(6-hydroxy-2-benzofuranyl)-2,2-dimethyl-5-chromenol | 13 | MOL004838 | 8-(6-hydroxy-2-benzofuranyl)-2,2-dimethyl-5-chromenol | 13 |
| MOL001454 | berberine | 12 | MOL001454 | berberine | 12 |
| MOL001340 | GA120 | 12 | MOL001340 | GA120 | 12 |
| MOL004820 | kanzonols W | 10 | MOL004820 | kanzonols W | 10 |
| MOL004815 | (E)-1-(2,4-dihydroxyphenyl)-3-(2,2-dimethylchromen-6-yl)prop-2-en-1-one | 10 | MOL004815 | (E)-1-(2,4-dihydroxyphenyl)-3-(2,2-dimethylchromen-6-yl)prop-2-en-1-one | 10 |
| MOL005016 | Odoratin | 9 | MOL005016 | Odoratin | 9 |
| MOL002897 | epiberberine | 8 | MOL002897 | epiberberine | 8 |
| MOL002140 | Perlolyrine | 8 | MOL002140 | Perlolyrine | 8 |
| MOL004911 | Glabrene | 7 | MOL004911 | Glabrene | 7 |
| MOL001358 | gibberellin 7 | 7 | MOL001358 | gibberellin 7 | 7 |
| MOL004991 | 7-Acetoxy-2-methylisoflavone | 6 | MOL004991 | 7-Acetoxy-2-methylisoflavone | 6 |
| MOL000492 | (+)-catechin | 6 | MOL000492 | (+)-catechin | 6 |
| MOL002565 | Medicarpin | 5 |  |  |  |
| MOL004959 | 1-Methoxyphaseollidin | 5 |  |  |  |
| MOL005003 | Licoagrocarpin | 5 |  |  |  |
| MOL002135 | Myricanone | 5 |  |  |  |
| MOL004974 | 3'-Methoxyglabridin | 5 |  |  |  |
| MOL004811 | Glyasperin C | 5 |  |  |  |
| MOL004912 | Glabrone | 5 |  |  |  |
| MOL004941 | (2R)-7-hydroxy-2-(4-hydroxyphenyl)chroman-4-one | 5 |  |  |  |
| MOL004908 | Glabridin | 4 |  |  |  |
| MOL004609 | Areapillin | 4 |  |  |  |
| MOL001689 | acacetin | 3 |  |  |  |
| MOL004891 | shinpterocarpin | 3 |  |  |  |
| MOL004857 | Gancaonin B | 3 |  |  |  |
| MOL002694 | 4-[(E)-4-(3,5-dimethoxy-4-oxo-1-cyclohexa-2,5-dienylidene)but-2-enylidene]-2,6-dimethoxycyclohexa-2,5-dien-1-one | 3 |  |  |  |
| MOL002714 | baicalein | 2 |  |  |  |
| MOL000500 | Vestitol | 2 |  |  |  |
| MOL004828 | Glepidotin A | 2 |  |  |  |
| MOL004915 | Eurycarpin A | 2 |  |  |  |
| MOL005012 | Licoagroisoflavone | 2 |  |  |  |
| MOL001792 | DFV | 2 |  |  |  |
| MOL004879 | Glycyrin | 2 |  |  |  |
| MOL004993 | 8-prenylated eriodictyol | 2 |  |  |  |
| MOL000422 | kaempferol | 1 |  |  |  |
| MOL005828 | nobiletin | 1 |  |  |  |
| MOL000392 | formononetin | 1 |  |  |  |
| MOL002341 | Hesperetin | 1 |  |  |  |
| MOL004980 | Inflacoumarin A | 1 |  |  |  |
| MOL004945 | (2S)-7-hydroxy-2-(4-hydroxyphenyl)-8-(3-methylbut-2-enyl)chroman-4-one | 1 |  |  |  |

**Part 7 contains Table S14 and Table S15.**

**Table S14 Results of molecular docking (kcal/mol)**

| Target |  | Random | Stable | Unstable | Selected Stable | Selected Unstable |
| --- | --- | --- | --- | --- | --- | --- |
| NCF1 | UA | -7.6840 | -7.8045 | -7.7575 | -7.9033 | -7.9211 |
| ACACA | SA | -8.3760 | -8.1866 | -8.0255 | -8.2933 | -8.1842 |
| ACP3 | SA | -8.7340 | -8.4134 | -8.3213 | -8.4667 | -8.3053 |
| ADRA1A | SA | -7.4520 | -7.2284 | -7.3447 | -7.2567 | -7.2474 |
| ADRA1B | SA | -7.7980 | -7.3418 | -7.2894 | -7.3500 | -7.2579 |
| ADRA1D | SA | -7.7880 | -7.2910 | -7.4787 | -7.2700 | -7.5105 |
| APOD | SA | -8.8180 | -8.9418 | -8.8809 | -8.8767 | -8.9790 |
| ATP5F1B | SA | -7.3200 | -7.4164 | -7.3915 | -7.4933 | -7.4790 |
| CASP7 | SA | -7.8120 | -8.1194 | -8.0383 | -8.2733 | -7.9842 |
| CASP8 | SA | -7.2520 | -7.3627 | -7.2319 | -7.3400 | -7.3737 |
| CHRM1 | SA | -8.4260 | -8.1537 | -8.2128 | -8.2000 | -8.3105 |
| CHRM2 | SA | -5.9640 | -5.7746 | -5.9085 | -5.8600 | -5.9421 |
| DGAT1 | SA | -9.0880 | -8.8836 | -8.8489 | -9.0067 | -8.9842 |
| DUOX2 | SA | -7.8420 | -7.7508 | -7.9000 | -7.8067 | -7.8842 |
| E2F2 | SA | -8.4160 | -8.0433 | -8.1872 | -8.1900 | -8.3000 |
| EPHB2 | SA | -7.7000 | -7.6522 | -7.6596 | -7.7300 | -7.6790 |
| ESR2 | SA | -7.3740 | -7.5269 | -7.7426 | -7.3867 | -8.2263 |
| GSTM1 | SA | -7.3580 | -7.1209 | -7.1064 | -7.1367 | -7.1579 |
| HK2 | SA | -8.2120 | -8.3388 | -8.2596 | -8.4200 | -8.2684 |
| HSD3B1 | SA | -8.2320 | -8.7224 | -8.6532 | -8.7500 | -8.7421 |
| MET | SA | -8.8200 | -9.0970 | -9.0106 | -9.1800 | -9.0947 |
| MT-ND6 | SA | -7.7020 | -7.4925 | -7.5766 | -7.6400 | -7.5579 |
| NCOA1 | SA | -8.1740 | -8.0970 | -8.1383 | -8.2100 | -8.0790 |
| NFATC1 | SA | -7.4220 | -7.4090 | -7.2936 | -7.5500 | -7.2790 |
| NKX3-1 | SA | -6.6500 | -6.5642 | -6.6872 | -6.6300 | -6.8158 |
| NOX5 | SA | -6.6240 | -6.8791 | -6.8766 | -6.9767 | -6.9474 |
| NPEPPS | SA | -8.8740 | -8.5015 | -8.4192 | -8.6567 | -8.5053 |
| PDE3A | SA | -8.6100 | -8.5045 | -8.3128 | -8.6100 | -8.5053 |
| PREP | SA | -8.2680 | -7.8672 | -7.9362 | -7.9267 | -8.0474 |
| PYGM | SA | -8.1840 | -8.2851 | -8.3489 | -8.5500 | -8.5053 |
| RASA1 | SA | -7.3320 | -7.2642 | -7.2404 | -7.2900 | -7.3316 |
| RXRA | SA | -6.8840 | -7.0522 | -7.0234 | -7.1067 | -7.1790 |
| SLC2A4 | SA | -8.2300 | -8.0731 | -8.2128 | -8.2933 | -8.1526 |
| SLPI | SA | -7.0360 | -7.2627 | -7.1830 | -7.5267 | -7.2579 |
| STAT1 | SA | -7.4160 | -7.1254 | -7.0553 | -7.2367 | -7.1579 |

**Table S15 50 random compounds**

| Random compounds | |
| --- | --- |
| TCM-(6aR,11aR)-3-Hydroxy-9,10-dimethoxypterocarpan.mol2 | TCM-31021.mol2 |
| TCM-34239.mol2 | TCM-33469.mol2 |
| TCM-12041.mol2 | TCM-33521.mol2 |
| TCM-1206.mol2 | TCM-3473.mol2 |
| TCM-12145.mol2 | TCM-37726.mol2 |
| TCM-12273.mol2 | TCM-39119.mol2 |
| TCM-13403.mol2 | TCM-39709.mol2 |
| TCM-13530.mol2 | TCM-41572.mol2 |
| TCM-15247.mol2 | TCM-41867.mol2 |
| TCM-1553.mol2 | TCM-43926.mol2 |
| TCM-18317.mol2 | TCM-44441.mol2 |
| TCM-18980.mol2 | TCM-45798.mol2 |
| TCM-20581.mol2 | TCM-46057.mol2 |
| TCM-2096.mol2 | TCM-48190.mol2 |
| TCM-22404.mol2 | TCM-6504.mol2 |
| TCM-23502.mol2 | TCM-8187.mol2 |
| TCM-23511.mol2 | TCM-8577.mol2 |
| TCM-23861.mol2 | TCM-N-Methylephedrine (11).mol2 |
| TCM-24205.mol2 | TCM-tcm03_000017.mol2 |
| TCM-24990.mol2 | TCM-tcm03_002133.mol2 |
| TCM-26031.mol2 | TCM-tcm03_003136.mol2 |
| TCM-26697.mol2 | TCM-tcm03_004891.mol2 |
| TCM-27520.mol2 | TCM-tcm03_005714.mol2 |
| TCM-29752.mol2 | TCM-tcm03_006227.mol2 |
| TCM-30853.mol2 | TCM-tcm03_008001.mol2 |

**Part 8 contains Table S16 and Table S17.**

**Table S16 Significantly differentially expressed miRNAs**

| ID | *p* value | logFC |  |
| --- | --- | --- | --- |
| hsa-miR-765 | 0.0182 | 2.22 | SA |
| hsa-miR-140-3p | 0.024 | -2.27 | SA |
| hsa-miR-599 | 1.32E-09 | 7.277464 | UA |
| hsa-miR-124-3p | 1.39E-09 | -9.08543 | UA |
| hsa-miR-374a-3p | 5.59E-09 | 5.566144 | UA |
| hsa-miR-20b-5p | 7.22E-09 | 6.159008 | UA |
| hsa-miR-522-3p | 1.02E-08 | -4.02745 | UA |
| hsa-miR-3664-5p | 1.65E-08 | 5.007002 | UA |
| hsa-miR-125b-5p | 7.64E-08 | -4.83704 | UA |
| hsa-miR-874 | 1.06E-07 | -4.05352 | UA |
| hsa-miR-363-5p | 1.42E-07 | -5.49455 | UA |
| hsa-miR-183-3p | 1.55E-07 | -4.48607 | UA |
| hsa-miR-4318 | 1.94E-07 | 4.112523 | UA |
| hsa-let-7f-5p | 2.12E-07 | 6.498784 | UA |
| hsa-miR-224-5p | 2.19E-07 | 4.728246 | UA |
| hsa-miR-892a | 2.43E-07 | 3.562029 | UA |
| hsa-miR-376b | 2.92E-07 | -3.41553 | UA |
| hsa-miR-199a-5p | 3.84E-07 | 6.967399 | UA |
| hsa-miR-668 | 3.85E-07 | -3.88086 | UA |
| hsa-miR-433 | 3.95E-07 | -3.96777 | UA |
| hsa-miR-3622b-3p | 4.03E-07 | -4.40459 | UA |
| hsa-miR-23a-3p | 4.05E-07 | 3.345961 | UA |
| hsa-miR-9-5p | 4.42E-07 | -5.3705 | UA |
| hsa-miR-223-3p | 4.94E-07 | 5.986513 | UA |
| hsa-miR-3144-5p | 5.36E-07 | -4.40019 | UA |
| hsa-miR-1827 | 6.24E-07 | 2.650296 | UA |
| hsa-miR-1253 | 6.4E-07 | -4.00447 | UA |
| hsa-miR-124-5p | 7.34E-07 | -3.56316 | UA |
| hsa-miR-1275 | 7.95E-07 | -5.23156 | UA |
| hsa-miR-933 | 8.55E-07 | -4.22668 | UA |
| hsa-miR-138-5p | 8.69E-07 | -4.79568 | UA |
| hsa-miR-938 | 8.88E-07 | -4.52364 | UA |
| hsa-miR-642b-3p | 9.72E-07 | -3.47858 | UA |
| hsa-miR-151a-3p | 9.91E-07 | 4.865114 | UA |
| hsa-miR-371a-3p | 1.09E-06 | 4.262759 | UA |
| hsa-miR-454-3p | 1.55E-06 | 4.200115 | UA |
| hsa-miR-505-3p | 1.59E-06 | 5.048675 | UA |
| hsa-miR-3617 | 0.0000016 | 3.300281 | UA |
| hsa-miR-664-5p | 1.67E-06 | 4.199431 | UA |
| hsa-miR-296-5p | 1.76E-06 | -3.15597 | UA |
| hsa-miR-26b-5p | 1.78E-06 | 4.519493 | UA |
| hsa-miR-148b-3p | 1.96E-06 | 4.360404 | UA |
| hsa-miR-222-3p | 1.98E-06 | 3.606249 | UA |
| hsa-miR-1 | 2.01E-06 | 7.528779 | UA |
| hsa-miR-501-3p | 2.08E-06 | 5.217529 | UA |
| hsa-miR-490-5p | 2.25E-06 | -2.74708 | UA |
| hsa-miR-3942-5p | 2.47E-06 | 2.740508 | UA |
| hsa-miR-1297 | 2.48E-06 | 5.30483 | UA |
| hsa-miR-9-3p | 2.57E-06 | -4.09683 | UA |
| hsa-miR-769-5p | 0.0000026 | 4.556684 | UA |
| hsa-miR-3171 | 2.64E-06 | 2.781642 | UA |
| hsa-miR-101-3p | 0.0000027 | 3.542475 | UA |
| hsa-miR-1269a | 2.74E-06 | 3.065861 | UA |
| hsa-miR-219-2-3p | 3.07E-06 | -4.38968 | UA |
| hsa-miR-30e-5p | 3.54E-06 | 3.412195 | UA |
| hsa-miR-155-3p | 3.71E-06 | 3.201448 | UA |
| hsa-miR-193b-3p | 3.81E-06 | -3.19546 | UA |
| hsa-let-7d-5p | 4.33E-06 | 4.014014 | UA |
| hsa-miR-3613-3p | 4.39E-06 | 2.990642 | UA |
| hsa-miR-4288 | 4.84E-06 | 2.770938 | UA |
| hsa-miR-1224-5p | 4.94E-06 | -2.66383 | UA |
| hsa-miR-3713 | 4.96E-06 | 3.819942 | UA |
| hsa-miR-144-5p | 5.03E-06 | 6.331154 | UA |
| hsa-miR-98 | 5.03E-06 | 4.286989 | UA |
| hsa-miR-130a-3p | 5.09E-06 | 4.089983 | UA |
| hsa-miR-421 | 5.16E-06 | 6.545545 | UA |
| hsa-miR-545-5p | 5.33E-06 | 2.759293 | UA |
| hsa-miR-4261 | 6.78E-06 | -3.69044 | UA |
| hsa-miR-1915-3p | 7.26E-06 | -2.62333 | UA |
| hsa-miR-218-5p | 8.21E-06 | -4.46063 | UA |
| hsa-miR-490-3p | 9.08E-06 | -3.08571 | UA |
| hsa-miR-548l | 9.29E-06 | 3.150308 | UA |
| hsa-miR-20a-5p | 0.0000109 | 6.838132 | UA |
| hsa-miR-4319 | 0.0000114 | -3.28875 | UA |
| hsa-miR-126-5p | 0.0000128 | 3.880915 | UA |
| hsa-miR-30d-5p | 0.0000128 | 3.144476 | UA |
| hsa-miR-142-5p | 0.000013 | 4.358307 | UA |
| hsa-let-7a-5p | 0.0000135 | 5.736727 | UA |
| hsa-miR-1972 | 0.0000143 | 3.240684 | UA |
| hsa-miR-500a-3p | 0.0000146 | 4.899511 | UA |
| hsa-miR-520c-3p | 0.0000148 | 4.17001 | UA |
| hsa-miR-190b | 0.000016 | 2.07099 | UA |
| hsa-miR-513a-5p | 0.0000164 | -3.71781 | UA |
| hsa-miR-626 | 0.0000165 | 2.203267 | UA |
| hsa-miR-3120-3p | 0.000019 | 3.393377 | UA |
| hsa-miR-196b-5p | 0.0000191 | 2.401814 | UA |
| hsa-miR-652-5p | 0.0000191 | -3.32114 | UA |
| hsa-miR-30a-5p | 0.0000196 | 3.730779 | UA |
| hsa-miR-27a-3p | 0.0000197 | 4.567718 | UA |
| hsa-miR-381 | 0.0000208 | -4.49137 | UA |
| hsa-miR-338-3p | 0.0000214 | -3.32887 | UA |
| hsa-miR-502-3p | 0.0000233 | 5.046799 | UA |
| hsa-miR-494 | 0.0000242 | 4.467145 | UA |
| hsa-miR-593-3p | 0.0000251 | 2.359368 | UA |
| hsa-miR-106b-3p | 0.0000257 | 4.338207 | UA |
| hsa-miR-181b-5p | 0.0000259 | -2.47109 | UA |
| hsa-miR-767-5p | 0.0000271 | -2.7609 | UA |
| hsa-miR-374a-5p | 0.0000276 | 3.220658 | UA |
| hsa-miR-660-5p | 0.0000282 | 3.999246 | UA |
| hsa-miR-3915 | 0.0000303 | -2.76631 | UA |
| hsa-miR-193a-3p | 0.0000347 | -2.95397 | UA |
| hsa-miR-129-2-3p | 0.0000354 | -2.47417 | UA |
| hsa-miR-191-5p | 0.0000361 | 3.442042 | UA |
| hsa-miR-21-5p | 0.0000364 | 6.738678 | UA |
| hsa-miR-28-3p | 0.0000376 | 5.052065 | UA |
| hsa-miR-4301 | 0.0000386 | -2.57627 | UA |
| hsa-miR-135a-3p | 0.0000398 | -2.64645 | UA |
| hsa-miR-585 | 0.0000463 | 4.691766 | UA |
| hsa-miR-671-5p | 0.0000505 | -3.37985 | UA |
| hsa-miR-873-5p | 0.0000508 | -4.79843 | UA |
| hsa-miR-23b-3p | 0.000051 | 4.049999 | UA |
| hsa-miR-129-5p | 0.0000515 | -3.66877 | UA |
| hsa-miR-765 | 0.0000581 | -3.01998 | UA |
| hsa-miR-373-5p | 0.000059 | 3.04736 | UA |
| hsa-miR-3130-3p | 0.0000622 | 4.045224 | UA |
| hsa-miR-3605-5p | 0.0000626 | 3.901332 | UA |
| hsa-miR-3165 | 0.0000683 | -2.60529 | UA |
| hsa-miR-766-3p | 0.0000691 | 3.118543 | UA |
| hsa-miR-374c-5p | 0.0000691 | 2.682082 | UA |
| hsa-miR-525-5p | 0.0000694 | -2.16683 | UA |
| hsa-miR-3186-5p | 0.000071 | 3.657646 | UA |
| hsa-miR-30a-3p | 0.0000731 | -2.56569 | UA |
| hsa-miR-223-5p | 0.0000768 | 2.425979 | UA |
| hsa-miR-103a-3p | 0.0000792 | 2.33628 | UA |
| hsa-miR-455-3p | 0.0000798 | 3.795426 | UA |
| hsa-miR-4262 | 0.0000843 | 3.992737 | UA |
| hsa-miR-140-5p | 0.0000848 | 2.929853 | UA |
| hsa-miR-548w | 0.0000861 | 2.064342 | UA |
| hsa-let-7g-5p | 0.0000873 | 4.933345 | UA |
| hsa-miR-631 | 0.0000879 | -4.75555 | UA |
| hsa-miR-92a-3p | 0.0000932 | 4.259199 | UA |
| hsa-miR-519e-3p | 0.0001 | 3.72937 | UA |
| hsa-miR-148a-3p | 0.000101 | 3.419253 | UA |
| hsa-miR-375 | 0.000106 | -3.70524 | UA |
| hsa-miR-517c-3p | 0.000107 | 2.358845 | UA |
| hsa-miR-3199 | 0.000108 | 6.213335 | UA |
| hsa-miR-106a-5p | 0.00011 | 5.793466 | UA |
| hsa-miR-211-5p | 0.000113 | -2.67274 | UA |
| hsa-miR-3146 | 0.000117 | -2.95998 | UA |
| hsa-miR-330-5p | 0.000121 | -2.43661 | UA |
| hsa-miR-3184-5p | 0.000122 | -2.30019 | UA |
| hsa-miR-3186-3p | 0.000123 | -2.61751 | UA |
| hsa-miR-3166 | 0.000126 | -4.65455 | UA |
| hsa-miR-340-3p | 0.00013 | 4.144231 | UA |
| hsa-miR-3945 | 0.000134 | -2.79222 | UA |
| hsa-miR-1301 | 0.000136 | 3.776813 | UA |
| hsa-miR-1276 | 0.000137 | 2.27298 | UA |
| hsa-miR-152 | 0.000141 | 4.637148 | UA |
| hsa-miR-27b-5p | 0.000143 | 4.813232 | UA |
| hsa-miR-3125 | 0.000147 | -3.72273 | UA |
| hsa-miR-711 | 0.000153 | -2.47701 | UA |
| hsa-miR-3714 | 0.000163 | -2.04187 | UA |
| hsa-miR-675-3p | 0.000169 | 4.26963 | UA |
| hsa-miR-17-5p | 0.000177 | 5.977747 | UA |
| hsa-miR-411-3p | 0.000178 | -2.32631 | UA |
| hsa-miR-429 | 0.000179 | 2.553227 | UA |
| hsa-miR-29a-3p | 0.000181 | -3.27557 | UA |
| hsa-miR-27b-3p | 0.000196 | 4.049201 | UA |
| hsa-miR-26a-5p | 0.000207 | 3.87878 | UA |
| hsa-miR-147b | 0.00022 | 3.007215 | UA |
| hsa-miR-93-5p | 0.000232 | 5.292935 | UA |
| hsa-miR-940 | 0.000236 | -2.20682 | UA |
| hsa-miR-4310 | 0.000239 | 3.606403 | UA |
| hsa-miR-516b-5p | 0.000242 | -2.38072 | UA |
| hsa-miR-548c-3p | 0.000243 | 3.313706 | UA |
| hsa-miR-320e | 0.000251 | 2.943595 | UA |
| hsa-miR-4295 | 0.00026 | 2.379462 | UA |
| hsa-miR-200c-3p | 0.00027 | 3.084029 | UA |
| hsa-miR-423-3p | 0.000272 | 3.007798 | UA |
| hsa-miR-320b | 0.000284 | 3.503816 | UA |
| hsa-miR-3913-5p | 0.000305 | 3.376473 | UA |
| hsa-miR-451a | 0.000321 | 2.244833 | UA |
| hsa-miR-301a-3p | 0.000323 | 4.299021 | UA |
| hsa-miR-29c-3p | 0.000332 | -3.82604 | UA |
| hsa-miR-1914-5p | 0.000384 | -2.07176 | UA |
| hsa-miR-224-3p | 0.000388 | -2.29317 | UA |
| hsa-miR-3610 | 0.000391 | -3.82441 | UA |
| hsa-miR-132-3p | 0.000419 | -2.67265 | UA |
| hsa-miR-29b-3p | 0.000449 | -3.86175 | UA |
| hsa-miR-374c-3p | 0.000473 | 2.350559 | UA |
| hsa-miR-1227 | 0.000476 | -2.40186 | UA |
| hsa-miR-486-5p | 0.000494 | 2.747329 | UA |
| hsa-miR-323a-3p | 0.000505 | -3.17686 | UA |
| hsa-miR-3191-3p | 0.000522 | 2.319764 | UA |
| hsa-miR-195-3p | 0.000536 | -2.21588 | UA |
| hsa-miR-4258 | 0.00054 | -2.28854 | UA |
| hsa-miR-561-3p | 0.000542 | 2.292185 | UA |
| hsa-let-7i-5p | 0.000556 | 3.703707 | UA |
| hsa-miR-1912 | 0.000587 | 2.0665 | UA |
| hsa-miR-671-3p | 0.000598 | 3.007858 | UA |
| hsa-miR-3690 | 0.000599 | 3.045262 | UA |
| hsa-miR-744-5p | 0.000624 | 3.226487 | UA |
| hsa-miR-1273e | 0.000637 | 3.170564 | UA |
| hsa-miR-3927 | 0.000643 | 2.589227 | UA |
| hsa-miR-3134 | 0.000648 | 2.292826 | UA |
| hsa-miR-1913 | 0.00072 | -2.34361 | UA |
| hsa-miR-887 | 0.000724 | -3.29861 | UA |
| hsa-miR-3668 | 0.000803 | -2.47601 | UA |
| hsa-miR-361-5p | 0.000813 | 3.067664 | UA |
| hsa-miR-4311 | 0.000817 | -2.50453 | UA |
| hsa-miR-4275 | 0.000819 | -2.54394 | UA |
| hsa-miR-126-3p | 0.000823 | 3.655366 | UA |
| hsa-miR-320c | 0.000835 | 3.896387 | UA |
| hsa-miR-548f | 0.000906 | 2.205451 | UA |
| hsa-miR-583 | 0.000909 | -3.36658 | UA |
| hsa-miR-329 | 0.000929 | 2.431704 | UA |
| hsa-miR-299-5p | 0.000934 | 2.581163 | UA |
| hsa-miR-1286 | 0.000943 | -3.37895 | UA |
| hsa-miR-889 | 0.000986 | 2.85096 | UA |
| hsa-miR-3163 | 0.000986 | 2.82735 | UA |
| hsa-miR-138-2-3p | 0.001 | -2.62454 | UA |
| hsa-miR-3177-3p | 0.00101 | 3.771891 | UA |
| hsa-miR-648 | 0.00103 | -2.62022 | UA |
| hsa-miR-3175 | 0.00112 | -2.84072 | UA |
| hsa-miR-1264 | 0.00113 | -2.31122 | UA |
| hsa-miR-378a-5p | 0.00113 | -2.00437 | UA |
| hsa-miR-431-5p | 0.00113 | 4.767948 | UA |
| hsa-miR-548d-5p | 0.00113 | 2.456074 | UA |
| hsa-miR-410 | 0.00114 | -3.50521 | UA |
| hsa-miR-16-5p | 0.00115 | 3.822627 | UA |
| hsa-miR-146a-5p | 0.0012 | 5.376512 | UA |
| hsa-miR-1245a | 0.0012 | 3.55431 | UA |
| hsa-miR-151a-5p | 0.00122 | 4.109245 | UA |
| hsa-miR-192-5p | 0.00122 | 3.059133 | UA |
| hsa-miR-16-2-3p | 0.00122 | 2.259162 | UA |
| hsa-miR-4317 | 0.00124 | 2.777434 | UA |
| hsa-miR-320d | 0.00136 | 2.819954 | UA |
| hsa-miR-181c-3p | 0.00137 | 2.840821 | UA |
| hsa-miR-4294 | 0.0014 | 5.34146 | UA |
| hsa-miR-154-3p | 0.00142 | -3.18825 | UA |
| hsa-miR-212-3p | 0.00148 | -2.0229 | UA |
| hsa-miR-143-3p | 0.0015 | 4.48114 | UA |
| hsa-miR-140-3p | 0.00155 | 3.00618 | UA |
| hsa-miR-943 | 0.00157 | 4.886329 | UA |
| hsa-miR-675-5p | 0.00168 | -2.86157 | UA |
| hsa-miR-652-3p | 0.00172 | 3.014825 | UA |
| hsa-miR-493-5p | 0.00186 | 3.143302 | UA |
| hsa-miR-301a-5p | 0.0019 | 2.121405 | UA |
| hsa-miR-221-5p | 0.00193 | 2.786548 | UA |
| hsa-miR-1273c | 0.00205 | -2.56312 | UA |
| hsa-miR-3168 | 0.00213 | 7.807531 | UA |
| hsa-miR-18b-5p | 0.00217 | 2.543638 | UA |
| hsa-miR-3152-3p | 0.00228 | 2.334197 | UA |
| hsa-miR-548v | 0.00228 | 3.887682 | UA |
| hsa-miR-935 | 0.00236 | 4.914502 | UA |
| hsa-miR-3612 | 0.00237 | 2.462178 | UA |
| hsa-let-7f-2-3p | 0.00245 | 2.586337 | UA |
| hsa-miR-146b-5p | 0.00254 | 5.112428 | UA |
| hsa-miR-542-5p | 0.00255 | -4.09349 | UA |
| hsa-miR-217 | 0.00261 | -2.12029 | UA |
| hsa-miR-122-3p | 0.00262 | 2.199556 | UA |
| hsa-miR-3148 | 0.00266 | 2.09734 | UA |
| hsa-miR-28-5p | 0.00284 | 4.164671 | UA |
| hsa-miR-1273a | 0.00305 | 2.331051 | UA |
| hsa-miR-133b | 0.00306 | 2.130765 | UA |
| hsa-miR-15b-3p | 0.00332 | 2.928156 | UA |
| hsa-miR-628-3p | 0.00335 | 2.419511 | UA |
| hsa-miR-1185-5p | 0.00335 | -2.5189 | UA |
| hsa-miR-548j | 0.00355 | 3.662919 | UA |
| hsa-miR-552 | 0.00381 | -2.59369 | UA |
| hsa-miR-764 | 0.00413 | -2.07609 | UA |
| hsa-miR-3188 | 0.00419 | 2.392984 | UA |
| hsa-miR-532-5p | 0.00419 | 3.722899 | UA |
| hsa-miR-323b-5p | 0.00422 | 4.144545 | UA |
| hsa-miR-300 | 0.00429 | 2.621265 | UA |
| hsa-miR-3143 | 0.00458 | -3.15273 | UA |
| hsa-miR-3169 | 0.00458 | 4.41573 | UA |
| hsa-miR-596 | 0.0047 | -2.48921 | UA |
| hsa-miR-4330 | 0.00477 | -2.59024 | UA |
| hsa-let-7g-3p | 0.00487 | -3.075 | UA |
| hsa-miR-181c-5p | 0.00488 | 2.058916 | UA |
| hsa-miR-4286 | 0.00499 | -2.79351 | UA |
| hsa-miR-486-3p | 0.00506 | 3.327823 | UA |
| hsa-miR-448 | 0.00506 | -3.29262 | UA |
| hsa-miR-3128 | 0.00516 | 3.955217 | UA |
| hsa-miR-205-5p | 0.00524 | -2.21177 | UA |
| hsa-miR-3605-3p | 0.00547 | 2.221194 | UA |
| hsa-miR-1537 | 0.00563 | 6.582477 | UA |
| hsa-miR-1257 | 0.00632 | -3.44007 | UA |
| hsa-miR-3183 | 0.00639 | 3.233309 | UA |
| hsa-miR-3607-3p | 0.00655 | -4.29405 | UA |
| hsa-miR-2355-5p | 0.00659 | 2.631858 | UA |
| hsa-miR-412 | 0.00689 | -2.21165 | UA |
| hsa-miR-491-3p | 0.00717 | -4.7424 | UA |
| hsa-miR-363-3p | 0.00855 | 2.337652 | UA |
| hsa-miR-3678-5p | 0.00874 | 2.684016 | UA |
| hsa-miR-122-5p | 0.00947 | 3.801336 | UA |
| hsa-miR-888-5p | 0.01 | -2.42148 | UA |
| hsa-miR-515-3p | 0.0102 | -2.99783 | UA |
| hsa-miR-214-5p | 0.0104 | 2.124727 | UA |
| hsa-miR-509-3-5p | 0.0107 | -3.2939 | UA |
| hsa-miR-3545-5p | 0.0108 | 2.758494 | UA |
| hsa-miR-641 | 0.0112 | 2.194348 | UA |
| hsa-miR-3545-3p | 0.0117 | -2.13467 | UA |
| hsa-miR-639 | 0.0117 | -2.02326 | UA |
| hsa-miR-186-5p | 0.0129 | 2.041394 | UA |
| hsa-miR-1299 | 0.013 | 2.184163 | UA |
| hsa-miR-3132 | 0.0136 | 6.718738 | UA |
| hsa-miR-720 | 0.0138 | -3.12504 | UA |
| hsa-miR-200c-5p | 0.0142 | -3.55151 | UA |
| hsa-miR-1181 | 0.0148 | -2.315 | UA |
| hsa-miR-25-3p | 0.0155 | 3.02238 | UA |
| hsa-miR-3140-3p | 0.0163 | 4.397367 | UA |
| hsa-miR-4325 | 0.017 | 2.289844 | UA |
| hsa-miR-1260a | 0.0171 | -2.80252 | UA |
| hsa-miR-663b | 0.0172 | -3.1018 | UA |
| hsa-miR-200b-3p | 0.0177 | 2.477938 | UA |
| hsa-miR-1260b | 0.018 | -3.2593 | UA |
| hsa-miR-1252 | 0.0185 | -2.19405 | UA |
| hsa-miR-30c-2-3p | 0.0193 | -2.42494 | UA |
| hsa-miR-3197 | 0.0193 | -2.59931 | UA |
| hsa-miR-1280 | 0.0197 | -2.74197 | UA |
| hsa-miR-597 | 0.0206 | -3.14332 | UA |
| hsa-miR-1270 | 0.0209 | -3.02153 | UA |
| hsa-miR-96-3p | 0.0212 | 2.572608 | UA |
| hsa-miR-605 | 0.0243 | -2.13723 | UA |
| hsa-miR-574-5p | 0.025 | 2.060856 | UA |
| hsa-miR-15b-5p | 0.0254 | 2.591107 | UA |
| hsa-miR-339-3p | 0.0262 | 3.829058 | UA |
| hsa-miR-3917 | 0.028 | 2.064445 | UA |
| hsa-miR-513c-5p | 0.0281 | -2.51596 | UA |
| hsa-miR-4268 | 0.0299 | 2.537395 | UA |
| hsa-miR-493-3p | 0.03 | 2.146774 | UA |
| hsa-miR-3650 | 0.0325 | 2.603826 | UA |
| hsa-miR-23b-5p | 0.033 | 2.079504 | UA |
| hsa-miR-3147 | 0.0334 | -2.02348 | UA |
| hsa-miR-4321 | 0.0385 | -2.44185 | UA |
| hsa-miR-185-5p | 0.0406 | 2.221121 | UA |
| hsa-miR-625-5p | 0.0425 | 3.88181 | UA |
| hsa-miR-3648 | 0.0437 | -2.94005 | UA |
| hsa-miR-758 | 0.0444 | -2.16662 | UA |
| hsa-miR-346 | 0.0453 | -3.00613 | UA |
| hsa-miR-511 | 0.0471 | 2.414401 | UA |
| hsa-miR-1266 | 0.0472 | 3.606288 | UA |
| hsa-miR-3195 | 0.0489 | -3.3668 | UA |
| hsa-miR-4299 | 0.0489 | -2.01619 | UA |
| hsa-miR-10b-5p | 0.0491 | 2.858776 | UA |
| hsa-miR-382-5p | 0.0492 | -2.17359 | UA |

**Table S17 miRNA functional enrichment**

| SAmiRNA | | UAmiRNA | |
| --- | --- | --- | --- |
| hsa03015 | hsa04672 | hsa05205 | hsa05235 |
| hsa04625 | hsa00510 | hsa04550 | hsa04919 |
| hsa00562 | hsa05110 | hsa05220 | hsa05211 |
| hsa04979 | hsa05010 | hsa04218 | hsa04912 |
| hsa04210 | hsa04380 | hsa05168 | hsa04213 |
| hsa05131 | hsa04650 | hsa04722 | hsa05145 |
| hsa05016 | hsa04961 | hsa05210 | hsa04910 |
| hsa04144 | hsa05417 | hsa04068 | hsa01524 |
| hsa04514 | hsa03460 | hsa04390 | hsa01523 |
| hsa03020 | hsa04810 | hsa05225 | hsa04810 |
| hsa04722 | hsa00240 | hsa04012 | hsa04659 |
| hsa05223 | hsa05134 | hsa05165 | hsa05231 |
| hsa05164 | hsa04120 | hsa05161 | hsa04310 |
| hsa04215 | hsa05022 | hsa04110 | hsa04730 |
| hsa04115 | hsa04370 | hsa04071 | hsa04215 |
| hsa05133 | hsa04978 | hsa05166 | hsa05203 |
| hsa04012 | hsa05416 | hsa05222 | hsa04371 |
| hsa04910 | hsa04723 | hsa05226 | hsa05218 |
| hsa05135 | hsa04929 | hsa01521 | hsa05217 |
| hsa05162 | hsa00010 | hsa01522 | hsa04920 |
| hsa05130 | hsa04932 | hsa04933 | hsa04066 |
| hsa05017 | hsa05165 | hsa05132 | hsa05164 |
| hsa03050 | hsa04920 | hsa05417 | hsa04520 |
| hsa04930 | hsa03010 | hsa05169 | hsa04750 |
| hsa04070 | hsa05230 | hsa04668 | hsa05017 |
| hsa04666 | hsa05218 | hsa05223 | hsa04014 |
| hsa04750 | hsa05220 | hsa05215 | hsa05134 |
| hsa00270 | hsa05100 | hsa05135 | hsa04330 |
| hsa04217 | hsa05140 | hsa04510 | hsa04370 |
| hsa00130 | hsa05412 | hsa05214 | hsa04915 |
| hsa04064 | hsa04721 | hsa04211 | hsa04530 |
| hsa04660 | hsa01521 | hsa05224 | hsa04932 |
| hsa04931 | hsa03018 | hsa05167 | hsa04934 |
| hsa04530 | hsa00983 | hsa05130 | hsa05010 |
| hsa04141 | hsa05152 | hsa04660 | hsa05416 |
| hsa04668 | hsa04360 | hsa05418 | hsa04670 |
| hsa04623 | hsa04911 | hsa05212 | hsa03013 |
| hsa04919 | hsa04512 | hsa04140 | hsa05120 |
| hsa05012 | hsa03013 | hsa04115 | hsa05152 |
| hsa04664 | hsa04727 | hsa04015 | hsa04921 |
| hsa05167 | hsa05410 | hsa04150 | hsa04380 |
| hsa04622 | hsa05032 | hsa03015 | hsa00562 |
| hsa04917 | hsa04062 | hsa05142 | hsa05219 |
| hsa05120 | hsa05323 | hsa04144 | hsa05207 |
| hsa04728 | hsa04350 | hsa05162 | hsa04666 |
| hsa04137 | hsa05414 | hsa05163 | hsa04922 |
| hsa00100 | hsa04010 | hsa04152 | hsa04137 |
| hsa00532 | hsa04060 | hsa04010 | hsa04662 |
| hsa01524 | hsa01522 | hsa04611 | hsa04072 |
| hsa05169 | hsa04510 | hsa05170 | hsa04540 |
| hsa01230 | hsa04061 | hsa04625 | hsa04657 |
| hsa05214 | hsa04914 | hsa04916 | hsa05131 |
| hsa05212 | hsa05415 | hsa04350 | hsa04064 |
| hsa05418 | hsa05203 | hsa05160 | hsa04022 |
| hsa00900 | hsa05205 | hsa04664 | hsa04130 |
| hsa00790 | hsa04974 | hsa04926 | hsa04062 |
| hsa05210 | hsa04620 | hsa05213 | hsa05140 |
| hsa01240 | hsa05206 | hsa05221 | hsa04725 |
| hsa04218 | hsa05170 | hsa04360 | hsa04340 |
| hsa05235 | hsa05166 | hsa04728 | hsa05022 |
| hsa04390 | hsa05145 | hsa04210 | hsa05230 |
| hsa05160 | hsa04724 | hsa04141 | hsa04658 |
| hsa04014 | hsa04726 | hsa04931 | hsa04914 |
| hsa05171 | hsa04152 | hsa04151 | hsa04961 |
| hsa04658 | hsa04110 | hsa04917 | hsa04070 |
| hsa05222 | hsa04080 | hsa05216 | hsa04392 |
| hsa04912 | hsa04926 | hsa04120 | hsa00670 |
| hsa04657 | hsa04068 | hsa04720 | hsa05171 |
| hsa01523 | hsa04270 | hsa04620 |  |
| hsa05225 | hsa04151 |  |  |
| hsa05231 | hsa04915 |  |  |
| hsa00051 | hsa05014 |  |  |
| hsa04933 | hsa04140 |  |  |
| hsa05142 | hsa04550 |  |  |
| hsa05132 | hsa03040 |  |  |
| hsa00250 | hsa05224 |  |  |
| hsa04659 | hsa04072 |  |  |
| hsa04621 | hsa04261 |  |  |
| hsa04066 | hsa04150 |  |  |
| hsa00260 | hsa04934 |  |  |
| hsa05033 | hsa04630 |  |  |
| hsa03440 | hsa05161 |  |  |
| hsa04670 | hsa04310 |  |  |
| hsa01200 | hsa04022 |  |  |
| hsa04940 | hsa04613 |  |  |
| hsa04962 | hsa05202 |  |  |
| hsa05020 | hsa04015 |  |  |
| hsa04071 | hsa04024 |  |  |
| hsa04935 | hsa05163 |  |  |
| hsa02010 | hsa04714 |  |  |
| hsa03420 | hsa04020 |  |  |
| hsa04973 | hsa05168 |  |  |
| hsa00520 | hsa04740 |  |  |
